# The Influence of Omega‐3 Fatty Acids and Probiotics on Hippocampal Inflammation and Glial Cells in a Chronic Anorexia Nervosa Rat Model

**DOI:** 10.1002/eat.24574

**Published:** 2025-10-18

**Authors:** A. C. Thelen, N. Andreani, N. M. Korten, M. Neumann, V. Verspohl, M. van Egmond, L. Kneisel, C. Voelz, L. Blischke, J. F. Baines, J. Baier, J. Schumacher, F. Kiessling, B. Herpertz‐Dahlmann, C. Beyer, L. Keller, J. Seitz, S. Trinh

**Affiliations:** ^1^ Institute of Neuroanatomy Aachen Germany; ^2^ Max Planck Institute for Evolutionary Biology Plön Germany; ^3^ Department of Biology and Biotechnology Charles Darwin Rome Italy; ^4^ Hannover Medical School (MHH) Hannover Germany; ^5^ Guest Group Evolutionary Medicine Max Planck Institute for Evolutionary Biology Plön Germany; ^6^ Section of Evolutionary Medicine, Institue for Experimental Medicine University of Kiel Kiel Germany; ^7^ Institute for Experimental Molecular Imaging RWTH Aachen University Aachen Germany; ^8^ Department of Child and Adolescent Psychiatry, Psychosomatics and Psychotherapy RWTH Aachen University Aachen Germany; ^9^ Department of Child and Adolescent Psychiatry, Psychosomatics and Psychotherapy, LVR‐University Hospital Essen University of Duisburg‐Essen Essen Germany

**Keywords:** anorexia nervosa, fatty acids/omega‐3, hippocampus, microbiome, probiotics

## Abstract

**Objective:**

Anorexia nervosa (AN) is a severe eating disorder associated with brain volume reduction, glial cell loss, microbiome alterations, and dysregulated pro‐inflammatory mechanisms. However, the underlying cellular mechanisms remain inadequately elucidated, and interventions addressing these alterations are lacking.

**Method:**

This study employed a chronic activity‐based anorexia (ABA) rat model to investigate hippocampal glial and neuronal cell alterations, inflammation, and microbial modifications in the gut. Omega‐3 fatty acids and a multi‐strain probiotic were examined as potentially protective agents. Cell count, proliferation, and apoptosis rates of microglia, neurons, astrocytes, and oligodendrocytes were analyzed in the hippocampus. Furthermore, local gene expression of pro‐inflammatory cytokines and microRNA was measured. The hippocampal volume was determined longitudinally using 7‐Tesla MRI scans before and after starvation. Finally, the fecal microbiome was analyzed to identify potential associations with brain and clinical characteristics.

**Results:**

Results confirmed the previously described reduction in astrocytes and newly demonstrated a decrease in oligodendrocytes in the hippocampus. Increased levels of IBA1‐positive cells and pro‐inflammatory cytokines suggest microglial activation in this region. Administering omega‐3 and probiotics to starved animals reduced neuroinflammation and microglial activation in the hippocampus, resulting in significantly increased neuronal cell counts in the omega‐3 group. Microbiome composition was primarily affected by the ABA model, and to a lesser extent by omega‐3 and probiotics.

**Discussion:**

These findings support the involvement of microglial activation in the pathogenesis of AN, potentially relevant to hippocampal re‐learning and thus important for psychotherapy. Further, omega‐3 and probiotics may serve as adjunct therapeutic strategies by modifying inflammation.

**Summary**

- Chronic food restriction increased inflammatory microglia and cytokines while reducing astrocytes, oligodendrocytes, and neurons in the hippocampus.
- Omega‐3 and probiotics reversed the hippocampal inflammation and presented a small yet detectable effect on the microbiome, so both agents might be beneficial additions in the treatment of AN, while the microbiome and microRNAs could mediate this effect.

## 1 Introduction

Anorexia nervosa (AN) is an eating disorder characterized by reduced energy intake, pathological underweight, body image disturbance, and excessive exercise (Herpertz et al. 2018). It primarily manifests in adolescent females, with a lifetime prevalence of 1%–4% (van Eeden et al. 2021) and has the highest mortality rate among mental disorders (Ferrari et al. 2022). Multimodal therapy is widely employed, but only 46% of patients experience full remission, and 25% encounter a chronic course (NICE 2017; Solmi et al. 2024).

The activity‐based anorexia (ABA) model is a well‐established rodent model for studying AN (Routtenberg and Kuznesof 1967; Schalla and Stengel 2019). Our group established a chronic version of the ABA model (≥ 30 days) to portray the protracted course of AN (Frintrop, Trinh, et al. 2018).

Besides psychological impairments, AN is associated with altered gut microbiota and inflammation (Neale and Hudson 2020; Seitz et al. 2020). A volume loss of 7.6% in gray matter has been detected in adolescent patients and reproduced in ABA rodents (Frintrop et al. 2019; Seitz et al. 2018). Among the affected regions, the hippocampus is essential for (re‐)learning, as needed during psychotherapy, and is capable of substantial neurogenesis in the adult mammalian brain (Fares et al. 2019; Moreno‐Jiménez et al. 2021). In patients with acute AN, the hippocampus volume is decreased, which has been associated with neuropsychological dysfunctions like memory loss (Bahnsen et al. 2024; Keeler et al. 2020). This may be linked to glial cell regulation, given their role in feeding‐related circuits (Frintrop et al. 2021; Yang et al. 2015).

Astrocytes support neuronal metabolism, neurotransmitter reuptake, and synaptic plasticity (Trepel 2021). In acute ABA, astrocyte loss was observed in the hippocampus, while astrocyte and oligodendrocyte loss was found in the cortex and corpus callosum in chronic ABA (Frintrop, Liesbrock, et al. 2018; Frintrop et al. 2019; Reyes‐Haro et al. 2016; Verspohl et al. 2025).

Microglia are the central nervous system's resident immune cells (Tay et al. 2017). In a dehydration‐induced AN rodent model, microglial density and protein levels of pro‐inflammatory markers were elevated in the hippocampus, aligning with increased pro‐inflammatory markers in patients with AN (Dalton et al. 2018; Ragu‐Varman et al. 2019; Reyes‐Ortega et al. 2020). The involvement of pro‐inflammatory microRNAs (miRNAs) is also being discussed in this context (Ponomarev et al. 2013; Voelz et al. 2024).

Neuronal cell reduction following ABA does not appear to account for brain volume loss, consistent with volume reversibility in weight‐restored patients (Frintrop et al. 2019; Seitz et al. 2018). However, acute starvation decreased dendritic branching and length of individual neurons in the dorsal hippocampus of rats. Additionally, the neuronal damage marker neurofilament light chain was increased in patients with AN, and brain‐derived neurotrophic factor (BDNF) showed divergent levels (Chowdhury, Barbarich‐Marsteller, et al. 2014; Hellerhoff et al. 2021; Trinh, Keller, et al. 2023).

The microbiome‐gut‐brain axis represents a bidirectional link between the gastrointestinal tract and the CNS (Socała et al. 2021). In AN, microbiota diversity and composition are altered (Seitz, Belheouane, et al. 2019). After weight restoration, the initial gut microbiome was partially restored (Mack et al. 2016; Schulz et al. 2021). Similar gut microbiome alterations were found in the ABA‐model (Breton et al. 2021; Hata et al. 2019; Trinh, Kogel, et al. 2023; Trinh et al. 2021). Therefore, the microbiome‐gut‐brain axis could be the target of novel treatment approaches.

Probiotics, defined as “live microorganisms which […] confer a health benefit on the host”, modulate the gut microbiome and influence neural, hormonal, and especially inflammatory pathways (Castelli et al. 2021; FAO/WHO 2001). Omega‐3 fatty acids (omega‐3) are polyunsaturated fats that control neurotransmission, inflammation, and cell survival in the gut and CNS and influence the microbiome (Bazinet and Layé 2014). Generally, supplementation of omega‐3 reduces pro‐inflammatory cytokines and inflammation (Kavyani et al. 2022; Zendedel et al. 2015). Their administration shows promise in neurological and neuropsychiatric diseases by positively affecting inflammation, neurotransmitters, and food intake (Gao et al. 2023; Goncalves et al. 2005; Mauler et al. 2009; Śliwińska and Jeziorek 2021).

We hypothesized that chronic starvation in an ABA model causes astrocyte and oligodendrocyte but not neuronal reductions, as well as an increased microglial cellular density in the hippocampus. Furthermore, we expected elevated pro‐inflammatory cytokines in the hippocampus. We anticipated anti‐inflammatory effects of the intervention with omega‐3 and probiotics, which would support their potential as add‐on treatments in AN‐therapy.

## 2 Materials and Methods

### 2.1 Animals

The experiments were performed on 60 female, 3‐week‐old Wistar rats (RjHAN:WI, Janvier, Germany). This number of animals was calculated by a power analysis a priori with a type I error of 5% (global, two‐sided) and a type II error of ≤ 20%, aiming for ≥ 80% power. One animal was excluded due to premature death during the acclimatization phase. Rats were housed individually in the specific pathogen‐free animal facility of the University Hospital RWTH Aachen. FELASA guidelines and DIN ISO 9001:2015 certifications were met. The cages had incorporated running wheels and tachometers (BC 5.12, Sigma, Neustadt, Germany). A standard temperature (22°C), humidity (55%), and 12 h light/dark cycle (lights on at 7 a.m.) was maintained. The Governmental Animal Care and Use Committee LANUK North Rhine‐Westphalia authorized the animal protocol. All experiments were executed in agreement with the German (Institute for Laboratory Animal Research (ILAR) 2011) and European (2010/63/EU Directive on the protection of animals used for scientific purposes 2010) legislation on animal studies.

### 2.2 Study Design

**FIGURE 1:**
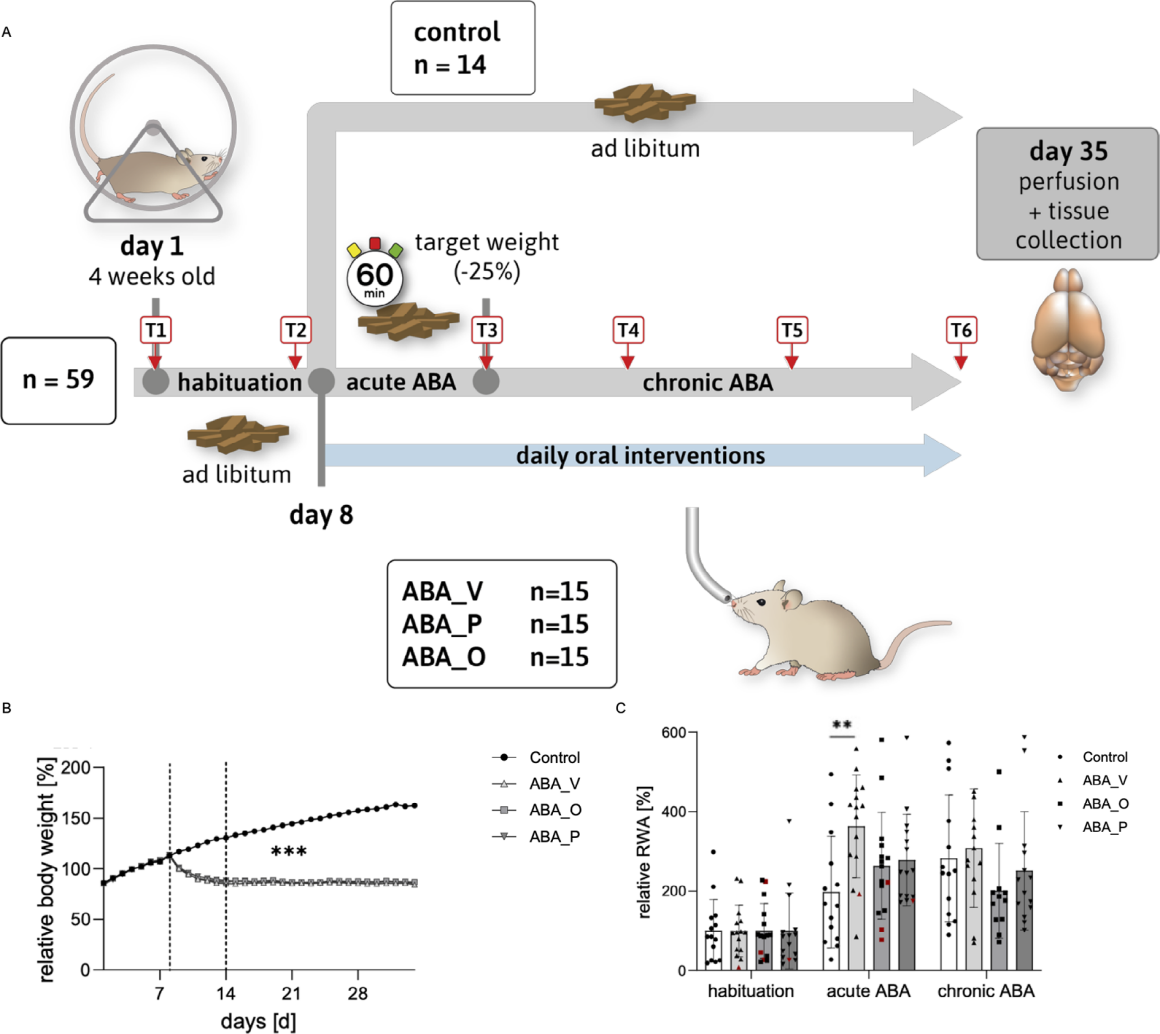
(A) Study design. An overview of the chronic ABA model with 4‐week‐old rats. ABA_O: starved animals with omega‐3 interventions, ABA_P: starved animals with probiotic interventions, ABA_V: starved animals with water interventions, C: Control. (B) Mean body weight per day normalized to body weight at habituation. (C) Mean running wheel activity during the different phases normalized to RWA at habituation. *p ≤ 0.05, ** *p* ≤ 0.01, ***p ≤ 0.001. Repeated measure ANOVA with post hoc Bonferroni test. Adapted from Verspohl et al. (2025), *Scientific Reports*, licensed under CC BY 4.0 (http://creativecommons.org/licenses/by/4.0/) (Verspohl et al. 2025). Timepoints of stool collection added to 1A.

The chronic ABA‐model was performed as described previously (Frintrop, Trinh, et al. 2018). After 1 week of acclimatization, rats were randomized into four groups: control (C, *n* = 14), vehicle (ABA_V, *n* = 15), omega‐A3 (ABA_O, *n* = 15), and probiotics (ABA_P, *n* = 15). Starting at habituation (days 1–7), food intake, running wheel activity (RWA), body weight, and stress levels were recorded daily with *ad libitum* food access. From day seven, interventions were administered daily via oral gavage: ABA_V received 1 mL water, ABA_O received omega‐3 (Opti3 Vegetology, Nottingham, UK) volume in mL equal to 1% of body weight, ABA_P received 40 mg OMNi‐BiOTiC SR‐9 (Graz, AT) in 1 mL water. During acute starvation (starting day 8), animals from the intervention groups had 1 h of daily feeding (1–2 p.m.) until a 25% weight loss target was reached. Since animals reached this target at different times, acute ABA was discontinued individually, and food intake was adjusted to maintain the target weight during chronic starvation. Rats that did not reach the target weight remained in acute ABA until day 35. Additional omega‐3 calories were compensated in the other groups. On day 35, rats were finalized. Stool samples were obtained from all rats at six time points: T1 (arrival/day 1), T2 (end of habituation/day 7), T3 (target weight/day 9–14), T4 (day 21), T5 (day 28), T6 (finalization/day 35). Figure 1A displays the study design.

### 2.3 Sample Collection and Preparation

At the conclusion of the experiments, rats were euthanized with 100% isoflurane (Piramal, Mumbai, India) and transcardially perfused with 150 mL phosphate‐buffered saline (Thermo Fisher Science, Waltham, USA). The brain was extracted and split into two hemispheres at the midsagittal line. Tissue samples of the hippocampus were taken from the left hemisphere, while the right hemisphere was post‐fixed with a 3.7% paraformaldehyde solution (pH 7.4), dehydrated in an increasing alcohol/xylol series, and embedded in paraffin (Merck, Darmstadt, Germany). With a microtome (RM2255, Leica, Wetzlar, Germany), 5 μm‐slices were cut frontally at Bregma −2.30.

### 2.4 Immunohistochemistry

The detection of specific proteins from two slices per animal was performed following a standard procedure as previously stated (Verspohl et al. 2025). The region of interest was in the medial hilus region of the right dentate gyrus of the hippocampal formation, as it offers a clear anatomical landmark across samples and is one of the primary sites of adult neurogenesis (Figure S1). Table S1 displays the applied antibodies. An Eclipse 80i microscope (Nikon, Tokyo, Japan) was used to digitize the stained tissue under consistent illumination. Two independent observers counted visible nuclei blindly with the ImageJ software (version 1.52a, NIH, Bethesda, USA). Results are presented as cells/mm^2^.

### 2.5 Real‐Time Polymerase Chain Reaction

RNA was extracted from the tissue of the whole hippocampal formation using phenol‐chloroform as described previously (Verspohl et al. 2025). Following RNA isolation, reverse transcription for small RNAs (Voelz et al. 2021) and other types of RNA was performed (Verspohl et al. 2025). The ratio between a target gene and stable reference genes (*miR‐103‐3p* and *miR‐107‐3p* for miRNA; *cyclophilin A* for cDNA) was determined to compare the relative RNA expression. Table S2 displays the used primers. Changes in the expression of target genes were displayed by the fold change relative to controls.

### 2.6 MRI‐Based Hippocampus Volume Measurement

A 7 Tesla MRI scanner (BioSpec 70/20 USR, Bruker, Billerica, USA) was used to perform magnetic resonance imaging of all animals after habituation (T2) and before finalization (T6). The applied volume coil RF Res 300 1H 112/086 QSN TO AD with a coverage of 42 mm in z‐direction and a surface coil T115533 (Bruker, Billerica, USA) is regularly used for rat imaging. Rats were anesthetized with 5% isoflurane (Piramal) in oxygen‐enriched air throughout the entire scan, maintaining a respiratory rate of 80 breaths/min. In T2‐weighted images (T2‐TurboRARE, field of view: 32 × 25 mm, slice thickness: 0.5 mm, pixel size: 128 × 100 μm, TR: 7234 ms, TE: 46 ms), the hippocampus was manually and blindly segmented by one observer. The software ITK‐Snap (version 4.0.1) was employed for segmentation (Figure S3A) (Yushkevich et al. 2006).

### 2.7 Analysis of the Microbiome of the Fecal Samples

The V3‐V4 region of the 16S rRNA gene was amplified using 515F–806R primers, forward: GTGCCAGCMGCCGCGGTAA and reverse: GGACTACHVGGGTWTCTAAT, with a dual barcoding approach (Caporaso et al. 2011). The resulting library was sequenced on an Illumina Miseq sequencer (300PE). During demultiplexing, only barcodes without mismatches were allowed (Casava, Illumina). Raw sequencing data were processed using the DADA2 pipeline, implemented in the *dada2* R package, version 1.16.0 (Callahan et al. 2016). The first 20 bases were trimmed from the 5′ end of forward or reverse sequences, truncating the 290 bases from the 3′ end of forward or reverse sequences. Reads were also truncated at the first instance of a quality score ≤ 3 (Table S3). An abundance table of amplicon sequence variants (ASVs) was generated, and their taxonomic annotation was obtained using the SILVA 138 database (Quast et al. 2013) (Table S4). The samples were randomly sub‐sampled to 10,000 sequences.

### 2.8 Statistical Analysis

All data were analyzed using SPSS (version 29, IBM, Chicago, USA) and visualized with GraphPad Prism (9.4.1, Boston, USA). Repeated‐measure ANOVA (bodyweight, RWA) or one‐way ANOVA (IHC, qPCR, MRI) with Bonferroni post hoc tests were executed for normally distributed data. Non‐normally distributed data were transformed or analyzed using Kruskal‐Wallis tests when transformation failed. Outliers were identified using Grubbs' test (Grubbs 1969). Results are presented as means with standard deviations. Bodyweight and RWA are normalized to the individual values of each animal during the habituation phase and reported as daily means for each group. Significance level was set to 0.05, and trend level was set to 0.1.

Microbiome data were analyzed with the *phyloseq* and *vegan* packages in R (version 4.4.1) to estimate α‐diversity (Shannon and Chao1 indexes) and β‐diversity (Bray‐Curtis distances) (McMurdie and Holmes 2013). Statistical tests of the α‐diversity were assessed with a linear model in *lme4* and *lmerTest* packages (Bates et al. 2014; Kuznetsova et al. 2015). Fixed effects were evaluated using ANOVA within the mixed model, with T1 and T2 as baseline covariates. A random intercept for individual rats was included to account for repeated measures. β‐diversity differences were assessed with a permutational analysis of variance (PERMANOVA; 10,000 permutations) test on Bray‐Curtis distances using the *adonis* function. *p*‐values were corrected using the Benjamini‐Hochberg correction. Differentially abundant core taxa at species and genus levels in samples from T3 were calculated in *Maaslin2* (Mallick et al. 2021). Core microbiome was measured in the *microbiome* package (Lahti and Sudarshan 2017), and it was identified as ASVs with a relative abundance of 1% and a prevalence of 25%. Graphical representation was performed with *ggplot2* in R (Wickham 2016). Raw sequences are uploaded under the project number PRJNA1234054.

### 2.9 Mediation Analysis

To investigate potential mediation effects of the microbiome on the hippocampus, we conducted a mediation analysis using taxonomic abundance data and qPCR‐derived expression levels (*Aif1, Tnf, Il6*, and *Bdnf*), number of MAP2‐ and IBA1‐positive cells, as well as mean body weights of the experimental phases, and days until target weight.

Core taxa were identified at T6 in the *microbiome* package (detection threshold of 0.1% relative abundance and a prevalence of at least 25% across samples) at ASV, genus, and family levels.

A preliminary association analysis was performed using MaAsLin2 to identify bacterial taxa significantly correlated with body weight or a brain variable. These significant taxa were then selected as candidate mediators for further mediation modeling.

We employed structural equation modeling (SEM) using *lavaan* to test whether microbial taxa mediated the relationship between experimental groups and the other variables. The model was structured with:

Path a: The effect of the experimental group on microbial taxa.

Path b: The effect of microbial taxa on experimental variables.

Path c: The direct effect of the experimental group on experimental variables.

Taxa identified as significant mediators were further analyzed via scatter plots to visualize their association with experimental variables.

## 3 Results

### 3.1 Bodyweight and Activity

For detailed results on the experimental model, body weight, food intake, and running wheel activity, please refer to Verspohl et al. (2025). Briefly, since the second day of the acute starvation, the normalized body weight and food intake of ABA animals were significantly lower compared to controls (*p* ≤ 0.001; Figure 1B). Mean days until target weight was reached did not differ significantly between ABA groups (*p* = 0.45). Five animals did not reach the target weight and remained in acute ABA until the conclusion of the experiment (ABA_V: *n* = 1; ABA_O: *n* = 3; ABA_P: *n* = 1). ABA_V showed significantly increased running wheel activity during acute starvation compared to controls (*p* = 0.0173), which appeared attenuated in the intervention groups (Figure 1C).

### 3.2 Microglia and Inflammation

**FIGURE 2:**
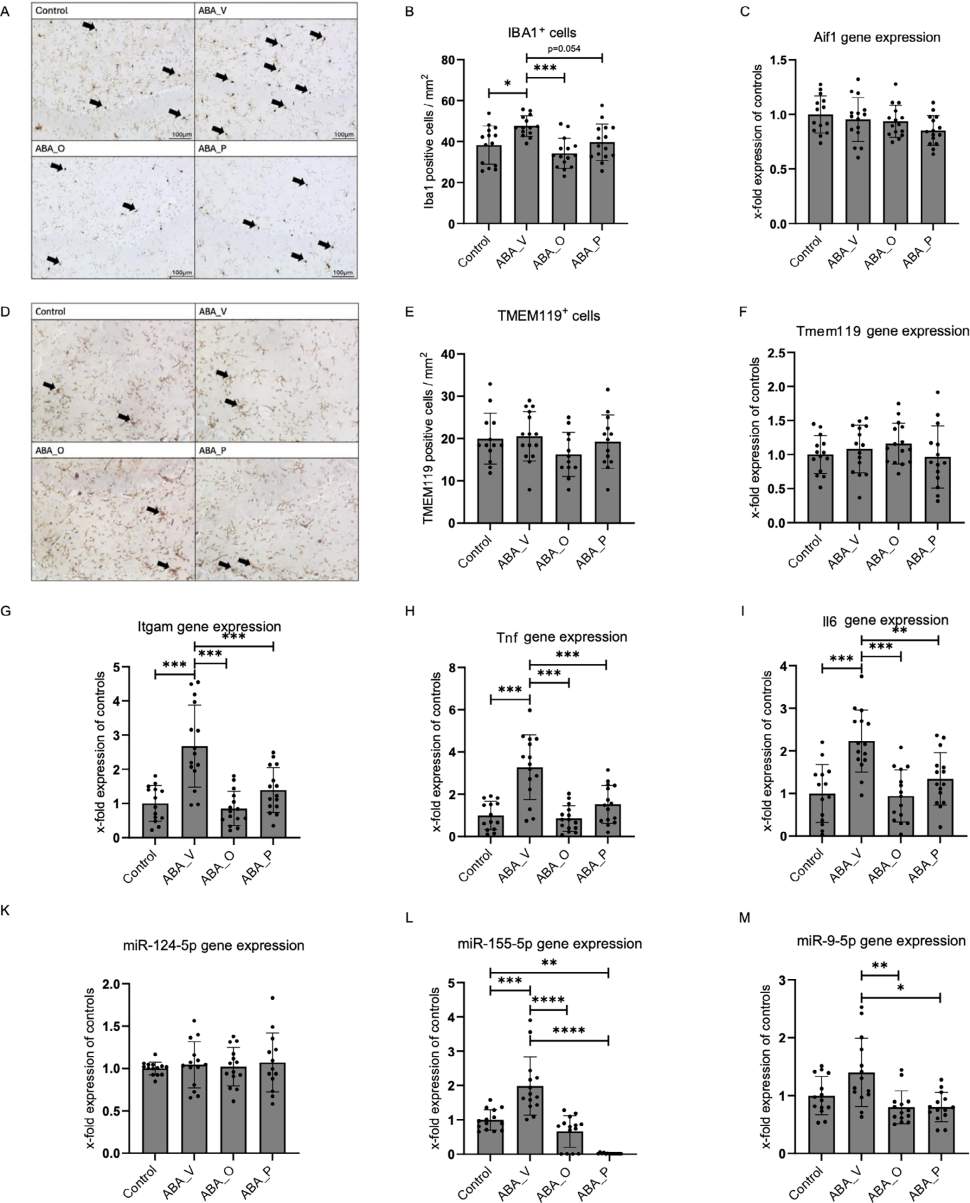
(A) Immunohistological staining for IBA1 from control, ABA_V, ABA_O, and ABA_P in the hippocampus. Microglia are marked exemplarily with arrows. (B) IBA1 positive cellular density/mm^2^. (C) Aif1 mRNA expression in relation to controls. (D) Immunohistological staining for TMEM119 from control, ABA_V, ABA_O, and ABA_P in the hippocampus. Microglia are marked exemplarily with arrows. (E) TMEM119 positive cellular density/mm^2^. (F) *Tmem119* mRNA expression in relation to controls. (G) *Itgam* mRNA expression in relation to controls. (H) *Tnf* mRNA expression in relation to controls. (I) *Il6* mRNA expression in relation to controls. (K) *miR‐124‐5p* expression in relation to controls. (L) *miR‐155‐5p* expression in relation to controls. (M) *miR‐9‐5p* expression in relation to controls. *p ≤ 0.05, **p ≤ 0.01, ***p ≤ 0.001. One‐way ANOVA with post hoc Bonferroni test. Control group with food ad libitum and no interventions, ABA_O: starved animals with omega‐3 interventions, ABA_P: starved animals with probiotic interventions, ABA_V: starved animals with water interventions.

There was a significant increase in the number of IBA1‐positive cells in the ABA_V group compared to control (*p* = 0.017), which was reduced again in ABA_O (*p* = 0.0002), and trend‐level reduced in ABA_P (*p* = 0.054) (Figure 2A,B).

Gene expression of *Aif1* (gene encoding IBA1) and *Tmem119*, along with the cellular density of *TMEM119*, was not significantly different (Figure 2C–F). *Itgam* mRNA expression (gene encoding CD11b) differed significantly, with elevated values in the ABA_V group compared to all other groups (all *p* ≤ 0.001; Figure 2G). Results for *Tnf* gene expression (all *p* ≤ 0.001; Figure 2H) and *Il6* gene expression (all *p* ≤ 0.01; Figure 2I) showed a similar increase in ABA_V and were reduced again in ABA_O and ABA_P.

Expression of *miR‐124‐5p* displayed no significant variations, while expression of *miR‐155‐5p* was again significantly higher in the ABA_V group relative to the other groups and *miR‐9‐5p* relative to ABA_O and ABA_P (all *p* < 0.01; Figure 2K–M).

### 3.3 Proliferation and Apoptosis

**FIGURE 3:**
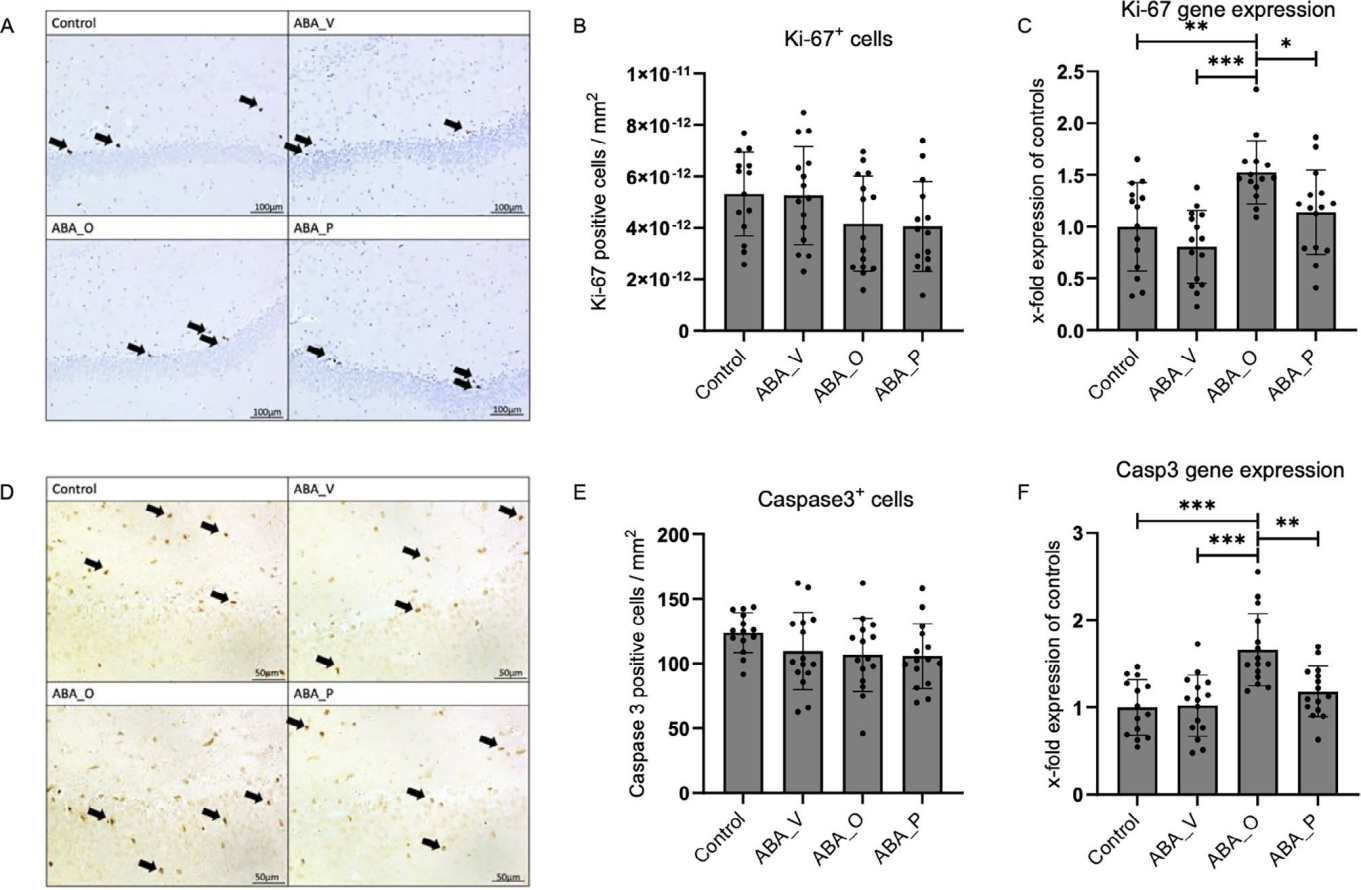
(A) Immunohistological staining for Ki‐67 from control, ABA_V, ABA_O, and ABA_P in the hippocampus. Proliferation is marked exemplarily with arrows. (B) Ki‐67 positive cellular densitiy/mm^2^. (C) *Ki‐67* mRNA expression in relation to controls. (D) Immunohistological staining for Caspase 3 from control, ABA_V, ABA_O, and ABA_P in the hippocampus. Apoptosis is marked exemplarily with arrows. (E) Caspase 3 positive cellular densitiy/mm^2^ (F) Caspase *3* mRNA expression in relation to controls. *p ≤ 0.05, **p ≤ 0.01, ***p ≤ 0.001. One‐way ANOVA with post hoc Bonferroni test. Control group with food ad libitum and no interventions, ABA_O: starved animals with omega‐3 interventions, ABA_P: starved animals with probiotic interventions, ABA_V: starved animals with water interventions.

Immunohistochemistry for proliferation and apoptosis markers revealed no significant alterations; however, it showed nominally reduced cellular densities in the ABA groups (ANOVA Ki‐67: *p* = 0.11, Figure 3A,B; ANOVA Caspase 3: *p* = 0.208, Figure 3D,E). Gene expression of *Ki‐67* and *Caspase 3* was significantly altered (Figure 3C,F): While no significant difference was found between controls and ABA_V, *Ki‐67* and *Casp3* expressions were significantly elevated in ABA_O compared to all other groups (all *p* < 0.01).

### 3.4 Neurons

**FIGURE 4:**
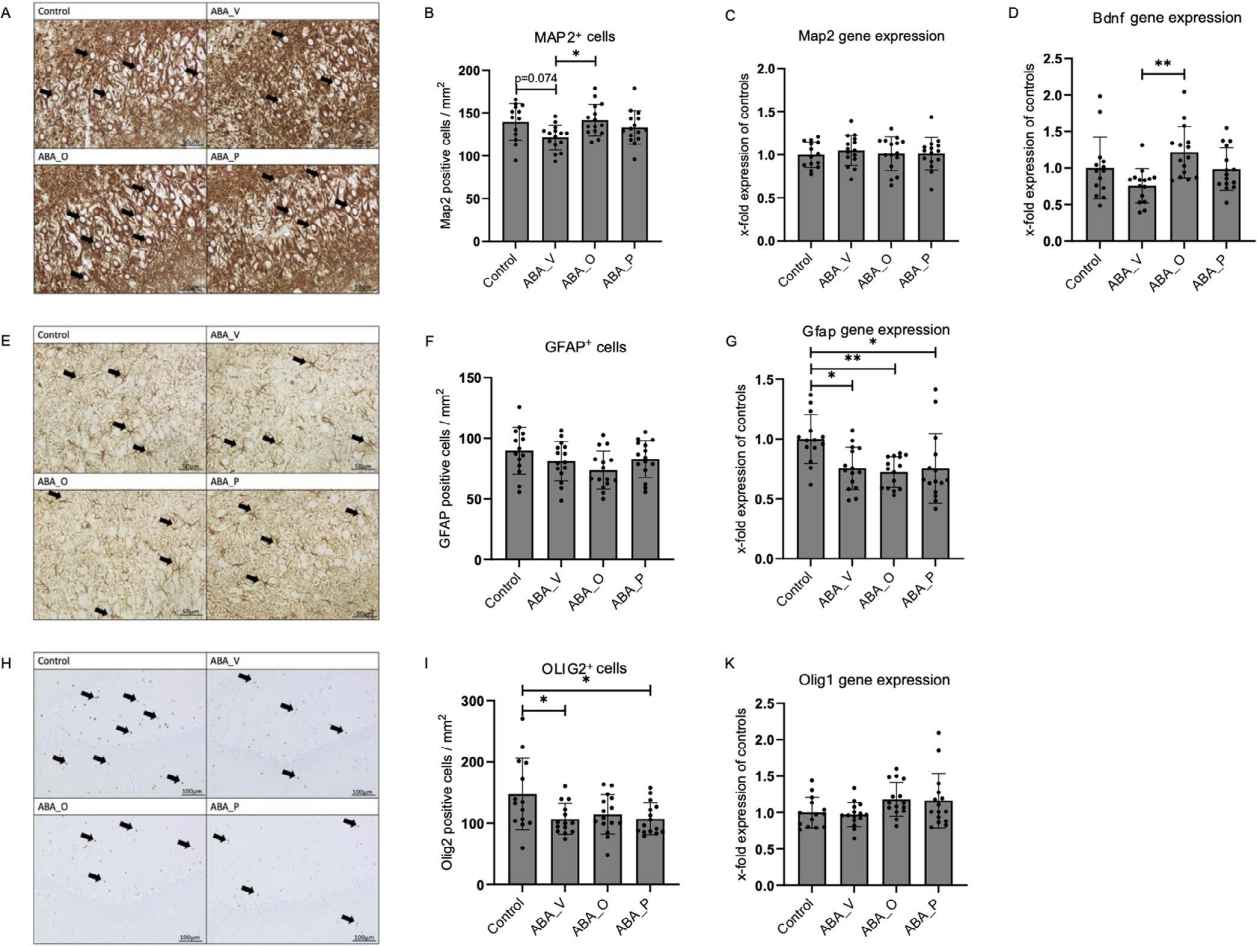
(A) Immunohistological staining for MAP2 from control, ABA_V, ABA_O, and ABA_P in the hippocampus. Neurons are marked exemplarily with arrows. (B) MAP2 positive cellular densitiy/mm^2^. (C) *Map2* mRNA expression in relation to controls. (D) *Bdnf* mRNA expression in relation to controls. (E) Immunohistological staining for GFAP‐positive astrocytes from control, ABA_V, ABA_O, and ABA_P in the hippocampus. Astrocytes are marked exemplarily with arrows. (F) GFAP positive astrocyte cellular densitiy/mm^2^. (G) *Gfap* mRNA expression in relation to control. (H) Immunohistological staining for OLIG2 from control, ABA_V, ABA_O, and ABA_P in the hippocampus. Oligodendrocytes are marked exemplarily with arrows. (I) OLIG2 positive cellular densitiy/mm^2^. (K) *Olig1* mRNA expression in relation to controls. *p ≤ 0.05, **p ≤ 0.01, ***p ≤ 0.001. One‐way ANOVA with post hoc Bonferroni test. Control group with food ad libitum and no interventions, ABA_O: starved animals with omega‐3 interventions, ABA_P: starved animals with probiotic interventions, ABA_V: starved animals with water interventions.

The cellular density of MAP2‐positive cells (Figure 4A,B) indicated a trend toward reduced density in ABA_V vs. controls (*p* = 0.074). Following the omega‐3 intervention, a significant increase in ABA_O animals was noted compared to ABA_V (*p* = 0.026). No significant differences were observed in *Map2* gene expression (ANOVA: *p* = 0.875; Figure 4C). Gene expression of *Bdnf* (Figure 4D) revealed significantly increased levels in ABA_O compared to ABA_V (*p* = 0.002), while *TrkB* expression did not differ significantly between groups (Figure S2).

### 3.5 Astrocytes and Oligodendrocytes

Numbers of GFAP‐positive astrocytes were trend‐level reduced in all ABA groups compared to the control group (ANOVA: *p* = 0.09; Figure 4E,F). However, a significant reduction of *Gfap* mRNA expression in ABA animals (Figure 4G) was observed compared to controls (ABA_V: *p* = 0.015, ABA_O: *p* = 0.005, ABA_P: *p* = 0.015).

Immunohistochemistry of OLIG2 displayed significantly lower cellular densities in the ABA_V and ABA_P groups but not in ABA_O (ABA_V: *p* = 0.037, ABA_O: *p* = 0.13, ABA_P: *p* = 0.034; Figure 4H,I), while divergences in the qPCR showed no differences (ANOVA: *p* = 0.058; Figure 4K).

### 3.6 Hippocampal Volume

The total hippocampus volume difference between the two measurements before and after starvation displayed no significant changes (ANOVA *p* = 0.213; Figure S3B–D).

### 3.7 Microbiome Composition

Fecal samples from six different timepoints were analyzed to highlight possible associations between brain changes and the gut microbiome. Sequencing of 343 samples, including the probiotic mix used as an intervention, generated a total of 19,679,496 sequences (56,877.16 ± 15,095.01; Table S4).

**FIGURE 5:**
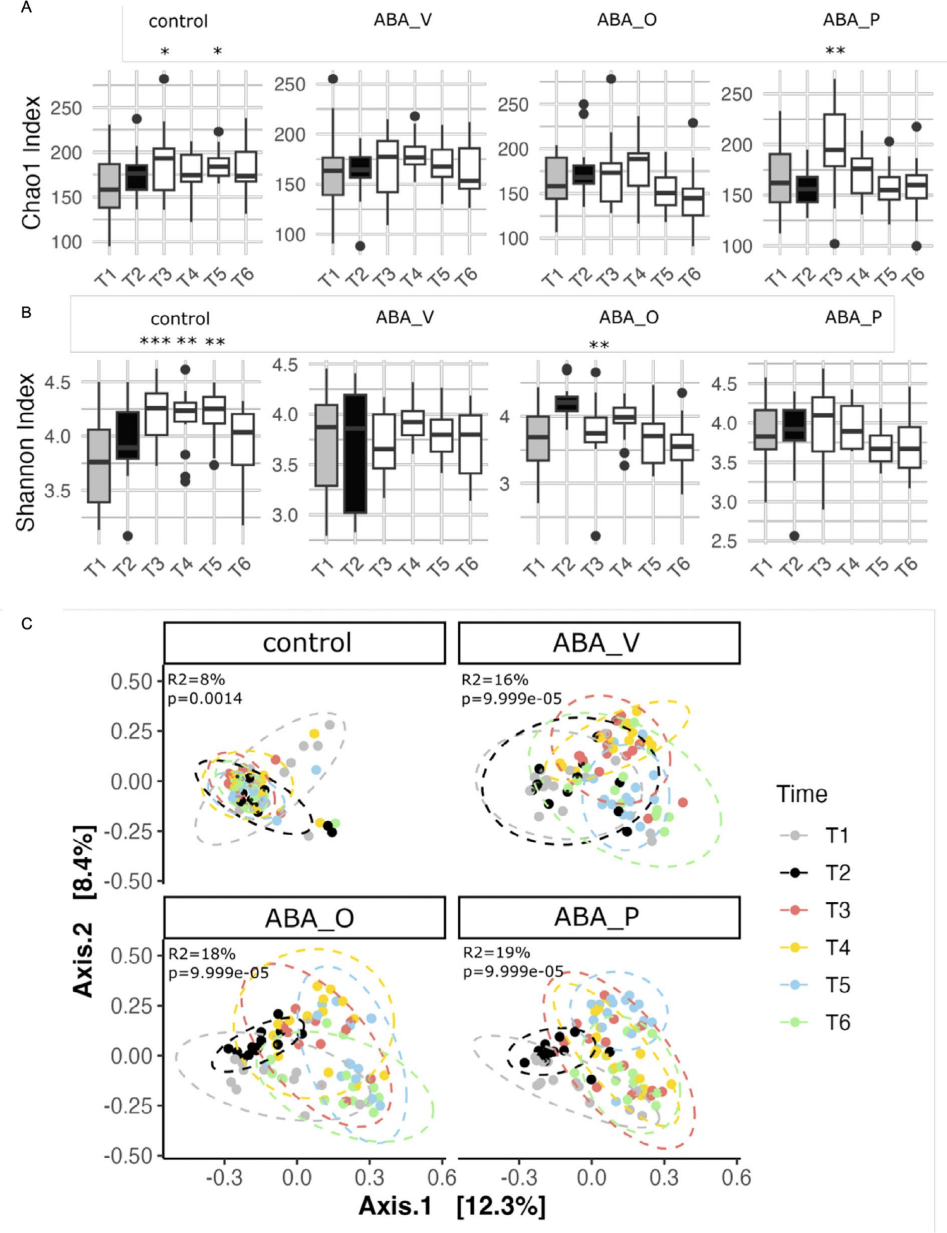
(A) Chao1 Index and (B) Shannon Index measure α‐diversity in the control and treatment groups (ABA_V, ABA_O, ABA_P) over time. Significant differences of timepoints to baseline measures (T1 and T2) are marked (linear model; *p < 0.05, **p < 0.01, ***p < 0.001). (C) Principal Coordinate Analysis (PCoA) based on Bray–Curtis dissimilarity showing β‐diversity of the microbial communities in the control and treatment groups. Each time point (T1–T6) is represented by a different color, and dashed ellipses indicate the 95% confidence intervals for each time point. The *R* ^2^ and *p*‐values indicate the proportion of variance explained and statistical significance for each group. Control group with food ad libitum and no interventions, ABA_O: starved animals with omega‐3 interventions, ABA_P: starved animals with probiotic interventions, ABA_V: starved animals with water interventions.

Analysis of the Chao1 index revealed no statistical difference at the first two sampling points and no batch effects. Group (ANOVA of linear model, *p*_GROUP_ = 0.0497), ABA (ANOVA of linear model, *p*_ABA_ = 0.02108), and timepoint (*p*_TIME_ ≤ 0.0005) significantly affected species richness. Specifically, acute starvation shows significantly higher species richness (linear model; *p*_T3–T5_ = 0.000439, *p*_T3–T6_ ≤ 0.0005). Within‐group analysis revealed a significantly higher species richness at T3 and T4 (*p*_T3_ = 0.0251 and *p*_T4_ = 0.0423) compared to T1 in the control group and significantly higher species richness at T3 (*p*_T3_ = 0.0053) compared to T1 in ABA_P (Figure 5A).

To calculate the Shannon index, we recorded no statistical difference at the first two sampling points and no batch effects. Group (ANOVA of linear model, *p*_GROUP_ ≤ 0.0005), ABA (*p*_ABA_ ≤ 0.0005), and timepoint (*p*_TIME_ ≤ 0.0005) significantly affected species evenness. Specifically, group and ABA explained significant variation (Table S5), and acute starvation again showed a significantly higher species evenness than the endpoint (linear model; *p*_T3–T6_ = 0.002097). Interestingly, the largest effect of time was recorded in the control group (Figure 5B), pointing to normal maturation effects in adolescent rats.

Analysis of β‐diversity based on the Bray–Curtis dissimilarity index non‐surprisingly revealed a greater overlap among fecal samples at T1 and T2 with fecal samples of rats belonging to the control group (Figure S4). This was confirmed by investigating the pairwise dissimilarity index across groups when considering samples between T3 and T6 (Figure S5). Control samples showed higher similarity to T2 samples and higher dissimilarity to the other groups, suggesting a greater effect of the ABA protocol over interventions. Overall, we recorded a batch effect, time effect, and group effect (all *p* ≤ 0.00005; Table S6). Figure 5C reports the PCoA plots based on the Bray–Curtis dissimilarity index divided by the 4 groups. Differentially abundant taxa in the fecal microbiome of rats were identified using Maaslin2 on fecal samples collected between T3 and T6 (Figure S6), confirming a good degree of similarity among interventions.

**FIGURE 6:**
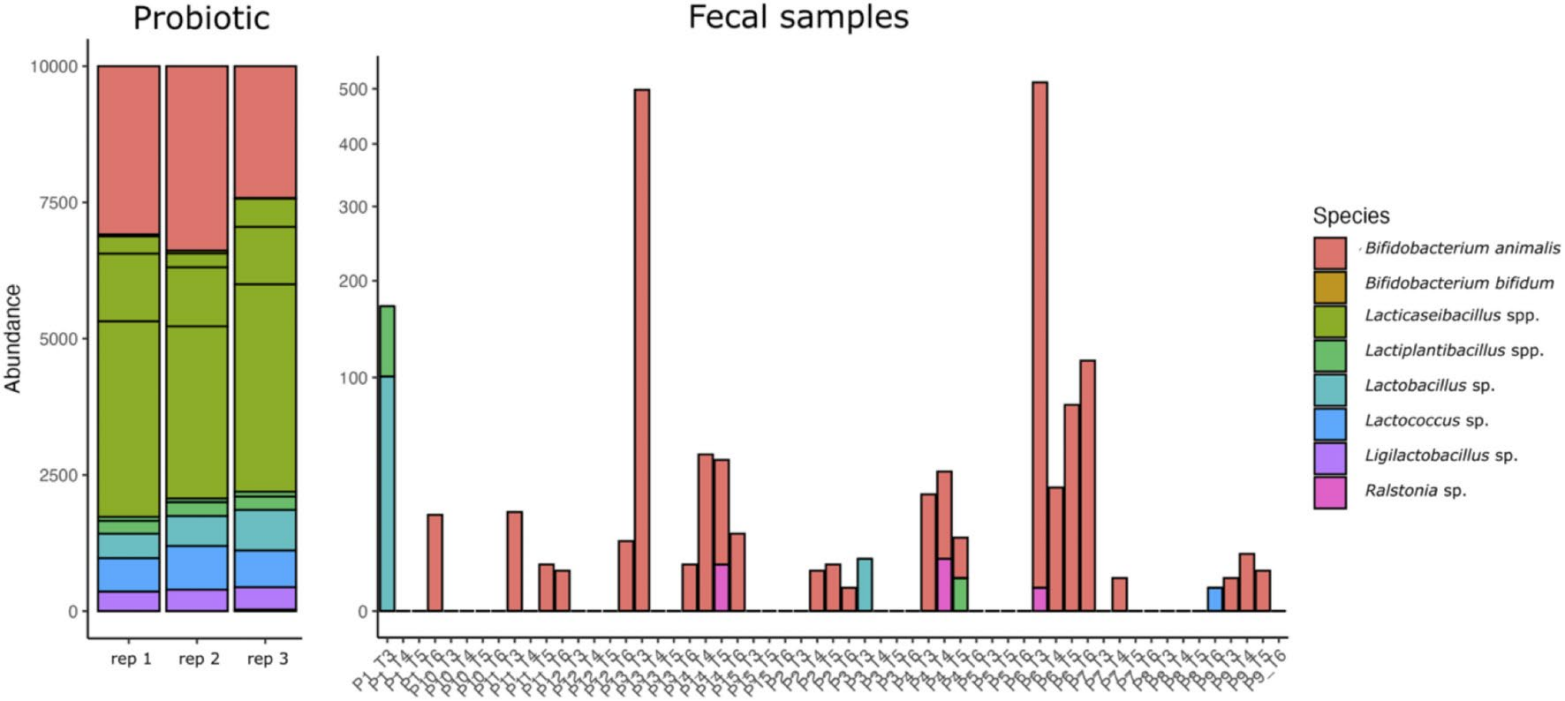
Microbial composition of the probiotic mixture and occurrence of probiotic SVs in the fecal microbiome of treated rats. Control group with food ad libitum and no interventions, ABA_O: starved animals with omega‐3 interventions, ABA_P: starved animals with probiotic interventions, ABA_V: starved animals with water interventions.

To test the persistence of microbial species, we sequenced the probiotic samples and quantified the presence of the same ASV in the fecal samples of treated rats from T3 to T6. We observed at least one ASV ascribed to one of the components of the probiotic mix in 28 out of 57 fecal samples analyzed. Overall, *Bifidobacterium animalis* was the bacterial species with higher persistence in the microbiome, but with some inter‐individual variability (Figure 6).

### 3.8 Mediation Analysis

Various candidate taxa for each analysis are reported in Table S6. Mediation analysis identified several taxa potentially linking microbial shifts to host gene expression and physiological responses.

At the ASV level, significant associations were found (Figure S7). Fewer associations emerged at the genus level (Figure S8).

At the family level, significant associations were found between bodyweight (days 1–8) and Marinifilaceae (*p* = 0.008) and Butyricicoccaceae (*p* = 0.017); bodyweight (days 9–14) and Eggerthellaceae (*p* = 0.038); *Tnf* expression and Anaerovoracaceae (*p* = 0.05), and IBA1‐positive cell counts and Akkermansiaceae (*p* = 0.017; Figure S9).

## 4 Discussion

Our findings demonstrate that chronic ABA induces a neuroinflammatory response in the hippocampus of adolescent female rats, including increased inflammatory markers and microglia. This effect was reversed by omega‐3 and probiotic interventions. Moreover, omega‐3 administration increased neuronal cellular density, Bdnf expression, and cell neogenesis. Chronic ABA furthermore resulted in glia cell reduction in the hippocampus, which remained unaltered by the interventions. The microbiome composition was primarily influenced by the ABA model, with minor effects from omega‐3 and probiotic intervention. Thus, omega‐3 and probiotics supplementation selectively ameliorated neuroinflammation, including microglia, induced modest effects on the microbiome, but did not prevent astrocyte or oligodendrocyte loss in the hippocampus.

### 4.1 Increased Microglia and Inflammation

Chronic ABA induced elevated hippocampal IBA1‐positive microglial cells and inflammation markers, consistent with prior results in an acute ABA model (Ragu‐Varman et al. 2019; Reyes‐Ortega et al. 2020). In contrast, microglial reduction was observed in the cerebral cortex and the corpus callosum of chronically starved rats, suggesting a region‐specific response, possibly due to the hippocampus's higher metabolic demand and baseline microglial density (Verspohl et al. 2025; Zimmermann et al. 2023). However, an increase in microglia and pro‐inflammatory cytokines aligns with findings of altered inflammatory processes in patients (elevated TNF‐α and IL‐6) (Dalton et al. 2018; Solmi et al. 2015), though specific results on inflammatory alterations are heterogeneous.

Hippocampal neuroinflammation may arise from two pathways. Firstly, starvation‐induced upregulation of miR‐155‐5p and miR‐9‐5p might promote inflammation by inducing cytokine release and microglial activation, and impairing the blood–brain barrier (Lopez‐Ramirez et al. 2014; Ponomarev et al. 2013; Xian et al. 2022). These two miRs could represent initial regulators in a complex pathophysiological network driving hippocampal neuroinflammation in AN (Käver et al. 2024; Korten et al. 2025).

Secondly, immigrating immune cells may contribute to the observed neuroinflammation. Different triggers, such as diseases, can induce microglial activation (Zusso et al. 2012). This activation encompasses an intricate process with modified cellular density, cell morphology, antigen presentation, gene expression, and cytokine release (Wittekindt et al. 2022). Although Aif1/IBA1 is commonly used as a microglia marker, it also labels activated macrophages, whereas TMEM119 is specific to resident microglia (Bennett et al. 2016).

Since the *Aif1* and *Tmem119* gene expressions were not elevated in this study, the observed increase in IBA1‐positive cells is unlikely to result from microglial activation. It might be explained by the infiltration of macrophages into the brain, followed by differentiation into microglia‐like cells during neuroinflammation (Bennett et al. 2016; O'Koren et al. 2016). We demonstrated amplified pro‐inflammatory cytokines in food‐restricted rats, which indicate neuroinflammation and are consistent with macrophage immigration. Furthermore, the mRNA expression of *Itgam* (marker for monocytes, macrophages, and other leukocytes) was elevated in the same animals. Collectively, these results support the possibility that immune cells immigrate into the starved brain.

### 4.2 Reversed Inflammation by Omega‐3 and Probiotics

IBA1 cellular density was lower in omega‐3‐treated animals, similar to those receiving probiotics. IBA1 regulates actin‐crosslinking during microglial activation (Sasaki et al. 2001). Omega‐3 suppresses inflammation by promoting inflammation‐resolving mediators, reflected in diminished gene expression of pro‐inflammatory cytokines, *Itgam, miR‐155‐5p*, and *miR‐9‐5p* in this study (Calder 2017). Thus, omega‐3 supplementation likely decreases microglial activation and immune cell immigration, resulting in fewer IBA1‐positive cells. Omega‐3 also increased MAP2‐positive neurons in the hippocampus, suggesting reduced inflammation benefits neuronal health. As omega‐3 is essential for neurogenesis, synaptic plasticity, and neurotransmission, a deficiency can impair cognitive functions and alter microbial composition (Bazinet and Layé 2014; Jayapala and Lim 2023; Robertson et al. 2017). Supplementation has shown first evidence in reducing inflammation (Kavyani et al. 2022) and improving weight restoration in patients with AN (Satogami et al. 2019; Shih 2017).

Probiotic administration had a similar, though less pronounced, effect in our trial. Via the microbiome‐gut‐brain axis, probiotics potentially modulate inflammation by influencing gut permeability, nutrient availability, and host inflammatory processes (Seitz, Trinh, et al. 2019). Previous studies associated probiotics with decreased pro‐inflammatory cytokines and improved psychiatric symptoms (Faghfouri et al. 2023; Pirbaglou et al. 2016). In this study, probiotics also reduced inflammatory cytokine expression in the hippocampus, indicating an anti‐inflammatory effect mediated by the microbiome.

### 4.3 Reduction of Neurons, Astrocytes, and Oligodendrocytes

Chronic starvation was associated with a trend‐level reduction in neuronal cellular densities in the hippocampus, contrasting with previous studies showing no changes in neuronal numbers in the cortex or corpus callosum (Frintrop, Liesbrock, et al. 2018; Frintrop et al. 2019; Verspohl et al. 2025). However, neuronal morphology seems to be affected with decreased branching in the dorsal and increased branching in the ventral hippocampus of ABA rodents (Chowdhury, Barbarich‐Marsteller, et al. 2014; Chowdhury, Ríos, et al. 2014). Reduced glial cell and neuron counts may reflect energy deficits in AN, potentially causing the (pseudo‐)atrophy in rodents and patients (Frintrop et al. 2019; Rosa‐Caldwell et al. 2023; Trinh, Käver, et al. 2023). Nonetheless, neurons might exhibit greater resilience, resulting in prioritized preservation and minimal reductions. Omega‐3 supplementation, and to a lesser extent probiotics, attenuated this trend‐level reduction, with significant improvements in hippocampal neuronal density and *Bdnf* gene expression. These novel findings within the ABA model align with omega‐3's documented neuroprotective role, fostering neurogenesis and brain metabolism in other animal models (Crupi et al. 2013; He et al. 2009; Sakamoto et al. 2007). The increased apoptosis rate in the omega‐3 group might reflect increased cellular turnover and enhanced regenerative capacity.

Reduced neuronal numbers, impaired synaptogenesis, limited astrocytic support, and nutritional deficits may contribute to cognitive impairments in patients with AN, potentially hindering psychotherapeutic progress and re‐learning (Bahnsen et al. 2024; Frintrop, Liesbrock, et al. 2018; Frintrop et al. 2019).

For the first time, we documented decreased oligodendrocyte cellular density in the hippocampus after chronic starvation (Frintrop, Liesbrock, et al. 2018), extending recent findings of Verspohl et al. (2025). Neuroimaging studies of patients with AN revealed white matter alterations (Griffiths et al. 2021; Miles et al. 2020). Thus, our findings might help to explain the underlying mechanism.

### 4.4 Hippocampus Volume

In the present study, hippocampal volume did not significantly change after starvation. Previous research reported reduced cerebral cortex and corpus callosum volumes in ABA rats and decreased brain volumes, including the hippocampus of patients with AN. Our results suggest greater resilience in rats (Frintrop et al. 2019; Keeler et al. 2020; Seitz et al. 2018). Two opposing processes could overlap: volume loss due to glia (and potentially neuronal) cell reduction may be counteracted by microglia/inflammation‐related swelling.

### 4.5 Microbiome Composition and Persistence of Probiotic Taxa

Our longitudinal microbiome profiling revealed significant effects of groups, ABA, and timepoint on α‐diversity and β‐diversity. Following acute starvation, species richness and evenness significantly increased in control and probiotic groups. The elevation in the control group likely reflects microbiome stabilization after habituation or normal maturation effects in adolescent animals. This appeared preserved in the probiotic group; in contrast, the ABA_V and ABA_O groups showed no such changes. The increase in community evenness in the control, but not ABA groups, suggests that ABA induces microbiome disruption, with potentially fewer dominating species.

Analysis of β‐diversity showed tighter clustering within ABA groups than with control, regardless of interventions. This finding underscores that, despite the expected effect of omega‐3 (reviewed in Fu et al. (2021)) and probiotic treatment (He et al. 2021), the effect of ABA itself is larger than that of the individual interventions.

Sequencing confirmed a stable composition of the administered probiotic mixture, with *Bifidobacterium animalis* showing the highest persistence in the host's gut after administration. While only a subset of animals showed detectable engraftment of probiotic strains, this indicates mostly transient effects of probiotics while being administered, but limited colonization. Despite low colonization, probiotic administration resulted in measurably reduced Il6 and Tnf levels, supporting its beneficial effects on ABA‐induced physiological changes, as demonstrated in our previous work (Trinh, Käver, et al. 2023).

### 4.6 Microbiota‐Host Mediation Findings

Mediation analysis identified several microbial taxa as potential intermediaries linking microbiota composition to host outcomes. Notably, *Akkermansia* was linked to IBA1‐positive cell counts, aligning with its known role in mucin degradation and gut barrier function and supporting its growing reputation as a possible therapeutic target in AN.

Although associations between microbial taxa and host variables were modest, visual trends suggested potential group‐specific patterns. Hence, microbial‐host interactions may depend on physiological or treatment context. However, given the exploratory nature and limited sample size, these findings should be interpreted cautiously. Nonetheless, our results suggest that subtle taxa‐specific microbial shifts may influence host outcomes despite the absence of broad compositional shifts.

### 4.7 Limitations

When analyzing the effects of probiotic supplements, the specific composition of the probiotic must be considered. Also, translating animal studies' findings to humans is appealing but potentially misleading. The results of ongoing clinical RCTs investigating omega‐3 and probiotics are needed to draw more definitive conclusions regarding patient care (Gröbner et al. 2022; Keller et al. 2022). Also, the absence of control groups receiving omega‐3 or probiotics without ABA limits our ability to distinguish treatment‐specific effects from the reversal of ABA‐induced alterations.

### 4.8 Conclusion

Our study revealed increased pro‐inflammatory cytokines after chronic starvation and increased microglial activation in the hippocampus. Omega‐3 and probiotics reversed the increased neuroinflammation and microglial activation, with a modest but detectable effect on the microbiome that could mediate this effect. Potential underlying mechanisms include immune modulation, miRNAs, and inflammation‐reducing properties of omega‐3 and probiotics, resulting in neuroprotection, increased cell neogenesis in the hippocampus, and, in the case of omega‐3, a significant increase in neuronal cellular density.

This research advances understanding of the pathophysiology and progression of AN, particularly concerning inflammatory processes in the hippocampus, with potential implications for re‐learning in psychotherapy. Moreover, it suggests that omega‐3 and probiotics may be beneficial additions to the treatment of AN.

## Author Contributions

**A. C. Thelen:** data curation, writing – original draft, visualization. **N. Andreani:** writing – review and editing, visualization, data curation, funding acquisition. **N. M. Korten:** writing – review and editing, data curation. **M. Neumann:** data curation, writing – review and editing. **V. Verspohl:** data curation, writing – review and editing, visualization. **M. van Egmond:** data curation, writing – review and editing. **L. Kneisel:** data curation, writing – review and editing. **C. Voelz:** conceptualization, data curation, writing – review and editing. **L. Blischke:** writing – review and editing, data curation. **J. F. Baines:** writing – review and editing, funding acquisition. **J. Baier:** data curation, writing – review and editing. **J. Schumacher:** data curation, writing – review and editing. **F. Kiessling:** data curation, writing – review and editing. **B. Herpertz‐Dahlmann:** writing – review and editing, funding acquisition. **C. Beyer:** conceptualization, writing – review and editing. **L. Keller:** writing – review and editing, data curation. **J. Seitz:** conceptualization, writing – review and editing, funding acquisition. **S. Trinh:** writing – review and editing, data curation, funding acquisition, conceptualization.

## Ethics Statement

All experimental procedures were conducted following institutional and national guidelines for the care and use of laboratory animals. The study protocol was approved under the number 81‐02.04.2021.A183.

## Conflicts of Interest

The authors declare no conflicts of interest.

## Supporting information


**Figure S1:** An image of the rat's right brain hemisphere to showcase where the hippocampus was studied. The hilus region of the dentate gyrus is marked with a black rectangle.

**Figure S2:** Gene expression of TrkB in the hippocampus in relation to controls. One‐way ANOVA with post hoc Bonferroni test.

**Figure S3:** (a) An example of MRI segmentation with ITK‐Snap (ABA_O, after habitation). The segmented hippocampus is displayed in red. (b) MRI volume difference of hippocampus between T2 and T6. (c) MRI volume of hippocampus at M1. (d) MRI volume of hippocampus at M2. *p ≤ 0.05, **p ≤ 0.01, ***p ≤ 0.001. One‐way ANOVA with post hoc Bonferroni test.

**Figure S4:** Principal Coordinate Analysis (PCoA) based on Bray–Curtis dissimilarity showing β‐diversity of the microbial communities in the control and treatment groups. Each time point (T1–T6) is represented by a different shape, each group is represented by a different color and dashed ellipses indicate the 95% confidence intervals for each time point. The *R* ^2^ and *p*‐values indicate the proportion of variance explained and statistical significance for each group.

**Figure S5:** Bar plot of mean Bray‐Curtis distances with standard deviation across experimental groups. The plot compares microbial community dissimilarity between different conditions (T2, K, V, O, P) using Bray‐Curtis distances. K, V, O, and P groups include fecal samples from T3 to T6 timepoints. Significant differences between groups are indicated (*p < 0.05, **p < 0.01, ***p < 0.001). Error bars represent standard deviations, highlighting the variation within each comparison.

**Figure S6:** Heatmap of significant ASVs and genera across experimental groups. The heatmap displays the coefficients (coef) of associations between bacterial genera and experimental groups (K, V, O, P), as identified by Maaslin2 analysis. ASVs and Genera are listed along the y‐axis, and groups are on the x‐axis. The color scale represents the direction and magnitude of the associations, with green indicating positive coefficients (taxa enriched in the control group) and red indicating negative coefficients (taxa enriched in the group reported on the x‐axis). Asterisks denote genera with statistically significant associations (*q*‐value < 0.05).

**Figure S7:** Associations between microbial ASVs and host variables across experimental groups. Each panel shows scatter plots of a microbial ASVs' relative abundance vs. specific host variables, with data points and linear regression lines color‐coded by the experimental group (see legend). Trend lines represent associations within each group.

**Figure S8:** Associations between microbial genera and host variables across experimental groups. Each panel shows scatter plots of the relative abundance of a microbial genus vs. specific host variables, with data points and linear regression lines color‐coded by the experimental group (see legend). Trend lines represent associations within each group.

**Figure S9:** Associations between microbial families and host variables across experimental groups. Each panel shows scatter plots of the relative abundance of a microbial family vs. specific host variables, with data points and linear regression lines color‐coded by the experimental group (see legend). Trend lines represent associations within each group.

**Table S1:** A list of all primary antibodies used for immunohistochemistry including target, antibody host, concentration, antigen‐retrieval, manufacturer, and catalog number.

**Table S2:** A list of all primers used for real‐time polymerase chain reaction including annealing temperature, direction, and nucleotide sequence (Thermo Fischer Science).

**Table S3:** Samples used for microbiome analysis.

**Table S4:** Abundance table of 16S rRNA amplicon sequence variants (ASVs) with taxonomic annotation of ASVs.

**Table S5:** Results of Chao1 and Shannon index.

**Table S6:** Results of mediation analysis.

## Data Availability

Raw data of microbiome sequencing have been submitted to SRA, but will be available upon publication. Reviewers can access raw data prior to the acceptance of the paper at the reviewer's link: https://dataview.ncbi.nlm.nih.gov/object/PRJNA1223159?reviewer=h1vr18otg7777g4e9k50mucpeu. Other data are available from the corresponding authors on reasonable request.
