# Supplementary figures and images for: The Influence of Omega‐3 Fatty Acids and Probiotics on Hippocampal Inflammation and Glial Cells in a Chronic Anorexia Nervosa Rat Model
Source: Int J Eat Disord. 2025 Oct 18;59(2):260–75. doi: 10.1002/eat.24574 (PMC12884241; doi:10.1002/eat.24574)

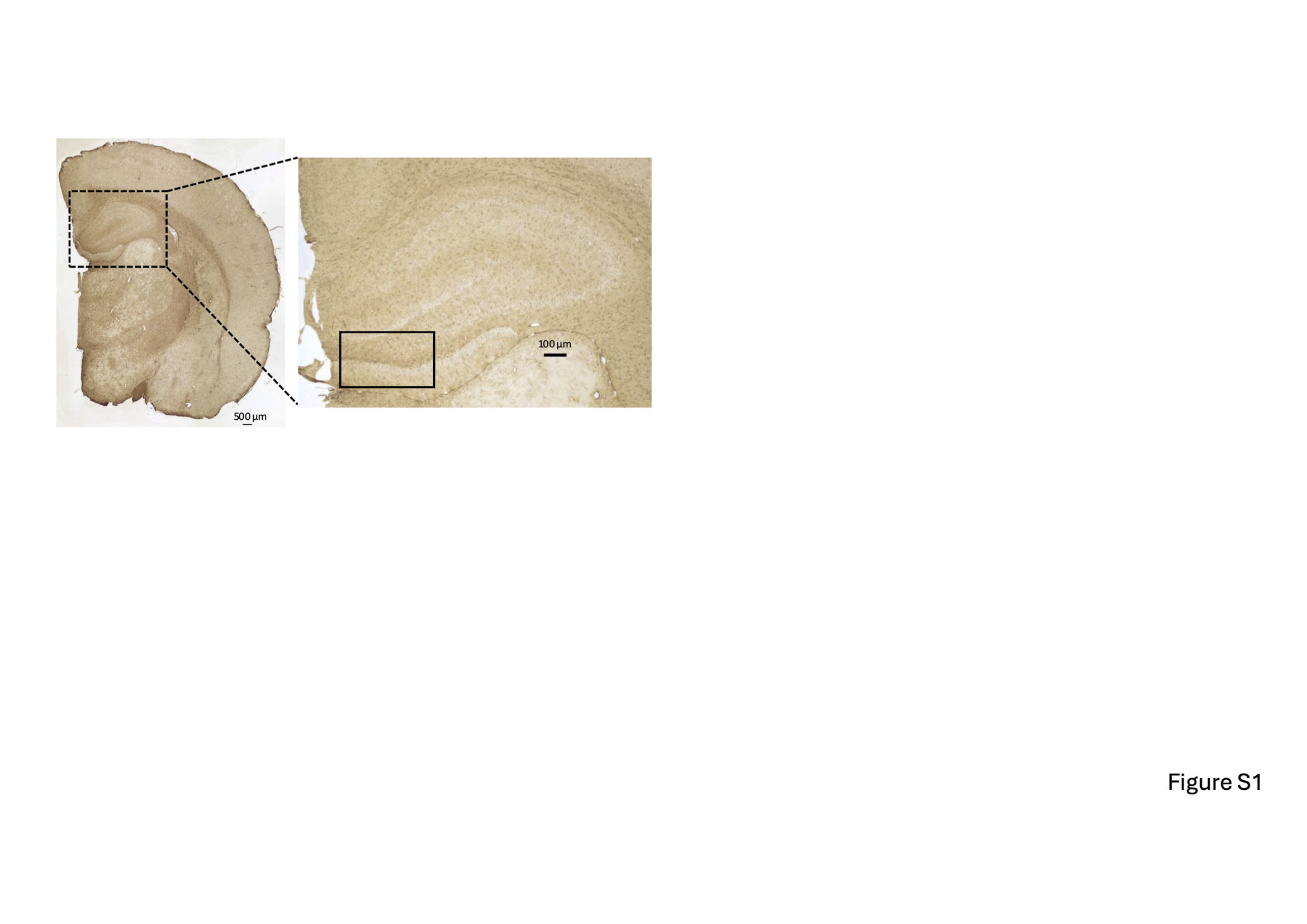

Supplement: Supplementary file 1 — Figure S1: An image of the rat's right brain hemisphere to showcase where the hippocampus was studied. The hilus region of the dentate gyrus is marked with a black rectangle. [file EAT-59-260-s002.tiff]

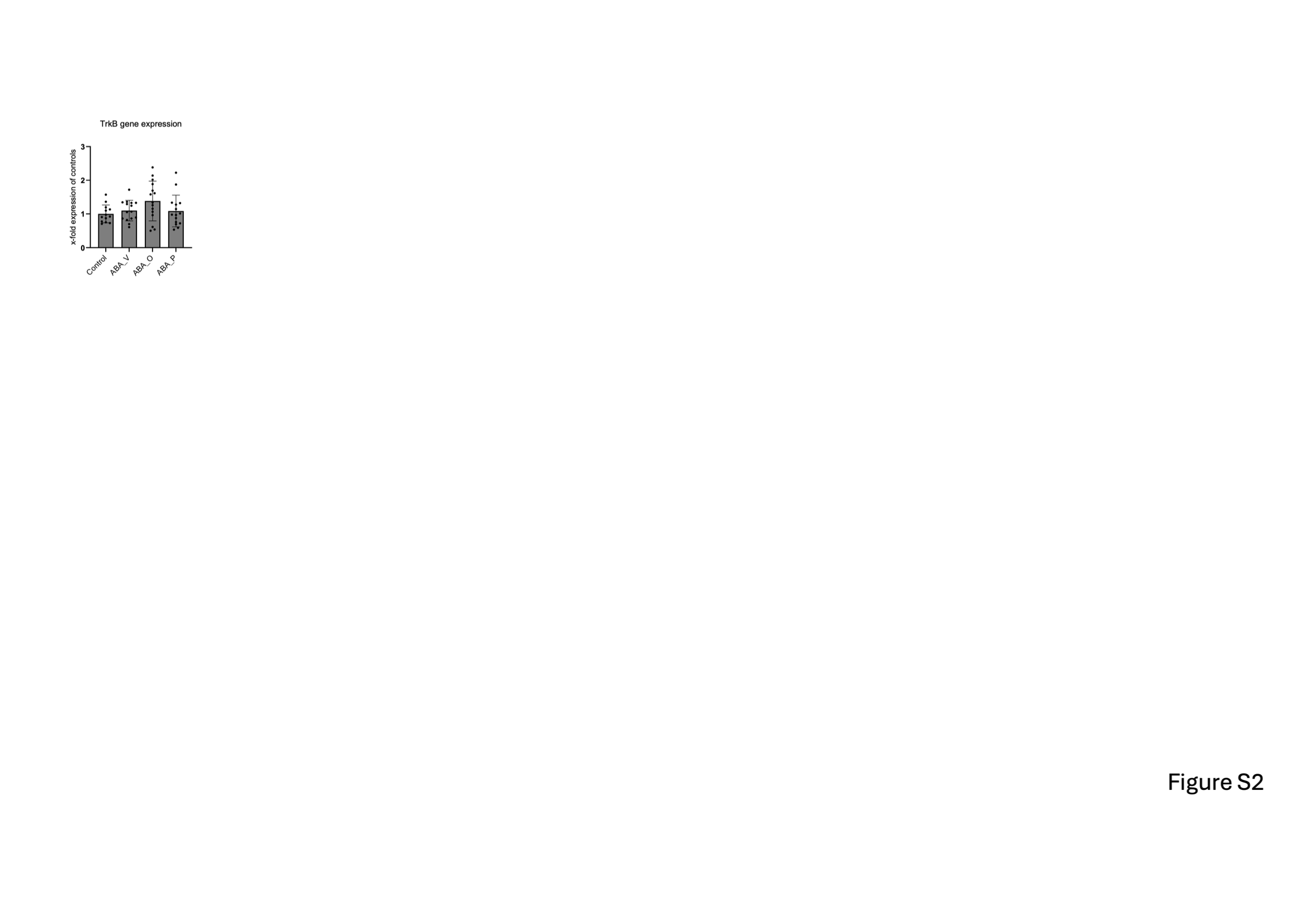

Supplement: Supplementary file 2 — Figure S2: Gene expression of TrkB in the hippocampus in relation to controls. One‐way ANOVA with post hoc Bonferroni test. [file EAT-59-260-s011.tiff]

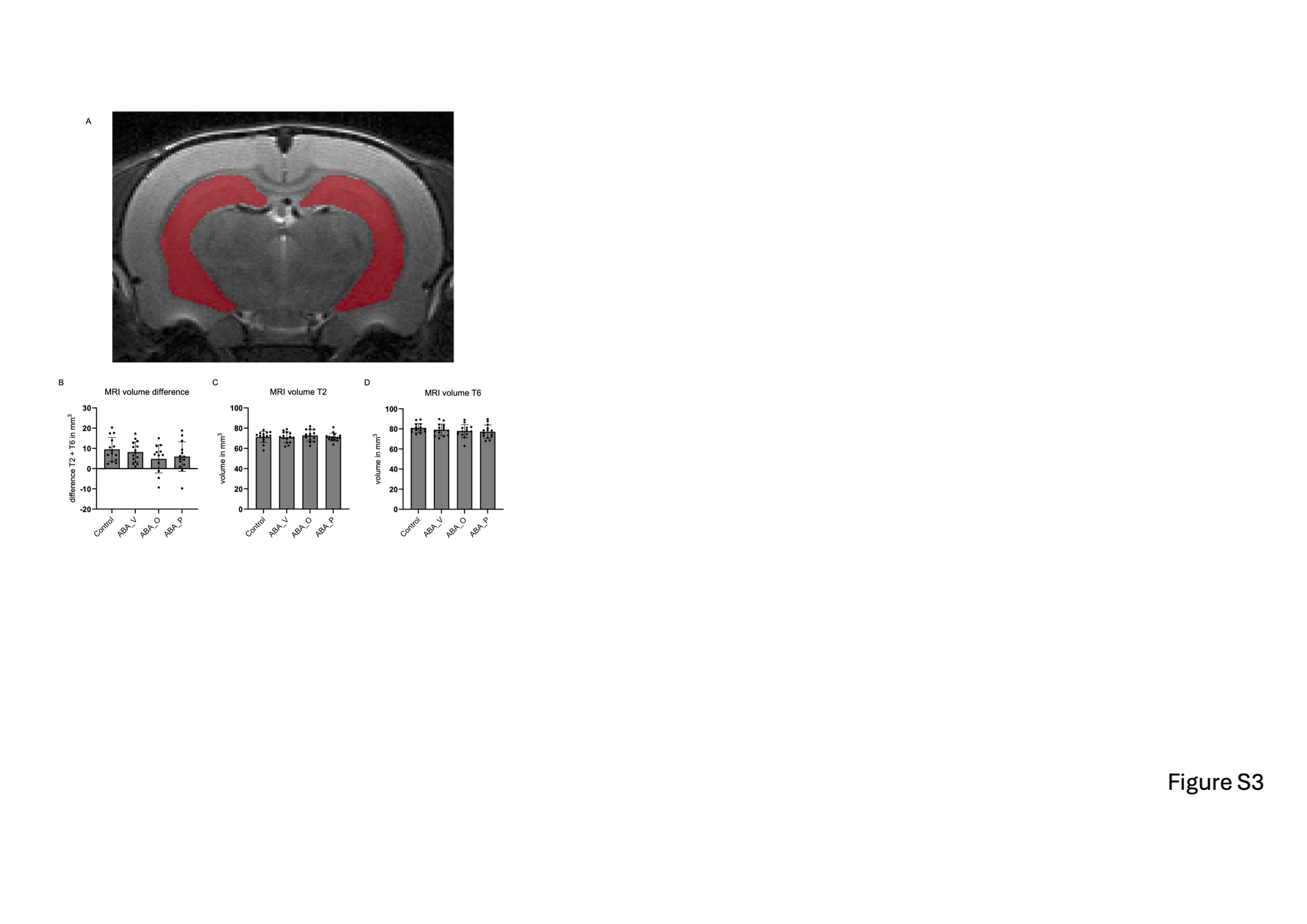

Supplement: Supplementary file 3 — Figure S3: (a) An example of MRI segmentation with ITK‐Snap (ABA_O, after habitation). The segmented hippocampus is displayed in red. (b) MRI volume difference of hippocampus between T2 and T6. (c) MRI volume of hippocampus at M1. (d) MRI volume of hippocampus at M2. *p ≤ 0.05, **p ≤ 0.01, ***p ≤ 0.001. One‐way ANOVA with post hoc Bonferroni test. [file EAT-59-260-s013.tiff]

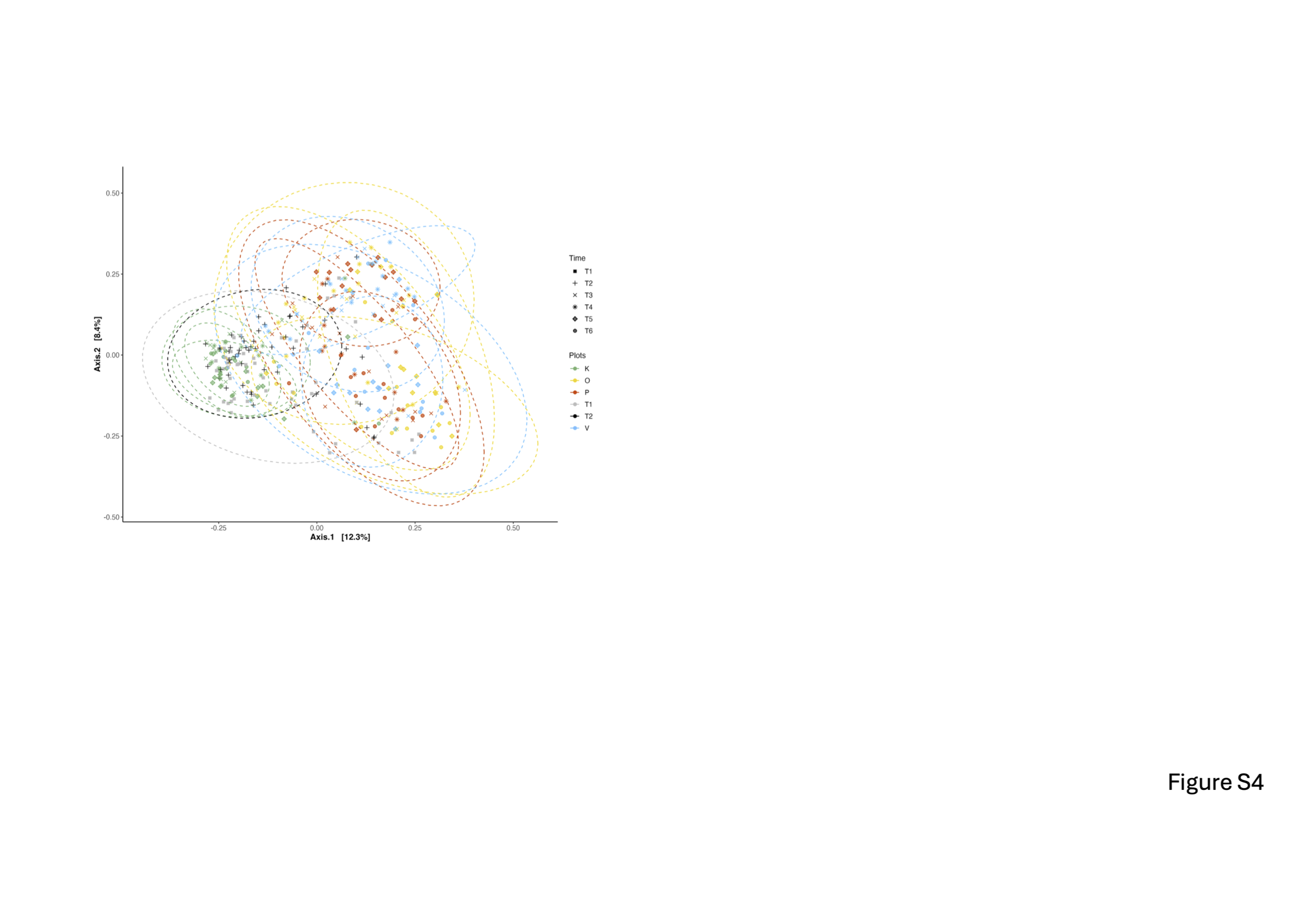

Supplement: Supplementary file 4 — Figure S4: Principal Coordinate Analysis (PCoA) based on Bray–Curtis dissimilarity showing β‐diversity of the microbial communities in the control and treatment groups. Each time point (T1–T6) is represented by a different shape, each group is represented by a different color and dashed ellipses indicate the 95% confidence intervals for each time point. The R 2 and p‐values indicate the proportion of variance explained and statistical significance for each group. [file EAT-59-260-s008.tiff]

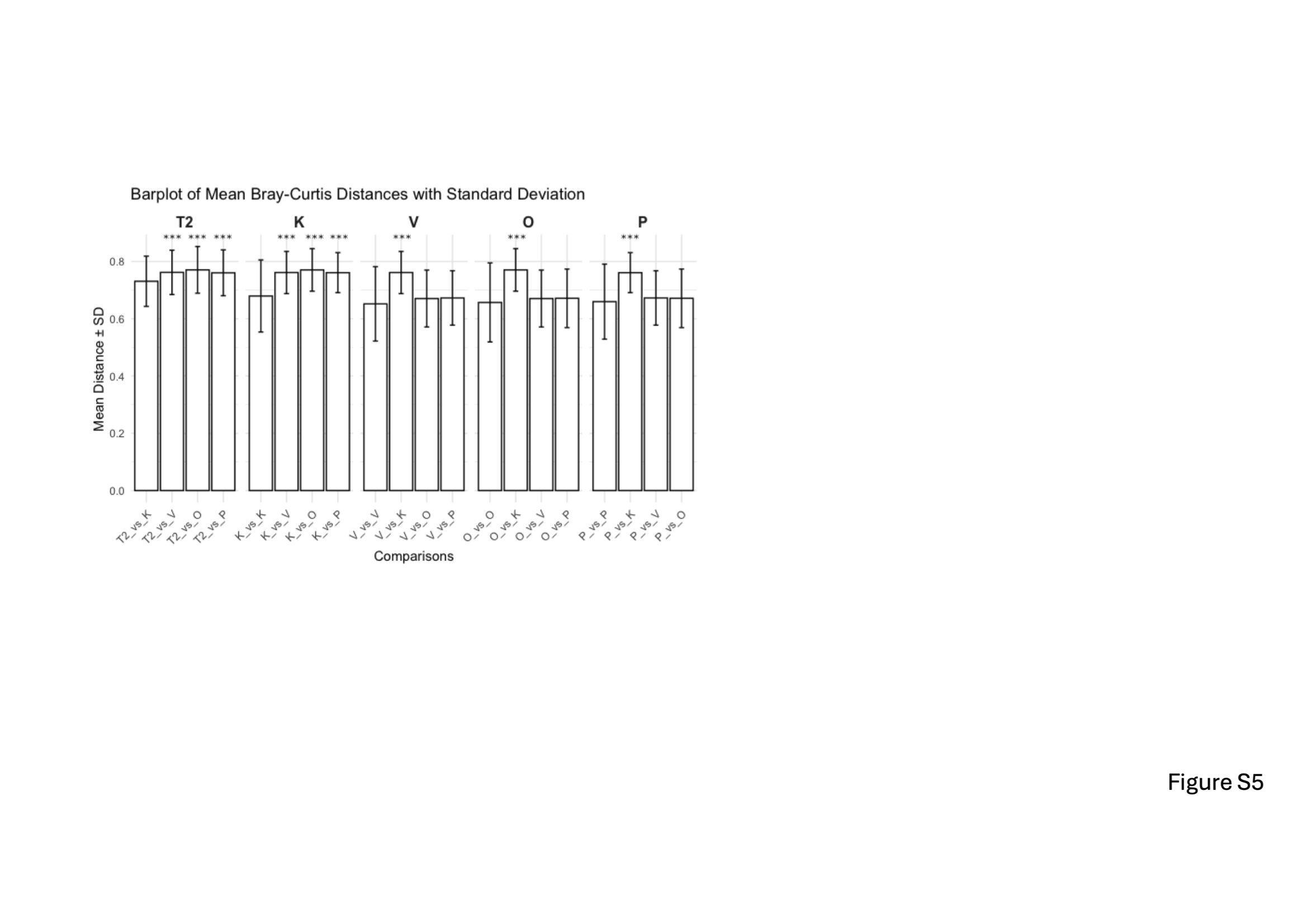

Supplement: Supplementary file 5 — Figure S5: Bar plot of mean Bray‐Curtis distances with standard deviation across experimental groups. The plot compares microbial community dissimilarity between different conditions (T2, K, V, O, P) using Bray‐Curtis distances. K, V, O, and P groups include fecal samples from T3 to T6 timepoints. Significant differences between groups are indicated (*p < 0.05, **p < 0.01, ***p < 0.001). Error bars represent standard deviations, highlighting the variation within each comparison. [file EAT-59-260-s015.tiff]

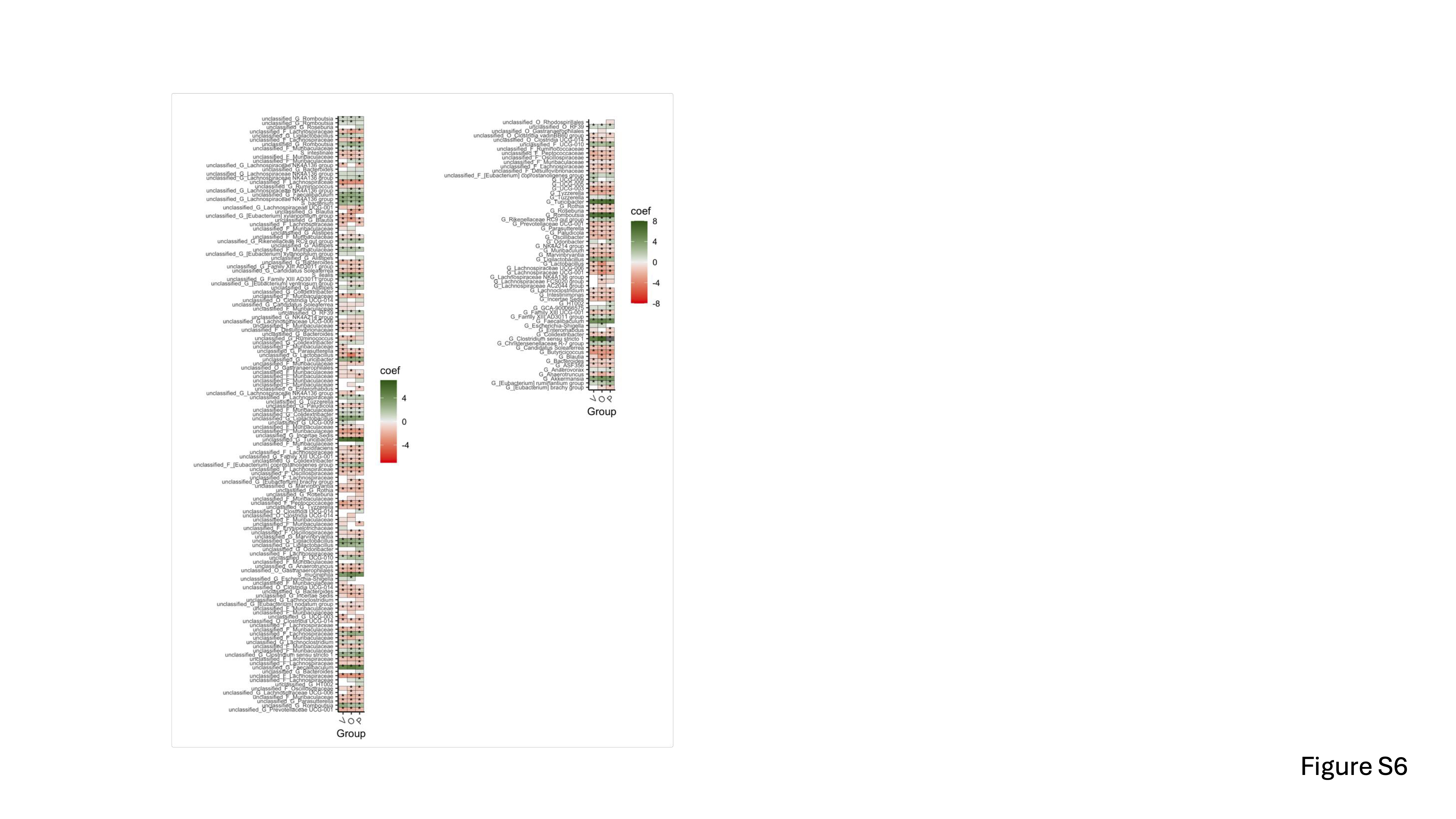

Supplement: Supplementary file 6 — Figure S6: Heatmap of significant ASVs and genera across experimental groups. The heatmap displays the coefficients (coef) of associations between bacterial genera and experimental groups (K, V, O, P), as identified by Maaslin2 analysis. ASVs and Genera are listed along the y‐axis, and groups are on the x‐axis. The color scale represents the direction and magnitude of the associations, with green indicating positive coefficients (taxa enriched in the control group) and red indicating negative coefficients (taxa enriched in the group reported on the x‐axis). Asterisks denote genera with statistically significant associations (q‐value < 0.05). [file EAT-59-260-s010.tiff]

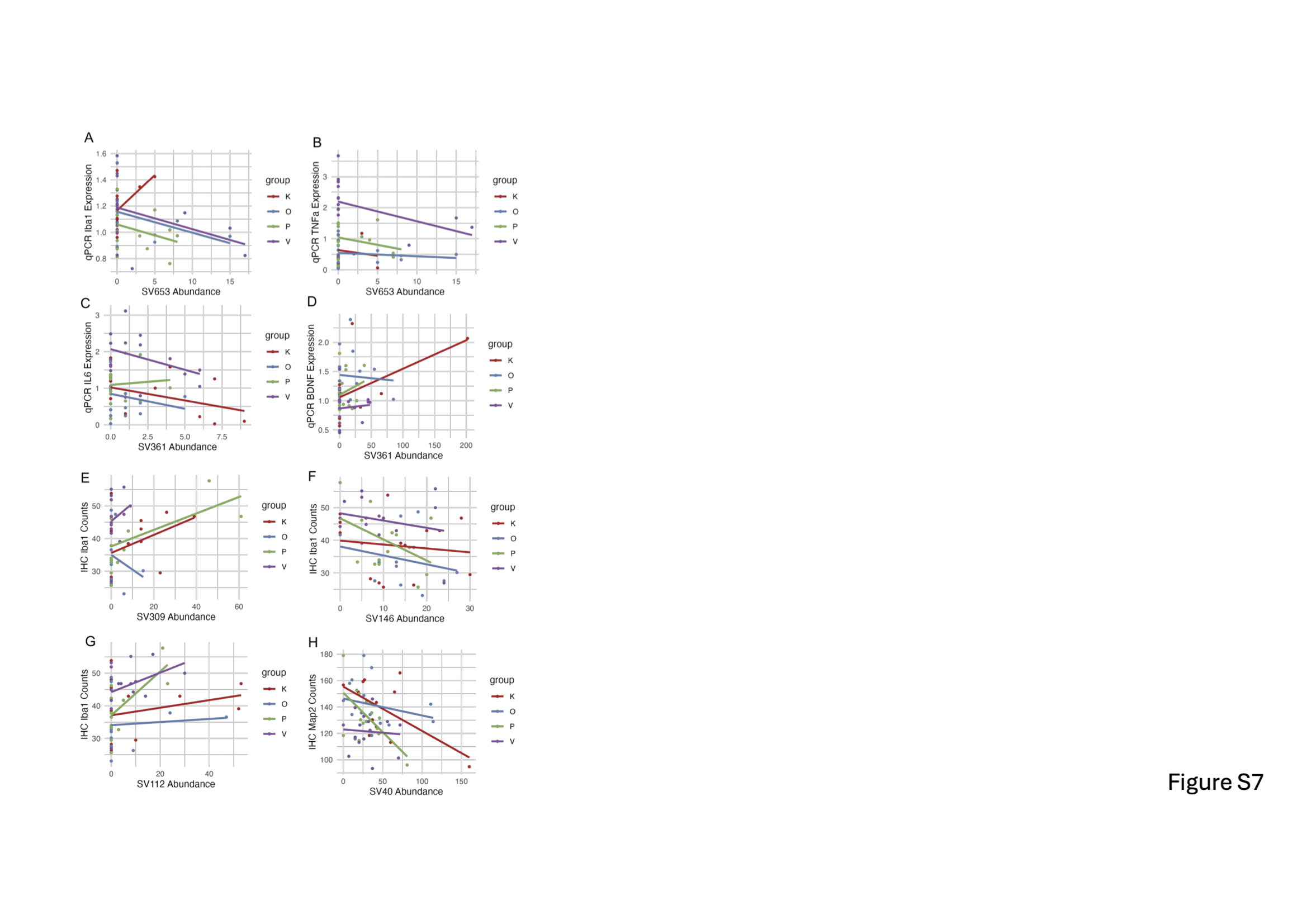

Supplement: Supplementary file 7 — Figure S7: Associations between microbial ASVs and host variables across experimental groups. Each panel shows scatter plots of a microbial ASVs' relative abundance vs. specific host variables, with data points and linear regression lines color‐coded by the experimental group (see legend). Trend lines represent associations within each group. [file EAT-59-260-s014.tiff]

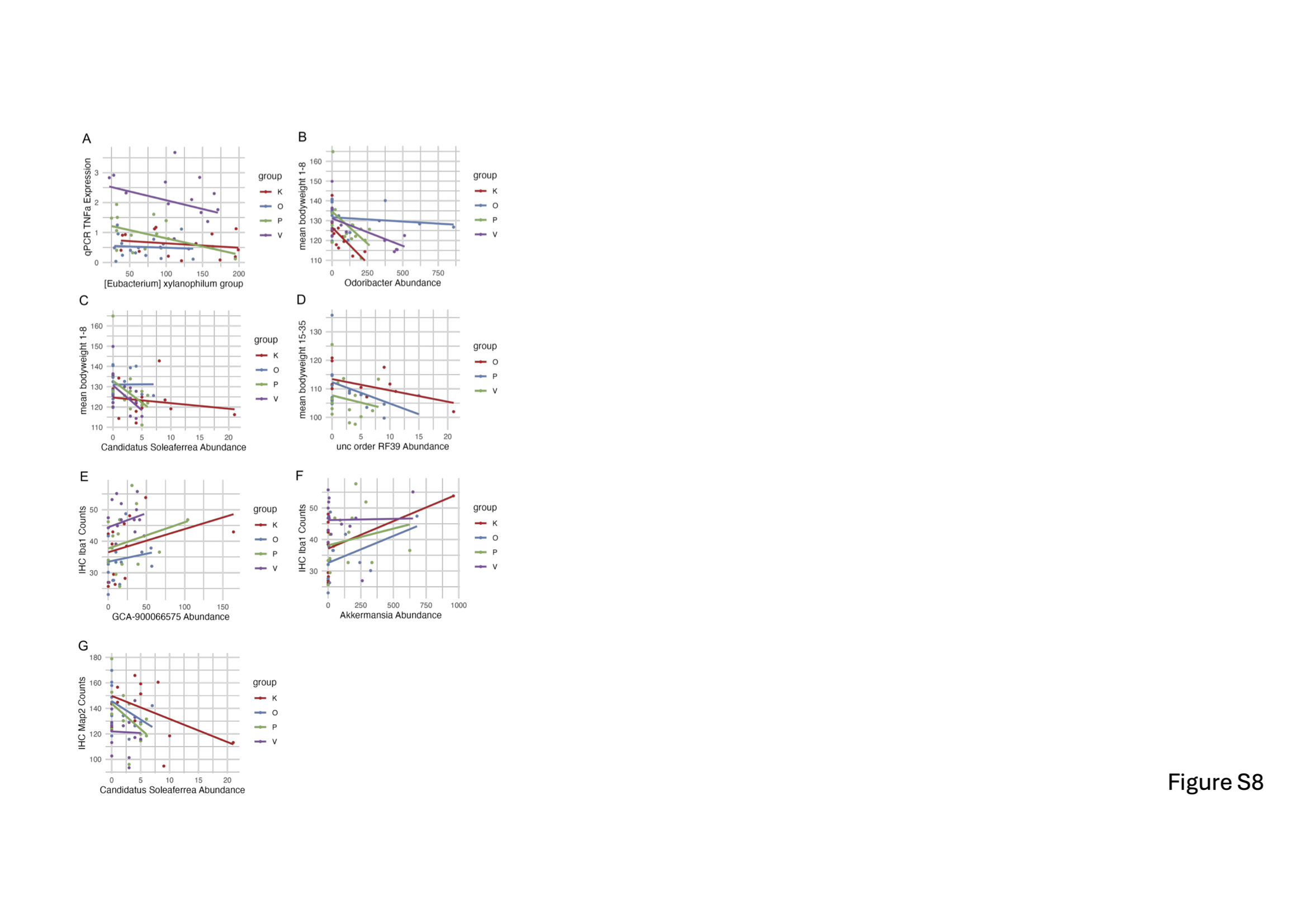

Supplement: Supplementary file 8 — Figure S8: Associations between microbial genera and host variables across experimental groups. Each panel shows scatter plots of the relative abundance of a microbial genus vs. specific host variables, with data points and linear regression lines color‐coded by the experimental group (see legend). Trend lines represent associations within each group. [file EAT-59-260-s007.tiff]

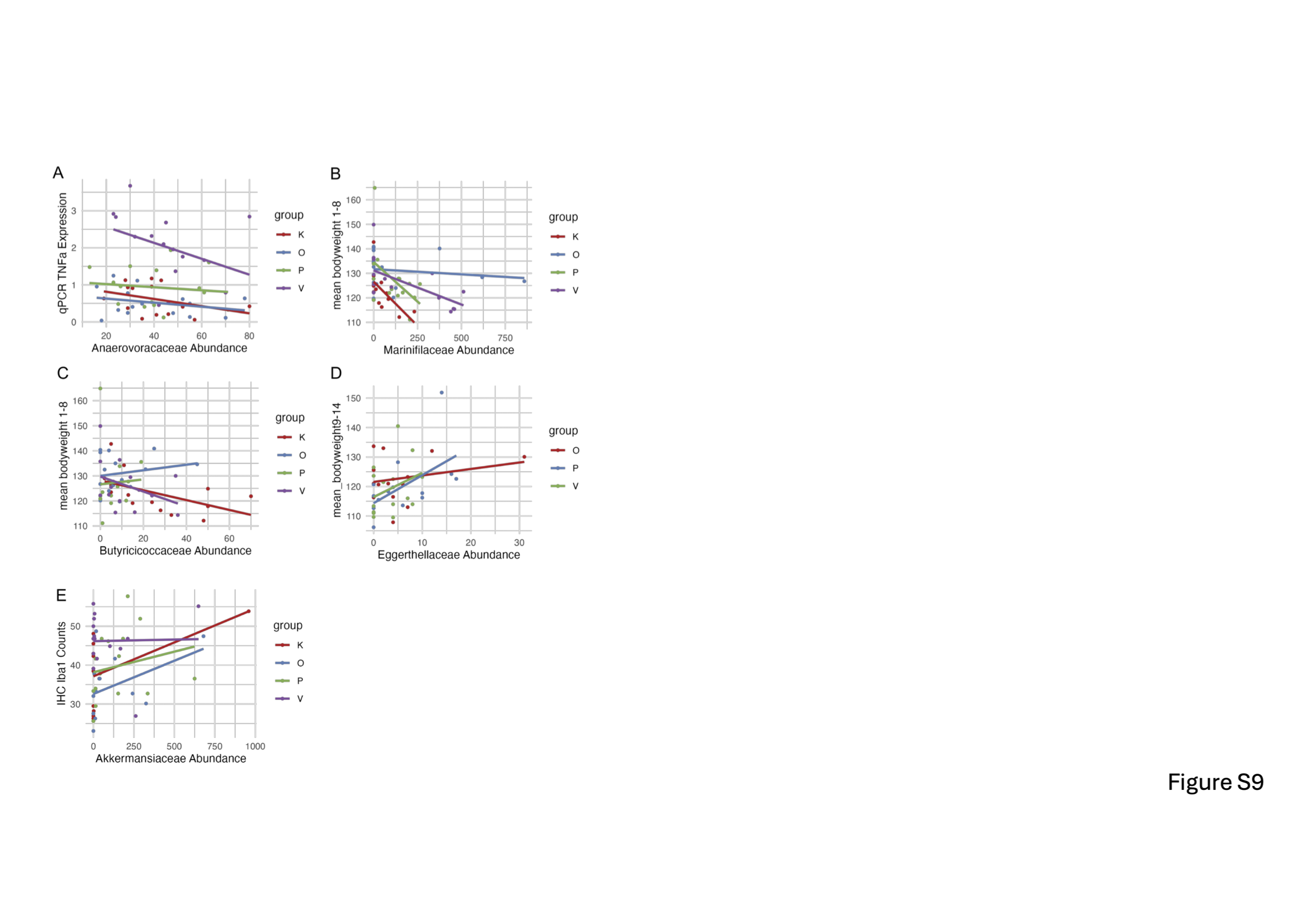

Supplement: Supplementary file 9 — Figure S9: Associations between microbial families and host variables across experimental groups. Each panel shows scatter plots of the relative abundance of a microbial family vs. specific host variables, with data points and linear regression lines color‐coded by the experimental group (see legend). Trend lines represent associations within each group. [file EAT-59-260-s004.tiff]
