# Supplementary material for: The Influence of Omega‐3 Fatty Acids and Probiotics on Hippocampal Inflammation and Glial Cells in a Chronic Anorexia Nervosa Rat Model
Source: Int J Eat Disord. 2025 Oct 18;59(2):260–75. doi: 10.1002/eat.24574 (PMC12884241; doi:10.1002/eat.24574)
Supplement: Supplementary file 10 — Table S1: A list of all primary antibodies used for immunohistochemistry including target, antibody host, concentration, antigen‐retrieval, manufacturer, and catalog number. [file EAT-59-260-s012.docx]

| **antibody** |  | **target** | **antibody host** | **concentration** | **antigen-retrieval** | **manufacturer** | **catalog number** |
| --- | --- | --- | --- | --- | --- | --- | --- |
| GFAP | glial fibrillary acid protein | astroglia | mouse | 1,430555556 | Tris-EDTA, 10 min | Santa Cruz, Dallas, TX, USA | Sc-33673 |
| IBA1 | ionized Calcium-Binding Adapter Molecule 1 | microglia | rabbit | 1:10 000 | Tris-EDTA, 10 min | Wako, Neuss, Germany | 019-19741 |
| TMEM119 | transmembrane protein 119 | microglia | rabbit | 1,430555556 | Citrate, 20 min | Abcam, Cambridge, UK | ab209064 |
| OLIG2 | oligodendrocyte transcription factor 1 | oligodendrocytes | mouse | 0,736111111 | Tris-EDTA, 20 min | Millipore, Darmstadt, Germany | MABN50 |
| MAP2 | microtubule-associated protein 2 | neurons | rabbit | 0,736111111 | Citrate, 20 min | Cell signaling, Danvers, MA, USA | 8707 |
| Ki-67 | Kiel antigen 67 | proliferation | rabbit | 1,083333333 | Citrate, 10 min | Abcam, Cambridge, UK | ab16667 |
| Caspase 3 | Caspase 3 | apoptosis | rabbit | 0,180555556 | Tris-EDTA, 10 min | BioVision, Waltham, MA, USA | 3015-100 |

**Supplementary Table 1**
