# Supplementary material for: The Influence of Omega‐3 Fatty Acids and Probiotics on Hippocampal Inflammation and Glial Cells in a Chronic Anorexia Nervosa Rat Model
Source: Int J Eat Disord. 2025 Oct 18;59(2):260–75. doi: 10.1002/eat.24574 (PMC12884241; doi:10.1002/eat.24574)
Supplement: Supplementary file 11 — Table S2: A list of all primers used for real‐time polymerase chain reaction including annealing temperature, direction, and nucleotide sequence (Thermo Fischer Science). [file EAT-59-260-s009.docx]

| **primer** |  | **annealing temperature** | **direction** | **nucleotide sequence** | **comment** |
| --- | --- | --- | --- | --- | --- |
| Cyclo A | cyclophilin A | 65 °C | s | 5‘-GGCAAATGCTGGACCAAACAC |  |
|  |  |  | as | 5‘-TTAGAGTTGTCCACAGTCGGAGATG |  |
| Gfap | glial fibrillary acid protein | 61 °C | s | 5‘-AGAAAACCGCATCACCATTC |  |
|  |  |  | as | 5‘-GCACACCTCACATCACATCC |  |
| Aif1 | allograft inflammatory factor 1 | 65 °C | s | 5’-TGGAGTTTGATCTGAATGGCAATG | gene encoding IBA1 |
|  |  |  | as | 5’-AGCCACTGGACACCTCTCTA |  |
| Tmem119 | transmembrane protein 119 | 62 °C | s | 5´-CAGTCGGACCGAGACAGTTG |  |
|  |  |  | as | 5´-TTTTCGGGGAAAGAGGACGG |  |
| Itgam | integrin subunit Alpha M | 60 °C | s | 5´-ACAGAGACCAAAGTGGAGCC | gene encoding CD11b |
|  |  |  | as | 5´-GCCACCGGCTTCATTCATCA |  |
| Tnf | tumor necrosis factor a | 64 °C | s | 5´-GGAGGGAGAACAGCAACTCC |  |
|  |  |  | as | 5´-TCTGCCAGTTCCACATCTCG |  |
| Il6 | interleukin 6 | 65 °C | s | 5´-GACTTCCAGCCAGTTGCCTTCTTG |  |
|  |  |  | as | 5´-TGGTCTGTTGTGGGTGGTATCCTC |  |
| Olig1 | oligodendrocyte transcription factor 1 | 63 °C | s | 5´-ACACCACCACGTGTCGGCTA |  |
|  |  |  | as | 5´-CTGCGTCTCTTCTTAGCCCAGA |  |
| Map2 | microtubule-associated protein 2 | 62 °C | s | 5‘-GCAAAGTAAGCCTGGTGA |  |
|  |  |  | as | 5‘-ATCTAAGGGAAGAGTGAAAC |  |
| Bdnf | brain-derived neurotrophic factor | 61 °C | s | 5‘-GCCACTGAAATGCGACTGAA |  |
|  |  |  | as | 5´-CACATCATTCCAGAGCCTGC |  |
| TrkB | tropomyosin receptor kinase B | 65 °C | s | 5´-GCACATCGCTCAGCAAATCG |  |
|  |  |  | as | 5´-ACAACTCCCAGGCTCCAGAC |  |
| Ki-67 | Kiel antigen 67 | 64 °C | s | 5´-CTGCAGAGAAGGTTGGGATAAA |  |
|  |  |  | as | 5´-CTGACTTTGCCCAGAGATGAA |  |
| Casp3 | Caspase 3 | 64 °C | S | 5´-CTTTGCGCCATGCTGAAACT |  |
|  |  |  | as | 5´-ATGACGACCTGGAACATCGG |  |
| miR-103-3p |  | 57 °C | s | 5´-GCAGAGCAGCATTGTACAG |  |
|  |  |  | as | 5´-GGTCCAGTTTTTTTTTTTTTTTCATAG |  |
| miR-107-3p |  | 56 °C | s | 5´-GCAGAGCAGCATTGTACAG |  |
|  |  |  | as | 5´-GGTCCAGTTTTTTTTTTTTTTTGATAG |  |
| miR-124-5p |  | 61 °C | s | 5´-GCAGCGTGTTCACAGC |  |
|  |  |  | as | 5´-TCCAGTTTTTTTTTTTTTTTATCAAGGT |  |
| miR-155-5p |  | 55 °C | s | 5´-CGCAGTTAATGCTAATTGTGATAG |  |
|  |  |  | as | 5´-AGGTCCAGTTTTTTTTTTTTTTTACC |  |
| miR-9-5p |  | 61 °C | s | 5´-GCAGTCTTTGGTTATCTAGCTG |  |
|  |  |  | as | 5´-GGTCCAGTTTTTTTTTTTTTTTTCATAC |  |

**Table S2**
