# Supplementary material for: The Influence of Omega‐3 Fatty Acids and Probiotics on Hippocampal Inflammation and Glial Cells in a Chronic Anorexia Nervosa Rat Model
Source: Int J Eat Disord. 2025 Oct 18;59(2):260–75. doi: 10.1002/eat.24574 (PMC12884241; doi:10.1002/eat.24574)
Supplement: Supplementary file 12 — Table S3: Samples used for microbiome analysis. [file EAT-59-260-s005.docx]

| **SampleID** | **total seq** | **filtered seq** | **time** | **group** | **ratID** | **batch** |  |
| --- | --- | --- | --- | --- | --- | --- | --- |
| ABA_Aachen_K1_T1 | 59422 | 19550 | T1 | K | K1 | B1 |  |
| ABA_Aachen_K1_T2 | 84272 | 17079 | T2 | K | K1 | B1 |  |
| ABA_Aachen_K1_T3 | 70311 | 15436 | T3 | K | K1 | B1 |  |
| ABA_Aachen_K1_T4 | 44868 | 15022 | T4 | K | K1 | B1 |  |
| ABA_Aachen_K1_T5 | 48059 | 10897 | T5 | K | K1 | B1 |  |
| ABA_Aachen_K1_T6 | 91289 | 20097 | T6 | K | K1 | B1 |  |
| ABA_Aachen_K10_T1 | 71186 | 14577 | T1 | K | K10 | B8 |  |
| ABA_Aachen_K10_T2 | 73452 | 14890 | T2 | K | K10 | B8 |  |
| ABA_Aachen_K10_T3 | 59094 | 19354 | T3 | K | K10 | B8 |  |
| ABA_Aachen_K10_T4 | 71800 | 15100 | T4 | K | K10 | B8 |  |
| ABA_Aachen_K10_T5 | 56734 | 13463 | T5 | K | K10 | B8 |  |
| ABA_Aachen_K10_T6 | 44791 | 12611 | T6 | K | K10 | B8 |  |
| ABA_Aachen_K11_T1 | 69680 | 11976 | T1 | K | K11 | B8 |  |
| ABA_Aachen_K11_T2 | 96412 | 18633 | T2 | K | K11 | B8 |  |
| ABA_Aachen_K11_T3 | 50497 | 13063 | T3 | K | K11 | B8 |  |
| ABA_Aachen_K11_T4 | 54144 | 17594 | T4 | K | K11 | B8 |  |
| ABA_Aachen_K11_T5 | 65762 | 17617 | T5 | K | K11 | B8 |  |
| ABA_Aachen_K11_T6 | 54343 | 15469 | T6 | K | K11 | B8 |  |
| ABA_Aachen_K12_T1 | 41619 | 15987 | T1 | K | K12 | B9 |  |
| ABA_Aachen_K12_T2 | 38258 | 17272 | T2 | K | K12 | B9 |  |
| ABA_Aachen_K12_T3 | 59096 | 24216 | T3 | K | K12 | B9 |  |
| ABA_Aachen_K12_T4 | 41714 | 19107 | T4 | K | K12 | B9 |  |
| ABA_Aachen_K12_T5 | 69831 | 20910 | T5 | K | K12 | B9 |  |
| ABA_Aachen_K12_T6 | 58642 | 17900 | T6 | K | K12 | B9 |  |
| ABA_Aachen_K13_T1 | 42272 | 10883 | T1 | K | K13 | B9 |  |
| ABA_Aachen_K13_T2 | 46204 | 19510 | T2 | K | K13 | B9 |  |
| ABA_Aachen_K13_T3 | 59021 | 10958 | T3 | K | K13 | B9 |  |
| ABA_Aachen_K13_T4 | 48224 | 12915 | T4 | K | K13 | B9 |  |
| ABA_Aachen_K13_T5 | 81107 | 23206 | T5 | K | K13 | B9 |  |
| ABA_Aachen_K13_T6 | 86947 | 21313 | T6 | K | K13 | B9 |  |
| ABA_Aachen_K14_T1 | 128506 | 29912 | T1 | K | K14 | B10 |  |
| ABA_Aachen_K14_T2 | 64908 | 24333 | T2 | K | K14 | B10 |  |
| ABA_Aachen_K14_T3 | 71394 | 29588 | T3 | K | K14 | B10 |  |
| ABA_Aachen_K14_T4 | 55613 | 11199 | T4 | K | K14 | B10 |  |
| ABA_Aachen_K14_T5 | 63402 | 16857 | T5 | K | K14 | B10 |  |
| ABA_Aachen_K14_T6 | 102611 | 29713 | T6 | K | K14 | B10 |  |
| ABA_Aachen_K2_T1 | 50227 | 19622 | T1 | K | K2 | B1 |  |
| ABA_Aachen_K2_T2 | 87549 | 14368 | T2 | K | K2 | B1 |  |
| ABA_Aachen_K2_T3 | 57999 | 10613 | T3 | K | K2 | B1 |  |
| ABA_Aachen_K2_T4 | 42676 | 14416 | T4 | K | K2 | B1 |  |
| ABA_Aachen_K2_T5 | 56546 | 13317 | T5 | K | K2 | B1 |  |
| ABA_Aachen_K2_T6 | 62519 | 17535 | T6 | K | K2 | B1 |  |
| ABA_Aachen_K3_T1 | 63257 | 12256 | T1 | K | K3 | B2 |  |
| ABA_Aachen_K3_T2 | 49236 | 19817 | T2 | K | K3 | B2 |  |
| ABA_Aachen_K3_T3 | 46256 | 16351 | T3 | K | K3 | B2 |  |
| ABA_Aachen_K3_T4 | 47917 | 18853 | T4 | K | K3 | B2 |  |
| ABA_Aachen_K3_T5 | 59184 | 12786 | T5 | K | K3 | B2 |  |
| ABA_Aachen_K3_T6 | 49496 | 15072 | T6 | K | K3 | B2 |  |
| ABA_Aachen_K4_T1 | 65738 | 13917 | T1 | K | K4 | B3 |  |
| ABA_Aachen_K4_T3 | 36277 | 14153 | T3 | K | K4 | B3 |  |
| ABA_Aachen_K4_T4 | 57925 | 21185 | T4 | K | K4 | B3 |  |
| ABA_Aachen_K4_T5 | 70413 | 12262 | T5 | K | K4 | B3 |  |
| ABA_Aachen_K4_T6 | 46926 | 12213 | T6 | K | K4 | B3 |  |
| ABA_Aachen_K5_T1 | 56951 | 12203 | T1 | K | K5 | B4 |  |
| ABA_Aachen_K5_T2 | 75962 | 15337 | T2 | K | K5 | B4 |  |
| ABA_Aachen_K5_T3 | 51304 | 11754 | T3 | K | K5 | B4 |  |
| ABA_Aachen_K5_T4 | 37422 | 13990 | T4 | K | K5 | B4 |  |
| ABA_Aachen_K5_T5 | 49488 | 14190 | T5 | K | K5 | B4 |  |
| ABA_Aachen_K5_T6 | 50491 | 16010 | T6 | K | K5 | B4 |  |
| ABA_Aachen_K6_T1 | 48777 | 10790 | T1 | K | K6 | B5 |  |
| ABA_Aachen_K6_T2 | 91242 | 18512 | T2 | K | K6 | B5 |  |
| ABA_Aachen_K6_T3 | 45762 | 11902 | T3 | K | K6 | B5 |  |
| ABA_Aachen_K6_T4 | 53035 | 11229 | T4 | K | K6 | B5 |  |
| ABA_Aachen_K6_T5 | 62438 | 15495 | T5 | K | K6 | B5 |  |
| ABA_Aachen_K6_T6 | 59123 | 17507 | T6 | K | K6 | B5 |  |
| ABA_Aachen_K7_T1 | 37986 | 20432 | T1 | K | K7 | B5 |  |
| ABA_Aachen_K7_T2 | 82848 | 13852 | T2 | K | K7 | B5 |  |
| ABA_Aachen_K7_T3 | 39668 | 16536 | T3 | K | K7 | B5 |  |
| ABA_Aachen_K7_T4 | 72355 | 13354 | T4 | K | K7 | B5 |  |
| ABA_Aachen_K7_T5 | 63033 | 11430 | T5 | K | K7 | B5 |  |
| ABA_Aachen_K7_T6 | 52517 | 15986 | T6 | K | K7 | B5 |  |
| ABA_Aachen_K8_T1 | 38907 | 17202 | T1 | K | K8 | B6 |  |
| ABA_Aachen_K8_T2 | 72811 | 10555 | T2 | K | K8 | B6 |  |
| ABA_Aachen_K8_T3 | 43460 | 16847 | T3 | K | K8 | B6 |  |
| ABA_Aachen_K8_T4 | 62105 | 13601 | T4 | K | K8 | B6 |  |
| ABA_Aachen_K8_T5 | 57957 | 15619 | T5 | K | K8 | B6 |  |
| ABA_Aachen_K8_T6 | 61273 | 15227 | T6 | K | K8 | B6 |  |
| ABA_Aachen_K9_T1 | 52391 | 12295 | T1 | K | K9 | B7 |  |
| ABA_Aachen_K9_T2 | 73084 | 16785 | T2 | K | K9 | B7 |  |
| ABA_Aachen_K9_T3 | 42411 | 16872 | T3 | K | K9 | B7 |  |
| ABA_Aachen_K9_T4 | 64178 | 12630 | T4 | K | K9 | B7 |  |
| ABA_Aachen_K9_T5 | 71344 | 18389 | T5 | K | K9 | B7 |  |
| ABA_Aachen_K9_T6 | 44983 | 12653 | T6 | K | K9 | B7 |  |
| ABA_Aachen_O1_T1 | 50812 | 21166 | T1 | O | O1 | B1 |  |
| ABA_Aachen_O1_T2 | 67370 | 22616 | T2 | O | O1 | B1 |  |
| ABA_Aachen_O1_T3 | 47487 | 20361 | T3 | O | O1 | B1 |  |
| ABA_Aachen_O1_T4 | 66672 | 16758 | T4 | O | O1 | B1 |  |
| ABA_Aachen_O1_T5 | 59350 | 28027 | T5 | O | O1 | B1 |  |
| ABA_Aachen_O1_T6 | 44870 | 15194 | T6 | O | O1 | B1 |  |
| ABA_Aachen_O10_T1 | 48865 | 18435 | T1 | O | O10 | B7 |  |
| ABA_Aachen_O10_T2 | 37335 | 11319 | T2 | O | O10 | B7 |  |
| ABA_Aachen_O10_T3 | 61229 | 23573 | T3 | O | O10 | B7 |  |
| ABA_Aachen_O10_T4 | 51547 | 15688 | T4 | O | O10 | B7 |  |
| ABA_Aachen_O10_T5 | 46622 | 18518 | T5 | O | O10 | B7 |  |
| ABA_Aachen_O10_T6 | 42841 | 13407 | T6 | O | O10 | B7 |  |
| ABA_Aachen_O11_T1 | 34106 | 12868 | T1 | O | O11 | B7 |  |
| ABA_Aachen_O11_T2 | 57465 | 13098 | T2 | O | O11 | B7 |  |
| ABA_Aachen_O11_T3 | 58612 | 14454 | T3 | O | O11 | B7 |  |
| ABA_Aachen_O11_T4 | 64666 | 19653 | T4 | O | O11 | B7 |  |
| ABA_Aachen_O11_T5 | 48796 | 12320 | T5 | O | O11 | B7 |  |
| ABA_Aachen_O11_T6 | 41179 | 11325 | T6 | O | O11 | B7 |  |
| ABA_Aachen_O12_T1 | 42352 | 15803 | T1 | O | O12 | B8 |  |
| ABA_Aachen_O12_T2 | 59464 | 11258 | T2 | O | O12 | B8 |  |
| ABA_Aachen_O12_T3 | 39592 | 10574 | T3 | O | O12 | B8 |  |
| ABA_Aachen_O12_T4 | 47239 | 14997 | T4 | O | O12 | B8 |  |
| ABA_Aachen_O12_T5 | 65578 | 16004 | T5 | O | O12 | B8 |  |
| ABA_Aachen_O12_T6 | 65153 | 16419 | T6 | O | O12 | B8 |  |
| ABA_Aachen_O13_T1 | 40580 | 12421 | T1 | O | O13 | B9 |  |
| ABA_Aachen_O13_T2 | 54784 | 11799 | T2 | O | O13 | B9 |  |
| ABA_Aachen_O13_T3 | 47331 | 18195 | T3 | O | O13 | B9 |  |
| ABA_Aachen_O13_T4 | 45371 | 13550 | T4 | O | O13 | B9 |  |
| ABA_Aachen_O13_T5 | 46746 | 14867 | T5 | O | O13 | B9 |  |
| ABA_Aachen_O13_T6 | 55102 | 10897 | T6 | O | O13 | B9 |  |
| ABA_Aachen_O14_T1 | 47482 | 14667 | T1 | O | O14 | B10 |  |
| ABA_Aachen_O14_T2 | 52922 | 21302 | T2 | O | O14 | B10 |  |
| ABA_Aachen_O14_T3 | 44019 | 11460 | T3 | O | O14 | B10 |  |
| ABA_Aachen_O14_T4 | 57439 | 17160 | T4 | O | O14 | B10 |  |
| ABA_Aachen_O14_T5 | 73420 | 20431 | T5 | O | O14 | B10 |  |
| ABA_Aachen_O14_T6 | 54690 | 12339 | T6 | O | O14 | B10 |  |
| ABA_Aachen_O15_T1 | 41914 | 12390 | T1 | O | O15 | B10 |  |
| ABA_Aachen_O15_T2 | 55221 | 10194 | T2 | O | O15 | B10 |  |
| ABA_Aachen_O15_T3 | 45541 | 10711 | T3 | O | O15 | B10 |  |
| ABA_Aachen_O15_T4 | 61526 | 20845 | T4 | O | O15 | B10 |  |
| ABA_Aachen_O15_T5 | 97493 | 27918 | T5 | O | O15 | B10 |  |
| ABA_Aachen_O15_T6 | 47802 | 10658 | T6 | O | O15 | B10 |  |
| ABA_Aachen_O2_T1 | 59365 | 26611 | T1 | O | O2 | B2 |  |
| ABA_Aachen_O2_T2 | 45946 | 16311 | T2 | O | O2 | B2 |  |
| ABA_Aachen_O2_T3 | 78998 | 31967 | T3 | O | O2 | B2 |  |
| ABA_Aachen_O2_T4 | 45144 | 13322 | T4 | O | O2 | B2 |  |
| ABA_Aachen_O2_T5 | 33202 | 13075 | T5 | O | O2 | B2 |  |
| ABA_Aachen_O2_T6 | 57101 | 16930 | T6 | O | O2 | B2 |  |
| ABA_Aachen_O3_T1 | 49882 | 14077 | T1 | O | O3 | B2 |  |
| ABA_Aachen_O3_T2 | 50063 | 21807 | T2 | O | O3 | B2 |  |
| ABA_Aachen_O3_T3 | 46827 | 19552 | T3 | O | O3 | B2 |  |
| ABA_Aachen_O3_T4 | 50395 | 13738 | T4 | O | O3 | B2 |  |
| ABA_Aachen_O3_T5 | 42337 | 18395 | T5 | O | O3 | B2 |  |
| ABA_Aachen_O3_T6 | 51485 | 14862 | T6 | O | O3 | B2 |  |
| ABA_Aachen_O4_T3 | 40491 | 17047 | T3 | O | O4 | B3 |  |
| ABA_Aachen_O4_T4 | 44236 | 12476 | T4 | O | O4 | B3 |  |
| ABA_Aachen_O4_T5 | 43120 | 18293 | T5 | O | O4 | B3 |  |
| ABA_Aachen_O4_T6 | 69949 | 18979 | T6 | O | O4 | B3 |  |
| ABA_Aachen_O5_T1 | 74581 | 14326 | T1 | O | O5 | B3 |  |
| ABA_Aachen_O5_T3 | 56842 | 10039 | T3 | O | O5 | B3 |  |
| ABA_Aachen_O5_T4 | 55028 | 14031 | T4 | O | O5 | B3 |  |
| ABA_Aachen_O5_T5 | 42467 | 20147 | T5 | O | O5 | B3 |  |
| ABA_Aachen_O5_T6 | 49656 | 14478 | T6 | O | O5 | B3 |  |
| ABA_Aachen_O6_T1 | 98884 | 18146 | T1 | O | O6 | B4 |  |
| ABA_Aachen_O6_T2 | 56035 | 10262 | T2 | O | O6 | B4 |  |
| ABA_Aachen_O6_T3 | 38762 | 16111 | T3 | O | O6 | B4 |  |
| ABA_Aachen_O6_T4 | 58478 | 16811 | T4 | O | O6 | B4 |  |
| ABA_Aachen_O6_T5 | 40861 | 14972 | T5 | O | O6 | B4 |  |
| ABA_Aachen_O6_T6 | 39396 | 12282 | T6 | O | O6 | B4 |  |
| ABA_Aachen_O7_T1 | 56420 | 10158 | T1 | O | O7 | B5 |  |
| ABA_Aachen_O7_T2 | 64396 | 12111 | T2 | O | O7 | B5 |  |
| ABA_Aachen_O7_T4 | 83683 | 23261 | T4 | O | O7 | B5 |  |
| ABA_Aachen_O7_T5 | 47886 | 17436 | T5 | O | O7 | B5 |  |
| ABA_Aachen_O7_T6 | 49191 | 12969 | T6 | O | O7 | B5 |  |
| ABA_Aachen_O8_T1 | 52659 | 18584 | T1 | O | O8 | B6 |  |
| ABA_Aachen_O8_T2 | 40576 | 11480 | T2 | O | O8 | B6 |  |
| ABA_Aachen_O8_T4 | 87312 | 24013 | T4 | O | O8 | B6 |  |
| ABA_Aachen_O8_T5 | 50536 | 17867 | T5 | O | O8 | B6 |  |
| ABA_Aachen_O8_T6 | 51173 | 15853 | T6 | O | O8 | B6 |  |
| ABA_Aachen_O9_T2 | 42810 | 13102 | T2 | O | O9 | B6 |  |
| ABA_Aachen_O9_T3 | 65453 | 30500 | T3 | O | O9 | B6 |  |
| ABA_Aachen_O9_T4 | 68917 | 18111 | T4 | O | O9 | B6 |  |
| ABA_Aachen_O9_T5 | 38602 | 14191 | T5 | O | O9 | B6 |  |
| ABA_Aachen_O9_T6 | 70164 | 21572 | T6 | O | O9 | B6 |  |
| ABA_Aachen_P1_T1 | 45438 | 14902 | T1 | P | P1 | B1 |  |
| ABA_Aachen_P1_T2 | 51968 | 10577 | T2 | P | P1 | B1 |  |
| ABA_Aachen_P1_T3 | 64334 | 14932 | T3 | P | P1 | B1 |  |
| ABA_Aachen_P1_T4 | 44604 | 12402 | T4 | P | P1 | B1 |  |
| ABA_Aachen_P1_T5 | 74752 | 16143 | T5 | P | P1 | B1 |  |
| ABA_Aachen_P1_T6 | 46608 | 11197 | T6 | P | P1 | B1 |  |
| ABA_Aachen_P10_T2 | 45705 | 12622 | T2 | P | P10 | B7 |  |
| ABA_Aachen_P10_T3 | 50557 | 19660 | T3 | P | P10 | B7 |  |
| ABA_Aachen_P10_T4 | 49315 | 11364 | T4 | P | P10 | B7 |  |
| ABA_Aachen_P10_T5 | 57476 | 18016 | T5 | P | P10 | B7 |  |
| ABA_Aachen_P10_T6 | 63009 | 14311 | T6 | P | P10 | B7 |  |
| ABA_Aachen_P11_T1 | 44882 | 11271 | T1 | P | P11 | B8 |  |
| ABA_Aachen_P11_T2 | 64292 | 13152 | T2 | P | P11 | B8 |  |
| ABA_Aachen_P11_T3 | 52658 | 22284 | T3 | P | P11 | B8 |  |
| ABA_Aachen_P11_T4 | 53615 | 15127 | T4 | P | P11 | B8 |  |
| ABA_Aachen_P11_T5 | 49204 | 13657 | T5 | P | P11 | B8 |  |
| ABA_Aachen_P11_T6 | 62329 | 17922 | T6 | P | P11 | B8 |  |
| ABA_Aachen_P12_T1 | 49805 | 15999 | T1 | P | P12 | B8 |  |
| ABA_Aachen_P12_T2 | 66714 | 17268 | T2 | P | P12 | B8 |  |
| ABA_Aachen_P12_T3 | 57505 | 22304 | T3 | P | P12 | B8 |  |
| ABA_Aachen_P12_T4 | 77869 | 19707 | T4 | P | P12 | B8 |  |
| ABA_Aachen_P12_T5 | 72386 | 19811 | T5 | P | P12 | B8 |  |
| ABA_Aachen_P12_T6 | 59358 | 16467 | T6 | P | P12 | B8 |  |
| ABA_Aachen_P13_T1 | 57290 | 18186 | T1 | P | P13 | B9 |  |
| ABA_Aachen_P13_T2 | 57243 | 10283 | T2 | P | P13 | B9 |  |
| ABA_Aachen_P13_T3 | 77092 | 31110 | T3 | P | P13 | B9 |  |
| ABA_Aachen_P13_T4 | 47694 | 12476 | T4 | P | P13 | B9 |  |
| ABA_Aachen_P13_T5 | 58749 | 17151 | T5 | P | P13 | B9 |  |
| ABA_Aachen_P13_T6 | 63995 | 19089 | T6 | P | P13 | B9 |  |
| ABA_Aachen_P14_T1 | 45241 | 19298 | T1 | P | P14 | B10 |  |
| ABA_Aachen_P14_T2 | 56226 | 11752 | T2 | P | P14 | B10 |  |
| ABA_Aachen_P14_T4 | 60646 | 14226 | T4 | P | P14 | B10 |  |
| ABA_Aachen_P14_T5 | 84338 | 25636 | T5 | P | P14 | B10 |  |
| ABA_Aachen_P14_T6 | 51725 | 14199 | T6 | P | P14 | B10 |  |
| ABA_Aachen_P15_T1 | 68621 | 13096 | T1 | P | P15 | B10 |  |
| ABA_Aachen_P15_T2 | 75499 | 21414 | T2 | P | P15 | B10 |  |
| ABA_Aachen_P15_T3 | 82407 | 33534 | T3 | P | P15 | B10 |  |
| ABA_Aachen_P15_T5 | 48339 | 17294 | T5 | P | P15 | B10 |  |
| ABA_Aachen_P15_T6 | 66945 | 17471 | T6 | P | P15 | B10 |  |
| ABA_Aachen_P2_T1 | 62011 | 23859 | T1 | P | P2 | B2 |  |
| ABA_Aachen_P2_T2 | 33458 | 14382 | T2 | P | P2 | B2 |  |
| ABA_Aachen_P2_T3 | 58680 | 10863 | T3 | P | P2 | B2 |  |
| ABA_Aachen_P2_T4 | 56590 | 16605 | T4 | P | P2 | B2 |  |
| ABA_Aachen_P2_T5 | 63269 | 15692 | T5 | P | P2 | B2 |  |
| ABA_Aachen_P2_T6 | 54411 | 18867 | T6 | P | P2 | B2 |  |
| ABA_Aachen_P3_T1 | 62541 | 20115 | T1 | P | P3 | B3 |  |
| ABA_Aachen_P3_T3 | 55220 | 21580 | T3 | P | P3 | B3 |  |
| ABA_Aachen_P3_T4 | 47867 | 14420 | T4 | P | P3 | B3 |  |
| ABA_Aachen_P3_T5 | 74674 | 20940 | T5 | P | P3 | B3 |  |
| ABA_Aachen_P3_T6 | 73324 | 14025 | T6 | P | P3 | B3 |  |
| ABA_Aachen_P4_T1 | 47634 | 10121 | T1 | P | P4 | B3 |  |
| ABA_Aachen_P4_T3 | 41033 | 11882 | T3 | P | P4 | B3 |  |
| ABA_Aachen_P4_T4 | 43819 | 13749 | T4 | P | P4 | B3 |  |
| ABA_Aachen_P4_T5 | 58160 | 16181 | T5 | P | P4 | B3 |  |
| ABA_Aachen_P4_T6 | 47128 | 15833 | T6 | P | P4 | B3 |  |
| ABA_Aachen_P5_T1 | 44758 | 10434 | T1 | P | P5 | B4 |  |
| ABA_Aachen_P5_T2 | 53177 | 10305 | T2 | P | P5 | B4 |  |
| ABA_Aachen_P5_T3 | 53365 | 20315 | T3 | P | P5 | B4 |  |
| ABA_Aachen_P5_T5 | 57951 | 14484 | T5 | P | P5 | B4 |  |
| ABA_Aachen_P5_T6 | 59870 | 19266 | T6 | P | P5 | B4 |  |
| ABA_Aachen_P6_T1 | 45140 | 10695 | T1 | P | P6 | B4 |  |
| ABA_Aachen_P6_T2 | 44422 | 17744 | T2 | P | P6 | B4 |  |
| ABA_Aachen_P6_T3 | 81407 | 36903 | T3 | P | P6 | B4 |  |
| ABA_Aachen_P6_T4 | 62480 | 18925 | T4 | P | P6 | B4 |  |
| ABA_Aachen_P6_T5 | 50691 | 16059 | T5 | P | P6 | B4 |  |
| ABA_Aachen_P6_T6 | 68261 | 18689 | T6 | P | P6 | B4 |  |
| ABA_Aachen_P7_T1 | 61424 | 16248 | T1 | P | P7 | B5 |  |
| ABA_Aachen_P7_T2 | 38961 | 10502 | T2 | P | P7 | B5 |  |
| ABA_Aachen_P7_T3 | 57903 | 21978 | T3 | P | P7 | B5 |  |
| ABA_Aachen_P7_T4 | 68943 | 18840 | T4 | P | P7 | B5 |  |
| ABA_Aachen_P7_T5 | 50841 | 14128 | T5 | P | P7 | B5 |  |
| ABA_Aachen_P7_T6 | 49276 | 14207 | T6 | P | P7 | B5 |  |
| ABA_Aachen_P8_T1 | 53520 | 11573 | T1 | P | P8 | B6 |  |
| ABA_Aachen_P8_T2 | 59058 | 13492 | T2 | P | P8 | B6 |  |
| ABA_Aachen_P8_T3 | 69875 | 26351 | T3 | P | P8 | B6 |  |
| ABA_Aachen_P8_T4 | 68657 | 18428 | T4 | P | P8 | B6 |  |
| ABA_Aachen_P8_T5 | 42474 | 14160 | T5 | P | P8 | B6 |  |
| ABA_Aachen_P8_T6 | 56680 | 15879 | T6 | P | P8 | B6 |  |
| ABA_Aachen_P9_T1 | 66425 | 13372 | T1 | P | P9 | B7 |  |
| ABA_Aachen_P9_T2 | 38591 | 16175 | T2 | P | P9 | B7 |  |
| ABA_Aachen_P9_T3 | 65283 | 25583 | T3 | P | P9 | B7 |  |
| ABA_Aachen_P9_T4 | 59659 | 13641 | T4 | P | P9 | B7 |  |
| ABA_Aachen_P9_T5 | 50688 | 14807 | T5 | P | P9 | B7 |  |
| ABA_Aachen_P9_T6 | 63239 | 19660 | T6 | P | P9 | B7 |  |
| ABA_Aachen_V1_T1 | 66780 | 11633 | T1 | V | V1 | B1 |  |
| ABA_Aachen_V1_T3 | 39964 | 11062 | T3 | V | V1 | B1 |  |
| ABA_Aachen_V1_T4 | 63289 | 25952 | T4 | V | V1 | B1 |  |
| ABA_Aachen_V1_T5 | 57029 | 15940 | T5 | V | V1 | B1 |  |
| ABA_Aachen_V1_T6 | 51369 | 16464 | T6 | V | V1 | B1 |  |
| ABA_Aachen_V10_T1 | 52251 | 13697 | T1 | V | V10 | B6 |  |
| ABA_Aachen_V10_T2 | 46350 | 17071 | T2 | V | V10 | B6 |  |
| ABA_Aachen_V10_T3 | 59422 | 15956 | T3 | V | V10 | B6 |  |
| ABA_Aachen_V10_T5 | 62032 | 16499 | T5 | V | V10 | B6 |  |
| ABA_Aachen_V10_T6 | 60837 | 17185 | T6 | V | V10 | B6 |  |
| ABA_Aachen_V11_T1 | 45495 | 10258 | T1 | V | V11 | B7 |  |
| ABA_Aachen_V11_T2 | 40701 | 15399 | T2 | V | V11 | B7 |  |
| ABA_Aachen_V11_T3 | 36186 | 14341 | T3 | V | V11 | B7 |  |
| ABA_Aachen_V11_T4 | 60143 | 16523 | T4 | V | V11 | B7 |  |
| ABA_Aachen_V11_T5 | 70032 | 19032 | T5 | V | V11 | B7 |  |
| ABA_Aachen_V11_T6 | 43852 | 14256 | T6 | V | V11 | B7 |  |
| ABA_Aachen_V12_T1 | 37664 | 16206 | T1 | V | V12 | B8 |  |
| ABA_Aachen_V12_T2 | 41910 | 15561 | T2 | V | V12 | B8 |  |
| ABA_Aachen_V12_T3 | 42875 | 19555 | T3 | V | V12 | B8 |  |
| ABA_Aachen_V12_T4 | 70525 | 18716 | T4 | V | V12 | B8 |  |
| ABA_Aachen_V12_T5 | 63701 | 19237 | T5 | V | V12 | B8 |  |
| ABA_Aachen_V12_T6 | 55658 | 18411 | T6 | V | V12 | B8 |  |
| ABA_Aachen_V13_T1 | 57339 | 16056 | T1 | V | V13 | B9 |  |
| ABA_Aachen_V13_T2 | 60535 | 19567 | T2 | V | V13 | B9 |  |
| ABA_Aachen_V13_T3 | 38254 | 16341 | T3 | V | V13 | B9 |  |
| ABA_Aachen_V13_T4 | 61649 | 17791 | T4 | V | V13 | B9 |  |
| ABA_Aachen_V13_T5 | 59426 | 16889 | T5 | V | V13 | B9 |  |
| ABA_Aachen_V13_T6 | 56745 | 16920 | T6 | V | V13 | B9 |  |
| ABA_Aachen_V14_T1 | 52112 | 23288 | T1 | V | V14 | B9 |  |
| ABA_Aachen_V14_T2 | 44506 | 10527 | T2 | V | V14 | B9 |  |
| ABA_Aachen_V14_T3 | 44282 | 19047 | T3 | V | V14 | B9 |  |
| ABA_Aachen_V14_T4 | 60803 | 18442 | T4 | V | V14 | B9 |  |
| ABA_Aachen_V14_T5 | 34682 | 13807 | T5 | V | V14 | B9 |  |
| ABA_Aachen_V14_T6 | 52096 | 15738 | T6 | V | V14 | B9 |  |
| ABA_Aachen_V15_T1 | 51089 | 21590 | T1 | V | V15 | B10 |  |
| ABA_Aachen_V15_T2 | 44785 | 16509 | T2 | V | V15 | B10 |  |
| ABA_Aachen_V15_T3 | 51199 | 20285 | T3 | V | V15 | B10 |  |
| ABA_Aachen_V15_T4 | 63541 | 18545 | T4 | V | V15 | B10 |  |
| ABA_Aachen_V15_T5 | 36886 | 16623 | T5 | V | V15 | B10 |  |
| ABA_Aachen_V15_T6 | 48423 | 17426 | T6 | V | V15 | B10 |  |
| ABA_Aachen_V2_T1 | 73706 | 15712 | T1 | V | V2 | B1 |  |
| ABA_Aachen_V2_T2 | 50774 | 22613 | T2 | V | V2 | B1 |  |
| ABA_Aachen_V2_T3 | 46864 | 12244 | T3 | V | V2 | B1 |  |
| ABA_Aachen_V2_T4 | 58350 | 16067 | T4 | V | V2 | B1 |  |
| ABA_Aachen_V2_T5 | 48919 | 15484 | T5 | V | V2 | B1 |  |
| ABA_Aachen_V2_T6 | 59364 | 18033 | T6 | V | V2 | B1 |  |
| ABA_Aachen_V3_T1 | 67236 | 15392 | T1 | V | V3 | B2 |  |
| ABA_Aachen_V3_T2 | 72738 | 34266 | T2 | V | V3 | B2 |  |
| ABA_Aachen_V3_T3 | 68059 | 29847 | T3 | V | V3 | B2 |  |
| ABA_Aachen_V3_T4 | 40039 | 11596 | T4 | V | V3 | B2 |  |
| ABA_Aachen_V3_T5 | 52930 | 15824 | T5 | V | V3 | B2 |  |
| ABA_Aachen_V3_T6 | 49822 | 14146 | T6 | V | V3 | B2 |  |
| ABA_Aachen_V4_T1 | 66375 | 16420 | T1 | V | V4 | B2 |  |
| ABA_Aachen_V4_T2 | 57853 | 26872 | T2 | V | V4 | B2 |  |
| ABA_Aachen_V4_T3 | 41135 | 10559 | T3 | V | V4 | B2 |  |
| ABA_Aachen_V4_T4 | 66770 | 17891 | T4 | V | V4 | B2 |  |
| ABA_Aachen_V4_T5 | 62225 | 15297 | T5 | V | V4 | B2 |  |
| ABA_Aachen_V4_T6 | 58494 | 17577 | T6 | V | V4 | B2 |  |
| ABA_Aachen_V5_T1 | 45135 | 17216 | T1 | V | V5 | B3 |  |
| ABA_Aachen_V5_T3 | 47082 | 21118 | T3 | V | V5 | B3 |  |
| ABA_Aachen_V5_T4 | 47471 | 12624 | T4 | V | V5 | B3 |  |
| ABA_Aachen_V5_T5 | 73965 | 19322 | T5 | V | V5 | B3 |  |
| ABA_Aachen_V5_T6 | 79732 | 19731 | T6 | V | V5 | B3 |  |
| ABA_Aachen_V6_T1 | 78899 | 11525 | T1 | V | V6 | B4 |  |
| ABA_Aachen_V6_T2 | 59093 | 23135 | T2 | V | V6 | B4 |  |
| ABA_Aachen_V6_T3 | 55451 | 23965 | T3 | V | V6 | B4 |  |
| ABA_Aachen_V6_T4 | 54222 | 17175 | T4 | V | V6 | B4 |  |
| ABA_Aachen_V6_T5 | 69738 | 20888 | T5 | V | V6 | B4 |  |
| ABA_Aachen_V6_T6 | 73546 | 20768 | T6 | V | V6 | B4 |  |
| ABA_Aachen_V7_T1 | 66510 | 13673 | T1 | V | V7 | B5 |  |
| ABA_Aachen_V7_T2 | 51958 | 16525 | T2 | V | V7 | B5 |  |
| ABA_Aachen_V7_T3 | 57739 | 24783 | T3 | V | V7 | B5 |  |
| ABA_Aachen_V7_T4 | 46965 | 12175 | T4 | V | V7 | B5 |  |
| ABA_Aachen_V7_T5 | 58514 | 17703 | T5 | V | V7 | B5 |  |
| ABA_Aachen_V7_T6 | 60042 | 18331 | T6 | V | V7 | B5 |  |
| ABA_Aachen_V8_T1 | 72878 | 12247 | T1 | V | V8 | B5 |  |
| ABA_Aachen_V8_T2 | 42511 | 12769 | T2 | V | V8 | B5 |  |
| ABA_Aachen_V8_T3 | 78946 | 30289 | T3 | V | V8 | B5 |  |
| ABA_Aachen_V8_T4 | 61596 | 15260 | T4 | V | V8 | B5 |  |
| ABA_Aachen_V8_T5 | 70480 | 14711 | T5 | V | V8 | B5 |  |
| ABA_Aachen_V8_T6 | 70573 | 20626 | T6 | V | V8 | B5 |  |
| ABA_Aachen_V9_T1 | 73942 | 11918 | T1 | V | V9 | B6 |  |
| ABA_Aachen_V9_T2 | 42013 | 15940 | T2 | V | V9 | B6 |  |
| ABA_Aachen_V9_T3 | 42183 | 13079 | T3 | V | V9 | B6 |  |
| ABA_Aachen_V9_T4 | 49533 | 13605 | T4 | V | V9 | B6 |  |
| ABA_Aachen_V9_T5 | 46427 | 17958 | T5 | V | V9 | B6 |  |
| ABA_Aachen_V9_T6 | 49310 | 13652 | T6 | V | V9 | B6 |  |
| psycobiotic89_1 | 108594 | 32604 | psyco | P | psyco | B0 | Fecal samples from rat psyco at timepoint psyco belonging to the P group. |
| psycobiotic89_2 | 84822 | 26248 | psyco | P | psyco | B0 | Fecal samples from rat psyco at timepoint psyco belonging to the P group. |
| psycobiotic89_3 | 132077 | 51304 | psyco | P | psyco | B0 | Fecal samples from rat psyco at timepoint psyco belonging to the P group. |

**Table S3**
