# Supplementary material for: The Influence of Omega‐3 Fatty Acids and Probiotics on Hippocampal Inflammation and Glial Cells in a Chronic Anorexia Nervosa Rat Model
Source: Int J Eat Disord. 2025 Oct 18;59(2):260–75. doi: 10.1002/eat.24574 (PMC12884241; doi:10.1002/eat.24574)
Supplement: Supplementary file 13 — Table S4: Abundance table of 16S rRNA amplicon sequence variants (ASVs) with taxonomic annotation of ASVs. [file EAT-59-260-s006.pdf]

Table S4

SV_n	Kingdom	Phylum	Class	Order	Family	Genus	Species	sequences
SV1	K_Bacteria	P_Bacteroidota	C_Bacteroidia	O_Bacteroidales	F_Prevotellaceae	G_Prevotellaceae UCG-001	unclassified_G_Prevotellaceae UCG-001	
SV2	K_Bacteria	P_Firmicutes	C_Bacilli	O_Lactobacillales	F_Lactobacillaceae	G_Ligilactobacillus	unclassified_G_Ligilactobacillus	
SV3	K_Bacteria	P_Firmicutes	C_Bacilli	O_Erysipelotrichales	F_Erysipelotrichaceae	G_Turicibacter	unclassified_G_Turicibacter	
SV4	K_Bacteria	P_Firmicutes	C_Bacilli	O_Lactobacillales	F_Lactobacillaceae	G_Lactobacillus	unclassified_G_Lactobacillus	
SV5	K_Bacteria	P_Firmicutes	C_Clostridia	O_Peptostreptococcales-Tissierellales	F_Peptostreptococcaceae	G_Romboutsia	S_ilealis	
SV6	K_Bacteria	P_Bacteroidota	C_Bacteroidia	O_Bacteroidales	F_Rikenellaceae	G_Alistipes	unclassified_G_Alistipes	
SV7	K_Bacteria	P_Firmicutes	C_Clostridia	O_Lachnospirales	F_Lachnospiraceae	G_Lachnospiraceae NK4A136 group	S_bacterium	
SV8	K_Bacteria	P_Firmicutes	C_Clostridia	O_Oscillospirales	F_Ruminococcaceae	G_Ruminococcus	unclassified_G_Ruminococcus	
SV9	K_Bacteria	P_Firmicutes	C_Clostridia	O_Peptostreptococcales-Tissierellales	F_Peptostreptococcaceae	G_Romboutsia	unclassified_G_Romboutsia	
SV10	K_Bacteria	P_Firmicutes	C_Clostridia	O_Peptostreptococcales-Tissierellales	F_Peptostreptococcaceae	G_Romboutsia	unclassified_G_Romboutsia	
SV11	K_Bacteria	P_Firmicutes	C_Bacilli	O_Lactobacillales	F_Lactobacillaceae	G_HT002	unclassified_G_HT002	
SV12	K_Bacteria	P_Firmicutes	C_Bacilli	O_Erysipelotrichales	F_Erysipelotrichaceae	G_Faecalibaculum	unclassified_G_Faecalibaculum	
SV13	K_Bacteria	P_Firmicutes	C_Clostridia	O_Clostridiales	F_Clostridiaceae	G_Clostridium sensu stricto 1	unclassified_G_Clostridium sensu stricto 1	
SV14	K_Bacteria	P_Bacteroidota	C_Bacteroidia	O_Bacteroidales	F_Muribaculaceae	unclassified_F_Muribaculaceae	unclassified_F_Muribaculaceae	
SV15	K_Bacteria	P_Firmicutes	C_Clostridia	O_Clostridiales	F_Clostridiaceae	G_Clostridium sensu stricto 1	unclassified_G_Clostridium sensu stricto 1	
SV16	K_Bacteria	P_Firmicutes	C_Clostridia	O_Lachnospirales	F_Lachnospiraceae	G_Lachnospiraceae NK4A136 group	unclassified_G_Lachnospiraceae NK4A136 group	
SV17	K_Bacteria	P_Bacteroidota	C_Bacteroidia	O_Bacteroidales	F_Bacteroidaceae	G_Bacteroides	unclassified_G_Bacteroides	
SV18	K_Bacteria	P_Verrucomicrobiota	C_Verrucomicrobiae	O_Verrucomicrobiales	F_Akkermansiaceae	G_Akkermansia	S_muciniphila	
SV19	K_Bacteria	P_Bacteroidota	C_Bacteroidia	O_Bacteroidales	F_Marinifilaceae	G_Odoribacter	unclassified_G_Odoribacter	
SV20	K_Bacteria	P_Firmicutes	C_Bacilli	O_Lactobacillales	F_Lactobacillaceae	G_Ligilactobacillus	unclassified_G_Ligilactobacillus	
SV21	K_Bacteria	P_Firmicutes	C_Clostridia	O_Peptostreptococcales-Tissierellales	F_Peptostreptococcaceae	G_Romboutsia	S_ilealis	
SV22	K_Bacteria	P_Bacteroidota	C_Bacteroidia	O_Bacteroidales	F_Muribaculaceae	unclassified_F_Muribaculaceae	unclassified_F_Muribaculaceae	
SV23	K_Bacteria	P_Firmicutes	C_Clostridia	O_Lachnospirales	F_Lachnospiraceae	G_Roseburia	unclassified_G_Roseburia	
SV24	K_Bacteria	P_Firmicutes	C_Clostridia	O_Lachnospirales	F_Lachnospiraceae	G_Marvinbryantia	unclassified_G_Marvinbryantia	
SV25	K_Bacteria	P_Firmicutes	C_Clostridia	O_Lachnospirales	F_Lachnospiraceae	G_Lachnospiraceae NK4A136 group	unclassified_G_Lachnospiraceae NK4A136 group	
SV26	K_Bacteria	P_Firmicutes	C_Clostridia	O_Oscillospirales	F_[Eubacterium] coprostanoligenes group	unclassified_F_[Eubacterium] coprostanoligenes grou	unclassified_F_[Eubacterium] coprostanoligenes group	
SV27	K_Bacteria	P_Firmicutes	C_Bacilli	O_Lactobacillales	F_Lactobacillaceae	G_Lacticaseibacillus	unclassified_G_Lacticaseibacillus	
SV28	K_Bacteria	P_Firmicutes	C_Clostridia	O_Clostridiales	F_Clostridiaceae	G_Clostridium sensu stricto 1	unclassified_G_Clostridium sensu stricto 1	
SV29	K_Bacteria	P_Bacteroidota	C_Bacteroidia	O_Bacteroidales	F_Bacteroidaceae	G_Bacteroides	S_acidifaciens	
SV30	K_Bacteria	P_Bacteroidota	C_Bacteroidia	O_Bacteroidales	F_Prevotellaceae	G_Alloprevotella	unclassified_G_Alloprevotella	
SV31	K_Bacteria	P_Actinobacteriota	C_Actinobacteria	O_Bifidobacteriales	F_Bifidobacteriaceae	G_Bifidobacterium	S_animalis	
SV32	K_Bacteria	P_Firmicutes	C_Bacilli	O_Lactobacillales	F_Lactobacillaceae	G_Ligilactobacillus	unclassified_G_Ligilactobacillus	
SV33	K_Bacteria	P_Bacteroidota	C_Bacteroidia	O_Bacteroidales	F_Muribaculaceae	unclassified_F_Muribaculaceae	unclassified_F_Muribaculaceae	
SV34	K_Bacteria	P_Firmicutes	C_Clostridia	O_Lachnospirales	F_Lachnospiraceae	G_Roseburia	unclassified_G_Roseburia	
SV35	K_Bacteria	P_Firmicutes	C_Clostridia	O_Lachnospirales	F_Lachnospiraceae	G_Lachnospiraceae NK4A136 group	unclassified_G_Lachnospiraceae NK4A136 group	
SV36	K_Bacteria	P_Firmicutes	C_Clostridia	O_Oscillospirales	F_Ruminococcaceae	G_Ruminococcus	unclassified_G_Ruminococcus	
SV37	K_Bacteria	P_Bacteroidota	C_Bacteroidia	O_Bacteroidales	F_Muribaculaceae	unclassified_F_Muribaculaceae	unclassified_F_Muribaculaceae	
SV38	K_Bacteria	P_Bacteroidota	C_Bacteroidia	O_Bacteroidales	F_Muribaculaceae	unclassified_F_Muribaculaceae	unclassified_F_Muribaculaceae	
SV39	K_Bacteria	P_Firmicutes	C_Bacilli	O_Erysipelotrichales	F_Erysipelotrichaceae	G_Turicibacter	unclassified_G_Turicibacter	
SV40	K_Bacteria	P_Proteobacteria	C_Gammaproteobacteria	O_Burkholderiales	F_Sutterellaceae	G_Parasutterella	unclassified_G_Parasutterella	
SV41	K_Bacteria	P_Firmicutes	C_Clostridia	O_Oscillospirales	F_Ruminococcaceae	G_Ruminococcus	unclassified_G_Ruminococcus	
SV42	K_Bacteria	P_Desulfobacterota	C_Desulfovibrionia	O_Desulfovibrionales	F_Desulfovibrionaceae	unclassified_F_Desulfovibrionaceae	unclassified_F_Desulfovibrionaceae	
SV43	K_Bacteria	P_Bacteroidota	C_Bacteroidia	O_Bacteroidales	F_Muribaculaceae	unclassified_F_Muribaculaceae	unclassified_F_Muribaculaceae	
SV44	K_Bacteria	P_Firmicutes	C_Clostridia	O_Lachnospirales	F_Lachnospiraceae	G_Lachnospiraceae UCG-006	unclassified_G_Lachnospiraceae UCG-006	
SV45	K_Bacteria	P_Firmicutes	C_Clostridia	O_Peptostreptococcales-Tissierellales	F_Peptostreptococcaceae	G_Romboutsia	unclassified_G_Romboutsia	
SV46	K_Bacteria	P_Bacteroidota	C_Bacteroidia	O_Bacteroidales	F_Muribaculaceae	unclassified_F_Muribaculaceae	unclassified_F_Muribaculaceae	
SV47	K_Bacteria	P_Bacteroidota	C_Bacteroidia	O_Bacteroidales	F_Rikenellaceae	G_Alistipes	unclassified_G_Alistipes	
SV48	K_Bacteria	P_Firmicutes	C_Clostridia	O_Lachnospirales	F_Lachnospiraceae	G_Lachnospiraceae NK4A136 group	S_bacterium	
SV49	K_Bacteria	P_Firmicutes	C_Clostridia	O_Peptostreptococcales-Tissierellales	F_Peptostreptococcaceae	G_Romboutsia	S_ilealis	
SV50	K_Bacteria	P_Firmicutes	C_Clostridia	O_Lachnospirales	F_Lachnospiraceae	G_[Eubacterium] ruminantium group	unclassified_G_[Eubacterium] ruminantium group	
SV51	K_Bacteria	P_Cyanobacteria	C_Vampirivibrionia	O_Gastranaerophilales	unclassified_O_Gastranaerophilales	unclassified_O_Gastranaerophilales	unclassified_O_Gastranaerophilales	
SV52	K_Bacteria	P_Bacteroidota	C_Bacteroidia	O_Bacteroidales	F_Bacteroidaceae	G_Bacteroides	unclassified_G_Bacteroides	
SV53	K_Bacteria	P_Bacteroidota	C_Bacteroidia	O_Bacteroidales	F_Rikenellaceae	G_Alistipes	unclassified_G_Alistipes	
SV54	K_Bacteria	P_Firmicutes	C_Clostridia	O_Lachnospirales	F_Lachnospiraceae	G_[Eubacterium] xylanophilum group	unclassified_G_[Eubacterium] xylanophilum group	
SV55	K_Bacteria	P_Bacteroidota	C_Bacteroidia	O_Bacteroidales	F_Muribaculaceae	unclassified_F_Muribaculaceae	unclassified_F_Muribaculaceae	
SV56	K_Bacteria	P_Bacteroidota	C_Bacteroidia	O_Bacteroidales	F_Rikenellaceae	G_Alistipes	unclassified_G_Alistipes	
SV57	K_Bacteria	P_Bacteroidota	C_Bacteroidia	O_Bacteroidales	F_Rikenellaceae	G_Rikenellaceae RC9 gut group	unclassified_G_Rikenellaceae RC9 gut group	
SV58	K_Bacteria	P_Bacteroidota	C_Bacteroidia	O_Bacteroidales	F_Muribaculaceae	unclassified_F_Muribaculaceae	unclassified_F_Muribaculaceae	
SV59	K_Bacteria	P_Firmicutes	C_Clostridia	O_Peptostreptococcales-Tissierellales	F_Peptostreptococcaceae	G_Romboutsia	unclassified_G_Romboutsia	
SV60	K_Bacteria	P_Firmicutes	C_Clostridia	O_Peptostreptococcales-Tissierellales	F_Peptostreptococcaceae	G_Romboutsia	S_ilealis	
SV61	K_Bacteria	P_Bacteroidota	C_Bacteroidia	O_Bacteroidales	F_Muribaculaceae	unclassified_F_Muribaculaceae	unclassified_F_Muribaculaceae	
SV62	K_Bacteria	P_Firmicutes	C_Clostridia	O_Lachnospirales	F_Lachnospiraceae	unclassified_F_Lachnospiraceae	unclassified_F_Lachnospiraceae	
SV63	K_Bacteria	P_Firmicutes	C_Clostridia	O_Lachnospirales	F_Lachnospiraceae	G_Lachnospiraceae NK4A136 group	unclassified_G_Lachnospiraceae NK4A136 group	
SV64	K_Bacteria	P_Firmicutes	C_Clostridia	O_Lachnospirales	F_Lachnospiraceae	G_Blautia	unclassified_G_Blautia	
SV65	K_Bacteria	P_Firmicutes	C_Clostridia	O_Lachnospirales	F_Lachnospiraceae	G_[Eubacterium] xylanophilum group	unclassified_G_[Eubacterium] xylanophilum group	
SV66	K_Bacteria	P_Firmicutes	C_Clostridia	O_Lachnospirales	F_Lachnospiraceae	G_Blautia	unclassified_G_Blautia	
SV67	K_Bacteria	P_Firmicutes	C_Clostridia	O_Lachnospirales	F_Lachnospiraceae	G_Lachnospiraceae UCG-001	unclassified_G_Lachnospiraceae UCG-001	
SV68	K_Bacteria	P_Firmicutes	C_Clostridia	O_Oscillospirales	F_Ruminococcaceae	G_Ruminococcus	unclassified_G_Ruminococcus	
SV69	K_Bacteria	P_Bacteroidota	C_Bacteroidia	O_Bacteroidales	F_Muribaculaceae	unclassified_F_Muribaculaceae	unclassified_F_Muribaculaceae	
SV70	K_Bacteria	P_Firmicutes	C_Clostridia	O_Lachnospirales	F_Lachnospiraceae	G_Lachnospiraceae NK4A136 group	unclassified_G_Lachnospiraceae NK4A136 group	
SV71	K_Bacteria	P_Firmicutes	C_Bacilli	O_Erysipelotrichales	F_Erysipelotrichaceae	G_Faecalibaculum	unclassified_G_Faecalibaculum	
SV72	K_Bacteria	P_Firmicutes	C_Clostridia	O_Lachnospirales	F_Lachnospiraceae	G_Lachnospiraceae NK4A136 group	unclassified_G_Lachnospiraceae NK4A136 group	
SV73	K_Bacteria	P_Firmicutes	C_Clostridia	O_Oscillospirales	F_Ruminococcaceae	G_Ruminococcus	unclassified_G_Ruminococcus	
SV74	K_Bacteria	P_Firmicutes	C_Clostridia	O_Lachnospirales	F_Lachnospiraceae	unclassified_F_Lachnospiraceae	unclassified_F_Lachnospiraceae	
SV75	K_Bacteria	P_Firmicutes	C_Clostridia	O_Lachnospirales	F_Lachnospiraceae	G_Lachnospiraceae NK4A136 group	unclassified_G_Lachnospiraceae NK4A136 group	
SV76	K_Bacteria	P_Firmicutes	C_Clostridia	O_Lachnospirales	F_Lachnospiraceae	G_Lachnospiraceae NK4A136 group	unclassified_G_Lachnospiraceae NK4A136 group	
SV77	K_Bacteria	P_Bacteroidota	C_Bacteroidia	O_Bacteroidales	F_Bacteroidaceae	G_Bacteroides	unclassified_G_Bacteroides	
SV78	K_Bacteria	P_Firmicutes	C_Clostridia	O_Lachnospirales	F_Lachnospiraceae	G_Lachnospiraceae NK4A136 group	unclassified_G_Lachnospiraceae NK4A136 group	
SV79	K_Bacteria	P_Bacteroidota	C_Bacteroidia	O_Bacteroidales	F_Rikenellaceae	G_Alistipes	unclassified_G_Alistipes	
SV80	K_Bacteria	P_Firmicutes	C_Clostridia	O_Lachnospirales	F_Lachnospiraceae	G_[Eubacterium] ruminantium group	unclassified_G_[Eubacterium] ruminantium group	
SV81	K_Bacteria	P_Bacteroidota	C_Bacteroidia	O_Bacteroidales	F_Muribaculaceae	unclassified_F_Muribaculaceae	unclassified_F_Muribaculaceae	
SV82	K_Bacteria	P_Bacteroidota	C_Bacteroidia	O_Bacteroidales	F_Muribaculaceae	unclassified_F_Muribaculaceae	unclassified_F_Muribaculaceae	
SV83	K_Bacteria	P_Bacteroidota	C_Bacteroidia	O_Bacteroidales	F_Muribaculaceae	G_Muribaculum	S_intestinale	
SV84	K_Bacteria	P_Firmicutes	C_Clostridia	O_Peptostreptococcales-Tissierellales	F_Peptostreptococcaceae	G_Romboutsia	S_ilealis	
SV85	K_Bacteria	P_Firmicutes	C_Clostridia	O_Oscillospirales	F_Ruminococcaceae	G_Ruminococcus	unclassified_G_Ruminococcus	
SV86	K_Bacteria	P_Bacteroidota	C_Bacteroidia	O_Bacteroidales	F_Muribaculaceae	unclassified_F_Muribaculaceae	unclassified_F_Muribaculaceae	
SV87	K_Bacteria	P_Firmicutes	C_Clostridia	O_Lachnospirales	F_Lachnospiraceae	G_Marvinbryantia	unclassified_G_Marvinbryantia	
SV88	K_Bacteria	P_Firmicutes	C_Clostridia	O_Oscillospirales	F_Ruminococcaceae	G_Ruminococcus	unclassified_G_Ruminococcus	
SV89	K_Bacteria	P_Firmicutes	C_Bacilli	O_Lactobacillales	F_Lactobacillaceae	G_Lacticaseibacillus	unclassified_G_Lacticaseibacillus	
SV90	K_Bacteria	P_Firmicutes	C_Clostridia	O_Lachnospirales	F_Lachnospiraceae	unclassified_F_Lachnospiraceae	unclassified_F_Lachnospiraceae	
SV91	K_Bacteria	P_Firmicutes	C_Clostridia	O_Lachnospirales	F_Lachnospiraceae	unclassified_F_Lachnospiraceae	unclassified_F_Lachnospiraceae	
SV92	K_Bacteria	P_Firmicutes	C_Bacilli	O_Lactobacillales	F_Lactobacillaceae	G_Ligilactobacillus	unclassified_G_Ligilactobacillus	
SV93	K_Bacteria	P_Firmicutes	C_Clostridia	O_Peptostreptococcales-Tissierellales	F_Peptostreptococcaceae	G_Romboutsia	S_ilealis	
SV94	K_Bacteria	P_Firmicutes	C_Clostridia	O_Oscillospirales	F_Ruminococcaceae	G_Ruminococcus	S_bromii	
SV95	K_Bacteria	P_Firmicutes	C_Clostridia	O_Lachnospirales	F_Lachnospiraceae	unclassified_F_Lachnospiraceae	unclassified_F_Lachnospiraceae	
SV96	K_Bacteria	P_Firmicutes	C_Clostridia	O_Lachnospirales	F_Lachnospiraceae	G_Roseburia	unclassified_G_Roseburia	
SV97	K_Bacteria	P_Firmicutes	C_Clostridia	O_Peptostreptococcales-Tissierellales	F_Peptostreptococcaceae	G_Romboutsia	unclassified_G_Romboutsia	
SV98	K_Bacteria	P_Firmicutes	C_Clostridia	O_Peptostreptococcales-Tissierellales	F_Peptostreptococcaceae	G_Romboutsia	unclassified_G_Romboutsia	
SV99	K_Bacteria	P_Proteobacteria	C_Alphaproteobacteria	O_Rhodospirillales	unclassified_O_Rhodospirillales	unclassified_O_Rhodospirillales	unclassified_O_Rhodospirillales	
SV100	K_Bacteria	P_Firmicutes	C_Clostridia	O_Lachnospirales	F_Lachnospiraceae	G_Roseburia	unclassified_G_Roseburia	
SV101	K_Bacteria	P_Proteobacteria	C_Gammaproteobacteria	O_Burkholderiales	F_Sutterellaceae	G_Parasutterella	unclassified_G_Parasutterella	
SV102	K_Bacteria	P_Bacteroidota	C_Bacteroidia	O_Bacteroidales	F_Muribaculaceae	unclassified_F_Muribaculaceae	unclassified_F_Muribaculaceae	
SV103	K_Bacteria	P_Firmicutes	C_Clostridia	O_Lachnospirales	F_Lachnospiraceae	G_Lachnoclostridium	unclassified_G_Lachnoclostridium	
SV104	K_Bacteria	P_Bacteroidota	C_Bacteroidia	O_Bacteroidales	F_Muribaculaceae	unclassified_F_Muribaculaceae	unclassified_F_Muribaculaceae	
SV105	K_Bacteria	P_Bacteroidota	C_Bacteroidia	O_Bacteroidales	F_Prevotellaceae	G_Prevotellaceae UCG-001	unclassified_G_Prevotellaceae UCG-001	
SV106	K_Bacteria	P_Firmicutes	C_Clostridia	O_Lachnospirales	F_Lachnospiraceae	G_Lachnospiraceae UCG-006	unclassified_G_Lachnospiraceae UCG-006	
SV107	K_Bacteria	P_Firmicutes	C_Clostridia	O_Lachnospirales	F_Lachnospiraceae	G_Lachnospiraceae NK4A136 group	unclassified_G_Lachnospiraceae NK4A136 group	
SV108	K_Bacteria	P_Firmicutes	C_Clostridia	O_Oscillospirales	F_Oscillospiraceae	unclassified_F_Oscillospiraceae	unclassified_F_Oscillospiraceae	
SV109	K_Bacteria	P_Firmicutes	C_Clostridia	O_Lachnospirales	F_Lachnospiraceae	G_Marvinbryantia	unclassified_G_Marvinbryantia	
SV110	K_Bacteria	P_Firmicutes	C_Clostridia	O_Lachnospirales	F_Lachnospiraceae	unclassified_F_Lachnospiraceae	unclassified_F_Lachnospiraceae	
SV111	K_Bacteria	P_Firmicutes	C_Clostridia	O_Lachnospirales	F_Lachnospiraceae	G_Lachnospiraceae NK4A136 group	unclassified_G_Lachnospiraceae NK4A136 group	
SV112	K_Bacteria	P_Firmicutes	C_Clostridia	O_Lachnospirales	F_Lachnospiraceae	unclassified_F_Lachnospiraceae	unclassified_F_Lachnospiraceae	
SV113	K_Bacteria	P_Firmicutes	C_Clostridia	O_Lachnospirales	F_Lachnospiraceae	unclassified_F_Lachnospiraceae	unclassified_F_Lachnospiraceae	
SV114	K_Bacteria	P_Firmicutes	C_Bacilli	O_Lactobacillales	F_Lactobacillaceae	G_Ligilactobacillus	unclassified_G_Ligilactobacillus	
SV115	K_Bacteria	P_Firmicutes	C_Clostridia	O_Clostridia UCG-014	unclassified_O_Clostridia UCG-014	unclassified_O_Clostridia UCG-014	unclassified_O_Clostridia UCG-014	
SV116	K_Bacteria	P_Bacteroidota	C_Bacteroidia	O_Bacteroidales	F_Bacteroidaceae	G_Bacteroides	unclassified_G_Bacteroides	
SV117	K_Bacteria	P_Firmicutes	C_Clostridia	O_Lachnospirales	F_Lachnospiraceae	unclassified_F_Lachnospiraceae	unclassified_F_Lachnospiraceae	
SV118	K_Bacteria	P_Firmicutes	C_Clostridia	O_Lachnospirales	F_Lachnospiraceae	G_Roseburia	unclassified_G_Roseburia	
SV119	K_Bacteria	P_Firmicutes	C_Clostridia	O_Oscillospirales	F_Ruminococcaceae	G_Ruminococcus	unclassified_G_Ruminococcus	
SV120	K_Bacteria	P_Firmicutes	C_Clostridia	O_Peptostreptococcales-Tissierellales	F_Anaerovoracaceae	G_[Eubacterium] nodatum group	unclassified_G_[Eubacterium] nodatum group	
SV121	K_Bacteria	P_Firmicutes	C_Clostridia	O_Lachnospirales	F_Lachnospiraceae	unclassified_F_Lachnospiraceae	unclassified_F_Lachnospiraceae	
SV122	K_Bacteria	P_Firmicutes	C_Clostridia	O_Lachnospirales	F_Lachnospiraceae	G_Roseburia	unclassified_G_Roseburia	
SV123	K_Bacteria	P_Firmicutes	C_Clostridia	O_Lachnospirales	F_Lachnospiraceae	unclassified_F_Lachnospiraceae	unclassified_F_Lachnospiraceae	
SV124	K_Bacteria	P_Firmicutes	C_Clostridia	O_Clostridiales	F_Clostridiaceae	G_Clostridium sensu stricto 1	unclassified_G_Clostridium sensu stricto 1	
SV125	K_Bacteria	P_Firmicutes	C_Clostridia	O_Oscillospirales	F_Ruminococcaceae	G_Ruminococcus	unclassified_G_Ruminococcus	
SV126	K_Bacteria	P_Firmicutes	C_Bacilli	O_Erysipelotrichales	F_Erysipelatoclostridiaceae	G_Erysipelatoclostridium	unclassified_G_Erysipelatoclostridium	
SV127	K_Bacteria	P_Firmicutes	C_Clostridia	O_Lachnospirales	F_Lachnospiraceae	G_Lachnospiraceae NK4A136 group	unclassified_G_Lachnospiraceae NK4A136 group	
SV128	K_Bacteria	P_Firmicutes	C_Clostridia	O_Lachnospirales	F_Lachnospiraceae	G_Marvinbryantia	unclassified_G_Marvinbryantia	
SV129	K_Bacteria	P_Firmicutes	C_Clostridia	O_Oscillospirales	F_[Eubacterium] coprostanoligenes group	unclassified_F_[Eubacterium] coprostanoligenes grou	unclassified_F_[Eubacterium] coprostanoligenes group	
SV130	K_Bacteria	P_Bacteroidota	C_Bacteroidia	O_Bacteroidales	F_Muribaculaceae	unclassified_F_Muribaculaceae	unclassified_F_Muribaculaceae	
SV131	K_Bacteria	P_Firmicutes	C_Clostridia	O_Peptostreptococcales-Tissierellales	F_Peptostreptococcaceae	G_Romboutsia	unclassified_G_Romboutsia	
SV132	K_Bacteria	P_Firmicutes	C_Bacilli	O_Lactobacillales	F_Streptococcaceae	G_Lactococcus	unclassified_G_Lactococcus	
SV133	K_Bacteria	P_Firmicutes	C_Clostridia	O_Lachnospirales	F_Lachnospiraceae	G_Marvinbryantia	unclassified_G_Marvinbryantia	
SV134	K_Bacteria	P_Bacteroidota	C_Bacteroidia	O_Bacteroidales	F_Muribaculaceae	unclassified_F_Muribaculaceae	unclassified_F_Muribaculaceae	
SV135	K_Bacteria	P_Firmicutes	C_Clostridia	O_Lachnospirales	F_Lachnospiraceae	G_A2	unclassified_G_A2	
SV136	K_Bacteria	P_Firmicutes	C_Clostridia	O_Lachnospirales	F_Lachnospiraceae	G_Lachnospiraceae AC2044 group	unclassified_G_Lachnospiraceae AC2044 group	

SV137	K Bacteria	P Firmicutes	C Clostridia	O Peptostreptococcales-Tissierellales	F Peptostreptococcaceae	G Romboutsia	unclassified G Romboutsia
SV138	K Bacteria	P Firmicutes	C Clostridia	O Lachnospirales	F Lachnospiraceae	G Lachnospiraceae NK4A136 group	unclassified G Lachnospiraceae NK4A136 group
SV139	K Bacteria	P Firmicutes	C Bacilli	O Lactobacillales	F Lactobacillaceae	G Lactobacillus	unclassified G Lactobacillus
SV140	K Bacteria	P Firmicutes	C Clostridia	O Lachnospirales	F Lachnospiraceae	G Lachnospiraceae NK4A136 group	unclassified G Lachnospiraceae NK4A136 group
SV141	K Bacteria	P Firmicutes	C Clostridia	O Lachnospirales	F Lachnospiraceae	G Lachnoclostridium	unclassified G Lachnoclostridium
SV142	K Bacteria	P Firmicutes	C Clostridia	O Lachnospirales	F Lachnospiraceae	G Lachnospiraceae NK4A136 group	unclassified G Lachnospiraceae NK4A136 group
SV143	K Bacteria	P Bacteroidota	C Bacteroidia	O Bacteroidales	F Muribaculaceae	unclassified F Muribaculaceae	unclassified F Muribaculaceae
SV144	K Bacteria	P Firmicutes	C Clostridia	O Peptostreptococcales-Tissierellales	F Peptostreptococcaceae	G Romboutsia	S ilealis
SV145	K Bacteria	P Firmicutes	C Clostridia	O Lachnospirales	F Lachnospiraceae	G Lachnospiraceae UCG-001	unclassified G Lachnospiraceae UCG-001
SV146	K Bacteria	P Bacteroidota	C Bacteroidia	O Bacteroidales	F Muribaculaceae	unclassified F Muribaculaceae	unclassified F Muribaculaceae
SV147	K Bacteria	P Firmicutes	C Clostridia	O Lachnospirales	F Lachnospiraceae	unclassified F Lachnospiraceae	unclassified F Lachnospiraceae
SV148	K Bacteria	P Firmicutes	C Clostridia	O Oscillospirales	F Ruminococcaceae	unclassified F Ruminococcaceae	unclassified F Ruminococcaceae
SV149	K Bacteria	P Firmicutes	C Clostridia	O Lachnospirales	F Lachnospiraceae	G Lachnospiraceae AC2044 group	unclassified G Lachnospiraceae AC2044 group
SV150	K Bacteria	P Firmicutes	C Clostridia	O Lachnospirales	F Lachnospiraceae	G Roseburia	unclassified G Roseburia
SV151	K Bacteria	P Firmicutes	C Clostridia	O Lachnospirales	F Lachnospiraceae	G Marvinbryantia	unclassified G Marvinbryantia
SV152	K Bacteria	P Bacteroidota	C Bacteroidia	O Bacteroidales	F Muribaculaceae	unclassified F Muribaculaceae	unclassified F Muribaculaceae
SV153	K Bacteria	P Firmicutes	C Clostridia	O Lachnospirales	F Lachnospiraceae	unclassified F Lachnospiraceae	unclassified F Lachnospiraceae
SV154	K Bacteria	P Firmicutes	C Clostridia	O Clostridia UCG-014	unclassified O Clostridia UCG-014	unclassified O Clostridia UCG-014	unclassified O Clostridia UCG-014
SV155	K Bacteria	P Firmicutes	C Clostridia	O Oscillospirales	F Oscillospiraceae	G UCG-003	unclassified G UCG-003
SV156	K Bacteria	P Bacteroidota	C Bacteroidia	O Bacteroidales	F Muribaculaceae	unclassified F Muribaculaceae	unclassified F Muribaculaceae
SV157	K Bacteria	P Firmicutes	C Clostridia	O Lachnospirales	F Lachnospiraceae	G Lachnoclostridium	unclassified G Lachnoclostridium
SV158	K Bacteria	P Bacteroidota	C Bacteroidia	O Bacteroidales	F Muribaculaceae	unclassified F Muribaculaceae	unclassified F Muribaculaceae
SV159	K Bacteria	P Firmicutes	C Clostridia	O Lachnospirales	F Lachnospiraceae	G Roseburia	unclassified G Roseburia
SV160	K Bacteria	P Firmicutes	C Clostridia	O Lachnospirales	F Lachnospiraceae	G Lachnospiraceae NK4A136 group	unclassified G Lachnospiraceae NK4A136 group
SV161	K Bacteria	P Firmicutes	C Clostridia	O Peptostreptococcales-Tissierellales	F Anaerovoracaceae	G [Eubacterium] nodatum group	unclassified G [Eubacterium] nodatum group
SV162	K Bacteria	P Firmicutes	C Clostridia	O Lachnospirales	F Lachnospiraceae	G Lachnoclostridium	unclassified G Lachnoclostridium
SV163	K Bacteria	P Firmicutes	C Clostridia	O Lachnospirales	F Lachnospiraceae	G Blautia	unclassified G Blautia
SV164	K Bacteria	P Firmicutes	C Clostridia	O Lachnospirales	F Lachnospiraceae	G Lachnospiraceae UCG-001	unclassified G Lachnospiraceae UCG-001
SV165	K Bacteria	P Bacteroidota	C Bacteroidia	O Bacteroidales	F Rikenellaceae	G Alistipes	unclassified G Alistipes
SV166	K Bacteria	P Firmicutes	C Clostridia	O Lachnospirales	F Lachnospiraceae	G Lachnospiraceae NK4A136 group	unclassified G Lachnospiraceae NK4A136 group
SV167	K Bacteria	P Firmicutes	C Clostridia	O Lachnospirales	F Lachnospiraceae	G Marvinbryantia	unclassified G Marvinbryantia
SV168	K Bacteria	P Firmicutes	C Clostridia	O Lachnospirales	F Lachnospiraceae	G A2	unclassified G A2
SV169	K Bacteria	P Firmicutes	C Clostridia	O Oscillospirales	F Ruminococcaceae	G Incertae Sedis	unclassified G Incertae Sedis
SV170	K Bacteria	P Firmicutes	C Clostridia	O Lachnospirales	F Lachnospiraceae	G Lachnospiraceae UCG-001	unclassified G Lachnospiraceae UCG-001
SV171	K Bacteria	P Firmicutes	C Clostridia	O Lachnospirales	F Lachnospiraceae	G Marvinbryantia	unclassified G Marvinbryantia
SV172	K Bacteria	P Firmicutes	C Clostridia	O Lachnospirales	F Lachnospiraceae	G [Eubacterium] ruminantium group	unclassified G [Eubacterium] ruminantium group
SV173	K Bacteria	P Firmicutes	C Clostridia	O Clostridia UCG-014	unclassified O Clostridia UCG-014	unclassified O Clostridia UCG-014	unclassified O Clostridia UCG-014
SV174	K Bacteria	P Bacteroidota	C Bacteroidia	O Bacteroidales	F Muribaculaceae	unclassified F Muribaculaceae	unclassified F Muribaculaceae
SV175	K Bacteria	P Firmicutes	C Clostridia	O Lachnospirales	F Lachnospiraceae	G Lachnospiraceae NK4A136 group	unclassified G Lachnospiraceae NK4A136 group
SV176	K Bacteria	P Firmicutes	C Bacilli	O Lactobacillales	F Lactobacillaceae	G Limosilactobacillus	unclassified G Limosilactobacillus
SV177	K Bacteria	P Firmicutes	C Clostridia	O Lachnospirales	F Lachnospiraceae	G Roseburia	unclassified G Roseburia
SV178	K Bacteria	P Proteobacteria	C Gammaproteobacteria	O Enterobacterales	F Enterobacteriaceae	G Escherichia-Shigella	unclassified G Escherichia-Shigella
SV179	K Bacteria	P Firmicutes	C Clostridia	O Clostridiales	F Clostridiaceae	G Clostridium sensu stricto 1	unclassified G Clostridium sensu stricto 1
SV180	K Bacteria	P Cyanobacteria	C Vampirivibrionia	O Gastranaerophilales	unclassified O Gastranaerophilales	unclassified O Gastranaerophilales	unclassified O Gastranaerophilales
SV181	K Bacteria	P Firmicutes	C Clostridia	O Lachnospirales	F Lachnospiraceae	G Blautia	unclassified G Blautia
SV182	K Bacteria	P Firmicutes	C Clostridia	O Oscillospirales	F Ruminococcaceae	G Anaerotruncus	unclassified G Anaerotruncus
SV183	K_Bacteria	P_Bacteroidota	C_Bacteroidia	O_Bacteroidales	F_Muribaculaceae	unclassified_F_Muribaculaceae	unclassified_F_Muribaculaceae
SV184	K Bacteria	P Firmicutes	C Clostridia	O Lachnospirales	F Lachnospiraceae	G Lachnospiraceae UCG-001	unclassified G Lachnospiraceae UCG-001
SV185	K Bacteria	P Firmicutes	C Clostridia	O Lachnospirales	F Lachnospiraceae	G Lachnospiraceae NK4A136 group	unclassified G Lachnospiraceae NK4A136 group
SV186	K Bacteria	P Firmicutes	C Clostridia	O Oscillospirales	F UCG-010	unclassified F UCG-010	unclassified F UCG-010
SV187	K Bacteria	P Firmicutes	C Clostridia	O Lachnospirales	F Lachnospiraceae	unclassified F Lachnospiraceae	unclassified F Lachnospiraceae
SV188	K Bacteria	P Firmicutes	C Clostridia	O Lachnospirales	F Lachnospiraceae	G [Eubacterium] xylanophilum group	unclassified G [Eubacterium] xylanophilum group
SV189	K_Bacteria	P_Firmicutes	C_Clostridia	O_Lachnospirales	F_Lachnospiraceae	unclassified_F_Lachnospiraceae	unclassified_F_Lachnospiraceae
SV190	K Bacteria	P Firmicutes	C Clostridia	O Lachnospirales	F Lachnospiraceae	unclassified F Lachnospiraceae	unclassified F Lachnospiraceae
SV191	K Bacteria	P Cyanobacteria	C Vampirivibrionia	O Gastranaerophilales	unclassified O Gastranaerophilales	unclassified O Gastranaerophilales	unclassified O Gastranaerophilales
SV192	K Bacteria	P Firmicutes	C Clostridia	O Lachnospirales	F Lachnospiraceae	G Lachnospiraceae NK4A136 group	unclassified G Lachnospiraceae NK4A136 group
SV193	K Bacteria	P Bacteroidota	C Bacteroidia	O Bacteroidales	F Muribaculaceae	unclassified F Muribaculaceae	unclassified F Muribaculaceae
SV194	K Bacteria	P Firmicutes	C Clostridia	O Peptostreptococcales-Tissierellales	F Peptostreptococcaceae	G Romboutsia	S ilealis
SV195	K_Bacteria	P_Firmicutes	C_Clostridia	O_Lachnospirales	F_Lachnospiraceae	G_Roseburia	unclassified_G_Roseburia
SV196	K Bacteria	P Firmicutes	C Clostridia	O Oscillospirales	F Oscillospiraceae	G Oscillibacter	unclassified G Oscillibacter
SV197	K Bacteria	P Firmicutes	C Clostridia	O Lachnospirales	F Lachnospiraceae	G Tyzzerella	unclassified G Tyzzerella
SV198	K Bacteria	P Firmicutes	C Clostridia	O Lachnospirales	F Lachnospiraceae	G Lachnospiraceae NK4A136 group	unclassified G Lachnospiraceae NK4A136 group
SV199	K Bacteria	P Firmicutes	C Clostridia	O Lachnospirales	F Lachnospiraceae	unclassified F Lachnospiraceae	unclassified F Lachnospiraceae
SV200	K Bacteria	P Firmicutes	C Clostridia	O Clostridia UCG-014	unclassified O Clostridia UCG-014	unclassified O Clostridia UCG-014	unclassified O Clostridia UCG-014
SV201	K_Bacteria	P_Firmicutes	C_Clostridia	O_Lachnospirales	F_Lachnospiraceae	G_Lachnospiraceae UCG-001	unclassified_G_Lachnospiraceae UCG-001
SV202	K Bacteria	P Firmicutes	C Clostridia	O Lachnospirales	F Defluviitaleaceae	G Defluviitaleaceae UCG-011	unclassified G Defluviitaleaceae UCG-011
SV203	K Bacteria	P Firmicutes	C Bacilli	O Lactobacillales	F Lactobacillaceae	G Ligilactobacillus	unclassified G Ligilactobacillus
SV204	K Bacteria	P Firmicutes	C Clostridia	O Clostridiales	F Clostridiaceae	G Clostridium sensu stricto 1	unclassified G Clostridium sensu stricto 1
SV205	K Bacteria	P Firmicutes	C Clostridia	O Lachnospirales	F Lachnospiraceae	unclassified F Lachnospiraceae	unclassified F Lachnospiraceae
SV206	K Bacteria	P Bacteroidota	C Bacteroidia	O Bacteroidales	F Muribaculaceae	unclassified F Muribaculaceae	unclassified F Muribaculaceae
SV207	K_Bacteria	P_Firmicutes	C_Clostridia	O_Lachnospirales	F_Lachnospiraceae	G_Marvinbryantia	unclassified_G_Marvinbryantia
SV208	K Bacteria	P Firmicutes	C Clostridia	O Lachnospirales	F Lachnospiraceae	G Lachnoclostridium	unclassified G Lachnoclostridium
SV209	K Bacteria	P Firmicutes	C Clostridia	O Oscillospirales	F Oscillospiraceae	unclassified F Oscillospiraceae	unclassified F Oscillospiraceae
SV210	K Bacteria	P Firmicutes	C Clostridia	O Clostridia UCG-014	unclassified O Clostridia UCG-014	unclassified O Clostridia UCG-014	unclassified O Clostridia UCG-014
SV211	K Bacteria	P Firmicutes	C Clostridia	O Lachnospirales	F Lachnospiraceae	G Lachnospiraceae NK4A136 group	unclassified G Lachnospiraceae NK4A136 group
SV212	K Bacteria	P Firmicutes	C Bacilli	O Erysipelotrichales	F Erysipelotrichaceae	unclassified F Erysipelotrichaceae	unclassified F Erysipelotrichaceae
SV213	K Bacteria	P Firmicutes	C Bacilli	O Lactobacillales	F Lactobacillaceae	G Lacticaseibacillus	unclassified G Lacticaseibacillus
SV214	K Bacteria	P Firmicutes	C Clostridia	O Lachnospirales	F Lachnospiraceae	G Roseburia	unclassified G Roseburia
SV215	K Bacteria	P Bacteroidota	C Bacteroidia	O Bacteroidales	F Muribaculaceae	unclassified F Muribaculaceae	unclassified F Muribaculaceae
SV216	K Bacteria	P Firmicutes	C Clostridia	O Peptostreptococcales-Tissierellales	F Peptostreptococcaceae	G Romboutsia	unclassified G Romboutsia
SV217	K Bacteria	P Firmicutes	C Bacilli	O Lactobacillales	F Lactobacillaceae	G Ligilactobacillus	unclassified G Ligilactobacillus
SV218	K Bacteria	P Firmicutes	C Clostridia	O Peptostreptococcales-Tissierellales	F Peptostreptococcaceae	G Romboutsia	S ilealis
SV219	K Bacteria	P Bacteroidota	C Bacteroidia	O Bacteroidales	F Muribaculaceae	unclassified F Muribaculaceae	unclassified F Muribaculaceae
SV220	K Bacteria	P Firmicutes	C Clostridia	O Clostridia UCG-014	unclassified O Clostridia UCG-014	unclassified O Clostridia UCG-014	unclassified O Clostridia UCG-014
SV221	K Bacteria	P Firmicutes	C Clostridia	O Clostridia UCG-014	unclassified O Clostridia UCG-014	unclassified O Clostridia UCG-014	unclassified O Clostridia UCG-014
SV222	K Bacteria	P Firmicutes	C Clostridia	O Lachnospirales	F Lachnospiraceae	G Marvinbryantia	unclassified G Marvinbryantia
SV223	K Bacteria	P Firmicutes	C Clostridia	O Lachnospirales	F Lachnospiraceae	G Tyzzerella	unclassified G Tyzzerella
SV224	K Bacteria	P Firmicutes	C Clostridia	O Peptococcales	F Peptococcaceae	unclassified F Peptococcaceae	unclassified F Peptococcaceae
SV225	K Bacteria	P Firmicutes	C Clostridia	O Oscillospirales	F Ruminococcaceae	G Ruminococcus	unclassified G Ruminococcus
SV226	K Bacteria	P Firmicutes	C Clostridia	O Clostridia UCG-014	unclassified O Clostridia UCG-014	unclassified O Clostridia UCG-014	unclassified O Clostridia UCG-014
SV227	K Bacteria	P Firmicutes	C Clostridia	O Lachnospirales	F Lachnospiraceae	G A2	unclassified G A2
SV228	K Bacteria	P Bacteroidota	C Bacteroidia	O Bacteroidales	F Muribaculaceae	unclassified F Muribaculaceae	unclassified F Muribaculaceae
SV229	K Bacteria	P Firmicutes	C Clostridia	O Lachnospirales	F Lachnospiraceae	G Roseburia	unclassified G Roseburia
SV230	K Bacteria	P Firmicutes	C Clostridia	O Clostridia UCG-014	unclassified O Clostridia UCG-014	unclassified O Clostridia UCG-014	unclassified O Clostridia UCG-014
SV231	K Bacteria	P Actinobacteriota	C Actinobacteria	O Micrococcales	F Micrococcaceae	G Rothia	unclassified G Rothia
SV232	K Bacteria	P Firmicutes	C Clostridia	O Lachnospirales	F Lachnospiraceae	G Roseburia	unclassified G Roseburia
SV233	K Bacteria	P Firmicutes	C Clostridia	O Lachnospirales	F Lachnospiraceae	G Marvinbryantia	unclassified G Marvinbryantia
SV234	K Bacteria	P Firmicutes	C Clostridia	O Lachnospirales	F Lachnospiraceae	unclassified F Lachnospiraceae	unclassified F Lachnospiraceae
SV235	K Bacteria	P Firmicutes	C Clostridia	O Lachnospirales	F Lachnospiraceae	G Lachnospiraceae NK4A136 group	unclassified G Lachnospiraceae NK4A136 group
SV236	K Bacteria	P Firmicutes	C Clostridia	O Lachnospirales	F Lachnospiraceae	G Marvinbryantia	unclassified G Marvinbryantia
SV237	K Bacteria	P Firmicutes	C Clostridia	O Lachnospirales	F Lachnospiraceae	G Lachnospiraceae UCG-001	unclassified G Lachnospiraceae UCG-001
SV238	K Bacteria	P Firmicutes	C Bacilli	O Lactobacillales	F Lactobacillaceae	G Lactobacillus	unclassified G Lactobacillus
SV239	K Bacteria	P Firmicutes	C Clostridia	O Lachnospirales	F Lachnospiraceae	unclassified F Lachnospiraceae	unclassified F Lachnospiraceae
SV240	K Bacteria	P Firmicutes	C Clostridia	O Lachnospirales	F Lachnospiraceae	G Lachnospiraceae NK4A136 group	unclassified G Lachnospiraceae NK4A136 group
SV241	K Bacteria	P Firmicutes	C Clostridia	O Oscillospirales	F Oscillospiraceae	unclassified F Oscillospiraceae	unclassified F Oscillospiraceae
SV242	K Bacteria	P Firmicutes	C Clostridia	O Lachnospirales	F Lachnospiraceae	unclassified F Lachnospiraceae	unclassified F Lachnospiraceae
SV243	K Bacteria	P Firmicutes	C Clostridia	O Clostridia UCG-014	unclassified O Clostridia UCG-014	unclassified O Clostridia UCG-014	unclassified O Clostridia UCG-014
SV244	K Bacteria	P Firmicutes	C Clostridia	O Peptostreptococcales-Tissierellales	F Anaerovoracaceae	G [Eubacterium] brachy group	unclassified G [Eubacterium] brachy group
SV245	K Bacteria	P Cyanobacteria	C Vampirivibrionia	O Gastranaerophilales	unclassified O Gastranaerophilales	unclassified O Gastranaerophilales	unclassified O Gastranaerophilales
SV246	K Bacteria	P Firmicutes	C Clostridia	O Lachnospirales	F Lachnospiraceae	unclassified F Lachnospiraceae	unclassified F Lachnospiraceae
SV247	K Bacteria	P Firmicutes	C Clostridia	O Clostridia UCG-014	unclassified O Clostridia UCG-014	unclassified O Clostridia UCG-014	unclassified O Clostridia UCG-014
SV248	K_Bacteria	P_Firmicutes	C_Clostridia	O_Lachnospirales	F_Lachnospiraceae	unclassified_F_Lachnospiraceae	unclassified_F_Lachnospiraceae
SV249	K Bacteria	P Firmicutes	C Clostridia	O Oscillospirales	F Oscillospiraceae	unclassified F Oscillospiraceae	unclassified F Oscillospiraceae
SV250	K Bacteria	P Firmicutes	C Clostridia	O Oscillospirales	F Ruminococcaceae	G Ruminococcus	unclassified G Ruminococcus
SV251	K Bacteria	P Firmicutes	C Bacilli	O Bacillales	F Bacillaceae	G Bacillus	unclassified G Bacillus
SV252	K Bacteria	P Bacteroidota	C Bacteroidia	O Bacteroidales	F Rikenellaceae	G Alistipes	unclassified G Alistipes
SV253	K Bacteria	P Firmicutes	C Clostridia	O Lachnospirales	F Lachnospiraceae	unclassified F Lachnospiraceae	unclassified F Lachnospiraceae
SV254	K_Bacteria	P_Firmicutes	C_Clostridia	O_Peptostreptococcales-Tissierellales	F_Peptostreptococcaceae	G_Romboutsia	unclassified_G_Romboutsia
SV255	K Bacteria	P Firmicutes	C Clostridia	O Lachnospirales	F Lachnospiraceae	G Lachnospiraceae NK4A136 group	unclassified G Lachnospiraceae NK4A136 group
SV256	K Bacteria	P Firmicutes	C Clostridia	O Lachnospirales	F Lachnospiraceae	G Lachnospiraceae NK4A136 group	unclassified G Lachnospiraceae NK4A136 group
SV257	K Bacteria	P Bacteroidota	C Bacteroidia	O Bacteroidales	F Muribaculaceae	unclassified F Muribaculaceae	unclassified F Muribaculaceae
SV258	K Bacteria	P Firmicutes	C Clostridia	O Lachnospirales	F Lachnospiraceae	unclassified F Lachnospiraceae	unclassified F Lachnospiraceae
SV259	K Bacteria	P Bacteroidota	C Bacteroidia	O Bacteroidales	F Muribaculaceae	unclassified F Muribaculaceae	unclassified F Muribaculaceae
SV260	K_Bacteria	P_Firmicutes	C_Clostridia	O_Lachnospirales	F_Lachnospiraceae	G_GCA-900066575	unclassified_G_GCA-900066575
SV261	K Bacteria	P Firmicutes	C Clostridia	O Lachnospirales	F Lachnospiraceae	unclassified F Lachnospiraceae	unclassified F Lachnospiraceae
SV262	K Bacteria	P Bacteroidota	C Bacteroidia	O Bacteroidales	F Muribaculaceae	unclassified F Muribaculaceae	unclassified F Muribaculaceae
SV263	K Bacteria	P Firmicutes	C Clostridia	O Oscillospirales	F Oscillospiraceae	G Oscillibacter	unclassified G Oscillibacter
SV264	K Bacteria	P Firmicutes	C Clostridia	O Lachnospirales	F Lachnospiraceae	G Lachnoclostridium	unclassified G Lachnoclostridium
SV265	K Bacteria	P Firmicutes	C Clostridia	O Clostridia UCG-014	unclassified O Clostridia UCG-014	unclassified O Clostridia UCG-014	unclassified O Clostridia UCG-014
SV266	K_Bacteria	P_Firmicutes	C_Clostridia	O_Lachnospirales	F_Lachnospiraceae	G_[Eubacterium] xylanophilum group	unclassified_G_[Eubacterium] xylanophilum group
SV267	K Bacteria	P Firmicutes	C Clostridia	O Lachnospirales	F Lachnospiraceae	G ASF356	unclassified G ASF356
SV268	K Bacteria	P Firmicutes	C Clostridia	O Oscillospirales	F Oscillospiraceae	G Colidextribacter	unclassified G Colidextribacter
SV269	K Bacteria	P Firmicutes	C Clostridia	O Oscillospirales	F Ruminococcaceae	G Ruminococcus	unclassified G Ruminococcus
SV270	K Bacteria	P Firmicutes	C Clostridia	O Clostridiales	F Clostridiaceae	G Clostridium sensu stricto 1	unclassified G Clostridium sensu stricto 1
SV271	K Bacteria	P Firmicutes	C Clostridia	O Lachnospirales	F Lachnospiraceae	unclassified F Lachnospiraceae	unclassified F Lachnospiraceae
SV272	K Bacteria	P Firmicutes	C Bacilli	O Lactobacillales	F Enterococcaceae	G Enterococcus	unclassified G Enterococcus
SV273	K Bacteria	P Firmicutes	C Clostridia	O Lachnospirales	F Lachnospiraceae	unclassified F Lachnospiraceae	unclassified F Lachnospiraceae
SV274	K Bacteria	P Firmicutes	C Clostridia	O Lachnospirales	F Lachnospiraceae	G Lachnospiraceae UCG-001	unclassified G Lachnospiraceae UCG-001

SV275	K_Bacteria	P_Firmicutes	C_Clostridia	O_Oscillospirales	F_Ruminococcaceae	unclassified_F_Ruminococcaceae	unclassified_F_Ruminococcaceae
SV276	K_Bacteria	P_Firmicutes	C_Clostridia	O_Lachnospirales	F_Lachnospiraceae	G_Lachnospiraceae NK4A136 group	unclassified_G_Lachnospiraceae NK4A136 group
SV277	K_Bacteria	P_Bacteroidota	C_Bacteroidia	O_Bacteroidales	F_Prevotellaceae	G_Prevotellaceae UCG-001	unclassified_G_Prevotellaceae UCG-001
SV278	K_Bacteria	P_Firmicutes	C_Clostridia	O_Lachnospirales	F_Lachnospiraceae	G_Anaerostipes	unclassified_G_Anaerostipes
SV279	K_Bacteria	P_Firmicutes	C_Clostridia	O_Clostridiales	F_Clostridiaceae	G_Clostridium sensu stricto 1	unclassified_G_Clostridium sensu stricto 1
SV280	K_Bacteria	P_Firmicutes	C_Clostridia	O_Lachnospirales	F_Lachnospiraceae	G_A2	unclassified_G_A2
SV281	K_Bacteria	P_Firmicutes	C_Clostridia	O_Lachnospirales	F_Lachnospiraceae	G_Lachnospiraceae NK4A136 group	unclassified_G_Lachnospiraceae NK4A136 group
SV282	K_Bacteria	P_Firmicutes	C_Bacilli	O_Erysipelotrichales	F_Erysipelatoclostridiaceae	G_Erysipelatoclostridium	unclassified_G_Erysipelatoclostridium
SV283	K_Bacteria	P_Firmicutes	C_Bacilli	O_Erysipelotrichales	F_Erysipelotrichaceae	G_Turicibacter	unclassified_G_Turicibacter
SV284	K_Bacteria	P_Firmicutes	C_Clostridia	O_Peptostreptococcales-Tissierellales	F_Anaerovoracaceae	G_Family XIII UCG-001	unclassified_G_Family XIII UCG-001
SV285	K_Bacteria	P_Firmicutes	C_Clostridia	O_Lachnospirales	F_Lachnospiraceae	unclassified_F_Lachnospiraceae	unclassified_F_Lachnospiraceae
SV286	K_Bacteria	P_Firmicutes	C_Bacilli	O_Lactobacillales	F_Lactobacillaceae	G_Lactiplantibacillus	unclassified_G_Lactiplantibacillus
SV287	K_Bacteria	P_Firmicutes	C_Clostridia	O_Lachnospirales	F_Lachnospiraceae	G_Lachnospiraceae NK4A136 group	unclassified_G_Lachnospiraceae NK4A136 group
SV288	K_Bacteria	P_Firmicutes	C_Clostridia	O_Lachnospirales	F_Defluviitaleaceae	G_Defluviitaleaceae UCG-011	unclassified_G_Defluviitaleaceae UCG-011
SV289	K_Bacteria	P_Firmicutes	C_Clostridia	O_Oscillospirales	F_Oscillospiraceae	G_Oscillibacter	unclassified_G_Oscillibacter
SV290	K_Bacteria	P_Bacteroidota	C_Bacteroidia	O_Bacteroidales	F_Muribaculaceae	unclassified_F_Muribaculaceae	unclassified_F_Muribaculaceae
SV291	K_Bacteria	P_Firmicutes	C_Bacilli	O_Erysipelotrichales	F_Erysipelotrichaceae	G_Turicibacter	unclassified_G_Turicibacter
SV292	K_Bacteria	P_Firmicutes	C_Clostridia	O_Lachnospirales	F_Lachnospiraceae	G_Blautia	unclassified_G_Blautia
SV293	K_Bacteria	P_Firmicutes	C_Clostridia	O_Clostridiales	F_Clostridiaceae	G_Clostridium sensu stricto 1	unclassified_G_Clostridium sensu stricto 1
SV294	K_Bacteria	P_Firmicutes	C_Clostridia	O_Clostridia UCG-014	unclassified_O_Clostridia UCG-014	unclassified_O_Clostridia UCG-014	unclassified_O_Clostridia UCG-014
SV295	K_Bacteria	P_Firmicutes	C_Clostridia	O_Lachnospirales	F_Lachnospiraceae	G_Lachnospiraceae UCG-001	unclassified_G_Lachnospiraceae UCG-001
SV296	K_Bacteria	P_Firmicutes	C_Clostridia	O_Clostridia UCG-014	unclassified_O_Clostridia UCG-014	unclassified_O_Clostridia UCG-014	unclassified_O_Clostridia UCG-014
SV297	K_Bacteria	P_Firmicutes	C_Clostridia	O_Lachnospirales	F_Lachnospiraceae	unclassified_F_Lachnospiraceae	unclassified_F_Lachnospiraceae
SV298	K_Bacteria	P_Bacteroidota	C_Bacteroidia	O_Bacteroidales	F_Prevotellaceae	G_Prevotellaceae UCG-001	unclassified_G_Prevotellaceae UCG-001
SV299	K_Bacteria	P_Firmicutes	C_Clostridia	O_Clostridiales	F_Clostridiaceae	G_Clostridium sensu stricto 1	unclassified_G_Clostridium sensu stricto 1
SV300	K_Bacteria	P_Firmicutes	C_Clostridia	O_Oscillospirales	F_Ruminococcaceae	G_Incertae Sedis	unclassified_G_Incertae Sedis
SV301	K_Bacteria	P_Firmicutes	C_Clostridia	O_Oscillospirales	F_Ruminococcaceae	G_Anaerotruncus	unclassified_G_Anaerotruncus
SV302	K_Bacteria	P_Bacteroidota	C_Bacteroidia	O_Bacteroidales	F_Muribaculaceae	unclassified_F_Muribaculaceae	unclassified_F_Muribaculaceae
SV303	K_Bacteria	P_Firmicutes	C_Clostridia	O_Lachnospirales	F_Lachnospiraceae	G_Lachnospiraceae NK4A136 group	unclassified_G_Lachnospiraceae NK4A136 group
SV304	K_Bacteria	P_Firmicutes	C_Clostridia	O_Lachnospirales	F_Lachnospiraceae	G_Lachnospiraceae NK4A136 group	unclassified_G_Lachnospiraceae NK4A136 group
SV305	K_Bacteria	P_Firmicutes	C_Clostridia	O_Peptostreptococcales-Tissierellales	F_Peptostreptococcaceae	G_Romboutsia	unclassified_G_Romboutsia
SV306	K_Bacteria	P_Firmicutes	C_Clostridia	O_Lachnospirales	F_Lachnospiraceae	unclassified_F_Lachnospiraceae	unclassified_F_Lachnospiraceae
SV307	K_Bacteria	P_Firmicutes	C_Clostridia	O_Lachnospirales	F_Lachnospiraceae	unclassified_F_Lachnospiraceae	unclassified_F_Lachnospiraceae
SV308	K_Bacteria	P_Firmicutes	C_Clostridia	O_Clostridiales	F_Clostridiaceae	G_Clostridium sensu stricto 1	unclassified_G_Clostridium sensu stricto 1
SV309	K_Bacteria	P_Bacteroidota	C_Bacteroidia	O_Bacteroidales	F_Muribaculaceae	unclassified_F_Muribaculaceae	unclassified_F_Muribaculaceae
SV310	K_Bacteria	P_Firmicutes	C_Clostridia	O_Lachnospirales	F_Lachnospiraceae	G_Lachnospiraceae NK4A136 group	S_bacterium
SV311	K_Bacteria	P_Firmicutes	C_Clostridia	O_Lachnospirales	F_Lachnospiraceae	G_Lachnospiraceae NK4A136 group	unclassified_G_Lachnospiraceae NK4A136 group
SV312	K_Bacteria	P_Firmicutes	C_Clostridia	O_Lachnospirales	F_Lachnospiraceae	unclassified_F_Lachnospiraceae	unclassified_F_Lachnospiraceae
SV313	K_Bacteria	P_Firmicutes	C_Clostridia	O_Lachnospirales	F_Lachnospiraceae	G_Lachnospiraceae NK4A136 group	unclassified_G_Lachnospiraceae NK4A136 group
SV314	K_Bacteria	P_Firmicutes	C_Clostridia	O_Lachnospirales	F_Lachnospiraceae	G_Lachnospiraceae NK4A136 group	unclassified_G_Lachnospiraceae NK4A136 group
SV315	K_Bacteria	P_Firmicutes	C_Clostridia	O_Lachnospirales	F_Lachnospiraceae	G_Lachnospiraceae NK4A136 group	unclassified_G_Lachnospiraceae NK4A136 group
SV316	K_Bacteria	P_Firmicutes	C_Clostridia	O_Oscillospirales	F_Butyricicoccaceae	G_UCG-009	unclassified_G_UCG-009
SV317	K_Bacteria	P_Firmicutes	C_Clostridia	O_Oscillospirales	F_Oscillospiraceae	unclassified_F_Oscillospiraceae	unclassified_F_Oscillospiraceae
SV318	K_Bacteria	P_Firmicutes	C_Bacilli	O_Staphylococcales	F_Staphylococcaceae	G_Staphylococcus	unclassified_G_Staphylococcus
SV319	K_Bacteria	P_Firmicutes	C_Clostridia	O_Lachnospirales	F_Lachnospiraceae	G_Lachnoclostridium	unclassified_G_Lachnoclostridium
SV320	K_Bacteria	P_Firmicutes	C_Clostridia	O_Oscillospirales	F_Ruminococcaceae	G_Ruminococcus	S_bromii
SV321	K_Bacteria	P_Firmicutes	C_Bacilli	O_Lactobacillales	F_Listeriaceae	G_Listeria	unclassified_G_Listeria
SV322	K_Bacteria	P_Firmicutes	C_Bacilli	O_Lactobacillales	F_Lactobacillaceae	G_Ligilactobacillus	unclassified_G_Ligilactobacillus
SV323	K_Bacteria	P_Firmicutes	C_Clostridia	O_Oscillospirales	F_Oscillospiraceae	G_Colidextribacter	unclassified_G_Colidextribacter
SV324	K_Bacteria	P_Firmicutes	C_Clostridia	O_Lachnospirales	F_Lachnospiraceae	G_[Ruminococcus] gauvreauii group	unclassified_G_[Ruminococcus] gauvreauii group
SV325	K_Bacteria	P_Firmicutes	C_Clostridia	O_Lachnospirales	F_Lachnospiraceae	G_Lachnoclostridium	unclassified_G_Lachnoclostridium
SV326	K_Bacteria	P_Firmicutes	C_Clostridia	O_Lachnospirales	F_Lachnospiraceae	G_Blautia	unclassified_G_Blautia
SV327	K_Bacteria	P_Firmicutes	C_Clostridia	O_Lachnospirales	F_Lachnospiraceae	G_Lachnospiraceae NK4A136 group	unclassified_G_Lachnospiraceae NK4A136 group
SV328	K_Bacteria	P_Firmicutes	C_Clostridia	O_Lachnospirales	F_Lachnospiraceae	unclassified_F_Lachnospiraceae	unclassified_F_Lachnospiraceae
SV329	K_Bacteria	P_Firmicutes	C_Clostridia	O_Clostridia UCG-014	unclassified_O_Clostridia UCG-014	unclassified_O_Clostridia UCG-014	unclassified_O_Clostridia UCG-014
SV330	K_Bacteria	P_Firmicutes	C_Clostridia	O_Lachnospirales	F_Lachnospiraceae	unclassified_F_Lachnospiraceae	unclassified_F_Lachnospiraceae
SV331	K_Bacteria	P_Firmicutes	C_Clostridia	O_Oscillospirales	F_Ruminococcaceae	G_Paludicola	unclassified_G_Paludicola
SV332	K_Bacteria	P_Firmicutes	C_Clostridia	O_Lachnospirales	F_Lachnospiraceae	unclassified_F_Lachnospiraceae	unclassified_F_Lachnospiraceae
SV333	K_Bacteria	P_Firmicutes	C_Clostridia	O_Lachnospirales	F_Lachnospiraceae	G_Lachnospiraceae NK4A136 group	unclassified_G_Lachnospiraceae NK4A136 group
SV334	K_Bacteria	P_Firmicutes	C_Clostridia	O_Lachnospirales	F_Lachnospiraceae	unclassified_F_Lachnospiraceae	unclassified_F_Lachnospiraceae
SV335	K_Bacteria	P_Firmicutes	C_Clostridia	O_Lachnospirales	F_Lachnospiraceae	G_Lachnoclostridium	unclassified_G_Lachnoclostridium
SV336	K_Bacteria	P_Firmicutes	C_Clostridia	O_Lachnospirales	F_Lachnospiraceae	G_Lachnoclostridium	unclassified_G_Lachnoclostridium
SV337	K_Bacteria	P_Firmicutes	C_Clostridia	O_Oscillospirales	F_Butyricicoccaceae	G_UCG-009	unclassified_G_UCG-009
SV338	K_Bacteria	P_Firmicutes	C_Clostridia	O_Lachnospirales	F_Lachnospiraceae	G_ASF356	unclassified_G_ASF356
SV339	K_Bacteria	P_Firmicutes	C_Clostridia	O_Lachnospirales	F_Lachnospiraceae	G_Marvinbryantia	unclassified_G_Marvinbryantia
SV340	K_Bacteria	P_Firmicutes	C_Clostridia	O_Oscillospirales	F_Ruminococcaceae	G_Incertae Sedis	unclassified_G_Incertae Sedis
SV341	K_Bacteria	P_Firmicutes	C_Clostridia	O_Oscillospirales	F_Oscillospiraceae	unclassified_F_Oscillospiraceae	unclassified_F_Oscillospiraceae
SV342	K_Bacteria	P_Firmicutes	C_Clostridia	O_Lachnospirales	F_Lachnospiraceae	G_GCA-900066575	unclassified_G_GCA-900066575
SV343	K_Bacteria	P_Firmicutes	C_Clostridia	O_Lachnospirales	F_Lachnospiraceae	G_Tuzzerella	unclassified_G_Tuzzerella
SV344	K_Bacteria	P_Firmicutes	C_Clostridia	O_Lachnospirales	F_Lachnospiraceae	G_Roseburia	unclassified_G_Roseburia
SV345	K_Bacteria	P_Firmicutes	C_Clostridia	O_Lachnospirales	F_Lachnospiraceae	G_Lachnospiraceae NK4A136 group	unclassified_G_Lachnospiraceae NK4A136 group
SV346	K_Bacteria	P_Firmicutes	C_Clostridia	O_Clostridia UCG-014	unclassified_O_Clostridia UCG-014	unclassified_O_Clostridia UCG-014	unclassified_O_Clostridia UCG-014
SV347	K_Bacteria	P_Firmicutes	C_Bacilli	O_Erysipelotrichales	F_Erysipelotrichaceae	G_Turicibacter	unclassified_G_Turicibacter
SV348	K_Bacteria	P_Firmicutes	C_Clostridia	O_Lachnospirales	F_Lachnospiraceae	unclassified_F_Lachnospiraceae	unclassified_F_Lachnospiraceae
SV349	K_Bacteria	P_Firmicutes	C_Clostridia	O_Lachnospirales	F_Lachnospiraceae	G_Lachnospiraceae NK4A136 group	unclassified_G_Lachnospiraceae NK4A136 group
SV350	K_Bacteria	P_Firmicutes	C_Clostridia	O_Lachnospirales	F_Lachnospiraceae	G_Lachnospiraceae UCG-006	unclassified_G_Lachnospiraceae UCG-006
SV351	K_Bacteria	P_Firmicutes	C_Clostridia	O_Lachnospirales	F_Lachnospiraceae	G_Marvinbryantia	unclassified_G_Marvinbryantia
SV352	K_Bacteria	P_Firmicutes	C_Clostridia	O_Peptostreptococcales-Tissierellales	F_Peptostreptococcaceae	G_Romboutsia	unclassified_G_Romboutsia
SV353	K_Bacteria	P_Actinobacteriota	C_Coriobacteriia	O_Coriobacteriales	F_Eggerthellaceae	G_Enterorhabdus	unclassified_G_Enterorhabdus
SV354	K_Bacteria	P_Proteobacteria	C_Alphaproteobacteria	O_Rhodospirillales	unclassified_O_Rhodospirillales	unclassified_O_Rhodospirillales	unclassified_O_Rhodospirillales
SV355	K_Bacteria	P_Firmicutes	C_Clostridia	O_Oscillospirales	F_Oscillospiraceae	G_Colidextribacter	unclassified_G_Colidextribacter
SV356	K_Bacteria	P_Firmicutes	C_Clostridia	O_Lachnospirales	F_Lachnospiraceae	G_Lachnospiraceae NK4A136 group	unclassified_G_Lachnospiraceae NK4A136 group
SV357	K_Bacteria	P_Firmicutes	C_Clostridia	O_Clostridia UCG-014	unclassified_O_Clostridia UCG-014	unclassified_O_Clostridia UCG-014	unclassified_O_Clostridia UCG-014
SV358	K_Bacteria	P_Firmicutes	C_Clostridia	O_Lachnospirales	F_Lachnospiraceae	G_A2	unclassified_G_A2
SV359	K_Bacteria	P_Bacteroidota	C_Bacteroidia	O_Bacteroidales	F_Muribaculaceae	unclassified_F_Muribaculaceae	unclassified_F_Muribaculaceae
SV360	K_Bacteria	P_Firmicutes	C_Clostridia	O_Peptostreptococcales-Tissierellales	F_Peptostreptococcaceae	G_Romboutsia	unclassified_G_Romboutsia
SV361	K_Bacteria	P_Bacteroidota	C_Bacteroidia	O_Bacteroidales	F_Bacteroidaceae	G_Bacteroides	unclassified_G_Bacteroides
SV362	K_Bacteria	P_Firmicutes	C_Clostridia	O_Oscillospirales	F_Oscillospiraceae	unclassified_F_Oscillospiraceae	unclassified_F_Oscillospiraceae
SV363	K_Bacteria	P_Firmicutes	C_Clostridia	O_Lachnospirales	F_Lachnospiraceae	unclassified_F_Lachnospiraceae	unclassified_F_Lachnospiraceae
SV364	K_Bacteria	P_Firmicutes	C_Clostridia	O_Lachnospirales	F_Lachnospiraceae	G_Marvinbryantia	unclassified_G_Marvinbryantia
SV365	K_Bacteria	P_Firmicutes	C_Clostridia	O_Lachnospirales	F_Lachnospiraceae	unclassified_F_Lachnospiraceae	unclassified_F_Lachnospiraceae
SV366	K_Bacteria	P_Firmicutes	C_Clostridia	O_Lachnospirales	F_Lachnospiraceae	G_Lachnospiraceae NK4A136 group	unclassified_G_Lachnospiraceae NK4A136 group
SV367	K_Bacteria	P_Firmicutes	C_Clostridia	O_Lachnospirales	F_Lachnospiraceae	unclassified_F_Lachnospiraceae	unclassified_F_Lachnospiraceae
SV368	K_Bacteria	P_Firmicutes	C_Clostridia	O_Peptostreptococcales-Tissierellales	F_Peptostreptococcaceae	G_Romboutsia	unclassified_G_Romboutsia
SV369	K_Bacteria	P_Bacteroidota	C_Bacteroidia	O_Bacteroidales	F_Muribaculaceae	unclassified_F_Muribaculaceae	unclassified_F_Muribaculaceae
SV370	K_Bacteria	P_Firmicutes	C_Clostridia	O_Oscillospirales	F_Ruminococcaceae	unclassified_F_Ruminococcaceae	unclassified_F_Ruminococcaceae
SV371	K_Bacteria	P_Firmicutes	C_Clostridia	O_Clostridiales	F_Clostridiaceae	G_Clostridium sensu stricto 1	unclassified_G_Clostridium sensu stricto 1
SV372	K_Bacteria	P_Firmicutes	C_Clostridia	O_Lachnospirales	F_Lachnospiraceae	G_Lachnoclostridium	unclassified_G_Lachnoclostridium
SV373	K_Bacteria	P_Proteobacteria	C_Gammaproteobacteria	O_Enterobacterales	F_Enterobacteriaceae	G_Salmonella	unclassified_G_Salmonella
SV374	K_Bacteria	P_Firmicutes	C_Clostridia	O_Lachnospirales	F_Lachnospiraceae	G_Lachnospiraceae UCG-006	unclassified_G_Lachnospiraceae UCG-006
SV375	K_Bacteria	P_Firmicutes	C_Clostridia	O_Lachnospirales	F_Lachnospiraceae	G_Marvinbryantia	unclassified_G_Marvinbryantia
SV376	K_Bacteria	P_Firmicutes	C_Clostridia	O_Lachnospirales	F_Lachnospiraceae	G_Lachnospiraceae UCG-001	unclassified_G_Lachnospiraceae UCG-001
SV377	K_Bacteria	P_Firmicutes	C_Clostridia	O_Lachnospirales	F_Lachnospiraceae	unclassified_F_Lachnospiraceae	unclassified_F_Lachnospiraceae
SV378	K_Bacteria	P_Firmicutes	C_Clostridia	O_Lachnospirales	F_Lachnospiraceae	G_Roseburia	unclassified_G_Roseburia
SV379	K_Bacteria	P_Firmicutes	C_Clostridia	O_Lachnospirales	F_Lachnospiraceae	unclassified_F_Lachnospiraceae	unclassified_F_Lachnospiraceae
SV380	K_Bacteria	P_Firmicutes	C_Clostridia	O_Lachnospirales	F_Lachnospiraceae	G_Lachnospiraceae NK4A136 group	unclassified_G_Lachnospiraceae NK4A136 group
SV381	K_Bacteria	P_Firmicutes	C_Clostridia	O_Lachnospirales	F_Lachnospiraceae	G_Roseburia	unclassified_G_Roseburia
SV382	K_Bacteria	P_Firmicutes	C_Clostridia	O_Clostridia UCG-014	unclassified_O_Clostridia UCG-014	unclassified_O_Clostridia UCG-014	unclassified_O_Clostridia UCG-014
SV383	K_Bacteria	P_Firmicutes	C_Clostridia	O_Lachnospirales	F_Lachnospiraceae	G_Lachnospiraceae NK4A136 group	unclassified_G_Lachnospiraceae NK4A136 group
SV384	K_Bacteria	P_Cyanobacteria	C_Vampirivibrionia	O_Gastranaerophilales	unclassified_O_Gastranaerophilales	unclassified_O_Gastranaerophilales	unclassified_O_Gastranaerophilales
SV385	K_Bacteria	P_Firmicutes	C_Clostridia	O_Lachnospirales	F_Lachnospiraceae	G_Lachnospiraceae NK4A136 group	unclassified_G_Lachnospiraceae NK4A136 group
SV386	K_Bacteria	P_Firmicutes	C_Clostridia	O_Clostridia UCG-014	unclassified_O_Clostridia UCG-014	unclassified_O_Clostridia UCG-014	unclassified_O_Clostridia UCG-014
SV387	K_Bacteria	P_Firmicutes	C_Clostridia	O_Lachnospirales	F_Lachnospiraceae	G_Lachnospiraceae NK4A136 group	unclassified_G_Lachnospiraceae NK4A136 group
SV388	K_Bacteria	P_Firmicutes	C_Clostridia	O_Lachnospirales	F_Lachnospiraceae	G_Lachnospiraceae NK4A136 group	unclassified_G_Lachnospiraceae NK4A136 group
SV389	K_Bacteria	P_Bacteroidota	C_Bacteroidia	O_Bacteroidales	F_Muribaculaceae	unclassified_F_Muribaculaceae	unclassified_F_Muribaculaceae
SV390	K_Bacteria	P_Firmicutes	C_Clostridia	O_Lachnospirales	F_Lachnospiraceae	unclassified_F_Lachnospiraceae	unclassified_F_Lachnospiraceae
SV391	K_Bacteria	P_Firmicutes	C_Clostridia	O_Lachnospirales	F_Lachnospiraceae	unclassified_F_Lachnospiraceae	unclassified_F_Lachnospiraceae
SV392	K_Bacteria	P_Firmicutes	C_Clostridia	O_Lachnospirales	F_Lachnospiraceae	G_Lachnospiraceae NK4A136 group	unclassified_G_Lachnospiraceae NK4A136 group
SV393	K_Bacteria	P_Firmicutes	C_Clostridia	O_Lachnospirales	F_Lachnospiraceae	G_[Eubacterium] xylanophilum group	unclassified_G_[Eubacterium] xylanophilum group
SV394	K_Bacteria	P_Firmicutes	C_Clostridia	O_Lachnospirales	F_Lachnospiraceae	G_Lachnospiraceae NK4A136 group	unclassified_G_Lachnospiraceae NK4A136 group
SV395	K_Bacteria	P_Firmicutes	C_Clostridia	O_Oscillospirales	F_[Eubacterium] coprostanoligenes group	unclassified_F_[Eubacterium] coprostanoligenes group	unclassified_F_[Eubacterium] coprostanoligenes group
SV396	K_Bacteria	P_Firmicutes	C_Clostridia	O_Oscillospirales	F_Oscillospiraceae	unclassified_F_Oscillospiraceae	unclassified_F_Oscillospiraceae
SV397	K_Bacteria	P_Firmicutes	C_Clostridia	O_Clostridiales	F_Clostridiaceae	G_Clostridium sensu stricto 1	unclassified_G_Clostridium sensu stricto 1
SV398	K_Bacteria	P_Bacteroidota	C_Bacteroidia	O_Bacteroidales	F_Prevotellaceae	G_Prevotellaceae UCG-001	unclassified_G_Prevotellaceae UCG-001
SV399	K_Bacteria	P_Bacteroidota	C_Bacteroidia	O_Bacteroidales	F_Muribaculaceae	unclassified_F_Muribaculaceae	unclassified_F_Muribaculaceae
SV400	K_Bacteria	P_Bacteroidota	C_Bacteroidia	O_Bacteroidales	F_Muribaculaceae	unclassified_F_Muribaculaceae	unclassified_F_Muribaculaceae
SV401	K_Bacteria	P_Bacteroidota	C_Bacteroidia	O_Bacteroidales	F_Prevotellaceae	G_Prevotellaceae UCG-001	unclassified_G_Prevotellaceae UCG-001
SV402	K_Bacteria	P_Firmicutes	C_Clostridia	O_Oscillospirales	F_Ruminococcaceae	G_Incertae Sedis	unclassified_G_Incertae Sedis
SV403	K_Bacteria	P_Firmicutes	C_Clostridia	O_Lachnospirales	F_Lachnospiraceae	G_Lachnospiraceae NK4A136 group	unclassified_G_Lachnospiraceae NK4A136 group
SV404	K_Bacteria	P_Firmicutes	C_Clostridia	O_Peptostreptococcales-Tissierellales	F_Peptostreptococcaceae	G_Romboutsia	unclassified_G_Romboutsia
SV405	K_Bacteria	P_Firmicutes	C_Clostridia	O_Lachnospirales	F_Lachnospiraceae	G_Lachnospiraceae NK4A136 group	unclassified_G_Lachnospiraceae NK4A136 group
SV406	K_Bacteria	P_Firmicutes	C_Clostridia	O_Lachnospirales	F_Lachnospiraceae	G_Blautia	unclassified_G_Blautia
SV407	K_Bacteria	P_Firmicutes	C_Clostridia	O_Lachnospirales	F_Lachnospiraceae	G_Lachnospiraceae NK4A136 group	unclassified_G_Lachnospiraceae NK4A136 group
SV408	K_Bacteria	P_Firmicutes	C_Clostridia	O_Oscillospirales	F_Ruminococcaceae	G_Ruminococcus	unclassified_G_Ruminococcus
SV409	K_Bacteria	P_Firmicutes	C_Clostridia	O_Oscillospirales	F_Oscillospiraceae	G_Colidextribacter	unclassified_G_Colidextribacter
SV410	K_Bacteria	P_Firmicutes	C_Clostridia	O_Oscillospirales	F_Oscillospiraceae	unclassified_F_Oscillospiraceae	unclassified_F_Oscillospiraceae
SV411	K_Bacteria	P_Firmicutes	C_Clostridia	O_Lachnospirales	F_Lachnospiraceae	G_Lachnospiraceae NK4A136 group	unclassified_G_Lachnospiraceae NK4A136 group
SV412	K_Bacteria	P_Firmicutes	C_Clostridia	O_Lachnospirales	F_Lachnospiraceae	unclassified_F_Lachnospiraceae	unclassified_F_Lachnospiraceae

SV413	K__Bacteria	P__Firmicutes	C__Clostridia	O__Lachnospirales	F__Lachnospiraceae	G__Lachnospiraceae NK4A136 group	unclassified_G__Lachnospiraceae NK4A136 group
SV414	K__Bacteria	P__Bacteroidota	C__Bacteroidia	O__Bacteroidales	F__Bacteroidaceae	G__Bacteroides	unclassified_G__Bacteroides
SV415	K__Bacteria	P__Firmicutes	C__Clostridia	O__Oscillospirales	F__UCG-010	unclassified_F__UCG-010	unclassified_F__UCG-010
SV416	K__Bacteria	P__Firmicutes	C__Clostridia	O__Peptostreptococcales-Tissierellales	F__Peptostreptococcaceae	G__Romboutsia	S__ilealis
SV417	K__Bacteria	P__Firmicutes	C__Clostridia	O__Lachnospirales	F__Lachnospiraceae	G__Lachnospiraceae UCG-001	unclassified_G__Lachnospiraceae UCG-001
SV418	K__Bacteria	P__Firmicutes	C__Clostridia	O__Oscillospirales	F__Oscillospiraceae	G__UCG-003	unclassified_G__UCG-003
SV419	K__Bacteria	P__Firmicutes	C__Clostridia	O__Oscillospirales	F__Ruminococcaceae	G__Incertae Sedis	unclassified_G__Incertae Sedis
SV420	K__Bacteria	P__Firmicutes	C__Clostridia	O__Lachnospirales	F__Lachnospiraceae	unclassified_F__Lachnospiraceae	unclassified_F__Lachnospiraceae
SV421	K__Bacteria	P__Firmicutes	C__Clostridia	O__Lachnospirales	F__Lachnospiraceae	G__Roseburia	unclassified_G__Roseburia
SV422	K__Bacteria	P__Firmicutes	C__Clostridia	O__Lachnospirales	F__Lachnospiraceae	G__Lachnoclostridium	unclassified_G__Lachnoclostridium
SV423	K__Bacteria	P__Actinobacteriota	C__Coriobacteriia	O__Coriobacteriales	F__Atopobiaceae	G__Coriobacteriaceae UCG-002	unclassified_G__Coriobacteriaceae UCG-002
SV424	K__Bacteria	P__Firmicutes	C__Clostridia	O__Lachnospirales	F__Lachnospiraceae	G__Lachnospiraceae NK4A136 group	unclassified_G__Lachnospiraceae NK4A136 group
SV425	K__Bacteria	P__Firmicutes	C__Clostridia	O__Lachnospirales	F__Lachnospiraceae	G__Lachnospiraceae UCG-001	unclassified_G__Lachnospiraceae UCG-001
SV426	K__Bacteria	P__Firmicutes	C__Clostridia	O__Lachnospirales	F__Lachnospiraceae	G__Lachnospiraceae NK4A136 group	unclassified_G__Lachnospiraceae NK4A136 group
SV427	K__Bacteria	P__Firmicutes	C__Clostridia	O__Lachnospirales	F__Lachnospiraceae	G__Lachnospiraceae NK4A136 group	unclassified_G__Lachnospiraceae NK4A136 group
SV428	K__Bacteria	P__Bacteroidota	C__Bacteroidia	O__Bacteroidales	F__Muribaculaceae	unclassified_F__Muribaculaceae	unclassified_F__Muribaculaceae
SV429	K__Bacteria	P__Firmicutes	C__Clostridia	O__Lachnospirales	F__Lachnospiraceae	G__Lachnoclostridium	unclassified_G__Lachnoclostridium
SV430	K__Bacteria	P__Firmicutes	C__Clostridia	O__Lachnospirales	F__Lachnospiraceae	G__Lachnospiraceae FCS020 group	unclassified_G__Lachnospiraceae FCS020 group
SV431	K__Bacteria	P__Firmicutes	C__Clostridia	O__Oscillospirales	F__Ruminococcaceae	G__Ruminococcus	unclassified_G__Ruminococcus
SV432	K__Bacteria	P__Firmicutes	C__Clostridia	O__Lachnospirales	F__Lachnospiraceae	unclassified_F__Lachnospiraceae	unclassified_F__Lachnospiraceae
SV433	K__Bacteria	P__Firmicutes	C__Clostridia	O__Lachnospirales	F__Lachnospiraceae	G__Eisenbergiella	S__massiliensis
SV434	K__Bacteria	P__Bacteroidota	C__Bacteroidia	O__Bacteroidales	F__Rikenellaceae	G__Alistipes	unclassified_G__Alistipes
SV435	K__Bacteria	P__Firmicutes	C__Clostridia	O__Lachnospirales	F__Lachnospiraceae	G__Lachnospiraceae UCG-001	unclassified_G__Lachnospiraceae UCG-001
SV436	K__Bacteria	P__Firmicutes	C__Clostridia	O__Lachnospirales	F__Lachnospiraceae	unclassified_F__Lachnospiraceae	unclassified_F__Lachnospiraceae
SV437	K__Bacteria	P__Firmicutes	C__Clostridia	O__Lachnospirales	F__Lachnospiraceae	unclassified_F__Lachnospiraceae	unclassified_F__Lachnospiraceae
SV438	K__Bacteria	P__Firmicutes	C__Clostridia	O__Lachnospirales	F__Lachnospiraceae	G__Lachnospiraceae NK4A136 group	unclassified_G__Lachnospiraceae NK4A136 group
SV439	K__Bacteria	P__Firmicutes	C__Clostridia	O__Clostridia UCG-014	unclassified_O__Clostridia UCG-014	unclassified_O__Clostridia UCG-014	unclassified_O__Clostridia UCG-014
SV440	K__Bacteria	P__Firmicutes	C__Clostridia	O__Lachnospirales	F__Lachnospiraceae	G__Lachnoclostridium	unclassified_G__Lachnoclostridium
SV441	K__Bacteria	P__Firmicutes	C__Clostridia	O__Oscillospirales	F__Oscillospiraceae	G__NK4A214 group	unclassified_G__NK4A214 group
SV442	K__Bacteria	P__Firmicutes	C__Bacilli	O__RF39	unclassified_O__RF39	unclassified_O__RF39	unclassified_O__RF39
SV443	K__Bacteria	P__Firmicutes	C__Clostridia	O__Oscillospirales	F__Butyricicoccaceae	G__Butyricicoccus	unclassified_G__Butyricicoccus
SV444	K__Bacteria	P__Firmicutes	C__Clostridia	O__Lachnospirales	F__Lachnospiraceae	unclassified_F__Lachnospiraceae	unclassified_F__Lachnospiraceae
SV445	K__Bacteria	P__Firmicutes	C__Clostridia	O__Lachnospirales	F__Lachnospiraceae	G__Lachnospiraceae NK4A136 group	unclassified_G__Lachnospiraceae NK4A136 group
SV446	K__Bacteria	P__Bacteroidota	C__Bacteroidia	O__Bacteroidales	F__Muribaculaceae	unclassified_F__Muribaculaceae	unclassified_F__Muribaculaceae
SV447	K__Bacteria	P__Firmicutes	C__Clostridia	O__Lachnospirales	F__Lachnospiraceae	G__Lachnospiraceae NK4A136 group	unclassified_G__Lachnospiraceae NK4A136 group
SV448	K__Bacteria	P__Firmicutes	C__Clostridia	O__Lachnospirales	F__Lachnospiraceae	unclassified_F__Lachnospiraceae	unclassified_F__Lachnospiraceae
SV449	K__Bacteria	P__Firmicutes	C__Clostridia	O__Lachnospirales	F__Lachnospiraceae	G__Lachnospiraceae NK4A136 group	unclassified_G__Lachnospiraceae NK4A136 group
SV450	K__Bacteria	P__Firmicutes	C__Clostridia	O__Lachnospirales	F__Lachnospiraceae	G__Roseburia	unclassified_G__Roseburia
SV451	K__Bacteria	P__Firmicutes	C__Bacilli	O__Lactobacillales	F__Lactobacillaceae	G__Ligilactobacillus	unclassified_G__Ligilactobacillus
SV452	K__Bacteria	P__Firmicutes	C__Clostridia	O__Oscillospirales	F__Oscillospiraceae	unclassified_F__Oscillospiraceae	unclassified_F__Oscillospiraceae
SV453	K__Bacteria	P__Firmicutes	C__Clostridia	O__Lachnospirales	F__Lachnospiraceae	G__Lachnospiraceae NK4A136 group	unclassified_G__Lachnospiraceae NK4A136 group
SV454	K__Bacteria	P__Firmicutes	C__Clostridia	O__Oscillospirales	F__Ruminococcaceae	G__Candidatus Soleaferrea	unclassified_G__Candidatus Soleaferrea
SV455	K__Bacteria	P__Firmicutes	C__Clostridia	O__Clostridia UCG-014	unclassified_O__Clostridia UCG-014	unclassified_O__Clostridia UCG-014	unclassified_O__Clostridia UCG-014
SV456	K__Bacteria	P__Firmicutes	C__Clostridia	O__Clostridia UCG-014	unclassified_O__Clostridia UCG-014	unclassified_O__Clostridia UCG-014	unclassified_O__Clostridia UCG-014
SV457	K__Bacteria	P__Firmicutes	C__Clostridia	O__Clostridia UCG-014	unclassified_O__Clostridia UCG-014	unclassified_O__Clostridia UCG-014	unclassified_O__Clostridia UCG-014
SV458	K__Bacteria	P__Firmicutes	C__Clostridia	O__Peptostreptococcales-Tissierellales	F__Peptostreptococcaceae	G__Romboutsia	unclassified_G__Romboutsia
SV459	K_Bacteria	P_Firmicutes	C_Clostridia	O_Lachnospirales	F_Lachnospiraceae	unclassified_F_Lachnospiraceae	unclassified_F_Lachnospiraceae
SV460	K__Bacteria	P__Firmicutes	C__Clostridia	O__Lachnospirales	F__Lachnospiraceae	unclassified_F__Lachnospiraceae	unclassified_F__Lachnospiraceae
SV461	K__Bacteria	P__Firmicutes	C__Clostridia	O__Oscillospirales	F__Oscillospiraceae	G__Colidextribacter	unclassified_G__Colidextribacter
SV462	K__Bacteria	P__Firmicutes	C__Clostridia	O__Oscillospirales	F__Oscillospiraceae	G__Colidextribacter	unclassified_G__Colidextribacter
SV463	K__Bacteria	P__Firmicutes	C__Clostridia	O__Lachnospirales	F__Lachnospiraceae	unclassified_F__Lachnospiraceae	unclassified_F__Lachnospiraceae
SV464	K__Bacteria	P__Bacteroidota	C__Bacteroidia	O__Bacteroidales	F__Muribaculaceae	unclassified_F__Muribaculaceae	unclassified_F__Muribaculaceae
SV465	K_Bacteria	P_Firmicutes	C_Clostridia	O_Lachnospirales	F_Lachnospiraceae	G_[Eubacterium] ruminantium group	unclassified_G_[Eubacterium] ruminantium group
SV466	K__Bacteria	P__Firmicutes	C__Clostridia	O__Lachnospirales	F__Lachnospiraceae	unclassified_F__Lachnospiraceae	unclassified_F__Lachnospiraceae
SV467	K__Bacteria	P__Firmicutes	C__Clostridia	O__Lachnospirales	F__Lachnospiraceae	unclassified_F__Lachnospiraceae	unclassified_F__Lachnospiraceae
SV468	K__Bacteria	P__Firmicutes	C__Clostridia	O__Lachnospirales	F__Lachnospiraceae	G__Lachnospiraceae NK4A136 group	unclassified_G__Lachnospiraceae NK4A136 group
SV469	K__Bacteria	P__Firmicutes	C__Clostridia	O__Oscillospirales	F__Oscillospiraceae	G__UCG-003	unclassified_G__UCG-003
SV470	K__Bacteria	P__Firmicutes	C__Clostridia	O__Oscillospirales	F__Ruminococcaceae	unclassified_F__Ruminococcaceae	unclassified_F__Ruminococcaceae
SV471	K_Bacteria	P_Firmicutes	C_Clostridia	O_Lachnospirales	F_Lachnospiraceae	G_Lachnospiraceae NK4A136 group	unclassified_G_Lachnospiraceae NK4A136 group
SV472	K__Bacteria	P__Firmicutes	C__Clostridia	O__Oscillospirales	F__Oscillospiraceae	unclassified_F__Oscillospiraceae	unclassified_F__Oscillospiraceae
SV473	K__Bacteria	P__Firmicutes	C__Clostridia	O__Lachnospirales	F__Lachnospiraceae	G__[Eubacterium] ventriosum group	unclassified_G__[Eubacterium] ventriosum group
SV474	K__Bacteria	P__Firmicutes	C__Clostridia	O__Oscillospirales	F__Oscillospiraceae	unclassified_F__Oscillospiraceae	unclassified_F__Oscillospiraceae
SV475	K__Bacteria	P__Firmicutes	C__Clostridia	O__Lachnospirales	F__Lachnospiraceae	G__Lachnospiraceae UCG-006	unclassified_G__Lachnospiraceae UCG-006
SV476	K__Bacteria	P__Firmicutes	C__Clostridia	O__Lachnospirales	F__Lachnospiraceae	G__Lachnoclostridium	unclassified_G__Lachnoclostridium
SV477	K_Bacteria	P_Firmicutes	C_Clostridia	O_Lachnospirales	F_Lachnospiraceae	G_Marvinbryantia	unclassified_G_Marvinbryantia
SV478	K__Bacteria	P__Firmicutes	C__Clostridia	O__Lachnospirales	F__Lachnospiraceae	G__ASF356	unclassified_G__ASF356
SV479	K__Bacteria	P__Firmicutes	C__Clostridia	O__Oscillospirales	F__Ruminococcaceae	unclassified_F__Ruminococcaceae	unclassified_F__Ruminococcaceae
SV480	K__Bacteria	P__Firmicutes	C__Clostridia	O__Oscillospirales	F__Oscillospiraceae	unclassified_F__Oscillospiraceae	unclassified_F__Oscillospiraceae
SV481	K__Bacteria	P__Firmicutes	C__Clostridia	O__Lachnospirales	F__Lachnospiraceae	G__[Eubacterium] ventriosum group	unclassified_G__[Eubacterium] ventriosum group
SV482	K__Bacteria	P__Firmicutes	C__Clostridia	O__Lachnospirales	F__Lachnospiraceae	G__GCA-900066575	unclassified_G__GCA-900066575
SV483	K_Bacteria	P_Firmicutes	C_Clostridia	O_Lachnospirales	F_Lachnospiraceae	G_Lachnospiraceae UCG-006	unclassified_G_Lachnospiraceae UCG-006
SV484	K__Bacteria	P__Firmicutes	C__Clostridia	O__Lachnospirales	F__Lachnospiraceae	G__Anaerostipes	unclassified_G__Anaerostipes
SV485	K__Bacteria	P__Firmicutes	C__Clostridia	O__Oscillospirales	F__Oscillospiraceae	G__Oscillibacter	unclassified_G__Oscillibacter
SV486	K__Bacteria	P__Cyanobacteria	C__Vampirivibrionia	O__Gastranaerophilales	unclassified_O__Gastranaerophilales	unclassified_O__Gastranaerophilales	unclassified_O__Gastranaerophilales
SV487	K__Bacteria	P__Firmicutes	C__Clostridia	O__Lachnospirales	F__Lachnospiraceae	unclassified_F__Lachnospiraceae	unclassified_F__Lachnospiraceae
SV488	K__Bacteria	P__Firmicutes	C__Clostridia	O__Clostridia UCG-014	unclassified_O__Clostridia UCG-014	unclassified_O__Clostridia UCG-014	unclassified_O__Clostridia UCG-014
SV489	K__Bacteria	P__Firmicutes	C__Clostridia	O__Oscillospirales	F__Ruminococcaceae	G__Ruminococcus	unclassified_G__Ruminococcus
SV490	K__Bacteria	P__Firmicutes	C__Clostridia	O__Oscillospirales	F__Ruminococcaceae	G__Anaerotruncus	unclassified_G__Anaerotruncus
SV491	K__Bacteria	P__Firmicutes	C__Clostridia	O__Clostridiales	F__Clostridiaceae	G__Clostridium sensu stricto 1	unclassified_G__Clostridium sensu stricto 1
SV492	K__Bacteria	P__Firmicutes	C__Clostridia	O__Peptostreptococcales-Tissierellales	F__Anaerovoracaceae	G__Family XIII AD3011 group	unclassified_G__Family XIII AD3011 group
SV493	K__Bacteria	P__Firmicutes	C__Clostridia	O__Lachnospirales	F__Lachnospiraceae	unclassified_F__Lachnospiraceae	unclassified_F__Lachnospiraceae
SV494	K__Bacteria	P__Firmicutes	C__Bacilli	O__Erysipelotrichales	F__Erysipelotrichaceae	G__Dubosiella	unclassified_G__Dubosiella
SV495	K__Bacteria	P__Firmicutes	C__Clostridia	O__Lachnospirales	F__Lachnospiraceae	G__Marvinbryantia	unclassified_G__Marvinbryantia
SV496	K__Bacteria	P__Firmicutes	C__Bacilli	O__Erysipelotrichales	F__Erysipelotrichaceae	G__Turicibacter	unclassified_G__Turicibacter
SV497	K__Bacteria	P__Firmicutes	C__Clostridia	O__Lachnospirales	F__Lachnospiraceae	G__Marvinbryantia	unclassified_G__Marvinbryantia
SV498	K__Bacteria	P__Firmicutes	C__Clostridia	O__Lachnospirales	F__Lachnospiraceae	G__Acetitomaculum	unclassified_G__Acetitomaculum
SV499	K__Bacteria	P__Firmicutes	C__Clostridia	O__Lachnospirales	F__Lachnospiraceae	unclassified_F__Lachnospiraceae	unclassified_F__Lachnospiraceae
SV500	K__Bacteria	P__Firmicutes	C__Clostridia	O__Lachnospirales	F__Lachnospiraceae	G__Lachnospiraceae NK4A136 group	unclassified_G__Lachnospiraceae NK4A136 group
SV501	K__Bacteria	P__Firmicutes	C__Clostridia	O__Oscillospirales	F__Ruminococcaceae	G__Candidatus Soleaferrea	unclassified_G__Candidatus Soleaferrea
SV502	K__Bacteria	P__Firmicutes	C__Clostridia	O__Lachnospirales	F__Lachnospiraceae	G__Roseburia	unclassified_G__Roseburia
SV503	K__Bacteria	P__Firmicutes	C__Clostridia	O__Peptostreptococcales-Tissierellales	F__Anaerovoracaceae	G__Family XIII AD3011 group	unclassified_G__Family XIII AD3011 group
SV504	K__Bacteria	P__Firmicutes	C__Clostridia	O__Lachnospirales	F__Lachnospiraceae	G__Lachnospiraceae NK4A136 group	unclassified_G__Lachnospiraceae NK4A136 group
SV505	K__Bacteria	P__Firmicutes	C__Clostridia	O__Lachnospirales	F__Lachnospiraceae	unclassified_F__Lachnospiraceae	unclassified_F__Lachnospiraceae
SV506	K__Bacteria	P__Firmicutes	C__Clostridia	O__Peptostreptococcales-Tissierellales	F__Peptostreptococcaceae	G__Romboutsia	unclassified_G__Romboutsia
SV507	K__Bacteria	P__Firmicutes	C__Bacilli	O__Erysipelotrichales	F__Erysipelotrichaceae	unclassified_F__Erysipelotrichaceae	unclassified_F__Erysipelotrichaceae
SV508	K__Bacteria	P__Bacteroidota	C__Bacteroidia	O__Bacteroidales	F__Muribaculaceae	G__Muribaculum	S__intestinale
SV509	K__Bacteria	P__Firmicutes	C__Clostridia	O__Clostridia UCG-014	unclassified_O__Clostridia UCG-014	unclassified_O__Clostridia UCG-014	unclassified_O__Clostridia UCG-014
SV510	K__Bacteria	P__Firmicutes	C__Clostridia	O__Lachnospirales	F__Lachnospiraceae	G__Lachnospiraceae NK4A136 group	unclassified_G__Lachnospiraceae NK4A136 group
SV511	K__Bacteria	P__Firmicutes	C__Clostridia	O__Lachnospirales	F__Lachnospiraceae	G__Lachnoclostridium	unclassified_G__Lachnoclostridium
SV512	K__Bacteria	P__Firmicutes	C__Clostridia	O__Lachnospirales	F__Lachnospiraceae	G__Marvinbryantia	unclassified_G__Marvinbryantia
SV513	K__Bacteria	P__Firmicutes	C__Clostridia	O__Lachnospirales	F__Lachnospiraceae	G__Blautia	unclassified_G__Blautia
SV514	K__Bacteria	P__Firmicutes	C__Clostridia	O__Lachnospirales	F__Lachnospiraceae	unclassified_F__Lachnospiraceae	unclassified_F__Lachnospiraceae
SV515	K__Bacteria	P__Firmicutes	C__Clostridia	O__Oscillospirales	F__Oscillospiraceae	unclassified_F__Oscillospiraceae	unclassified_F__Oscillospiraceae
SV516	K__Bacteria	P__Firmicutes	C__Clostridia	O__Lachnospirales	F__Lachnospiraceae	G__Lachnospiraceae UCG-001	unclassified_G__Lachnospiraceae UCG-001
SV517	K__Bacteria	P__Firmicutes	C__Clostridia	O__Lachnospirales	F__Lachnospiraceae	G__Roseburia	unclassified_G__Roseburia
SV518	K__Bacteria	P__Firmicutes	C__Clostridia	O__Lachnospirales	F__Lachnospiraceae	unclassified_F__Lachnospiraceae	unclassified_F__Lachnospiraceae
SV519	K__Bacteria	P__Proteobacteria	C__Gammaproteobacteria	O__Burkholderiales	F__Sutterellaceae	G__Parasutterella	unclassified_G__Parasutterella
SV520	K__Bacteria	P__Firmicutes	C__Clostridia	O__Lachnospirales	F__Lachnospiraceae	G__A2	unclassified_G__A2
SV521	K__Bacteria	P__Firmicutes	C__Clostridia	O__Oscillospirales	F__Ruminococcaceae	G__Ruminococcus	unclassified_G__Ruminococcus
SV522	K__Bacteria	P__Firmicutes	C__Clostridia	O__Lachnospirales	F__Lachnospiraceae	unclassified_F__Lachnospiraceae	unclassified_F__Lachnospiraceae
SV523	K__Bacteria	P__Bacteroidota	C__Bacteroidia	O__Bacteroidales	F__Muribaculaceae	unclassified_F__Muribaculaceae	unclassified_F__Muribaculaceae
SV524	K_Bacteria	P_Firmicutes	C_Bacilli	O_Erysipelotrichales	F_Erysipelatoclostridiaceae	G_Candidatus Stoquefichus	unclassified_G_Candidatus Stoquefichus
SV525	K__Bacteria	P__Firmicutes	C__Clostridia	O__Lachnospirales	F__Lachnospiraceae	G__Lachnospiraceae UCG-001	unclassified_G__Lachnospiraceae UCG-001
SV526	K__Bacteria	P__Firmicutes	C__Clostridia	O__Oscillospirales	F__Ruminococcaceae	G__Ruminococcus	unclassified_G__Ruminococcus
SV527	K__Bacteria	P__Firmicutes	C__Clostridia	O__Lachnospirales	F__Lachnospiraceae	unclassified_F__Lachnospiraceae	unclassified_F__Lachnospiraceae
SV528	K__Bacteria	P__Firmicutes	C__Bacilli	O__Erysipelotrichales	F__Erysipelotrichaceae	unclassified_F__Erysipelotrichaceae	unclassified_F__Erysipelotrichaceae
SV529	K__Bacteria	P__Firmicutes	C__Clostridia	O__Oscillospirales	F__Butyricicoccaceae	G__Butyricicoccus	unclassified_G__Butyricicoccus
SV530	K_Bacteria	P_Firmicutes	C_Clostridia	O_Lachnospirales	F_Lachnospiraceae	G_Lachnoclostridium	unclassified_G_Lachnoclostridium
SV531	K__Bacteria	P__Proteobacteria	C__Alphaproteobacteria	O__Rhodospirillales	unclassified_O__Rhodospirillales	unclassified_O__Rhodospirillales	unclassified_O__Rhodospirillales
SV532	K__Bacteria	P__Firmicutes	C__Clostridia	O__Christensenellales	F__Christensenellaceae	G__Christensenellaceae R-7 group	unclassified_G__Christensenellaceae R-7 group
SV533	K__Bacteria	P__Firmicutes	C__Clostridia	O__Lachnospirales	F__Lachnospiraceae	G__Lachnoclostridium	unclassified_G__Lachnoclostridium
SV534	K__Bacteria	P__Firmicutes	C__Clostridia	O__Oscillospirales	F__Ruminococcaceae	G__Anaerotruncus	unclassified_G__Anaerotruncus
SV535	K__Bacteria	P__Firmicutes	C__Bacilli	O__Lactobacillales	F__Lactobacillaceae	G__HT002	unclassified_G__HT002
SV536	K_Bacteria	P_Firmicutes	C_Clostridia	O_Lachnospirales	F_Lachnospiraceae	G_Blautia	unclassified_G_Blautia
SV537	K__Bacteria	P__Firmicutes	C__Clostridia	O__Lachnospirales	F__Lachnospiraceae	G__Lachnospiraceae NK4A136 group	unclassified_G__Lachnospiraceae NK4A136 group
SV538	K__Bacteria	P__Firmicutes	C__Clostridia	O__Lachnospirales	F__Lachnospiraceae	unclassified_F__Lachnospiraceae	unclassified_F__Lachnospiraceae
SV539	K__Bacteria	P__Firmicutes	C__Clostridia	O__Lachnospirales	F__Lachnospiraceae	G__Blautia	unclassified_G__Blautia
SV540	K__Bacteria	P__Firmicutes	C__Clostridia	O__Lachnospirales	F__Lachnospiraceae	unclassified_F__Lachnospiraceae	unclassified_F__Lachnospiraceae
SV541	K__Bacteria	P__Firmicutes	C__Clostridia	O__Oscillospirales	F__Ruminococcaceae	G__Incertae Sedis	unclassified_G__Incertae Sedis
SV542	K_Bacteria	P_Firmicutes	C_Clostridia	O_Lachnospirales	F_Lachnospiraceae	G_A2	unclassified_G_A2
SV543	K__Bacteria	P__Firmicutes	C__Clostridia	O__Lachnospirales	F__Lachnospiraceae	unclassified_F__Lachnospiraceae	unclassified_F__Lachnospiraceae
SV544	K__Bacteria	P__Firmicutes	C__Bacilli	O__Lactobacillales	F__Lactobacillaceae	G__Lactobacillus	unclassified_G__Lactobacillus
SV545	K__Bacteria	P__Firmicutes	C__Clostridia	O__Oscillospirales	F__Oscillospiraceae	unclassified_F__Oscillospiraceae	unclassified_F__Oscillospiraceae
SV546	K__Bacteria	P__Firmicutes	C__Bacilli	O__Erysipelotrichales	F__Erysipelotrichaceae	G__Turicibacter	unclassified_G__Turicibacter
SV547	K__Bacteria	P__Firmicutes	C__Bacilli	O__Erysipelotrichales	F__Erysipelotrichaceae	G__Turicibacter	unclassified_G__Turicibacter
SV548	K__Bacteria	P__Firmicutes	C__Clostridia	O__Lachnospirales	F__Lachnospiraceae	unclassified_F__Lachnospiraceae	unclassified_F__Lachnospiraceae
SV549	K__Bacteria	P__Firmicutes	C__Clostridia	O__Lachnospirales	F__Lachnospiraceae	G__Marvinbryantia	unclassified_G__Marvinbryantia
SV550	K__Bacteria	P__Firmicutes	C__Clostridia	O__Lachnospirales	F__Lachnospiraceae	G__Lachnospiraceae NK4A136 group	unclassified_G__Lachnospiraceae NK4A136 group

SV551	K Bacteria	P Firmicutes	C Clostridia	O Lachnospirales	F Lachnospiraceae	unclassified F Lachnospiraceae	unclassified F Lachnospiraceae
SV552	K Bacteria	P Firmicutes	C Clostridia	O Oscillospirales	F Ruminococcaceae	G Paludicola	unclassified G Paludicola
SV553	K Bacteria	P Firmicutes	C Clostridia	O Lachnospirales	F Lachnospiraceae	G Lachnospiraceae UCG-001	unclassified G Lachnospiraceae UCG-001
SV554	K Bacteria	P Firmicutes	C Clostridia	O Oscillospirales	F Oscillospiraceae	G Colidextribacter	unclassified G Colidextribacter
SV555	K Bacteria	P Firmicutes	C Bacilli	O Erysipelotrichales	F Erysipelotrichaceae	G Dubosiella	unclassified G Dubosiella
SV556	K Bacteria	P Firmicutes	C Clostridia	O Lachnospirales	F Lachnospiraceae	G Roseburia	unclassified G Roseburia
SV557	K Bacteria	P Firmicutes	C Clostridia	O Oscillospirales	F Oscillospiraceae	G UCG-005	unclassified G UCG-005
SV558	K Bacteria	P Firmicutes	C Clostridia	O Lachnospirales	F Lachnospiraceae	G Lachnospiraceae NK4A136 group	unclassified G Lachnospiraceae NK4A136 group
SV559	K Bacteria	P Firmicutes	C Clostridia	O Lachnospirales	F Lachnospiraceae	unclassified F Lachnospiraceae	unclassified F Lachnospiraceae
SV560	K Bacteria	P Desulfobacterota	C Desulfovibrionia	O Desulfovibrionales	F Desulfovibrionaceae	G Bilophila	unclassified G Bilophila
SV561	K Bacteria	P Firmicutes	C Clostridia	O Lachnospirales	F Lachnospiraceae	unclassified F Lachnospiraceae	unclassified F Lachnospiraceae
SV562	K Bacteria	P Firmicutes	C Clostridia	O Lachnospirales	F Lachnospiraceae	unclassified F Lachnospiraceae	unclassified F Lachnospiraceae
SV563	K Bacteria	P Bacteroidota	C Bacteroidia	O Bacteroidales	F Muribaculaceae	unclassified F Muribaculaceae	unclassified F Muribaculaceae
SV564	K Bacteria	P Firmicutes	C Clostridia	O Lachnospirales	F Lachnospiraceae	G Lachnospiraceae NK4A136 group	unclassified G Lachnospiraceae NK4A136 group
SV565	K Bacteria	P Bacteroidota	C Bacteroidia	O Bacteroidales	F Muribaculaceae	unclassified F Muribaculaceae	unclassified F Muribaculaceae
SV566	K Bacteria	P Firmicutes	C Clostridia	O Clostridia UCG-014	unclassified O Clostridia UCG-014	unclassified O Clostridia UCG-014	unclassified O Clostridia UCG-014
SV567	K Bacteria	P Bacteroidota	C Bacteroidia	O Bacteroidales	F Muribaculaceae	unclassified F Muribaculaceae	unclassified F Muribaculaceae
SV568	K Bacteria	P Cyanobacteria	C Vampirivibrionia	O Gastranaerophilales	unclassified O Gastranaerophilales	unclassified O Gastranaerophilales	unclassified O Gastranaerophilales
SV569	K Bacteria	P Firmicutes	C Clostridia	O Clostridia UCG-014	unclassified O Clostridia UCG-014	unclassified O Clostridia UCG-014	unclassified O Clostridia UCG-014
SV570	K Bacteria	P Proteobacteria	C Alphaproteobacteria	O Rhodospirillales	unclassified O Rhodospirillales	unclassified O Rhodospirillales	unclassified O Rhodospirillales
SV571	K Bacteria	P Firmicutes	C Clostridia	O Lachnospirales	F Lachnospiraceae	unclassified F Lachnospiraceae	unclassified F Lachnospiraceae
SV572	K Bacteria	P Firmicutes	C Clostridia	O Lachnospirales	F Lachnospiraceae	G Lachnospiraceae NK4A136 group	unclassified G Lachnospiraceae NK4A136 group
SV573	K Bacteria	P Firmicutes	C Clostridia	O Lachnospirales	F Lachnospiraceae	unclassified F Lachnospiraceae	unclassified F Lachnospiraceae
SV574	K Bacteria	P Firmicutes	C Clostridia	O Lachnospirales	F Lachnospiraceae	G Acetatifactor	unclassified G Acetatifactor
SV575	K Bacteria	P Firmicutes	C Clostridia	O Lachnospirales	F Lachnospiraceae	unclassified F Lachnospiraceae	unclassified F Lachnospiraceae
SV576	K Bacteria	P Firmicutes	C Clostridia	O Lachnospirales	F Lachnospiraceae	unclassified F Lachnospiraceae	unclassified F Lachnospiraceae
SV577	K Bacteria	P Firmicutes	C Bacilli	O Lactobacillales	F Lactobacillaceae	G Lactiplantibacillus	unclassified G Lactiplantibacillus
SV578	K Bacteria	P Firmicutes	C Clostridia	O Oscillospirales	F Ruminococcaceae	G Anaerotruncus	unclassified G Anaerotruncus
SV579	K Bacteria	P Firmicutes	C Clostridia	O Lachnospirales	F Lachnospiraceae	G Lachnospiraceae NK4A136 group	unclassified G Lachnospiraceae NK4A136 group
SV580	K Bacteria	P Firmicutes	C Clostridia	O Lachnospirales	F Lachnospiraceae	G Lachnospiraceae NK4A136 group	unclassified G Lachnospiraceae NK4A136 group
SV581	K Bacteria	P Actinobacteriota	C Coriobacteriia	O Coriobacteriales	F Eggerthellaceae	G Enterorhabdus	unclassified G Enterorhabdus
SV582	K Bacteria	P Firmicutes	C Clostridia	O Oscillospirales	F Oscillospiraceae	unclassified F Oscillospiraceae	unclassified F Oscillospiraceae
SV583	K Bacteria	P Firmicutes	C Clostridia	O Lachnospirales	F Lachnospiraceae	G Roseburia	unclassified G Roseburia
SV584	K Bacteria	P Firmicutes	C Clostridia	O Lachnospirales	F Lachnospiraceae	G A2	unclassified G A2
SV585	K Bacteria	P Bacteroidota	C Bacteroidia	O Bacteroidales	F Muribaculaceae	G Muribaculum	S intestinale
SV586	K Bacteria	P Firmicutes	C Clostridia	O Lachnospirales	F Lachnospiraceae	G Roseburia	unclassified G Roseburia
SV587	K Bacteria	P Firmicutes	C Clostridia	O Lachnospirales	F Lachnospiraceae	G Roseburia	unclassified G Roseburia
SV588	K Bacteria	P Firmicutes	C Clostridia	O Lachnospirales	F Lachnospiraceae	G Marvinbryantia	unclassified G Marvinbryantia
SV589	K Bacteria	P Firmicutes	C Clostridia	O Lachnospirales	F Lachnospiraceae	G [Eubacterium] xylanophilum group	unclassified G [Eubacterium] xylanophilum group
SV590	K Bacteria	P Firmicutes	C Clostridia	O Clostridia vadinBB60 group	unclassified O Clostridia vadinBB60 group	unclassified O Clostridia vadinBB60 group	unclassified O Clostridia vadinBB60 group
SV591	K Bacteria	P Firmicutes	C Clostridia	O Oscillospirales	F Ruminococcaceae	G Incertae Sedis	unclassified G Incertae Sedis
SV592	K Bacteria	P Firmicutes	C Clostridia	O Lachnospirales	F Lachnospiraceae	unclassified F Lachnospiraceae	unclassified F Lachnospiraceae
SV593	K Bacteria	P Firmicutes	C Clostridia	O Lachnospirales	F Lachnospiraceae	unclassified F Lachnospiraceae	unclassified F Lachnospiraceae
SV594	K Bacteria	P Firmicutes	C Clostridia	O Lachnospirales	F Lachnospiraceae	G Lachnospiraceae NK4A136 group	unclassified G Lachnospiraceae NK4A136 group
SV595	K Bacteria	P Bacteroidota	C Bacteroidia	O Bacteroidales	F Muribaculaceae	unclassified F Muribaculaceae	unclassified F Muribaculaceae
SV596	K Bacteria	P Firmicutes	C Clostridia	O Oscillospirales	F Ruminococcaceae	G Incertae Sedis	unclassified G Incertae Sedis
SV597	K_Bacteria	P_Firmicutes	C_Clostridia	O_Christensenellales	F_Christensenellaceae	G_Christensenellaceae R-7 group	unclassified_G_Christensenellaceae R-7 group
SV598	K Bacteria	P Firmicutes	C Clostridia	O Oscillospirales	F Ruminococcaceae	G UBA1819	unclassified G UBA1819
SV599	K Bacteria	P Bacteroidota	C Bacteroidia	O Bacteroidales	F Prevotellaceae	G Prevotellaceae UCG-001	unclassified G Prevotellaceae UCG-001
SV600	K Bacteria	P Firmicutes	C Bacilli	O Erysipelotrichales	F Erysipelotrichaceae	G Turicibacter	unclassified G Turicibacter
SV601	K Bacteria	P Bacteroidota	C Bacteroidia	O Bacteroidales	F Rikenellaceae	G Alistipes	unclassified G Alistipes
SV602	K Bacteria	P Firmicutes	C Clostridia	O Lachnospirales	F Lachnospiraceae	G Lachnoclostridium	unclassified G Lachnoclostridium
SV603	K_Bacteria	P_Firmicutes	C_Clostridia	O_Lachnospirales	F_Lachnospiraceae	G_Lachnospiraceae NK4A136 group	unclassified_G_Lachnospiraceae NK4A136 group
SV604	K Bacteria	P Bacteroidota	C Bacteroidia	O Bacteroidales	F Muribaculaceae	unclassified F Muribaculaceae	unclassified F Muribaculaceae
SV605	K Bacteria	P Firmicutes	C Clostridia	O Oscillospirales	F Ruminococcaceae	unclassified F Ruminococcaceae	unclassified F Ruminococcaceae
SV606	K Bacteria	P Firmicutes	C Clostridia	O Lachnospirales	F Lachnospiraceae	G Lachnospiraceae FCS020 group	unclassified G Lachnospiraceae FCS020 group
SV607	K Bacteria	P Firmicutes	C Clostridia	O Lachnospirales	F Lachnospiraceae	unclassified F Lachnospiraceae	unclassified F Lachnospiraceae
SV608	K Bacteria	P Firmicutes	C Clostridia	O Lachnospirales	F Lachnospiraceae	G Lachnospiraceae UCG-001	unclassified G Lachnospiraceae UCG-001
SV609	K_Bacteria	P_Firmicutes	C_Clostridia	O_Lachnospirales	F_Lachnospiraceae	G_Roseburia	unclassified_G_Roseburia
SV610	K Bacteria	P Firmicutes	C Clostridia	O Lachnospirales	F Lachnospiraceae	unclassified F Lachnospiraceae	unclassified F Lachnospiraceae
SV611	K Bacteria	P Bacteroidota	C Bacteroidia	O Bacteroidales	F Muribaculaceae	unclassified F Muribaculaceae	unclassified F Muribaculaceae
SV612	K Bacteria	P Bacteroidota	C Bacteroidia	O Bacteroidales	F Prevotellaceae	G Prevotellaceae UCG-001	unclassified G Prevotellaceae UCG-001
SV613	K Bacteria	P Firmicutes	C Clostridia	O Oscillospirales	F Ruminococcaceae	unclassified F Ruminococcaceae	unclassified F Ruminococcaceae
SV614	K Bacteria	P Firmicutes	C Clostridia	O Lachnospirales	F Lachnospiraceae	G [Eubacterium] xylanophilum group	unclassified G [Eubacterium] xylanophilum group
SV615	K_Bacteria	P_Actinobacteriota	C_Coriobacteriia	O_Coriobacteriales	F_Eggerthellaceae	G_Enterorhabdus	unclassified_G_Enterorhabdus
SV616	K Bacteria	P Firmicutes	C Clostridia	O Lachnospirales	F Lachnospiraceae	G Lachnospiraceae UCG-001	unclassified G Lachnospiraceae UCG-001
SV617	K Bacteria	P Firmicutes	C Clostridia	O Lachnospirales	F Lachnospiraceae	unclassified F Lachnospiraceae	unclassified F Lachnospiraceae
SV618	K Bacteria	P Firmicutes	C Clostridia	O Lachnospirales	F Lachnospiraceae	G Marvinbryantia	unclassified G Marvinbryantia
SV619	K Bacteria	P Firmicutes	C Clostridia	O Lachnospirales	F Lachnospiraceae	G Lachnospiraceae UCG-001	unclassified G Lachnospiraceae UCG-001
SV620	K Bacteria	P Firmicutes	C Clostridia	O Lachnospirales	F Lachnospiraceae	G A2	unclassified G A2
SV621	K_Bacteria	P_Firmicutes	C_Clostridia	O_Lachnospirales	F_Lachnospiraceae	unclassified_F_Lachnospiraceae	unclassified_F_Lachnospiraceae
SV622	K Bacteria	P Firmicutes	C Clostridia	O Lachnospirales	F Lachnospiraceae	G Lachnospiraceae NK4A136 group	unclassified G Lachnospiraceae NK4A136 group
SV623	K Bacteria	P Firmicutes	C Clostridia	O Lachnospirales	F Lachnospiraceae	G Roseburia	unclassified G Roseburia
SV624	K Bacteria	P Firmicutes	C Clostridia	O Peptostreptococcales-Tissierellales	F Peptostreptococcaceae	G Romboutsia	unclassified G Romboutsia
SV625	K Bacteria	P Firmicutes	C Clostridia	O Lachnospirales	F Lachnospiraceae	G Tyzzerella	unclassified G Tyzzerella
SV626	K Bacteria	P Firmicutes	C Clostridia	O Lachnospirales	F Lachnospiraceae	G Lachnospiraceae NK4A136 group	unclassified G Lachnospiraceae NK4A136 group
SV627	K Bacteria	P Firmicutes	C Clostridia	O Lachnospirales	F Lachnospiraceae	G Lachnospiraceae UCG-006	unclassified G Lachnospiraceae UCG-006
SV628	K Bacteria	P Firmicutes	C Clostridia	O Lachnospirales	F Lachnospiraceae	unclassified F Lachnospiraceae	unclassified F Lachnospiraceae
SV629	K Bacteria	P Firmicutes	C Clostridia	O Lachnospirales	F Lachnospiraceae	unclassified F Lachnospiraceae	unclassified F Lachnospiraceae
SV630	K Bacteria	P Firmicutes	C Clostridia	O Lachnospirales	F Lachnospiraceae	unclassified F Lachnospiraceae	unclassified F Lachnospiraceae
SV631	K Bacteria	P Firmicutes	C Clostridia	O Lachnospirales	F Lachnospiraceae	G Lachnoclostridium	unclassified G Lachnoclostridium
SV632	K Bacteria	P Bacteroidota	C Bacteroidia	O Bacteroidales	F Rikenellaceae	G Alistipes	unclassified G Alistipes
SV633	K Bacteria	P Firmicutes	C Clostridia	O Oscillospirales	F Ruminococcaceae	unclassified F Ruminococcaceae	unclassified F Ruminococcaceae
SV634	K Bacteria	P Firmicutes	C Clostridia	O Clostridia UCG-014	unclassified O Clostridia UCG-014	unclassified O Clostridia UCG-014	unclassified O Clostridia UCG-014
SV635	K Bacteria	P Bacteroidota	C Bacteroidia	O Bacteroidales	F Prevotellaceae	G Prevotellaceae UCG-001	unclassified G Prevotellaceae UCG-001
SV636	K Bacteria	P Firmicutes	C Clostridia	O Lachnospirales	F Lachnospiraceae	G Roseburia	unclassified G Roseburia
SV637	K Bacteria	P Bacteroidota	C Bacteroidia	O Bacteroidales	F Muribaculaceae	unclassified F Muribaculaceae	unclassified F Muribaculaceae
SV638	K Bacteria	P Firmicutes	C Clostridia	O Oscillospirales	F Oscillospiraceae	G Oscillibacter	unclassified G Oscillibacter
SV639	K Bacteria	P Firmicutes	C Clostridia	O Lachnospirales	F Lachnospiraceae	unclassified F Lachnospiraceae	unclassified F Lachnospiraceae
SV640	K Bacteria	P Bacteroidota	C Bacteroidia	O Bacteroidales	F Muribaculaceae	unclassified F Muribaculaceae	unclassified F Muribaculaceae
SV641	K Bacteria	P Firmicutes	C Clostridia	O Peptostreptococcales-Tissierellales	F Peptostreptococcaceae	G Romboutsia	unclassified G Romboutsia
SV642	K Bacteria	P Firmicutes	C Clostridia	O Lachnospirales	F Lachnospiraceae	unclassified F Lachnospiraceae	unclassified F Lachnospiraceae
SV643	K Bacteria	P Firmicutes	C Clostridia	O Oscillospirales	F Oscillospiraceae	G Colidextribacter	unclassified G Colidextribacter
SV644	K Bacteria	P Proteobacteria	C Gammaproteobacteria	O Enterobacterales	F Enterobacteriaceae	G Enterobacter	unclassified G Enterobacter
SV645	K Bacteria	P Firmicutes	C Clostridia	O Lachnospirales	F Lachnospiraceae	G [Eubacterium] xylanophilum group	unclassified G [Eubacterium] xylanophilum group
SV646	K Bacteria	P Firmicutes	C Clostridia	O Lachnospirales	F Lachnospiraceae	unclassified F Lachnospiraceae	unclassified F Lachnospiraceae
SV647	K Bacteria	P Firmicutes	C Clostridia	O Lachnospirales	F Lachnospiraceae	G Marvinbryantia	unclassified G Marvinbryantia
SV648	K Bacteria	P Firmicutes	C Clostridia	O Oscillospirales	F Ruminococcaceae	unclassified F Ruminococcaceae	unclassified F Ruminococcaceae
SV649	K Bacteria	P Firmicutes	C Clostridia	O Lachnospirales	F Lachnospiraceae	unclassified F Lachnospiraceae	unclassified F Lachnospiraceae
SV650	K Bacteria	P Firmicutes	C Clostridia	O Lachnospirales	F Lachnospiraceae	G Lachnospiraceae UCG-001	unclassified G Lachnospiraceae UCG-001
SV651	K Bacteria	P Firmicutes	C Clostridia	O Clostridia UCG-014	unclassified O Clostridia UCG-014	unclassified O Clostridia UCG-014	unclassified O Clostridia UCG-014
SV652	K Bacteria	P Firmicutes	C Bacilli	O Erysipelotrichales	F Erysipelotrichaceae	G Merdibacter	unclassified G Merdibacter
SV653	K Bacteria	P Firmicutes	C Clostridia	O Peptostreptococcales-Tissierellales	F Anaerovoracaceae	G Family XIII AD3011 group	unclassified G Family XIII AD3011 group
SV654	K Bacteria	P Firmicutes	C Clostridia	O Lachnospirales	F Lachnospiraceae	unclassified F Lachnospiraceae	unclassified F Lachnospiraceae
SV655	K Bacteria	P Firmicutes	C Clostridia	O Peptostreptococcales-Tissierellales	F Peptostreptococcaceae	G Romboutsia	unclassified G Romboutsia
SV656	K Bacteria	P Firmicutes	C Clostridia	O Oscillospirales	F Oscillospiraceae	G Colidextribacter	unclassified G Colidextribacter
SV657	K Bacteria	P Firmicutes	C Clostridia	O Lachnospirales	F Lachnospiraceae	G GCA-900066575	unclassified G GCA-900066575
SV658	K Bacteria	P Firmicutes	C Clostridia	O Oscillospirales	F Oscillospiraceae	G Intestinimonas	unclassified G Intestinimonas
SV659	K Bacteria	P Firmicutes	C Clostridia	O Lachnospirales	F Lachnospiraceae	G Tyzzerella	unclassified G Tyzzerella
SV660	K Bacteria	P Firmicutes	C Clostridia	O Lachnospirales	F Lachnospiraceae	unclassified F Lachnospiraceae	unclassified F Lachnospiraceae
SV661	K Bacteria	P Firmicutes	C Clostridia	O Lachnospirales	F Lachnospiraceae	G Lachnoclostridium	unclassified G Lachnoclostridium
SV662	K_Bacteria	P_Firmicutes	C_Clostridia	O_Oscillospirales	F_Ruminococcaceae	G_UBA1819	unclassified_G_UBA1819
SV663	K Bacteria	P Firmicutes	C Clostridia	O Oscillospirales	F Ruminococcaceae	unclassified F Ruminococcaceae	unclassified F Ruminococcaceae
SV664	K Bacteria	P Firmicutes	C Clostridia	O Lachnospirales	F Lachnospiraceae	G [Ruminococcus] gnavus group	unclassified G [Ruminococcus] gnavus group
SV665	K Bacteria	P Firmicutes	C Clostridia	O Lachnospirales	F Lachnospiraceae	unclassified F Lachnospiraceae	unclassified F Lachnospiraceae
SV666	K Bacteria	P Firmicutes	C Clostridia	O Lachnospirales	F Lachnospiraceae	unclassified F Lachnospiraceae	unclassified F Lachnospiraceae
SV667	K Bacteria	P Firmicutes	C Clostridia	O Lachnospirales	F Lachnospiraceae	G Lachnospiraceae NK4A136 group	unclassified G Lachnospiraceae NK4A136 group
SV668	K_Bacteria	P_Firmicutes	C_Clostridia	O_Clostridia UCG-014	unclassified_O_Clostridia UCG-014	unclassified_O_Clostridia UCG-014	unclassified_O_Clostridia UCG-014
SV669	K Bacteria	P Bacteroidota	C Bacteroidia	O Bacteroidales	F Muribaculaceae	unclassified F Muribaculaceae	unclassified F Muribaculaceae
SV670	K Bacteria	P Firmicutes	C Clostridia	O Lachnospirales	F Lachnospiraceae	G Lachnoclostridium	unclassified G Lachnoclostridium
SV671	K Bacteria	P Firmicutes	C Clostridia	O Clostridia UCG-014	unclassified O Clostridia UCG-014	unclassified O Clostridia UCG-014	unclassified O Clostridia UCG-014
SV672	K Bacteria	P Firmicutes	C Clostridia	O Oscillospirales	F Ruminococcaceae	G Incertae Sedis	unclassified G Incertae Sedis
SV673	K Bacteria	P Firmicutes	C Clostridia	O Lachnospirales	F Lachnospiraceae	unclassified F Lachnospiraceae	unclassified F Lachnospiraceae
SV674	K_Bacteria	P_Firmicutes	C_Clostridia	O_Lachnospirales	F_Lachnospiraceae	unclassified_F_Lachnospiraceae	unclassified_F_Lachnospiraceae
SV675	K Bacteria	P Firmicutes	C Clostridia	O Clostridia UCG-014	unclassified O Clostridia UCG-014	unclassified O Clostridia UCG-014	unclassified O Clostridia UCG-014
SV676	K Bacteria	P Firmicutes	C Bacilli	O Erysipelotrichales	F Erysipelotrichaceae	G Turicibacter	unclassified G Turicibacter
SV677	K Bacteria	P Firmicutes	C Clostridia	O Lachnospirales	F Lachnospiraceae	G Frisingicoccus	S caecimuris
SV678	K Bacteria	P Firmicutes	C Clostridia	O Lachnospirales	F Lachnospiraceae	unclassified F Lachnospiraceae	unclassified F Lachnospiraceae
SV679	K Bacteria	P Bacteroidota	C Bacteroidia	O Bacteroidales	F Bacteroidaceae	G Bacteroides	unclassified G Bacteroides
SV680	K_Bacteria	P_Firmicutes	C_Clostridia	O_Lachnospirales	F_Lachnospiraceae	G_GCA-900066575	unclassified_G_GCA-900066575
SV681	K Bacteria	P Firmicutes	C Clostridia	O Clostridia UCG-014	unclassified O Clostridia UCG-014	unclassified O Clostridia UCG-014	unclassified O Clostridia UCG-014
SV682	K Bacteria	P Firmicutes	C Bacilli	O Lactobacillales	F Lactobacillaceae	G Lactobacillus	unclassified G Lactobacillus
SV683	K Bacteria	P Firmicutes	C Clostridia	O Oscillospirales	F Oscillospiraceae	G UCG-003	unclassified G UCG-003
SV684	K Bacteria	P Firmicutes	C Clostridia	O Lachnospirales	F Lachnospiraceae	G [Eubacterium] ruminantium group	unclassified G [Eubacterium] ruminantium group
SV685	K Bacteria	P Firmicutes	C Clostridia	O Lachnospirales	F Lachnospiraceae	unclassified F Lachnospiraceae	unclassified F Lachnospiraceae
SV686	K Bacteria	P Firmicutes	C Clostridia	O Lachnospirales	F Lachnospiraceae	unclassified F Lachnospiraceae	unclassified F Lachnospiraceae
SV687	K Bacteria	P Firmicutes	C Clostridia	O Oscillospirales	F Oscillospiraceae	G Colidextribacter	unclassified G Colidextribacter
SV688	K Bacteria	P Firmicutes	C Clostridia	O Lachnospirales	F Lachnospiraceae	unclassified F Lachnospiraceae	unclassified F Lachnospiraceae

SV689	K_Bacteria	P_Firmicutes	C_Clostridia	O_Oscillospirales	F_Ruminococcaceae	G_Anaerotruncus	unclassified_G_Anaerotruncus
SV690	K_Bacteria	P_Firmicutes	C_Clostridia	O_Lachnospirales	F_Lachnospiraceae	unclassified_F_Lachnospiraceae	unclassified_F_Lachnospiraceae
SV691	K_Bacteria	P_Bacteroidota	C_Bacteroidia	O_Bacteroidales	F_Muribaculaceae	unclassified_F_Muribaculaceae	unclassified_F_Muribaculaceae
SV692	K_Bacteria	P_Firmicutes	C_Clostridia	O_Clostridia UCG-014	unclassified_O_Clostridia UCG-014	unclassified_O_Clostridia UCG-014	unclassified_O_Clostridia UCG-014
SV693	K_Bacteria	P_Firmicutes	C_Clostridia	O_Lachnospirales	F_Lachnospiraceae	G_[Ruminococcus] gauvreauii group	unclassified_G_[Ruminococcus] gauvreauii group
SV694	K_Bacteria	P_Proteobacteria	C_Gammaproteobacteria	O_Pseudomonadales	F_Pseudomonadaceae	G_Pseudomonas	unclassified_G_Pseudomonas
SV695	K_Bacteria	P_Firmicutes	C_Clostridia	O_Lachnospirales	F_Lachnospiraceae	unclassified_F_Lachnospiraceae	unclassified_F_Lachnospiraceae
SV696	K_Bacteria	P_Firmicutes	C_Clostridia	O_Oscillospirales	F_Oscillospiraceae	unclassified_F_Oscillospiraceae	unclassified_F_Oscillospiraceae
SV697	K_Bacteria	P_Firmicutes	C_Clostridia	O_Lachnospirales	F_Lachnospiraceae	unclassified_F_Lachnospiraceae	unclassified_F_Lachnospiraceae
SV698	K_Bacteria	P_Firmicutes	C_Clostridia	O_Oscillospirales	F_Ruminococcaceae	G_UBA1819	unclassified_G_UBA1819
SV699	K_Bacteria	P_Firmicutes	C_Clostridia	O_Lachnospirales	F_Lachnospiraceae	G_ASF356	unclassified_G_ASF356
SV700	K_Bacteria	P_Firmicutes	C_Clostridia	O_Oscillospirales	F_Oscillospiraceae	unclassified_F_Oscillospiraceae	unclassified_F_Oscillospiraceae
SV701	K_Bacteria	P_Firmicutes	C_Clostridia	O_Clostridia UCG-014	unclassified_O_Clostridia UCG-014	unclassified_O_Clostridia UCG-014	unclassified_O_Clostridia UCG-014
SV702	K_Bacteria	P_Firmicutes	C_Clostridia	O_Lachnospirales	F_Lachnospiraceae	G_Lachnospiraceae FCS020 group	unclassified_G_Lachnospiraceae FCS020 group
SV703	K_Bacteria	P_Bacteroidota	C_Bacteroidia	O_Bacteroidales	F_Muribaculaceae	unclassified_F_Muribaculaceae	unclassified_F_Muribaculaceae
SV704	K_Bacteria	P_Firmicutes	C_Bacilli	O_Lactobacillales	F_Lactobacillaceae	G_Lactobacillus	unclassified_G_Lactobacillus
SV705	K_Bacteria	P_Firmicutes	C_Clostridia	O_Lachnospirales	F_Lachnospiraceae	G_Lachnoclostridium	unclassified_G_Lachnoclostridium
SV706	K_Bacteria	P_Firmicutes	C_Clostridia	O_Oscillospirales	F_Butyricicoccaceae	G_Butyricicoccus	unclassified_G_Butyricicoccus
SV707	K_Bacteria	P_Firmicutes	C_Clostridia	O_Oscillospirales	F_Oscillospiraceae	unclassified_F_Oscillospiraceae	unclassified_F_Oscillospiraceae
SV708	K_Bacteria	P_Firmicutes	C_Clostridia	O_Oscillospirales	F_Ruminococcaceae	G_Ruminococcus	unclassified_G_Ruminococcus
SV709	K_Bacteria	P_Firmicutes	C_Clostridia	O_Lachnospirales	F_Lachnospiraceae	G_Lachnoclostridium	unclassified_G_Lachnoclostridium
SV710	K_Bacteria	P_Firmicutes	C_Clostridia	O_Oscillospirales	F_Ruminococcaceae	G_Paludicola	unclassified_G_Paludicola
SV711	K_Bacteria	P_Firmicutes	C_Clostridia	O_Oscillospirales	F_Oscillospiraceae	G_Oscillibacter	unclassified_G_Oscillibacter
SV712	K_Bacteria	P_Firmicutes	C_Clostridia	O_Oscillospirales	F_Butyricicoccaceae	G_Butyricicoccus	unclassified_G_Butyricicoccus
SV713	K_Bacteria	P_Firmicutes	C_Clostridia	O_Lachnospirales	F_Lachnospiraceae	G_Lachnospiraceae NK4A136 group	unclassified_G_Lachnospiraceae NK4A136 group
SV714	K_Bacteria	P_Firmicutes	C_Clostridia	O_Lachnospirales	F_Lachnospiraceae	unclassified_F_Lachnospiraceae	unclassified_F_Lachnospiraceae
SV715	K_Bacteria	P_Firmicutes	C_Clostridia	O_Lachnospirales	F_Lachnospiraceae	unclassified_F_Lachnospiraceae	unclassified_F_Lachnospiraceae
SV716	K_Bacteria	P_Firmicutes	C_Bacilli	O_Erysipelotrichales	F_Erysipelotrichaceae	G_Holdemania	S_massiliensis
SV717	K_Bacteria	P_Firmicutes	C_Clostridia	O_Lachnospirales	F_Lachnospiraceae	G_Lachnospiraceae NK4A136 group	unclassified_G_Lachnospiraceae NK4A136 group
SV718	K_Bacteria	P_Firmicutes	C_Clostridia	O_Oscillospirales	F_Oscillospiraceae	unclassified_F_Oscillospiraceae	unclassified_F_Oscillospiraceae
SV719	K_Bacteria	P_Firmicutes	C_Clostridia	O_Lachnospirales	F_Lachnospiraceae	G_Lachnospiraceae UCG-001	unclassified_G_Lachnospiraceae UCG-001
SV720	K_Bacteria	P_Firmicutes	C_Clostridia	O_Clostridia UCG-014	unclassified_O_Clostridia UCG-014	unclassified_O_Clostridia UCG-014	unclassified_O_Clostridia UCG-014
SV721	K_Bacteria	P_Bacteroidota	C_Bacteroidia	O_Bacteroidales	F_Muribaculaceae	unclassified_F_Muribaculaceae	unclassified_F_Muribaculaceae
SV722	K_Bacteria	P_Firmicutes	C_Clostridia	O_Lachnospirales	F_Lachnospiraceae	unclassified_F_Lachnospiraceae	unclassified_F_Lachnospiraceae
SV723	K_Bacteria	P_Firmicutes	C_Clostridia	O_Lachnospirales	F_Lachnospiraceae	G_Lachnoclostridium	unclassified_G_Lachnoclostridium
SV724	K_Bacteria	P_Firmicutes	C_Clostridia	O_Lachnospirales	F_Lachnospiraceae	unclassified_F_Lachnospiraceae	unclassified_F_Lachnospiraceae
SV725	K_Bacteria	P_Firmicutes	C_Clostridia	O_Lachnospirales	F_Lachnospiraceae	G_Lachnospiraceae NK4A136 group	unclassified_G_Lachnospiraceae NK4A136 group
SV726	K_Bacteria	P_Firmicutes	C_Clostridia	O_Lachnospirales	F_Lachnospiraceae	G_Blautia	unclassified_G_Blautia
SV727	K_Bacteria	P_Firmicutes	C_Clostridia	O_Clostridia UCG-014	unclassified_O_Clostridia UCG-014	unclassified_O_Clostridia UCG-014	unclassified_O_Clostridia UCG-014
SV728	K_Bacteria	P_Bacteroidota	C_Bacteroidia	O_Bacteroidales	F_Muribaculaceae	unclassified_F_Muribaculaceae	unclassified_F_Muribaculaceae
SV729	K_Bacteria	P_Firmicutes	C_Clostridia	O_Lachnospirales	F_Lachnospiraceae	G_Lachnospiraceae NK4A136 group	unclassified_G_Lachnospiraceae NK4A136 group
SV730	K_Bacteria	P_Firmicutes	C_Clostridia	O_Lachnospirales	F_Lachnospiraceae	G_Roseburia	unclassified_G_Roseburia
SV731	K_Bacteria	P_Firmicutes	C_Clostridia	O_Oscillospirales	F_Ruminococcaceae	G_Incertae Sedis	unclassified_G_Incertae Sedis
SV732	K_Bacteria	P_Firmicutes	C_Clostridia	O_Lachnospirales	F_Lachnospiraceae	unclassified_F_Lachnospiraceae	unclassified_F_Lachnospiraceae
SV733	K_Bacteria	P_Firmicutes	C_Clostridia	O_Lachnospirales	F_Lachnospiraceae	G_Roseburia	unclassified_G_Roseburia
SV734	K_Bacteria	P_Firmicutes	C_Clostridia	O_Lachnospirales	F_Lachnospiraceae	G_Lachnospiraceae NK4A136 group	unclassified_G_Lachnospiraceae NK4A136 group
SV735	K_Bacteria	P_Firmicutes	C_Clostridia	O_Lachnospirales	F_Lachnospiraceae	unclassified_F_Lachnospiraceae	unclassified_F_Lachnospiraceae
SV736	K_Bacteria	P_Firmicutes	C_Clostridia	O_Lachnospirales	F_Lachnospiraceae	G_Marvinbryantia	unclassified_G_Marvinbryantia
SV737	K_Bacteria	P_Firmicutes	C_Clostridia	O_Lachnospirales	F_Lachnospiraceae	G_Marvinbryantia	unclassified_G_Marvinbryantia
SV738	K_Bacteria	P_Firmicutes	C_Clostridia	O_Oscillospirales	F_Oscillospiraceae	unclassified_F_Oscillospiraceae	unclassified_F_Oscillospiraceae
SV739	K_Bacteria	P_Firmicutes	C_Clostridia	O_Lachnospirales	F_Lachnospiraceae	G_Lachnospiraceae NK4A136 group	unclassified_G_Lachnospiraceae NK4A136 group
SV740	K_Bacteria	P_Firmicutes	C_Clostridia	O_Lachnospirales	F_Lachnospiraceae	unclassified_F_Lachnospiraceae	unclassified_F_Lachnospiraceae
SV741	K_Bacteria	P_Firmicutes	C_Clostridia	O_Lachnospirales	F_Lachnospiraceae	unclassified_F_Lachnospiraceae	unclassified_F_Lachnospiraceae
SV742	K_Bacteria	P_Firmicutes	C_Clostridia	O_Clostridia UCG-014	unclassified_O_Clostridia UCG-014	unclassified_O_Clostridia UCG-014	unclassified_O_Clostridia UCG-014
SV743	K_Bacteria	P_Firmicutes	C_Clostridia	O_Oscillospirales	F_Oscillospiraceae	G_Intestinimonas	unclassified_G_Intestinimonas
SV744	K_Bacteria	P_Firmicutes	C_Clostridia	O_Lachnospirales	F_Lachnospiraceae	G_Lachnoclostridium	unclassified_G_Lachnoclostridium
SV745	K_Bacteria	P_Firmicutes	C_Clostridia	O_Oscillospirales	F_Oscillospiraceae	unclassified_F_Oscillospiraceae	unclassified_F_Oscillospiraceae
SV746	K_Bacteria	P_Firmicutes	C_Clostridia	O_Lachnospirales	F_Lachnospiraceae	G_Lachnospiraceae NK4A136 group	unclassified_G_Lachnospiraceae NK4A136 group
SV747	K_Bacteria	P_Firmicutes	C_Clostridia	O_Clostridiales	F_Clostridiaceae	G_Clostridium sensu stricto 1	unclassified_G_Clostridium sensu stricto 1
SV748	K_Bacteria	P_Firmicutes	C_Clostridia	O_Oscillospirales	F_Ruminococcaceae	G_UBA1819	unclassified_G_UBA1819
SV749	K_Bacteria	P_Proteobacteria	C_Gammaproteobacteria	O_Burkholderiales	F_Burkholderiaceae	G_Ralstonia	unclassified_G_Ralstonia
SV750	K_Bacteria	P_Bacteroidota	C_Bacteroidia	O_Bacteroidales	F_Muribaculaceae	unclassified_F_Muribaculaceae	unclassified_F_Muribaculaceae
SV751	K_Bacteria	P_Firmicutes	C_Clostridia	O_Lachnospirales	F_Lachnospiraceae	G_Lachnospiraceae UCG-001	unclassified_G_Lachnospiraceae UCG-001
SV752	K_Bacteria	P_Bacteroidota	C_Bacteroidia	O_Bacteroidales	F_Muribaculaceae	unclassified_F_Muribaculaceae	unclassified_F_Muribaculaceae
SV753	K_Bacteria	P_Firmicutes	C_Clostridia	O_Oscillospirales	F_Ruminococcaceae	G_Ruminococcus	unclassified_G_Ruminococcus
SV754	K_Bacteria	P_Firmicutes	C_Clostridia	O_Lachnospirales	F_Lachnospiraceae	G_Lachnospiraceae UCG-001	unclassified_G_Lachnospiraceae UCG-001
SV755	K_Bacteria	P_Firmicutes	C_Clostridia	O_Lachnospirales	F_Lachnospiraceae	G_Tyzzerella	unclassified_G_Tyzzerella
SV756	K_Bacteria	P_Firmicutes	C_Clostridia	O_Lachnospirales	F_Defluviitaleaceae	G_Defluviitaleaceae UCG-011	unclassified_G_Defluviitaleaceae UCG-011
SV757	K_Bacteria	P_Firmicutes	C_Clostridia	O_Lachnospirales	F_Lachnospiraceae	G_Lachnospiraceae NK4A136 group	unclassified_G_Lachnospiraceae NK4A136 group
SV758	K_Bacteria	P_Firmicutes	C_Bacilli	O_Lactobacillales	F_Lactobacillaceae	G_HT002	unclassified_G_HT002
SV759	K_Bacteria	P_Firmicutes	C_Clostridia	O_Oscillospirales	F_Oscillospiraceae	unclassified_F_Oscillospiraceae	unclassified_F_Oscillospiraceae
SV760	K_Bacteria	P_Firmicutes	C_Clostridia	O_Lachnospirales	F_Lachnospiraceae	G_Lachnoclostridium	unclassified_G_Lachnoclostridium
SV761	K_Bacteria	P_Firmicutes	C_Clostridia	O_Lachnospirales	F_Lachnospiraceae	G_Lachnospiraceae NK4A136 group	S_bacterium
SV762	K_Bacteria	P_Firmicutes	C_Clostridia	O_Oscillospirales	F_Ruminococcaceae	unclassified_F_Ruminococcaceae	unclassified_F_Ruminococcaceae
SV763	K_Bacteria	P_Firmicutes	C_Clostridia	O_Lachnospirales	F_Lachnospiraceae	G_Lachnospiraceae NK4A136 group	unclassified_G_Lachnospiraceae NK4A136 group
SV764	K_Bacteria	P_Firmicutes	C_Clostridia	O_Lachnospirales	F_Lachnospiraceae	G_GCA-900066575	unclassified_G_GCA-900066575
SV765	K_Bacteria	P_Firmicutes	C_Clostridia	O_Lachnospirales	F_Lachnospiraceae	G_Lachnospiraceae FCS020 group	unclassified_G_Lachnospiraceae FCS020 group
SV766	K_Bacteria	P_Firmicutes	C_Clostridia	O_Lachnospirales	F_Lachnospiraceae	G_Lachnospiraceae UCG-001	unclassified_G_Lachnospiraceae UCG-001
SV767	K_Bacteria	P_Firmicutes	C_Clostridia	O_Oscillospirales	F_Oscillospiraceae	G_Colidextribacter	unclassified_G_Colidextribacter
SV768	K_Bacteria	P_Patescibacteria	C_Saccharimonadia	O_Saccharimonadales	F_Saccharimonadaceae	G_Candidatus Saccharimonas	unclassified_G_Candidatus Saccharimonas
SV769	K_Bacteria	P_Firmicutes	C_Clostridia	O_Lachnospirales	F_Lachnospiraceae	G_Lachnospiraceae NK4A136 group	unclassified_G_Lachnospiraceae NK4A136 group
SV770	K_Bacteria	P_Firmicutes	C_Clostridia	O_Lachnospirales	F_Lachnospiraceae	G_Lachnospiraceae NK4A136 group	unclassified_G_Lachnospiraceae NK4A136 group
SV771	K_Bacteria	P_Firmicutes	C_Clostridia	O_Lachnospirales	F_Lachnospiraceae	G_Lachnospiraceae NK4A136 group	unclassified_G_Lachnospiraceae NK4A136 group
SV772	K_Bacteria	P_Firmicutes	C_Clostridia	O_Lachnospirales	F_Lachnospiraceae	G_Lachnoclostridium	unclassified_G_Lachnoclostridium
SV773	K_Bacteria	P_Firmicutes	C_Clostridia	O_Peptostreptococcales-Tissierellales	F_Anaerovoracaceae	G_Anaerovorax	unclassified_G_Anaerovorax
SV774	K_Bacteria	P_Firmicutes	C_Bacilli	O_Staphylococcales	F_Staphylococcaceae	G_Staphylococcus	S_aureus
SV775	K_Bacteria	P_Firmicutes	C_Clostridia	O_Lachnospirales	F_Lachnospiraceae	G_Roseburia	unclassified_G_Roseburia
SV776	K_Bacteria	P_Firmicutes	C_Clostridia	O_Lachnospirales	F_Lachnospiraceae	unclassified_F_Lachnospiraceae	unclassified_F_Lachnospiraceae
SV777	K_Bacteria	P_Firmicutes	C_Clostridia	O_Clostridia UCG-014	unclassified_O_Clostridia UCG-014	unclassified_O_Clostridia UCG-014	unclassified_O_Clostridia UCG-014
SV778	K_Bacteria	P_Firmicutes	C_Clostridia	O_Lachnospirales	F_Lachnospiraceae	G_Marvinbryantia	unclassified_G_Marvinbryantia
SV779	K_Bacteria	P_Firmicutes	C_Clostridia	O_Clostridia UCG-014	unclassified_O_Clostridia UCG-014	unclassified_O_Clostridia UCG-014	unclassified_O_Clostridia UCG-014
SV780	K_Bacteria	P_Firmicutes	C_Clostridia	O_Lachnospirales	F_Lachnospiraceae	G_Lachnospiraceae NK4A136 group	unclassified_G_Lachnospiraceae NK4A136 group
SV781	K_Bacteria	P_Firmicutes	C_Clostridia	O_Lachnospirales	F_Lachnospiraceae	G_Lachnospiraceae UCG-001	unclassified_G_Lachnospiraceae UCG-001
SV782	K_Bacteria	P_Firmicutes	C_Clostridia	O_Clostridia UCG-014	unclassified_O_Clostridia UCG-014	unclassified_O_Clostridia UCG-014	unclassified_O_Clostridia UCG-014
SV783	K_Bacteria	P_Bacteroidota	C_Bacteroidia	O_Bacteroidales	F_Rikenellaceae	G_Alistipes	unclassified_G_Alistipes
SV784	K_Bacteria	P_Firmicutes	C_Clostridia	O_Lachnospirales	F_Lachnospiraceae	G_Marvinbryantia	unclassified_G_Marvinbryantia
SV785	K_Bacteria	P_Firmicutes	C_Clostridia	O_Lachnospirales	F_Lachnospiraceae	unclassified_F_Lachnospiraceae	unclassified_F_Lachnospiraceae
SV786	K_Bacteria	P_Firmicutes	C_Clostridia	O_Clostridia UCG-014	unclassified_O_Clostridia UCG-014	unclassified_O_Clostridia UCG-014	unclassified_O_Clostridia UCG-014
SV787	K_Bacteria	P_Firmicutes	C_Clostridia	O_Lachnospirales	F_Lachnospiraceae	unclassified_F_Lachnospiraceae	unclassified_F_Lachnospiraceae
SV788	K_Bacteria	P_Firmicutes	C_Clostridia	O_Lachnospirales	F_Lachnospiraceae	G_Roseburia	unclassified_G_Roseburia
SV789	K_Bacteria	P_Bacteroidota	C_Bacteroidia	O_Bacteroidales	F_Muribaculaceae	unclassified_F_Muribaculaceae	unclassified_F_Muribaculaceae
SV790	K_Bacteria	P_Firmicutes	C_Clostridia	O_Lachnospirales	F_Lachnospiraceae	G_Lachnoclostridium	unclassified_G_Lachnoclostridium
SV791	K_Bacteria	P_Firmicutes	C_Clostridia	O_Lachnospirales	F_Lachnospiraceae	unclassified_F_Lachnospiraceae	unclassified_F_Lachnospiraceae
SV792	K_Bacteria	P_Firmicutes	C_Clostridia	O_Oscillospirales	F_Oscillospiraceae	G_Intestinimonas	unclassified_G_Intestinimonas
SV793	K_Bacteria	P_Firmicutes	C_Clostridia	O_Lachnospirales	F_Lachnospiraceae	G_Lachnospiraceae NK4A136 group	unclassified_G_Lachnospiraceae NK4A136 group
SV794	K_Bacteria	P_Firmicutes	C_Clostridia	O_Oscillospirales	F_Ruminococcaceae	G_Incertae Sedis	unclassified_G_Incertae Sedis
SV795	K_Bacteria	P_Patescibacteria	C_Saccharimonadia	O_Saccharimonadales	F_Saccharimonadaceae	G_Candidatus Saccharimonas	unclassified_G_Candidatus Saccharimonas
SV796	K_Bacteria	P_Firmicutes	C_Clostridia	O_Oscillospirales	F_Ruminococcaceae	G_Negativibacillus	unclassified_G_Negativibacillus
SV797	K_Bacteria	P_Firmicutes	C_Clostridia	O_Lachnospirales	F_Lachnospiraceae	G_Tuzzerella	unclassified_G_Tuzzerella
SV798	K_Bacteria	P_Firmicutes	C_Clostridia	O_Oscillospirales	F_Ruminococcaceae	G_Paludicola	unclassified_G_Paludicola
SV799	K_Bacteria	P_Firmicutes	C_Clostridia	O_Lachnospirales	F_Lachnospiraceae	G_Lachnospiraceae NK4A136 group	unclassified_G_Lachnospiraceae NK4A136 group
SV800	K_Bacteria	P_Firmicutes	C_Clostridia	O_Lachnospirales	F_Lachnospiraceae	G_GCA-900066575	unclassified_G_GCA-900066575
SV801	K_Bacteria	P_Firmicutes	C_Clostridia	O_Lachnospirales	F_Lachnospiraceae	G_Roseburia	unclassified_G_Roseburia
SV802	K_Bacteria	P_Firmicutes	C_Clostridia	O_Oscillospirales	F_Ruminococcaceae	G_Ruminococcus	unclassified_G_Ruminococcus
SV803	K_Bacteria	P_Firmicutes	C_Bacilli	O_Erysipelotrichales	F_Erysipelotrichaceae	G_Turicibacter	unclassified_G_Turicibacter
SV804	K_Bacteria	P_Firmicutes	C_Clostridia	O_Oscillospirales	F_Ruminococcaceae	unclassified_F_Ruminococcaceae	unclassified_F_Ruminococcaceae
SV805	K_Bacteria	P_Firmicutes	C_Clostridia	O_Lachnospirales	F_Lachnospiraceae	unclassified_F_Lachnospiraceae	unclassified_F_Lachnospiraceae
SV806	K_Bacteria	P_Firmicutes	C_Clostridia	O_Lachnospirales	F_Lachnospiraceae	G_[Ruminococcus] gnavus group	unclassified_G_[Ruminococcus] gnavus group
SV807	K_Bacteria	P_Firmicutes	C_Clostridia	O_Oscillospirales	F_Oscillospiraceae	G_Colidextribacter	unclassified_G_Colidextribacter
SV808	K_Bacteria	P_Firmicutes	C_Clostridia	O_Lachnospirales	F_Lachnospiraceae	G_Marvinbryantia	unclassified_G_Marvinbryantia
SV809	K_Bacteria	P_Firmicutes	C_Clostridia	O_Oscillospirales	F_Ruminococcaceae	G_Ruminococcus	unclassified_G_Ruminococcus
SV810	K_Bacteria	P_Verrucomicrobiota	C_Verrucomicrobiae	O_Verrucomicrobiales	F_Akkermansiaceae	G_Akkermansia	unclassified_G_Akkermansia
SV811	K_Bacteria	P_Firmicutes	C_Clostridia	O_Clostridia UCG-014	unclassified_O_Clostridia UCG-014	unclassified_O_Clostridia UCG-014	unclassified_O_Clostridia UCG-014
SV812	K_Bacteria	P_Actinobacteriota	C_Coriobacteriia	O_Coriobacteriales	F_Eggerthellaceae	G_Enterorhabdus	unclassified_G_Enterorhabdus
SV813	K_Bacteria	P_Firmicutes	C_Clostridia	O_Lachnospirales	F_Lachnospiraceae	G_Lachnospiraceae AC2044 group	unclassified_G_Lachnospiraceae AC2044 group
SV814	K_Bacteria	P_Firmicutes	C_Clostridia	O_Lachnospirales	F_Lachnospiraceae	G_Lachnospiraceae NK4A136 group	S_bacterium
SV815	K_Bacteria	P_Firmicutes	C_Clostridia	O_Lachnospirales	F_Lachnospiraceae	G_Lachnoclostridium	unclassified_G_Lachnoclostridium
SV816	K_Bacteria	P_Firmicutes	C_Clostridia	O_Lachnospirales	F_Lachnospiraceae	unclassified_F_Lachnospiraceae	unclassified_F_Lachnospiraceae
SV817	K_Bacteria	P_Cyanobacteria	C_Vampirivibrionia	O_Gastranaerophilales	unclassified_O_Gastranaerophilales	unclassified_O_Gastranaerophilales	unclassified_O_Gastranaerophilales
SV818	K_Bacteria	P_Firmicutes	C_Clostridia	O_Oscillospirales	F_Oscillospiraceae	unclassified_F_Oscillospiraceae	unclassified_F_Oscillospiraceae
SV819	K_Bacteria	P_Firmicutes	C_Clostridia	O_Lachnospirales	F_Lachnospiraceae	unclassified_F_Lachnospiraceae	unclassified_F_Lachnospiraceae
SV820	K_Bacteria	P_Firmicutes	C_Clostridia	O_Lachnospirales	F_Lachnospiraceae	G_Lachnospiraceae NK4A136 group	unclassified_G_Lachnospiraceae NK4A136 group
SV821	K_Bacteria	P_Firmicutes	C_Clostridia	O_Lachnospirales	F_Lachnospiraceae	G_GCA-900066575	unclassified_G_GCA-900066575
SV822	K_Bacteria	P_Firmicutes	C_Bacilli	O_Lactobacillales	F_Lactobacillaceae	G_Lactobacillus	unclassified_G_Lactobacillus
SV823	K_Bacteria	P_Firmicutes	C_Clostridia	O_Lachnospirales	F_Lachnospiraceae	unclassified_F_Lachnospiraceae	unclassified_F_Lachnospiraceae
SV824	K_Bacteria	P_Bacteroidota	C_Bacteroidia	O_Bacteroidales	F_Marinifilaceae	G_Odoribacter	unclassified_G_Odoribacter
SV825	K_Bacteria	P_Firmicutes	C_Clostridia	O_Oscillospirales	F_Ruminococcaceae	G_Anaerotruncus	unclassified_G_Anaerotruncus
SV826	K_Bacteria	P_Firmicutes	C_Clostridia	O_Lachnospirales	F_Lachnospiraceae	G_Lachnospiraceae NK4A136 group	unclassified_G_Lachnospiraceae NK4A136 group

SV827	K_Bacteria	P_Firmicutes	C_Clostridia	O_Lachnospirales	F_Lachnospiraceae	unclassified_F_Lachnospiraceae	unclassified_F_Lachnospiraceae
SV828	K_Bacteria	P_Bacteroidota	C_Bacteroidia	O_Bacteroidales	F_Bacteroidaceae	G_Bacteroides	unclassified_G_Bacteroides
SV829	K_Bacteria	P_Firmicutes	C_Clostridia	O_Oscillospirales	F_Oscillospiraceae	G_Colidextribacter	unclassified_G_Colidextribacter
SV830	K_Bacteria	P_Firmicutes	C_Clostridia	O_Lachnospirales	F_Lachnospiraceae	G_ASF356	unclassified_G_ASF356
SV831	K_Bacteria	P_Firmicutes	C_Clostridia	O_Lachnospirales	F_Lachnospiraceae	G_Lachnoclostridium	unclassified_G_Lachnoclostridium
SV832	K_Bacteria	P_Firmicutes	C_Clostridia	O_Oscillospirales	F_Oscillospiraceae	unclassified_F_Oscillospiraceae	unclassified_F_Oscillospiraceae
SV833	K_Bacteria	P_Firmicutes	C_Clostridia	O_Lachnospirales	F_Lachnospiraceae	G_ASF356	unclassified_G_ASF356
SV834	K_Bacteria	P_Firmicutes	C_Clostridia	O_Lachnospirales	F_Lachnospiraceae	unclassified_F_Lachnospiraceae	unclassified_F_Lachnospiraceae
SV835	K_Bacteria	P_Bacteroidota	C_Bacteroidia	O_Bacteroidales	F_Muribaculaceae	unclassified_F_Muribaculaceae	unclassified_F_Muribaculaceae
SV836	K_Bacteria	P_Firmicutes	C_Clostridia	O_Lachnospirales	F_Lachnospiraceae	G_Lachnospiraceae NK4A136 group	unclassified_G_Lachnospiraceae NK4A136 group
SV837	K_Bacteria	P_Firmicutes	C_Clostridia	O_Lachnospirales	F_Lachnospiraceae	G_Blautia	unclassified_G_Blautia
SV838	K_Bacteria	P_Firmicutes	C_Clostridia	O_Clostridia UCG-014	unclassified_O_Clostridia UCG-014	unclassified_O_Clostridia UCG-014	unclassified_O_Clostridia UCG-014
SV839	K_Bacteria	P_Firmicutes	C_Clostridia	O_Lachnospirales	F_Lachnospiraceae	G_Lachnospiraceae NK4A136 group	unclassified_G_Lachnospiraceae NK4A136 group
SV840	K_Bacteria	P_Firmicutes	C_Clostridia	O_Clostridiales	F_Clostridiaceae	G_Clostridium sensu stricto 1	unclassified_G_Clostridium sensu stricto 1
SV841	K_Bacteria	P_Firmicutes	C_Clostridia	O_Lachnospirales	F_Lachnospiraceae	G_Lachnoclostridium	unclassified_G_Lachnoclostridium
SV842	K_Bacteria	P_Firmicutes	C_Clostridia	O_Lachnospirales	F_Lachnospiraceae	G_Lachnospiraceae NK4A136 group	unclassified_G_Lachnospiraceae NK4A136 group
SV843	K_Bacteria	P_Firmicutes	C_Clostridia	O_Lachnospirales	F_Lachnospiraceae	unclassified_F_Lachnospiraceae	unclassified_F_Lachnospiraceae
SV844	K_Bacteria	P_Firmicutes	C_Clostridia	O_Lachnospirales	F_Lachnospiraceae	G_Lachnospiraceae UCG-001	unclassified_G_Lachnospiraceae UCG-001
SV845	K_Bacteria	P_Firmicutes	C_Bacilli	O_Lactobacillales	F_Lactobacillaceae	unclassified_F_Lactobacillaceae	unclassified_F_Lactobacillaceae
SV846	K_Bacteria	P_Firmicutes	C_Clostridia	O_Lachnospirales	F_Lachnospiraceae	G_Lachnospiraceae UCG-001	unclassified_G_Lachnospiraceae UCG-001
SV847	K_Bacteria	P_Firmicutes	C_Clostridia	O_Christensenellales	F_Christensenellaceae	G_Christensenellaceae R-7 group	unclassified_G_Christensenellaceae R-7 group
SV848	K_Bacteria	P_Firmicutes	C_Clostridia	O_Lachnospirales	F_Lachnospiraceae	G_Lachnospiraceae UCG-001	unclassified_G_Lachnospiraceae UCG-001
SV849	K_Bacteria	P_Firmicutes	C_Clostridia	O_Lachnospirales	F_Defluviitaleaceae	G_Defluviitaleaceae UCG-011	unclassified_G_Defluviitaleaceae UCG-011
SV850	K_Bacteria	P_Firmicutes	C_Clostridia	O_Lachnospirales	F_Lachnospiraceae	unclassified_F_Lachnospiraceae	unclassified_F_Lachnospiraceae
SV851	K_Bacteria	P_Firmicutes	C_Clostridia	O_Lachnospirales	F_Lachnospiraceae	G_Lachnoclostridium	unclassified_G_Lachnoclostridium
SV852	K_Bacteria	P_Firmicutes	C_Clostridia	O_Lachnospirales	F_Lachnospiraceae	G_GCA-900066575	unclassified_G_GCA-900066575
SV853	K_Bacteria	P_Firmicutes	C_Clostridia	O_Lachnospirales	F_Lachnospiraceae	G_Lachnospiraceae NK4A136 group	unclassified_G_Lachnospiraceae NK4A136 group
SV854	K_Bacteria	P_Firmicutes	C_Clostridia	O_Lachnospirales	F_Lachnospiraceae	G_Lachnospiraceae UCG-006	unclassified_G_Lachnospiraceae UCG-006
SV855	K_Bacteria	P_Firmicutes	C_Clostridia	O_Oscillospirales	F_Oscillospiraceae	unclassified_F_Oscillospiraceae	unclassified_F_Oscillospiraceae
SV856	K_Bacteria	P_Firmicutes	C_Clostridia	O_Oscillospirales	F_Ruminococcaceae	G_Ruminococcus	unclassified_G_Ruminococcus
SV857	K_Bacteria	P_Firmicutes	C_Clostridia	O_Lachnospirales	F_Lachnospiraceae	G_Lachnospiraceae UCG-001	unclassified_G_Lachnospiraceae UCG-001
SV858	K_Bacteria	P_Firmicutes	C_Clostridia	O_Lachnospirales	F_Lachnospiraceae	G_Lachnospiraceae UCG-001	unclassified_G_Lachnospiraceae UCG-001
SV859	K_Bacteria	P_Firmicutes	C_Clostridia	O_Oscillospirales	F_Ruminococcaceae	G_Incertae Sedis	unclassified_G_Incertae Sedis
SV860	K_Bacteria	P_Firmicutes	C_Clostridia	O_Lachnospirales	F_Lachnospiraceae	G_Lachnospiraceae UCG-006	unclassified_G_Lachnospiraceae UCG-006
SV861	K_Bacteria	P_Bacteroidota	C_Bacteroidia	O_Bacteroidales	F_Rikenellaceae	G_Alistipes	unclassified_G_Alistipes
SV862	K_Bacteria	P_Firmicutes	C_Clostridia	O_Lachnospirales	F_Lachnospiraceae	G_Lachnospiraceae FCS020 group	unclassified_G_Lachnospiraceae FCS020 group
SV863	K_Bacteria	P_Firmicutes	C_Clostridia	O_Clostridiales	F_Clostridiaceae	G_Clostridium sensu stricto 1	unclassified_G_Clostridium sensu stricto 1
SV864	K_Bacteria	P_Firmicutes	C_Clostridia	O_Lachnospirales	F_Lachnospiraceae	G_Lachnospiraceae NK4A136 group	unclassified_G_Lachnospiraceae NK4A136 group
SV865	K_Bacteria	P_Firmicutes	C_Clostridia	O_Lachnospirales	F_Lachnospiraceae	G_GCA-900066575	unclassified_G_GCA-900066575
SV866	K_Bacteria	P_Firmicutes	C_Clostridia	O_Lachnospirales	F_Lachnospiraceae	unclassified_F_Lachnospiraceae	unclassified_F_Lachnospiraceae
SV867	K_Bacteria	P_Firmicutes	C_Clostridia	O_Lachnospirales	F_Lachnospiraceae	G_GCA-900066575	unclassified_G_GCA-900066575
SV868	K_Bacteria	P_Firmicutes	C_Clostridia	O_Oscillospirales	F_Ruminococcaceae	G_Incertae Sedis	unclassified_G_Incertae Sedis
SV869	K_Bacteria	P_Firmicutes	C_Clostridia	O_Clostridia UCG-014	unclassified_O_Clostridia UCG-014	unclassified_O_Clostridia UCG-014	unclassified_O_Clostridia UCG-014
SV870	K_Bacteria	P_Firmicutes	C_Clostridia	O_Oscillospirales	F_Ruminococcaceae	G_Incertae Sedis	unclassified_G_Incertae Sedis
SV871	K_Bacteria	P_Firmicutes	C_Clostridia	O_Lachnospirales	F_Lachnospiraceae	G_Eisenbergiella	unclassified_G_Eisenbergiella
SV872	K_Bacteria	P_Firmicutes	C_Clostridia	O_Lachnospirales	F_Lachnospiraceae	G_Lachnoclostridium	unclassified_G_Lachnoclostridium
SV873	K_Bacteria	P_Firmicutes	C_Clostridia	O_Lachnospirales	F_Lachnospiraceae	G_Acetitomaculum	unclassified_G_Acetitomaculum
SV874	K_Bacteria	P_Firmicutes	C_Clostridia	O_Clostridia vadinBB60 group	unclassified_O_Clostridia vadinBB60 group	unclassified_O_Clostridia vadinBB60 group	unclassified_O_Clostridia vadinBB60 group
SV875	K_Bacteria	P_Firmicutes	C_Clostridia	O_Lachnospirales	F_Lachnospiraceae	G_Lachnospiraceae UCG-001	unclassified_G_Lachnospiraceae UCG-001
SV876	K_Bacteria	P_Firmicutes	C_Clostridia	O_Oscillospirales	F_Butyricicoccaceae	G_UCG-009	unclassified_G_UCG-009
SV877	K_Bacteria	P_Firmicutes	C_Clostridia	O_Oscillospirales	F_Ruminococcaceae	G_Anaerotruncus	unclassified_G_Anaerotruncus
SV878	K_Bacteria	P_Bacteroidota	C_Bacteroidia	O_Bacteroidales	F_Marinifilaceae	G_Odoribacter	unclassified_G_Odoribacter
SV879	K_Bacteria	P_Firmicutes	C_Clostridia	O_Clostridiales	F_Clostridiaceae	G_Clostridium sensu stricto 1	unclassified_G_Clostridium sensu stricto 1
SV880	K_Bacteria	P_Firmicutes	C_Clostridia	O_Oscillospirales	F_Oscillospiraceae	unclassified_F_Oscillospiraceae	unclassified_F_Oscillospiraceae
SV881	K_Bacteria	P_Firmicutes	C_Clostridia	O_Lachnospirales	F_Lachnospiraceae	G_Lachnoclostridium	unclassified_G_Lachnoclostridium
SV882	K_Bacteria	P_Firmicutes	C_Clostridia	O_Lachnospirales	F_Defluviitaleaceae	G_Defluviitaleaceae UCG-011	unclassified_G_Defluviitaleaceae UCG-011
SV883	K_Bacteria	P_Firmicutes	C_Clostridia	O_Lachnospirales	F_Lachnospiraceae	G_Lachnospiraceae NK4A136 group	unclassified_G_Lachnospiraceae NK4A136 group
SV884	K_Bacteria	P_Firmicutes	C_Clostridia	O_Lachnospirales	F_Lachnospiraceae	G_Marvinbryantia	unclassified_G_Marvinbryantia
SV885	K_Bacteria	P_Firmicutes	C_Clostridia	O_Christensenellales	F_Christensenellaceae	G_Christensenellaceae R-7 group	unclassified_G_Christensenellaceae R-7 group
SV886	K_Bacteria	P_Firmicutes	C_Clostridia	O_Clostridia UCG-014	unclassified_O_Clostridia UCG-014	unclassified_O_Clostridia UCG-014	unclassified_O_Clostridia UCG-014
SV887	K_Bacteria	P_Bacteroidota	C_Bacteroidia	O_Bacteroidales	F_Bacteroidaceae	G_Bacteroides	unclassified_G_Bacteroides
SV888	K_Bacteria	P_Firmicutes	C_Clostridia	O_Lachnospirales	F_Lachnospiraceae	G_[Eubacterium] xylanophilum group	unclassified_G_[Eubacterium] xylanophilum group
SV889	K_Bacteria	P_Firmicutes	C_Clostridia	O_Lachnospirales	F_Lachnospiraceae	G_Lachnospiraceae UCG-001	unclassified_G_Lachnospiraceae UCG-001
SV890	K_Bacteria	P_Firmicutes	C_Clostridia	O_Lachnospirales	F_Lachnospiraceae	unclassified_F_Lachnospiraceae	unclassified_F_Lachnospiraceae
SV891	K_Bacteria	P_Actinobacteriota	C_Actinobacteria	O_Micrococcales	F_Micrococcaceae	G_Rothia	unclassified_G_Rothia
SV892	K_Bacteria	P_Firmicutes	C_Clostridia	O_Lachnospirales	F_Lachnospiraceae	unclassified_F_Lachnospiraceae	unclassified_F_Lachnospiraceae
SV893	K_Bacteria	P_Firmicutes	C_Clostridia	O_Lachnospirales	F_Lachnospiraceae	G_Lachnospiraceae NK4A136 group	unclassified_G_Lachnospiraceae NK4A136 group
SV894	K_Bacteria	P_Cyanobacteria	C_Vampirivibrionia	O_Gastranaerophilales	unclassified_O_Gastranaerophilales	unclassified_O_Gastranaerophilales	unclassified_O_Gastranaerophilales
SV895	K_Bacteria	P_Firmicutes	C_Clostridia	O_Peptostreptococcales-Tissierellales	F_Peptostreptococcaceae	G_Romboutsia	unclassified_G_Romboutsia
SV896	K_Bacteria	P_Bacteroidota	C_Bacteroidia	O_Bacteroidales	F_Muribaculaceae	unclassified_F_Muribaculaceae	unclassified_F_Muribaculaceae
SV897	K_Bacteria	P_Firmicutes	C_Clostridia	O_Christensenellales	F_Christensenellaceae	unclassified_F_Christensenellaceae	unclassified_F_Christensenellaceae
SV898	K_Bacteria	P_Firmicutes	C_Clostridia	O_Lachnospirales	F_Lachnospiraceae	unclassified_F_Lachnospiraceae	unclassified_F_Lachnospiraceae
SV899	K_Bacteria	P_Firmicutes	C_Clostridia	O_Clostridiales	F_Clostridiaceae	G_Clostridium sensu stricto 1	unclassified_G_Clostridium sensu stricto 1
SV900	K_Bacteria	P_Firmicutes	C_Clostridia	O_Lachnospirales	F_Lachnospiraceae	G_Lachnospiraceae NK4A136 group	unclassified_G_Lachnospiraceae NK4A136 group
SV901	K_Bacteria	P_Firmicutes	C_Clostridia	O_Oscillospirales	F_Oscillospiraceae	G_Intestinimonas	unclassified_G_Intestinimonas
SV902	K_Bacteria	P_Firmicutes	C_Clostridia	O_Lachnospirales	F_Lachnospiraceae	unclassified_F_Lachnospiraceae	unclassified_F_Lachnospiraceae
SV903	K_Bacteria	P_Firmicutes	C_Clostridia	O_Clostridia vadinBB60 group	unclassified_O_Clostridia vadinBB60 group	unclassified_O_Clostridia vadinBB60 group	unclassified_O_Clostridia vadinBB60 group
SV904	K_Bacteria	P_Bacteroidota	C_Bacteroidia	O_Bacteroidales	F_Rikenellaceae	G_Alistipes	unclassified_G_Alistipes
SV905	K_Bacteria	P_Firmicutes	C_Clostridia	O_Clostridiales	F_Clostridiaceae	G_Clostridium sensu stricto 1	unclassified_G_Clostridium sensu stricto 1
SV906	K_Bacteria	P_Firmicutes	C_Clostridia	O_Lachnospirales	F_Lachnospiraceae	G_Lachnospiraceae UCG-001	unclassified_G_Lachnospiraceae UCG-001
SV907	K_Bacteria	P_Firmicutes	C_Clostridia	O_Lachnospirales	F_Lachnospiraceae	G_Lachnospiraceae NK4A136 group	unclassified_G_Lachnospiraceae NK4A136 group
SV908	K_Bacteria	P_Bacteroidota	C_Bacteroidia	O_Bacteroidales	F_Muribaculaceae	unclassified_F_Muribaculaceae	unclassified_F_Muribaculaceae
SV909	K_Bacteria	P_Firmicutes	C_Clostridia	O_Lachnospirales	F_Lachnospiraceae	G_Roseburia	unclassified_G_Roseburia
SV910	K_Bacteria	P_Bacteroidota	C_Bacteroidia	O_Bacteroidales	F_Prevotellaceae	G_Prevotellaceae UCG-001	unclassified_G_Prevotellaceae UCG-001
SV911	K_Bacteria	P_Firmicutes	C_Clostridia	O_Lachnospirales	F_Lachnospiraceae	unclassified_F_Lachnospiraceae	unclassified_F_Lachnospiraceae
SV912	K_Bacteria	P_Firmicutes	C_Clostridia	O_Oscillospirales	F_Ruminococcaceae	G_Incertae Sedis	unclassified_G_Incertae Sedis
SV913	K_Bacteria	P_Firmicutes	C_Clostridia	O_Lachnospirales	F_Lachnospiraceae	G_Lachnospiraceae UCG-001	unclassified_G_Lachnospiraceae UCG-001
SV914	K_Bacteria	P_Firmicutes	C_Clostridia	O_Peptostreptococcales-Tissierellales	F_Anaerovoracaceae	G_Family XIII UCG-001	unclassified_G_Family XIII UCG-001
SV915	K_Bacteria	P_Firmicutes	C_Clostridia	O_Lachnospirales	F_Lachnospiraceae	G_Roseburia	unclassified_G_Roseburia
SV916	K_Bacteria	P_Firmicutes	C_Clostridia	O_Lachnospirales	F_Lachnospiraceae	G_Lachnospiraceae NK4A136 group	unclassified_G_Lachnospiraceae NK4A136 group
SV917	K_Bacteria	P_Firmicutes	C_Clostridia	O_Lachnospirales	F_Lachnospiraceae	G_Acetitomaculum	unclassified_G_Acetitomaculum
SV918	K_Bacteria	P_Firmicutes	C_Clostridia	O_Lachnospirales	F_Lachnospiraceae	unclassified_F_Lachnospiraceae	unclassified_F_Lachnospiraceae
SV919	K_Bacteria	P_Firmicutes	C_Clostridia	O_Oscillospirales	F_Ruminococcaceae	G_Ruminococcus	unclassified_G_Ruminococcus
SV920	K_Bacteria	P_Bacteroidota	C_Bacteroidia	O_Bacteroidales	F_Muribaculaceae	unclassified_F_Muribaculaceae	unclassified_F_Muribaculaceae
SV921	K_Bacteria	P_Firmicutes	C_Clostridia	O_Lachnospirales	F_Lachnospiraceae	G_Lachnospiraceae UCG-006	unclassified_G_Lachnospiraceae UCG-006
SV922	K_Bacteria	P_Firmicutes	C_Clostridia	O_Clostridia UCG-014	unclassified_O_Clostridia UCG-014	unclassified_O_Clostridia UCG-014	unclassified_O_Clostridia UCG-014
SV923	K_Bacteria	P_Firmicutes	C_Clostridia	O_Oscillospirales	F_Oscillospiraceae	G_Oscillibacter	unclassified_G_Oscillibacter
SV924	K_Bacteria	P_Firmicutes	C_Clostridia	O_Lachnospirales	F_Lachnospiraceae	G_A2	unclassified_G_A2
SV925	K_Bacteria	P_Firmicutes	C_Clostridia	O_Lachnospirales	F_Lachnospiraceae	G_Lachnoclostridium	unclassified_G_Lachnoclostridium
SV926	K_Bacteria	P_Firmicutes	C_Clostridia	O_Lachnospirales	F_Lachnospiraceae	G_Eisenbergiella	S_massiliensis
SV927	K_Bacteria	P_Firmicutes	C_Clostridia	O_Oscillospirales	F_Oscillospiraceae	G_Oscillibacter	unclassified_G_Oscillibacter
SV928	K_Bacteria	P_Firmicutes	C_Clostridia	O_Lachnospirales	F_Lachnospiraceae	G_Roseburia	unclassified_G_Roseburia
SV929	K_Bacteria	P_Firmicutes	C_Bacilli	O_Lactobacillales	F_Lactobacillaceae	G_Lactobacillus	unclassified_G_Lactobacillus
SV930	K_Bacteria	P_Firmicutes	C_Clostridia	O_Lachnospirales	F_Lachnospiraceae	unclassified_F_Lachnospiraceae	unclassified_F_Lachnospiraceae
SV931	K_Bacteria	P_Bacteroidota	C_Bacteroidia	O_Bacteroidales	F_Muribaculaceae	unclassified_F_Muribaculaceae	unclassified_F_Muribaculaceae
SV932	K_Bacteria	P_Firmicutes	C_Clostridia	O_Oscillospirales	F_Oscillospiraceae	unclassified_F_Oscillospiraceae	unclassified_F_Oscillospiraceae
SV933	K_Bacteria	P_Bacteroidota	C_Bacteroidia	O_Bacteroidales	F_Muribaculaceae	unclassified_F_Muribaculaceae	unclassified_F_Muribaculaceae
SV934	K_Bacteria	P_Cyanobacteria	C_Vampirivibrionia	O_Gastranaerophilales	unclassified_O_Gastranaerophilales	unclassified_O_Gastranaerophilales	unclassified_O_Gastranaerophilales
SV935	K_Bacteria	P_Firmicutes	C_Clostridia	O_Lachnospirales	F_Lachnospiraceae	G_Lachnospiraceae UCG-001	unclassified_G_Lachnospiraceae UCG-001
SV936	K_Bacteria	P_Firmicutes	C_Clostridia	O_Peptostreptococcales-Tissierellales	F_Anaerovoracaceae	G_Family XIII UCG-001	unclassified_G_Family XIII UCG-001
SV937	K_Bacteria	P_Firmicutes	C_Clostridia	O_Oscillospirales	F_UCG-010	unclassified_F_UCG-010	unclassified_F_UCG-010
SV938	K_Bacteria	P_Firmicutes	C_Clostridia	O_Lachnospirales	F_Lachnospiraceae	G_Lachnospiraceae NK4A136 group	unclassified_G_Lachnospiraceae NK4A136 group
SV939	K_Bacteria	P_Firmicutes	C_Clostridia	O_Lachnospirales	F_Lachnospiraceae	G_Lachnospiraceae FCS020 group	unclassified_G_Lachnospiraceae FCS020 group
SV940	K_Bacteria	P_Firmicutes	C_Clostridia	O_Lachnospirales	F_Lachnospiraceae	G_Lachnospiraceae NK4A136 group	unclassified_G_Lachnospiraceae NK4A136 group
SV941	K_Bacteria	P_Firmicutes	C_Clostridia	O_Oscillospirales	F_Ruminococcaceae	G_Incertae Sedis	unclassified_G_Incertae Sedis
SV942	K_Bacteria	P_Bacteroidota	C_Bacteroidia	O_Bacteroidales	F_Rikenellaceae	G_Alistipes	unclassified_G_Alistipes
SV943	K_Bacteria	P_Bacteroidota	C_Bacteroidia	O_Bacteroidales	F_Muribaculaceae	unclassified_F_Muribaculaceae	unclassified_F_Muribaculaceae
SV944	K_Bacteria	P_Firmicutes	C_Clostridia	O_Lachnospirales	F_Lachnospiraceae	G_[Ruminococcus] gnavus group	unclassified_G_[Ruminococcus] gnavus group
SV945	K_Bacteria	P_Firmicutes	C_Clostridia	O_Oscillospirales	F_Oscillospiraceae	G_Colidextribacter	unclassified_G_Colidextribacter
SV946	K_Bacteria	P_Firmicutes	C_Clostridia	O_Oscillospirales	F_Ruminococcaceae	unclassified_F_Ruminococcaceae	unclassified_F_Ruminococcaceae
SV947	K_Bacteria	P_Firmicutes	C_Bacilli	O_Lactobacillales	F_Lactobacillaceae	G_Lacticaseibacillus	unclassified_G_Lacticaseibacillus
SV948	K_Bacteria	P_Firmicutes	C_Clostridia	unclassified_C_Clostridia	unclassified_C_Clostridia	unclassified_C_Clostridia	unclassified_C_Clostridia
SV949	K_Bacteria	P_Firmicutes	C_Clostridia	O_Lachnospirales	F_Lachnospiraceae	unclassified_F_Lachnospiraceae	unclassified_F_Lachnospiraceae
SV950	K_Bacteria	P_Firmicutes	C_Clostridia	O_Oscillospirales	F_Oscillospiraceae	G_Oscillibacter	unclassified_G_Oscillibacter
SV951	K_Bacteria	P_Firmicutes	C_Clostridia	O_Lachnospirales	F_Lachnospiraceae	unclassified_F_Lachnospiraceae	unclassified_F_Lachnospiraceae
SV952	K_Bacteria	P_Firmicutes	C_Clostridia	O_Lachnospirales	F_Lachnospiraceae	unclassified_F_Lachnospiraceae	unclassified_F_Lachnospiraceae
SV953	K_Bacteria	P_Firmicutes	C_Clostridia	O_Clostridia UCG-014	unclassified_O_Clostridia UCG-014	unclassified_O_Clostridia UCG-014	unclassified_O_Clostridia UCG-014
SV954	K_Bacteria	P_Firmicutes	C_Clostridia	O_Oscillospirales	F_Butyricicoccaceae	G_Butyricicoccus	unclassified_G_Butyricicoccus
SV955	K_Bacteria	P_Firmicutes	C_Clostridia	O_Oscillospirales	F_[Eubacterium] coprostanoligenes group	unclassified_F_[Eubacterium] coprostanoligenes grou	unclassified_F_[Eubacterium] coprostanoligenes group
SV956	K_Bacteria	P_Firmicutes	C_Clostridia	O_Lachnospirales	F_Lachnospiraceae	G_Marvinbryantia	unclassified_G_Marvinbryantia
SV957	K_Bacteria	P_Bacteroidota	C_Bacteroidia	O_Bacteroidales	F_Muribaculaceae	unclassified_F_Muribaculaceae	unclassified_F_Muribaculaceae
SV958	K_Bacteria	P_Firmicutes	C_Clostridia	O_Oscillospirales	F_Ruminococcaceae	unclassified_F_Ruminococcaceae	unclassified_F_Ruminococcaceae
SV959	K_Bacteria	P_Firmicutes	C_Clostridia	O_Lachnospirales	F_Lachnospiraceae	unclassified_F_Lachnospiraceae	unclassified_F_Lachnospiraceae
SV960	K_Bacteria	P_Firmicutes	C_Bacilli	O_Lactobacillales	F_Lactobacillaceae	G_Lactobacillus	unclassified_G_Lactobacillus
SV961	K_Bacteria	P_Firmicutes	C_Clostridia	O_Lachnospirales	F_Lachnospiraceae	G_Lachnospiraceae NK4A136 group	unclassified_G_Lachnospiraceae NK4A136 group
SV962	K_Bacteria	P_Firmicutes	C_Clostridia	O_Lachnospirales	F_Lachnospiraceae	unclassified_F_Lachnospiraceae	unclassified_F_Lachnospiraceae
SV963	K_Bacteria	P_Firmicutes	C_Clostridia	O_Oscillospirales	F_Oscillospiraceae	G_UCG-003	unclassified_G_UCG-003
SV964	K_Bacteria	P_Bacteroidota	C_Bacteroidia	O_Bacteroidales	F_Prevotellaceae	G_Prevotellaceae UCG-001	unclassified_G_Prevotellaceae UCG-001

SV965	K Bacteria	P Firmicutes	C Clostridia	O Oscillospirales	F Oscillospiraceae	G Intestinimonas	unclassified G Intestinimonas
SV966	K Bacteria	P Firmicutes	C Clostridia	O Lachnospirales	F Lachnospiraceae	unclassified F Lachnospiraceae	unclassified F Lachnospiraceae
SV967	K Bacteria	P Firmicutes	C Clostridia	O Oscillospirales	F Ruminococcaceae	unclassified F Ruminococcaceae	unclassified F Ruminococcaceae
SV968	K Bacteria	P Firmicutes	C Clostridia	O Lachnospirales	F Lachnospiraceae	G Lachnoclostridium	unclassified G Lachnoclostridium
SV969	K Bacteria	P Firmicutes	C Clostridia	O Clostridia UCG-014	unclassified O Clostridia UCG-014	unclassified O Clostridia UCG-014	unclassified O Clostridia UCG-014
SV970	K Bacteria	P Actinobacteriota	C Coriobacteriia	O Coriobacteriales	F Eggerthellaceae	G Enterorhabdus	unclassified G Enterorhabdus
SV971	K Bacteria	P Firmicutes	C Clostridia	O Clostridiales	F Clostridiaceae	G Clostridium sensu stricto 1	unclassified G Clostridium sensu stricto 1
SV972	K Bacteria	P Firmicutes	C Clostridia	O Lachnospirales	F Lachnospiraceae	G GCA-900066575	unclassified G GCA-900066575
SV973	K Bacteria	P Firmicutes	C Clostridia	O Lachnospirales	F Lachnospiraceae	G Lachnospiraceae NK4A136 group	unclassified G Lachnospiraceae NK4A136 group
SV974	K Bacteria	P Firmicutes	C Clostridia	O Lachnospirales	F Lachnospiraceae	G Roseburia	unclassified G Roseburia
SV975	K Bacteria	P Proteobacteria	C Gammaproteobacteria	O Enterobacterales	F Enterobacteriaceae	G Escherichia-Shigella	unclassified G Escherichia-Shigella
SV976	K Bacteria	P Firmicutes	C Clostridia	O Lachnospirales	F Lachnospiraceae	unclassified F Lachnospiraceae	unclassified F Lachnospiraceae
SV977	K Bacteria	P Firmicutes	C Clostridia	O Lachnospirales	F Lachnospiraceae	G Lachnospiraceae UCG-001	unclassified G Lachnospiraceae UCG-001
SV978	K Bacteria	P Firmicutes	C Clostridia	O Lachnospirales	F Lachnospiraceae	G Lachnospiraceae UCG-001	unclassified G Lachnospiraceae UCG-001
SV979	K Bacteria	P Firmicutes	C Clostridia	O Lachnospirales	F Lachnospiraceae	G Marvinbryantia	unclassified G Marvinbryantia
SV980	K Bacteria	P Firmicutes	C Clostridia	O Oscillospirales	F Ruminococcaceae	unclassified F Ruminococcaceae	unclassified F Ruminococcaceae
SV981	K Bacteria	P Firmicutes	C Clostridia	O Lachnospirales	F Lachnospiraceae	G Lachnospiraceae FCS020 group	unclassified G Lachnospiraceae FCS020 group
SV982	K Bacteria	P Firmicutes	C Clostridia	O Oscillospirales	F Ruminococcaceae	G Incertae Sedis	unclassified G Incertae Sedis
SV983	K Bacteria	P Firmicutes	C Clostridia	O Lachnospirales	F Lachnospiraceae	G GCA-900066575	unclassified G GCA-900066575
SV984	K Bacteria	P Firmicutes	C Clostridia	O Clostridia UCG-014	unclassified O Clostridia UCG-014	unclassified O Clostridia UCG-014	unclassified O Clostridia UCG-014
SV985	K Bacteria	P Firmicutes	C Clostridia	O Lachnospirales	F Lachnospiraceae	G Lachnospiraceae FCS020 group	unclassified G Lachnospiraceae FCS020 group
SV986	K Bacteria	P Firmicutes	C Clostridia	O Oscillospirales	F Oscillospiraceae	G Oscillibacter	unclassified G Oscillibacter
SV987	K Bacteria	P Firmicutes	C Clostridia	O Lachnospirales	F Lachnospiraceae	unclassified F Lachnospiraceae	unclassified F Lachnospiraceae
SV988	K Bacteria	P Firmicutes	C Clostridia	O Lachnospirales	F Lachnospiraceae	G Lachnospiraceae NK4A136 group	unclassified G Lachnospiraceae NK4A136 group
SV989	K Bacteria	P Firmicutes	C Clostridia	O Lachnospirales	F Lachnospiraceae	G Lachnospiraceae NK4A136 group	unclassified G Lachnospiraceae NK4A136 group
SV990	K Bacteria	P Firmicutes	C Clostridia	O Oscillospirales	F Oscillospiraceae	G Colidextribacter	unclassified G Colidextribacter
SV991	K Bacteria	P Firmicutes	C Clostridia	O Oscillospirales	F Ruminococcaceae	G Harryflintia	unclassified G Harryflintia
SV992	K Bacteria	P Firmicutes	C Clostridia	O Oscillospirales	F Oscillospiraceae	G Oscillibacter	unclassified G Oscillibacter
SV993	K Bacteria	P Firmicutes	C Clostridia	O Lachnospirales	F Lachnospiraceae	G Lachnoclostridium	unclassified G Lachnoclostridium
SV994	K Bacteria	P Firmicutes	C Clostridia	O Lachnospirales	F Lachnospiraceae	unclassified F Lachnospiraceae	unclassified F Lachnospiraceae
SV995	K Bacteria	P Firmicutes	C Clostridia	O Oscillospirales	F Ruminococcaceae	unclassified F Ruminococcaceae	unclassified F Ruminococcaceae
SV996	K Bacteria	P Firmicutes	C Clostridia	O Lachnospirales	F Lachnospiraceae	unclassified F Lachnospiraceae	unclassified F Lachnospiraceae
SV997	K Bacteria	P Firmicutes	C Clostridia	O Lachnospirales	F Lachnospiraceae	G Lachnospiraceae NK4A136 group	unclassified G Lachnospiraceae NK4A136 group
SV998	K Bacteria	P Firmicutes	C Clostridia	O Christensenellales	F Christensenellaceae	G Christensenellaceae R-7 group	unclassified G Christensenellaceae R-7 group
SV999	K Bacteria	P Firmicutes	C Clostridia	O Oscillospirales	F Oscillospiraceae	G Oscillibacter	unclassified G Oscillibacter
SV1000	K Bacteria	P Firmicutes	C Clostridia	O Oscillospirales	F Ruminococcaceae	G Incertae Sedis	unclassified G Incertae Sedis
SV1001	K Bacteria	P Bacteroidota	C Bacteroidia	O Bacteroidales	F Muribaculaceae	unclassified F Muribaculaceae	unclassified F Muribaculaceae
SV1002	K Bacteria	P Firmicutes	C Clostridia	O Lachnospirales	F Lachnospiraceae	G Roseburia	unclassified G Roseburia
SV1003	K Bacteria	P Firmicutes	C Clostridia	O Lachnospirales	F Lachnospiraceae	G Lachnoclostridium	unclassified G Lachnoclostridium
SV1004	K Bacteria	P Firmicutes	C Clostridia	O Lachnospirales	F Lachnospiraceae	G Lachnospiraceae UCG-001	unclassified G Lachnospiraceae UCG-001
SV1005	K Bacteria	P Firmicutes	C Bacilli	O RF39	unclassified O RF39	unclassified O RF39	unclassified O RF39
SV1006	K Bacteria	P Firmicutes	C Bacilli	O Erysipelotrichales	F Erysipelotrichaceae	G Turicibacter	unclassified G Turicibacter
SV1007	K Bacteria	P Firmicutes	C Clostridia	O Oscillospirales	F Ruminococcaceae	unclassified F Ruminococcaceae	unclassified F Ruminococcaceae
SV1008	K Bacteria	P Bacteroidota	C Bacteroidia	O Bacteroidales	F Marinifilaceae	G Odoribacter	unclassified G Odoribacter
SV1009	K Bacteria	P Firmicutes	C Clostridia	O Lachnospirales	F Lachnospiraceae	G Lachnospiraceae NK4A136 group	unclassified G Lachnospiraceae NK4A136 group
SV1010	K Bacteria	P Firmicutes	C Clostridia	O Lachnospirales	F Lachnospiraceae	G Lachnospiraceae NK4A136 group	unclassified G Lachnospiraceae NK4A136 group
SV1011	K_Bacteria	P_Firmicutes	C_Clostridia	O_Lachnospirales	F_Lachnospiraceae	G_Lachnospiraceae UCG-001	unclassified_G_Lachnospiraceae UCG-001
SV1012	K Bacteria	P Firmicutes	C Clostridia	O Oscillospirales	F Oscillospiraceae	G Colidextribacter	unclassified G Colidextribacter
SV1013	K Bacteria	P Firmicutes	C Clostridia	O Oscillospirales	F UCG-010	unclassified F UCG-010	unclassified F UCG-010
SV1014	K Bacteria	P Firmicutes	C Clostridia	O Lachnospirales	F Lachnospiraceae	G Lachnospiraceae NK4A136 group	unclassified G Lachnospiraceae NK4A136 group
SV1015	K Bacteria	P Firmicutes	C Clostridia	O Oscillospirales	F Oscillospiraceae	unclassified F Oscillospiraceae	unclassified F Oscillospiraceae
SV1016	K Bacteria	P Firmicutes	C Clostridia	O Lachnospirales	F Lachnospiraceae	unclassified F Lachnospiraceae	unclassified F Lachnospiraceae
SV1017	K_Bacteria	P_Firmicutes	C_Clostridia	O_Clostridia UCG-014	unclassified_O_Clostridia UCG-014	unclassified_O_Clostridia UCG-014	unclassified_O_Clostridia UCG-014
SV1018	K Bacteria	P Firmicutes	C Clostridia	O Lachnospirales	F Lachnospiraceae	unclassified F Lachnospiraceae	unclassified F Lachnospiraceae
SV1019	K Bacteria	P Firmicutes	C Clostridia	O Lachnospirales	F Lachnospiraceae	G Marvinbryantia	unclassified G Marvinbryantia
SV1020	K Bacteria	P Firmicutes	C Clostridia	O Oscillospirales	F Ruminococcaceae	G Ruminococcus	unclassified G Ruminococcus
SV1021	K Bacteria	P Firmicutes	C Clostridia	O Lachnospirales	F Lachnospiraceae	unclassified F Lachnospiraceae	unclassified F Lachnospiraceae
SV1022	K Bacteria	P Firmicutes	C Clostridia	O Oscillospirales	F Oscillospiraceae	G Colidextribacter	unclassified G Colidextribacter
SV1023	K_Bacteria	P_Actinobacteriota	C_Coriobacteriia	O_Coriobacteriales	F_Eggerthellaceae	G_Enterorhabdus	unclassified_G_Enterorhabdus
SV1024	K Bacteria	P Bacteroidota	C Bacteroidia	O Bacteroidales	F Prevotellaceae	G Prevotellaceae UCG-001	unclassified G Prevotellaceae UCG-001
SV1025	K Bacteria	P Firmicutes	C Clostridia	O Oscillospirales	F Ruminococcaceae	G Ruminococcus	unclassified G Ruminococcus
SV1026	K Bacteria	P Firmicutes	C Clostridia	O Oscillospirales	F Oscillospiraceae	G Intestinimonas	unclassified G Intestinimonas
SV1027	K Bacteria	P Firmicutes	C Clostridia	O Lachnospirales	F Lachnospiraceae	G Lachnospiraceae UCG-001	unclassified G Lachnospiraceae UCG-001
SV1028	K Bacteria	P Firmicutes	C Clostridia	O Lachnospirales	F Lachnospiraceae	unclassified F Lachnospiraceae	unclassified F Lachnospiraceae
SV1029	K_Bacteria	P_Firmicutes	C_Bacilli	O_RF39	unclassified_O_RF39	unclassified_O_RF39	unclassified_O_RF39
SV1030	K Bacteria	P Firmicutes	C Bacilli	O Lactobacillales	F Lactobacillaceae	G HT002	unclassified G HT002
SV1031	K Bacteria	P Firmicutes	C Clostridia	O Lachnospirales	F Lachnospiraceae	G Lachnospiraceae NK4A136 group	unclassified G Lachnospiraceae NK4A136 group
SV1032	K Bacteria	P Firmicutes	C Clostridia	O Lachnospirales	F Lachnospiraceae	unclassified F Lachnospiraceae	unclassified F Lachnospiraceae
SV1033	K Bacteria	P Firmicutes	C Bacilli	O Lactobacillales	F Lactobacillaceae	G HT002	unclassified G HT002
SV1034	K Bacteria	P Firmicutes	C Clostridia	O Oscillospirales	F Oscillospiraceae	unclassified F Oscillospiraceae	unclassified F Oscillospiraceae
SV1035	K_Bacteria	P_Firmicutes	C_Clostridia	O_Lachnospirales	F_Lachnospiraceae	G_Acetatifactor	unclassified_G_Acetatifactor
SV1036	K Bacteria	P Firmicutes	C Clostridia	O Oscillospirales	F Ruminococcaceae	G Ruminococcus	unclassified G Ruminococcus
SV1037	K Bacteria	P Firmicutes	C Clostridia	O Clostridia UCG-014	unclassified O Clostridia UCG-014	unclassified O Clostridia UCG-014	unclassified O Clostridia UCG-014
SV1038	K Bacteria	P Firmicutes	C Clostridia	O Clostridia UCG-014	unclassified O Clostridia UCG-014	unclassified O Clostridia UCG-014	unclassified O Clostridia UCG-014
SV1039	K Bacteria	P Cyanobacteria	C Vampirivibrionia	O Gastranaerophilales	unclassified O Gastranaerophilales	unclassified O Gastranaerophilales	unclassified O Gastranaerophilales
SV1040	K Bacteria	P Firmicutes	C Clostridia	O Oscillospirales	F Oscillospiraceae	G Colidextribacter	unclassified G Colidextribacter
SV1041	K Bacteria	P Cyanobacteria	C Vampirivibrionia	O Gastranaerophilales	unclassified O Gastranaerophilales	unclassified O Gastranaerophilales	unclassified O Gastranaerophilales
SV1042	K Bacteria	P Verrucomicrobiota	C Verrucomicrobiae	O Verrucomicrobiales	F Akkermansiaceae	G Akkermansia	unclassified G Akkermansia
SV1043	K Bacteria	P Firmicutes	C Clostridia	O Oscillospirales	F Ruminococcaceae	G Ruminococcus	unclassified G Ruminococcus
SV1044	K Bacteria	P Firmicutes	C Clostridia	O Lachnospirales	F Lachnospiraceae	G Marvinbryantia	unclassified G Marvinbryantia
SV1045	K Bacteria	P Firmicutes	C Bacilli	O Lactobacillales	F Lactobacillaceae	G Ligilactobacillus	unclassified G Ligilactobacillus
SV1046	K Bacteria	P Firmicutes	C Clostridia	O Peptococcales	F Peptococcaceae	G Peptococcus	unclassified G Peptococcus
SV1047	K Bacteria	P Firmicutes	C Clostridia	O Lachnospirales	F Lachnospiraceae	unclassified F Lachnospiraceae	unclassified F Lachnospiraceae
SV1048	K Bacteria	P Firmicutes	C Clostridia	O Lachnospirales	F Lachnospiraceae	G Roseburia	unclassified G Roseburia
SV1049	K Bacteria	P Firmicutes	C Clostridia	O Lachnospirales	F Lachnospiraceae	G Dorea	unclassified G Dorea
SV1050	K Bacteria	P Firmicutes	C Clostridia	O Peptostreptococcales-Tissierellales	F Peptostreptococcaceae	unclassified F Peptostreptococcaceae	unclassified F Peptostreptococcaceae
SV1051	K Bacteria	P Bacteroidota	C Bacteroidia	O Bacteroidales	F Muribaculaceae	unclassified F Muribaculaceae	unclassified F Muribaculaceae
SV1052	K Bacteria	P Firmicutes	C Clostridia	O Lachnospirales	F Lachnospiraceae	unclassified F Lachnospiraceae	unclassified F Lachnospiraceae
SV1053	K Bacteria	P Firmicutes	C Clostridia	O Lachnospirales	F Lachnospiraceae	G Roseburia	unclassified G Roseburia
SV1054	K Bacteria	P Firmicutes	C Clostridia	O Oscillospirales	F Butyricicoccaceae	G Butyricicoccus	unclassified G Butyricicoccus
SV1055	K Bacteria	P Firmicutes	C Clostridia	O Lachnospirales	F Lachnospiraceae	unclassified F Lachnospiraceae	unclassified F Lachnospiraceae
SV1056	K Bacteria	P Firmicutes	C Clostridia	O Lachnospirales	F Lachnospiraceae	G ASF356	unclassified G ASF356
SV1057	K Bacteria	P Firmicutes	C Clostridia	O Lachnospirales	F Lachnospiraceae	G GCA-900066575	unclassified G GCA-900066575
SV1058	K Bacteria	P Firmicutes	C Clostridia	O Lachnospirales	F Lachnospiraceae	G Lachnoclostridium	unclassified G Lachnoclostridium
SV1059	K Bacteria	P Firmicutes	C Clostridia	O Oscillospirales	F Oscillospiraceae	G Colidextribacter	unclassified G Colidextribacter
SV1060	K Bacteria	P Firmicutes	C Clostridia	O Oscillospirales	F Oscillospiraceae	unclassified F Oscillospiraceae	unclassified F Oscillospiraceae
SV1061	K Bacteria	P Firmicutes	C Clostridia	O Oscillospirales	F Oscillospiraceae	G Oscillibacter	unclassified G Oscillibacter
SV1062	K Bacteria	P Firmicutes	C Clostridia	O Lachnospirales	F Lachnospiraceae	G Tuzzerella	unclassified G Tuzzerella
SV1063	K Bacteria	P Firmicutes	C Clostridia	O Clostridiales	F Clostridiaceae	G Clostridium sensu stricto 1	unclassified G Clostridium sensu stricto 1
SV1064	K Bacteria	P Firmicutes	C Clostridia	O Oscillospirales	F Ruminococcaceae	unclassified F Ruminococcaceae	unclassified F Ruminococcaceae
SV1065	K Bacteria	P Firmicutes	C Clostridia	O Lachnospirales	F Lachnospiraceae	unclassified F Lachnospiraceae	unclassified F Lachnospiraceae
SV1066	K Bacteria	P Cyanobacteria	C Vampirivibrionia	O Gastranaerophilales	unclassified O Gastranaerophilales	unclassified O Gastranaerophilales	unclassified O Gastranaerophilales
SV1067	K Bacteria	P Firmicutes	C Clostridia	O Lachnospirales	F Lachnospiraceae	G Marvinbryantia	unclassified G Marvinbryantia
SV1068	K Bacteria	P Firmicutes	C Clostridia	O Lachnospirales	F Lachnospiraceae	G GCA-900066575	unclassified G GCA-900066575
SV1069	K Bacteria	P Firmicutes	C Clostridia	O Oscillospirales	F Butyricicoccaceae	G Butyricicoccus	unclassified G Butyricicoccus
SV1070	K Bacteria	P Firmicutes	C Clostridia	O Lachnospirales	F Lachnospiraceae	G Lachnospiraceae NK4A136 group	unclassified G Lachnospiraceae NK4A136 group
SV1071	K Bacteria	P Firmicutes	C Clostridia	O Lachnospirales	F Lachnospiraceae	G Lachnospiraceae NK4A136 group	unclassified G Lachnospiraceae NK4A136 group
SV1072	K Bacteria	P Firmicutes	C Clostridia	O Lachnospirales	F Lachnospiraceae	G [Ruminococcus] gauvreauii group	unclassified G [Ruminococcus] gauvreauii group
SV1073	K Bacteria	P Firmicutes	C Clostridia	O Lachnospirales	F Lachnospiraceae	G Lachnospiraceae NK4A136 group	unclassified G Lachnospiraceae NK4A136 group
SV1074	K Bacteria	P Firmicutes	C Clostridia	O Lachnospirales	F Lachnospiraceae	G Lachnospiraceae NK4A136 group	unclassified G Lachnospiraceae NK4A136 group
SV1075	K Bacteria	P Firmicutes	C Clostridia	O Lachnospirales	F Lachnospiraceae	G Lachnospiraceae NK4A136 group	S bacterium
SV1076	K_Bacteria	P_Firmicutes	C_Clostridia	O_Lachnospirales	F_Lachnospiraceae	unclassified_F_Lachnospiraceae	unclassified_F_Lachnospiraceae
SV1077	K Bacteria	P Firmicutes	C Clostridia	O Clostridia UCG-014	unclassified O Clostridia UCG-014	unclassified O Clostridia UCG-014	unclassified O Clostridia UCG-014
SV1078	K Bacteria	P Firmicutes	C Clostridia	O Lachnospirales	F Lachnospiraceae	G [Eubacterium] ruminantium group	unclassified G [Eubacterium] ruminantium group
SV1079	K Bacteria	P Firmicutes	C Clostridia	O Lachnospirales	F Lachnospiraceae	G GCA-900066575	unclassified G GCA-900066575
SV1080	K Bacteria	P Firmicutes	C Clostridia	O Oscillospirales	F Oscillospiraceae	unclassified F Oscillospiraceae	unclassified F Oscillospiraceae
SV1081	K Bacteria	P Firmicutes	C Clostridia	O Peptostreptococcales-Tissierellales	F Anaerovoracaceae	G Family XIII AD3011 group	unclassified G Family XIII AD3011 group
SV1082	K_Bacteria	P_Firmicutes	C_Clostridia	O_Oscillospirales	F_Oscillospiraceae	G_Colidextribacter	unclassified_G_Colidextribacter
SV1083	K Bacteria	P Firmicutes	C Clostridia	O Lachnospirales	F Lachnospiraceae	G Lachnospiraceae NK4A136 group	unclassified G Lachnospiraceae NK4A136 group
SV1084	K Bacteria	P Bacteroidota	C Bacteroidia	O Bacteroidales	F Bacteroidaceae	G Bacteroides	unclassified G Bacteroides
SV1085	K Bacteria	P Firmicutes	C Clostridia	O Lachnospirales	F Lachnospiraceae	G Marvinbryantia	unclassified G Marvinbryantia
SV1086	K Bacteria	P Bacteroidota	C Bacteroidia	O Bacteroidales	F Muribaculaceae	unclassified F Muribaculaceae	unclassified F Muribaculaceae
SV1087	K Bacteria	P Firmicutes	C Clostridia	O Clostridia UCG-014	unclassified O Clostridia UCG-014	unclassified O Clostridia UCG-014	unclassified O Clostridia UCG-014
SV1088	K_Bacteria	P_Firmicutes	C_Clostridia	O_Lachnospirales	F_Lachnospiraceae	G_Lachnoclostridium	unclassified_G_Lachnoclostridium
SV1089	K Bacteria	P Firmicutes	C Clostridia	O Clostridia UCG-014	unclassified O Clostridia UCG-014	unclassified O Clostridia UCG-014	unclassified O Clostridia UCG-014
SV1090	K Bacteria	P Firmicutes	C Clostridia	O Peptostreptococcales-Tissierellales	F Anaerovoracaceae	G Family XIII AD3011 group	unclassified G Family XIII AD3011 group
SV1091	K Bacteria	P Firmicutes	C Clostridia	O Lachnospirales	F Lachnospiraceae	G ASF356	unclassified G ASF356
SV1092	K Bacteria	P Firmicutes	C Clostridia	O Oscillospirales	F Oscillospiraceae	G Colidextribacter	unclassified G Colidextribacter
SV1093	K Bacteria	P Firmicutes	C Clostridia	O Lachnospirales	F Lachnospiraceae	G Lachnospiraceae NK4A136 group	unclassified G Lachnospiraceae NK4A136 group
SV1094	K_Bacteria	P_Firmicutes	C_Clostridia	O_Lachnospirales	F_Lachnospiraceae	unclassified_F_Lachnospiraceae	unclassified_F_Lachnospiraceae
SV1095	K Bacteria	P Firmicutes	C Clostridia	O Lachnospirales	F Lachnospiraceae	G Murimonas	S intestini
SV1096	K Bacteria	P Firmicutes	C Clostridia	O Oscillospirales	F Oscillospiraceae	unclassified F Oscillospiraceae	unclassified F Oscillospiraceae
SV1097	K Bacteria	P Firmicutes	C Clostridia	O Lachnospirales	F Lachnospiraceae	G Lachnospiraceae UCG-001	unclassified G Lachnospiraceae UCG-001
SV1098	K Bacteria	P Firmicutes	C Clostridia	O Oscillospirales	F Oscillospiraceae	unclassified F Oscillospiraceae	unclassified F Oscillospiraceae
SV1099	K Bacteria	P Firmicutes	C Clostridia	O Lachnospirales	F Lachnospiraceae	G Lachnospiraceae UCG-006	unclassified G Lachnospiraceae UCG-006
SV1100	K Bacteria	P Firmicutes	C Clostridia	O Lachnospirales	F Lachnospiraceae	G Roseburia	unclassified G Roseburia
SV1101	K Bacteria	P Firmicutes	C Clostridia	O Lachnospirales	F Lachnospiraceae	unclassified F Lachnospiraceae	unclassified F Lachnospiraceae
SV1102	K Bacteria	P Firmicutes	C Clostridia	O Peptostreptococcales-Tissierellales	F Peptostreptococcaceae	G Romboutsia	unclassified G Romboutsia

SV1103	K Bacteria	P Firmicutes	C Clostridia	O Oscillospirales	F Oscillospiraceae	unclassified F Oscillospiraceae	unclassified F Oscillospiraceae
SV1104	K Bacteria	P Firmicutes	C Clostridia	O Lachnospirales	F Lachnospiraceae	G Lachnospiraceae NK4A136 group	unclassified G Lachnospiraceae NK4A136 group
SV1105	K Bacteria	P Firmicutes	C Clostridia	O Lachnospirales	F Lachnospiraceae	unclassified F Lachnospiraceae	unclassified F Lachnospiraceae
SV1106	K Bacteria	P Firmicutes	C Clostridia	O Lachnospirales	F Lachnospiraceae	G Lachnospiraceae FCS020 group	unclassified G Lachnospiraceae FCS020 group
SV1107	K Bacteria	P Proteobacteria	C Gammaproteobacteria	O Enterobacterales	F Enterobacteriaceae	G Salmonella	S enterica
SV1108	K Bacteria	P Firmicutes	C Clostridia	O Lachnospirales	F Lachnospiraceae	G Lachnospiraceae UCG-006	unclassified G Lachnospiraceae UCG-006
SV1109	K Bacteria	P Firmicutes	C Clostridia	O Lachnospirales	F Lachnospiraceae	G Roseburia	unclassified G Roseburia
SV1110	K Bacteria	P Firmicutes	C Clostridia	O Lachnospirales	F Lachnospiraceae	G Lachnospiraceae UCG-001	unclassified G Lachnospiraceae UCG-001
SV1111	K Bacteria	P Firmicutes	C Clostridia	O Lachnospirales	F Lachnospiraceae	unclassified F Lachnospiraceae	unclassified F Lachnospiraceae
SV1112	K Bacteria	P Firmicutes	C Clostridia	O Lachnospirales	F Lachnospiraceae	unclassified F Lachnospiraceae	unclassified F Lachnospiraceae
SV1113	K Bacteria	P Firmicutes	C Clostridia	O Peptostreptococcales-Tissierellales	F Peptostreptococcaceae	G Romboutsia	unclassified G Romboutsia
SV1114	K Bacteria	P Firmicutes	C Clostridia	O Oscillospirales	F Oscillospiraceae	G Colidextribacter	unclassified G Colidextribacter
SV1115	K Bacteria	P Firmicutes	C Clostridia	O Lachnospirales	F Lachnospiraceae	unclassified F Lachnospiraceae	unclassified F Lachnospiraceae
SV1116	K Bacteria	P Firmicutes	C Clostridia	O Lachnospirales	F Lachnospiraceae	G Lachnospiraceae NK4A136 group	unclassified G Lachnospiraceae NK4A136 group
SV1117	K Bacteria	P Firmicutes	C Clostridia	O Oscillospirales	F Ruminococcaceae	G Incertae Sedis	unclassified G Incertae Sedis
SV1118	K Bacteria	P Firmicutes	C Clostridia	O Clostridia UCG-014	unclassified O Clostridia UCG-014	unclassified O Clostridia UCG-014	unclassified O Clostridia UCG-014
SV1119	K Bacteria	P Firmicutes	C Clostridia	O Lachnospirales	F Lachnospiraceae	unclassified F Lachnospiraceae	unclassified F Lachnospiraceae
SV1120	K Bacteria	P Firmicutes	C Clostridia	O Lachnospirales	F Lachnospiraceae	G GCA-900066575	unclassified G GCA-900066575
SV1121	K Bacteria	P Firmicutes	C Clostridia	O Clostridia UCG-014	unclassified O Clostridia UCG-014	unclassified O Clostridia UCG-014	unclassified O Clostridia UCG-014
SV1122	K Bacteria	P Firmicutes	C Clostridia	O Lachnospirales	F Lachnospiraceae	G Lachnospiraceae UCG-010	unclassified G Lachnospiraceae UCG-010
SV1123	K Bacteria	P Firmicutes	C Clostridia	O Lachnospirales	F Lachnospiraceae	G A2	unclassified G A2
SV1124	K Bacteria	P Firmicutes	C Clostridia	O Clostridiales	F Clostridiaceae	G Clostridium sensu stricto 1	unclassified G Clostridium sensu stricto 1
SV1125	K Bacteria	P Bacteroidota	C Bacteroidia	O Bacteroidales	F Prevotellaceae	G Prevotellaceae UCG-001	unclassified G Prevotellaceae UCG-001
SV1126	K Bacteria	P Firmicutes	C Bacilli	O Erysipelotrichales	F Erysipelatoclostridiaceae	G Erysipelatoclostridium	unclassified G Erysipelatoclostridium
SV1127	K Bacteria	P Firmicutes	C Clostridia	O Oscillospirales	F Oscillospiraceae	G Colidextribacter	unclassified G Colidextribacter
SV1128	K Bacteria	P Firmicutes	C Clostridia	O Clostridia UCG-014	unclassified O Clostridia UCG-014	unclassified O Clostridia UCG-014	unclassified O Clostridia UCG-014
SV1129	K Bacteria	P Firmicutes	C Clostridia	O Lachnospirales	F Lachnospiraceae	G Marvinbryantia	unclassified G Marvinbryantia
SV1130	K Bacteria	P Firmicutes	C Bacilli	O Staphylococcales	F Staphylococcaceae	G Staphylococcus	unclassified G Staphylococcus
SV1131	K Bacteria	P Firmicutes	C Clostridia	O Lachnospirales	F Lachnospiraceae	unclassified F Lachnospiraceae	unclassified F Lachnospiraceae
SV1132	K Bacteria	P Firmicutes	C Clostridia	O Lachnospirales	F Lachnospiraceae	unclassified F Lachnospiraceae	unclassified F Lachnospiraceae
SV1133	K Bacteria	P Firmicutes	C Bacilli	O Lactobacillales	F Enterococcaceae	G Enterococcus	unclassified G Enterococcus
SV1134	K Bacteria	P Firmicutes	C Clostridia	O Oscillospirales	F Ruminococcaceae	G Incertae Sedis	unclassified G Incertae Sedis
SV1135	K Bacteria	P Firmicutes	C Clostridia	O Lachnospirales	F Lachnospiraceae	G Marvinbryantia	unclassified G Marvinbryantia
SV1136	K Bacteria	P Firmicutes	C Clostridia	O Lachnospirales	F Lachnospiraceae	G Marvinbryantia	unclassified G Marvinbryantia
SV1137	K Bacteria	P Firmicutes	C Clostridia	O Peptostreptococcales-Tissierellales	F Peptostreptococcaceae	G Romboutsia	unclassified G Romboutsia
SV1138	K Bacteria	P Firmicutes	C Clostridia	O Lachnospirales	F Lachnospiraceae	G Lachnospiraceae NK4A136 group	unclassified G Lachnospiraceae NK4A136 group
SV1139	K Bacteria	P Firmicutes	C Clostridia	O Christensenellales	F Christensenellaceae	G Christensenellaceae R-7 group	unclassified G Christensenellaceae R-7 group
SV1140	K Bacteria	P Firmicutes	C Clostridia	O Lachnospirales	F Lachnospiraceae	G Roseburia	unclassified G Roseburia
SV1141	K Bacteria	P Firmicutes	C Clostridia	O Lachnospirales	F Lachnospiraceae	G Lachnospiraceae NK4A136 group	unclassified G Lachnospiraceae NK4A136 group
SV1142	K Bacteria	P Firmicutes	C Clostridia	O Clostridia UCG-014	unclassified O Clostridia UCG-014	unclassified O Clostridia UCG-014	unclassified O Clostridia UCG-014
SV1143	K Bacteria	P Firmicutes	C Clostridia	O Oscillospirales	F Oscillospiraceae	unclassified F Oscillospiraceae	unclassified F Oscillospiraceae
SV1144	K Bacteria	P Firmicutes	C Clostridia	O Oscillospirales	F Ruminococcaceae	unclassified F Ruminococcaceae	unclassified F Ruminococcaceae
SV1145	K Bacteria	P Firmicutes	C Clostridia	O Clostridia UCG-014	unclassified O Clostridia UCG-014	unclassified O Clostridia UCG-014	unclassified O Clostridia UCG-014
SV1146	K Bacteria	P Firmicutes	C Clostridia	O Clostridia UCG-014	unclassified O Clostridia UCG-014	unclassified O Clostridia UCG-014	unclassified O Clostridia UCG-014
SV1147	K Bacteria	P Firmicutes	C Clostridia	O Oscillospirales	F Ruminococcaceae	unclassified F Ruminococcaceae	unclassified F Ruminococcaceae
SV1148	K Bacteria	P Firmicutes	C Clostridia	O Lachnospirales	F Lachnospiraceae	G Blautia	unclassified G Blautia
SV1149	K_Bacteria	P_Firmicutes	C_Clostridia	O_Lachnospirales	F_Lachnospiraceae	unclassified_F_Lachnospiraceae	unclassified_F_Lachnospiraceae
SV1150	K Bacteria	P Bacteroidota	C Bacteroidia	O Bacteroidales	F Muribaculaceae	unclassified F Muribaculaceae	unclassified F Muribaculaceae
SV1151	K Bacteria	P Firmicutes	C Bacilli	O Lactobacillales	F Lactobacillaceae	G Ligilactobacillus	unclassified G Ligilactobacillus
SV1152	K Bacteria	P Firmicutes	C Clostridia	O Lachnospirales	F Lachnospiraceae	G Lachnospiraceae NK4A136 group	unclassified G Lachnospiraceae NK4A136 group
SV1153	K Bacteria	P Firmicutes	C Clostridia	O Oscillospirales	F Oscillospiraceae	G Oscillibacter	unclassified G Oscillibacter
SV1154	K Bacteria	P Firmicutes	C Clostridia	O Oscillospirales	F Ruminococcaceae	G Candidatus Soleaferrea	unclassified G Candidatus Soleaferrea
SV1155	K_Bacteria	P_Firmicutes	C_Clostridia	O_Oscillospirales	F_Oscillospiraceae	G_Colidextribacter	unclassified_G_Colidextribacter
SV1156	K Bacteria	P Firmicutes	C Clostridia	O Oscillospirales	F Ruminococcaceae	G UBA1819	unclassified G UBA1819
SV1157	K Bacteria	P Firmicutes	C Clostridia	O Oscillospirales	F Butyricicoccaceae	G Butyricicoccus	unclassified G Butyricicoccus
SV1158	K Bacteria	P Bacteroidota	C Bacteroidia	O Bacteroidales	F Bacteroidaceae	G Bacteroides	unclassified G Bacteroides
SV1159	K Bacteria	P Firmicutes	C Clostridia	O Lachnospirales	F Lachnospiraceae	G A2	unclassified G A2
SV1160	K Bacteria	P Firmicutes	C Clostridia	O Oscillospirales	F Ruminococcaceae	G Incertae Sedis	unclassified G Incertae Sedis
SV1161	K_Bacteria	P_Firmicutes	C_Clostridia	O_Lachnospirales	F_Lachnospiraceae	G_ASF356	unclassified_G_ASF356
SV1162	K Bacteria	P Firmicutes	C Clostridia	O Lachnospirales	F Lachnospiraceae	G [Eubacterium] ventriosum group	unclassified G [Eubacterium] ventriosum group
SV1163	K Bacteria	P Firmicutes	C Clostridia	O Clostridia UCG-014	unclassified O Clostridia UCG-014	unclassified O Clostridia UCG-014	unclassified O Clostridia UCG-014
SV1164	K Bacteria	P Firmicutes	C Clostridia	O Oscillospirales	F Oscillospiraceae	G NK4A214 group	unclassified G NK4A214 group
SV1165	K Bacteria	P Firmicutes	C Clostridia	O Clostridia UCG-014	unclassified O Clostridia UCG-014	unclassified O Clostridia UCG-014	unclassified O Clostridia UCG-014
SV1166	K Bacteria	P Firmicutes	C Clostridia	O Lachnospirales	F Lachnospiraceae	unclassified F Lachnospiraceae	unclassified F Lachnospiraceae
SV1167	K_Bacteria	P_Firmicutes	C_Clostridia	O_Lachnospirales	F_Lachnospiraceae	G_GCA-900066575	unclassified_G_GCA-900066575
SV1168	K Bacteria	P Firmicutes	C Clostridia	O Lachnospirales	F Lachnospiraceae	G Lachnoclostridium	unclassified G Lachnoclostridium
SV1169	K Bacteria	P Firmicutes	C Clostridia	O Lachnospirales	F Lachnospiraceae	G [Eubacterium] xylanophilum group	unclassified G [Eubacterium] xylanophilum group
SV1170	K Bacteria	P Firmicutes	C Clostridia	O Lachnospirales	F Lachnospiraceae	unclassified F Lachnospiraceae	unclassified F Lachnospiraceae
SV1171	K Bacteria	P Firmicutes	C Clostridia	O Clostridia UCG-014	unclassified O Clostridia UCG-014	unclassified O Clostridia UCG-014	unclassified O Clostridia UCG-014
SV1172	K Bacteria	P Firmicutes	C Clostridia	O Oscillospirales	F Oscillospiraceae	unclassified F Oscillospiraceae	unclassified F Oscillospiraceae
SV1173	K_Bacteria	P_Firmicutes	C_Clostridia	O_Lachnospirales	F_Lachnospiraceae	G_Lachnoclostridium	unclassified_G_Lachnoclostridium
SV1174	K Bacteria	P Firmicutes	C Bacilli	O Staphylococcales	F Staphylococcaceae	G Jeotgalicoccus	unclassified G Jeotgalicoccus
SV1175	K Bacteria	P Firmicutes	C Clostridia	O Clostridia UCG-014	unclassified O Clostridia UCG-014	unclassified O Clostridia UCG-014	unclassified O Clostridia UCG-014
SV1176	K Bacteria	P Bacteroidota	C Bacteroidia	O Bacteroidales	F Muribaculaceae	unclassified F Muribaculaceae	unclassified F Muribaculaceae
SV1177	K Bacteria	P Firmicutes	C Clostridia	O Clostridia UCG-014	unclassified O Clostridia UCG-014	unclassified O Clostridia UCG-014	unclassified O Clostridia UCG-014
SV1178	K Bacteria	P Firmicutes	C Clostridia	O Clostridia UCG-014	unclassified O Clostridia UCG-014	unclassified O Clostridia UCG-014	unclassified O Clostridia UCG-014
SV1179	K Bacteria	P Firmicutes	C Clostridia	O Lachnospirales	F Lachnospiraceae	G ASF356	unclassified G ASF356
SV1180	K Bacteria	P Firmicutes	C Clostridia	O Lachnospirales	F Lachnospiraceae	G Marvinbryantia	unclassified G Marvinbryantia
SV1181	K Bacteria	P Firmicutes	C Clostridia	O Lachnospirales	F Lachnospiraceae	unclassified F Lachnospiraceae	unclassified F Lachnospiraceae
SV1182	K Bacteria	P Firmicutes	C Clostridia	O Lachnospirales	F Lachnospiraceae	G Lachnospiraceae NK4A136 group	unclassified G Lachnospiraceae NK4A136 group
SV1183	K Bacteria	P Firmicutes	C Clostridia	O Lachnospirales	F Lachnospiraceae	unclassified F Lachnospiraceae	unclassified F Lachnospiraceae
SV1184	K Bacteria	P Firmicutes	C Clostridia	O Clostridiales	F Clostridiaceae	G Clostridium sensu stricto 1	unclassified G Clostridium sensu stricto 1
SV1185	K Bacteria	P Firmicutes	C Clostridia	O Lachnospirales	F Lachnospiraceae	unclassified F Lachnospiraceae	unclassified F Lachnospiraceae
SV1186	K Bacteria	P Firmicutes	C Clostridia	O Clostridia vadinBB60 group	unclassified O Clostridia vadinBB60 group	unclassified O Clostridia vadinBB60 group	unclassified O Clostridia vadinBB60 group
SV1187	K Bacteria	P Firmicutes	C Clostridia	O Lachnospirales	F Lachnospiraceae	G ASF356	unclassified G ASF356
SV1188	K Bacteria	P Firmicutes	C Clostridia	O Lachnospirales	F Lachnospiraceae	unclassified F Lachnospiraceae	unclassified F Lachnospiraceae
SV1189	K Bacteria	P Firmicutes	C Clostridia	O Lachnospirales	F Lachnospiraceae	G Lachnospiraceae NK4A136 group	unclassified G Lachnospiraceae NK4A136 group
SV1190	K Bacteria	P Firmicutes	C Clostridia	O Lachnospirales	F Lachnospiraceae	unclassified F Lachnospiraceae	unclassified F Lachnospiraceae
SV1191	K Bacteria	P Bacteroidota	C Bacteroidia	O Bacteroidales	F Tannerellaceae	unclassified F Tannerellaceae	unclassified F Tannerellaceae
SV1192	K Bacteria	P Firmicutes	C Clostridia	O Oscillospirales	F Ruminococcaceae	G Incertae Sedis	unclassified G Incertae Sedis
SV1193	K Bacteria	P Firmicutes	C Clostridia	O Lachnospirales	F Lachnospiraceae	G Marvinbryantia	unclassified G Marvinbryantia
SV1194	K Bacteria	P Firmicutes	C Clostridia	O Oscillospirales	F Oscillospiraceae	G Oscillibacter	unclassified G Oscillibacter
SV1195	K Bacteria	P Bacteroidota	C Bacteroidia	O Bacteroidales	F Muribaculaceae	unclassified F Muribaculaceae	unclassified F Muribaculaceae
SV1196	K Bacteria	P Firmicutes	C Clostridia	O Lachnospirales	F Lachnospiraceae	unclassified F Lachnospiraceae	unclassified F Lachnospiraceae
SV1197	K Bacteria	P Firmicutes	C Clostridia	O Lachnospirales	F Lachnospiraceae	G Roseburia	unclassified G Roseburia
SV1198	K Bacteria	P Bacteroidota	C Bacteroidia	O Bacteroidales	F Marinifilaceae	G Odoribacter	unclassified G Odoribacter
SV1199	K Bacteria	P Firmicutes	C Clostridia	O Lachnospirales	F Lachnospiraceae	G Roseburia	unclassified G Roseburia
SV1200	K Bacteria	P Firmicutes	C Clostridia	O Lachnospirales	F Lachnospiraceae	G ASF356	unclassified G ASF356
SV1201	K Bacteria	P Actinobacteriota	C Coriobacteriia	O Coriobacteriales	F Eggerthellaceae	G Enterorhabdus	S mucosicola
SV1202	K Bacteria	P Firmicutes	C Clostridia	O Lachnospirales	F Lachnospiraceae	G Lachnoclostridium	unclassified G Lachnoclostridium
SV1203	K Bacteria	P Firmicutes	C Clostridia	O Oscillospirales	F Butyricicoccaceae	G Butyricicoccus	unclassified G Butyricicoccus
SV1204	K Bacteria	P Actinobacteriota	C Coriobacteriia	O Coriobacteriales	F Eggerthellaceae	G Enterorhabdus	unclassified G Enterorhabdus
SV1205	K Bacteria	P Bacteroidota	C Bacteroidia	O Bacteroidales	F Bacteroidaceae	G Bacteroides	unclassified G Bacteroides
SV1206	K Bacteria	P Firmicutes	C Clostridia	O Lachnospirales	F Lachnospiraceae	G GCA-900066575	unclassified G GCA-900066575
SV1207	K Bacteria	P Bacteroidota	C Bacteroidia	O Bacteroidales	F Prevotellaceae	G Prevotellaceae UCG-001	unclassified G Prevotellaceae UCG-001
SV1208	K Bacteria	P Firmicutes	C Clostridia	O Lachnospirales	F Lachnospiraceae	G Lachnospiraceae NK4A136 group	unclassified G Lachnospiraceae NK4A136 group
SV1209	K Bacteria	P Firmicutes	C Clostridia	O Oscillospirales	F [Eubacterium] coprostanoligenes group	unclassified F [Eubacterium] coprostanoligenes grou	unclassified F [Eubacterium] coprostanoligenes group
SV1210	K Bacteria	P Firmicutes	C Clostridia	O Lachnospirales	F Lachnospiraceae	G Murimonas	S intestini
SV1211	K Bacteria	P Firmicutes	C Clostridia	O Oscillospirales	F Oscillospiraceae	G Colidextribacter	unclassified G Colidextribacter
SV1212	K Bacteria	P Bacteroidota	C Bacteroidia	O Bacteroidales	F Muribaculaceae	unclassified F Muribaculaceae	unclassified F Muribaculaceae
SV1213	K Bacteria	P Firmicutes	C Clostridia	O Lachnospirales	F Lachnospiraceae	G [Eubacterium] ventriosum group	unclassified G [Eubacterium] ventriosum group
SV1214	K_Bacteria	P_Firmicutes	C_Clostridia	O_Oscillospirales	F_Ruminococcaceae	G_Incertae Sedis	unclassified_G_Incertae Sedis
SV1215	K Bacteria	P Firmicutes	C Clostridia	O Lachnospirales	F Lachnospiraceae	G Lachnospiraceae NK4A136 group	unclassified G Lachnospiraceae NK4A136 group
SV1216	K Bacteria	P Firmicutes	C Clostridia	O Lachnospirales	F Lachnospiraceae	unclassified F Lachnospiraceae	unclassified F Lachnospiraceae
SV1217	K Bacteria	P Firmicutes	C Clostridia	O Lachnospirales	F Lachnospiraceae	G Lachnospiraceae NK4A136 group	unclassified G Lachnospiraceae NK4A136 group
SV1218	K Bacteria	P Firmicutes	C Clostridia	O Oscillospirales	F Oscillospiraceae	unclassified F Oscillospiraceae	unclassified F Oscillospiraceae
SV1219	K Bacteria	P Firmicutes	C Clostridia	O Lachnospirales	F Lachnospiraceae	G Lachnospiraceae NK4A136 group	unclassified G Lachnospiraceae NK4A136 group
SV1220	K_Bacteria	P_Firmicutes	C_Clostridia	O_Lachnospirales	F_Lachnospiraceae	G_[Eubacterium] xylanophilum group	unclassified_G_[Eubacterium] xylanophilum group
SV1221	K Bacteria	P Firmicutes	C Clostridia	O Oscillospirales	F Oscillospiraceae	unclassified F Oscillospiraceae	unclassified F Oscillospiraceae
SV1222	K Bacteria	P Firmicutes	C Clostridia	O Lachnospirales	F Lachnospiraceae	G Lachnospiraceae UCG-006	unclassified G Lachnospiraceae UCG-006
SV1223	K Bacteria	P Firmicutes	C Clostridia	O Clostridia UCG-014	unclassified O Clostridia UCG-014	unclassified O Clostridia UCG-014	unclassified O Clostridia UCG-014
SV1224	K Bacteria	P Firmicutes	C Clostridia	O Clostridia UCG-014	unclassified O Clostridia UCG-014	unclassified O Clostridia UCG-014	unclassified O Clostridia UCG-014
SV1225	K Bacteria	P Firmicutes	C Clostridia	O Clostridia UCG-014	unclassified O Clostridia UCG-014	unclassified O Clostridia UCG-014	unclassified O Clostridia UCG-014
SV1226	K_Bacteria	P_Bacteroidota	C_Bacteroidia	O_Bacteroidales	F_Muribaculaceae	unclassified_F_Muribaculaceae	unclassified_F_Muribaculaceae
SV1227	K Bacteria	P Firmicutes	C Clostridia	O Peptostreptococcales-Tissierellales	F Anaerovoracaceae	G Family XIII UCG-001	unclassified G Family XIII UCG-001
SV1228	K Bacteria	P Firmicutes	C Clostridia	O Oscillospirales	F Oscillospiraceae	G Colidextribacter	unclassified G Colidextribacter
SV1229	K Bacteria	P Firmicutes	C Clostridia	O Lachnospirales	F Lachnospiraceae	G Lachnospiraceae UCG-006	unclassified G Lachnospiraceae UCG-006
SV1230	K Bacteria	P Firmicutes	C Clostridia	O Oscillospirales	F Ruminococcaceae	G Ruminococcus	unclassified G Ruminococcus
SV1231	K Bacteria	P Firmicutes	C Clostridia	O Lachnospirales	F Lachnospiraceae	unclassified F Lachnospiraceae	unclassified F Lachnospiraceae
SV1232	K_Bacteria	P_Firmicutes	C_Clostridia	O_Lachnospirales	F_Lachnospiraceae	unclassified_F_Lachnospiraceae	unclassified_F_Lachnospiraceae
SV1233	K Bacteria	P Firmicutes	C Clostridia	O Lachnospirales	F Lachnospiraceae	G A2	unclassified G A2
SV1234	K Bacteria	P Firmicutes	C Clostridia	O Lachnospirales	F Lachnospiraceae	G Blautia	unclassified G Blautia
SV1235	K Bacteria	P Firmicutes	C Clostridia	O Oscillospirales	F Ruminococcaceae	unclassified F Ruminococcaceae	unclassified F Ruminococcaceae
SV1236	K Bacteria	P Firmicutes	C Clostridia	O Lachnospirales	F Lachnospiraceae	G Lachnospiraceae NK4A136 group	unclassified G Lachnospiraceae NK4A136 group
SV1237	K Bacteria	P Firmicutes	C Clostridia	O Clostridia UCG-014	unclassified O Clostridia UCG-014	unclassified O Clostridia UCG-014	unclassified O Clostridia UCG-014
SV1238	K Bacteria	P Firmicutes	C Clostridia	O Oscillospirales	F Oscillospiraceae	G Colidextribacter	unclassified G Colidextribacter
SV1239	K Bacteria	P Firmicutes	C Clostridia	O Clostridia UCG-014	unclassified O Clostridia UCG-014	unclassified O Clostridia UCG-014	unclassified O Clostridia UCG-014
SV1240	K Bacteria	P Firmicutes	C Clostridia	O Peptostreptococcales-Tissierellales	F Anaerovoracaceae	G [Eubacterium] nodatum group	unclassified G [Eubacterium] nodatum group

SV1241	K Bacteria	P Firmicutes	C Clostridia	O Clostridia UCG-014	unclassified O Clostridia UCG-014	unclassified O Clostridia UCG-014	unclassified O Clostridia UCG-014
SV1242	K Bacteria	P Firmicutes	C Clostridia	O Oscillospirales	F Oscillospiraceae	G Colidextribacter	unclassified G Colidextribacter
SV1243	K Bacteria	P Firmicutes	C Clostridia	O Lachnospirales	F Lachnospiraceae	G Lachnospiraceae NK4A136 group	unclassified G Lachnospiraceae NK4A136 group
SV1244	K Bacteria	P Actinobacteriota	C Coriobacteriia	O Coriobacteriales	F Eggerthellaceae	G Enterorhabdus	unclassified G Enterorhabdus
SV1245	K Bacteria	P Firmicutes	C Clostridia	O Clostridia UCG-014	unclassified O Clostridia UCG-014	unclassified O Clostridia UCG-014	unclassified O Clostridia UCG-014
SV1246	K Bacteria	P Firmicutes	C Clostridia	O Lachnospirales	F Lachnospiraceae	G Marvinbryantia	unclassified G Marvinbryantia
SV1247	K Bacteria	P Firmicutes	C Clostridia	O Lachnospirales	F Lachnospiraceae	G Lachnospiraceae UCG-001	unclassified G Lachnospiraceae UCG-001
SV1248	K Bacteria	P Firmicutes	C Clostridia	O Lachnospirales	F Lachnospiraceae	G Lachnospiraceae NK4A136 group	unclassified G Lachnospiraceae NK4A136 group
SV1249	K Bacteria	P Firmicutes	C Clostridia	O Oscillospirales	F Oscillospiraceae	G Colidextribacter	unclassified G Colidextribacter
SV1250	K Bacteria	P Firmicutes	C Clostridia	O Lachnospirales	F Lachnospiraceae	unclassified F Lachnospiraceae	unclassified F Lachnospiraceae
SV1251	K Bacteria	P Firmicutes	C Clostridia	O Lachnospirales	F Lachnospiraceae	G Tuzzerella	unclassified G Tuzzerella
SV1252	K Bacteria	P Firmicutes	C Clostridia	O Peptostreptococcales-Tissierellales	F Peptostreptococcaceae	G Romboutsia	unclassified G Romboutsia
SV1253	K Bacteria	P Bacteroidota	C Bacteroidia	O Bacteroidales	F Prevotellaceae	G Prevotellaceae UCG-001	unclassified G Prevotellaceae UCG-001
SV1254	K Bacteria	P Firmicutes	C Clostridia	O Lachnospirales	F Lachnospiraceae	unclassified F Lachnospiraceae	unclassified F Lachnospiraceae
SV1255	K Bacteria	P Bacteroidota	C Bacteroidia	O Bacteroidales	F Prevotellaceae	G Prevotellaceae UCG-001	unclassified G Prevotellaceae UCG-001
SV1256	K Bacteria	P Firmicutes	C Clostridia	O Oscillospirales	F Oscillospiraceae	G Oscillibacter	unclassified G Oscillibacter
SV1257	K Bacteria	P Firmicutes	C Clostridia	O Oscillospirales	F Ruminococcaceae	G Incertae Sedis	unclassified G Incertae Sedis
SV1258	K Bacteria	P Firmicutes	C Clostridia	O Clostridia UCG-014	unclassified O Clostridia UCG-014	unclassified O Clostridia UCG-014	unclassified O Clostridia UCG-014
SV1259	K Bacteria	P Firmicutes	C Clostridia	O Lachnospirales	F Lachnospiraceae	G Lachnospiraceae NK4A136 group	unclassified G Lachnospiraceae NK4A136 group
SV1260	K Bacteria	P Firmicutes	C Clostridia	O Clostridia UCG-014	unclassified O Clostridia UCG-014	unclassified O Clostridia UCG-014	unclassified O Clostridia UCG-014
SV1261	K Bacteria	P Firmicutes	C Clostridia	O Lachnospirales	F Lachnospiraceae	unclassified F Lachnospiraceae	unclassified F Lachnospiraceae
SV1262	K Bacteria	P Firmicutes	C Clostridia	O Clostridia UCG-014	unclassified O Clostridia UCG-014	unclassified O Clostridia UCG-014	unclassified O Clostridia UCG-014
SV1263	K Bacteria	P Firmicutes	C Clostridia	O Clostridia UCG-014	unclassified O Clostridia UCG-014	unclassified O Clostridia UCG-014	unclassified O Clostridia UCG-014
SV1264	K Bacteria	P Firmicutes	C Clostridia	O Lachnospirales	F Lachnospiraceae	G Blautia	unclassified G Blautia
SV1265	K Bacteria	P Firmicutes	C Clostridia	O Lachnospirales	F Lachnospiraceae	unclassified F Lachnospiraceae	unclassified F Lachnospiraceae
SV1266	K Bacteria	P Firmicutes	C Clostridia	O Lachnospirales	F Lachnospiraceae	unclassified F Lachnospiraceae	unclassified F Lachnospiraceae
SV1267	K Bacteria	P Firmicutes	C Clostridia	O Lachnospirales	F Lachnospiraceae	G Roseburia	unclassified G Roseburia
SV1268	K Bacteria	P Firmicutes	C Clostridia	O Clostridia UCG-014	unclassified O Clostridia UCG-014	unclassified O Clostridia UCG-014	unclassified O Clostridia UCG-014
SV1269	K Bacteria	P Firmicutes	C Clostridia	O Clostridiales	F Clostridiaceae	G Clostridium sensu stricto 1	unclassified G Clostridium sensu stricto 1
SV1270	K Bacteria	P Firmicutes	C Clostridia	O Clostridia UCG-014	unclassified O Clostridia UCG-014	unclassified O Clostridia UCG-014	unclassified O Clostridia UCG-014
SV1271	K Bacteria	P Firmicutes	C Clostridia	O Oscillospirales	F Ruminococcaceae	unclassified F Ruminococcaceae	unclassified F Ruminococcaceae
SV1272	K Bacteria	P Firmicutes	C Clostridia	O Lachnospirales	F Lachnospiraceae	unclassified F Lachnospiraceae	unclassified F Lachnospiraceae
SV1273	K Bacteria	P Firmicutes	C Bacilli	O Erysipelotrichales	F Erysipelotrichaceae	G Turicibacter	unclassified G Turicibacter
SV1274	K Bacteria	P Firmicutes	C Clostridia	O Lachnospirales	F Lachnospiraceae	unclassified F Lachnospiraceae	unclassified F Lachnospiraceae
SV1275	K Bacteria	P Firmicutes	C Clostridia	O Clostridia UCG-014	unclassified O Clostridia UCG-014	unclassified O Clostridia UCG-014	unclassified O Clostridia UCG-014
SV1276	K Bacteria	P Firmicutes	C Clostridia	O Peptostreptococcales-Tissierellales	F Peptostreptococcaceae	G Romboutsia	unclassified G Romboutsia
SV1277	K Bacteria	P Firmicutes	C Clostridia	O Oscillospirales	F Oscillospiraceae	G Oscillibacter	unclassified G Oscillibacter
SV1278	K Bacteria	P Firmicutes	C Clostridia	O Lachnospirales	F Lachnospiraceae	unclassified F Lachnospiraceae	unclassified F Lachnospiraceae
SV1279	K Bacteria	P Firmicutes	C Clostridia	O Oscillospirales	F Ruminococcaceae	G Ruminococcus	unclassified G Ruminococcus
SV1280	K Bacteria	P Firmicutes	C Clostridia	O Lachnospirales	F Lachnospiraceae	G Lachnospiraceae NK4A136 group	unclassified G Lachnospiraceae NK4A136 group
SV1281	K Bacteria	P Firmicutes	C Clostridia	O Lachnospirales	F Lachnospiraceae	G Lachnospiraceae NK4A136 group	unclassified G Lachnospiraceae NK4A136 group
SV1282	K Bacteria	P Firmicutes	C Clostridia	O Lachnospirales	F Lachnospiraceae	unclassified F Lachnospiraceae	unclassified F Lachnospiraceae
SV1283	K Bacteria	P Firmicutes	C Clostridia	O Lachnospirales	F Lachnospiraceae	G Lachnospiraceae FCS020 group	unclassified G Lachnospiraceae FCS020 group
SV1284	K Bacteria	P Firmicutes	C Clostridia	O Lachnospirales	F Lachnospiraceae	G Lachnospiraceae FCS020 group	unclassified G Lachnospiraceae FCS020 group
SV1285	K Bacteria	P Firmicutes	C Clostridia	O Lachnospirales	F Lachnospiraceae	unclassified F Lachnospiraceae	unclassified F Lachnospiraceae
SV1286	K Bacteria	P Firmicutes	C Clostridia	O Oscillospirales	F Oscillospiraceae	unclassified F Oscillospiraceae	unclassified F Oscillospiraceae
SV1287	K_Bacteria	P_Firmicutes	C_Clostridia	O_Oscillospirales	F_Ruminococcaceae	unclassified_F_Ruminococcaceae	unclassified_F_Ruminococcaceae
SV1288	K Bacteria	P Firmicutes	C Clostridia	O Lachnospirales	F Lachnospiraceae	G Marvinbryantia	unclassified G Marvinbryantia
SV1289	K Bacteria	P Firmicutes	C Clostridia	O Lachnospirales	F Lachnospiraceae	G Lachnospiraceae UCG-001	unclassified G Lachnospiraceae UCG-001
SV1290	K Bacteria	P Bacteroidota	C Bacteroidia	O Bacteroidales	F Bacteroidaceae	G Bacteroides	unclassified G Bacteroides
SV1291	K Bacteria	P Firmicutes	C Clostridia	O Lachnospirales	F Lachnospiraceae	G [Eubacterium] xylanophilum group	unclassified G [Eubacterium] xylanophilum group
SV1292	K Bacteria	P Firmicutes	C Clostridia	O Oscillospirales	F Oscillospiraceae	G Colidextribacter	unclassified G Colidextribacter
SV1293	K_Bacteria	P_Firmicutes	C_Clostridia	O_Lachnospirales	F_Lachnospiraceae	G_GCA-900066575	unclassified_G_GCA-900066575
SV1294	K Bacteria	P Firmicutes	C Clostridia	O Oscillospirales	F Oscillospiraceae	G Oscillibacter	unclassified G Oscillibacter
SV1295	K Bacteria	P Bacteroidota	C Bacteroidia	O Bacteroidales	F Muribaculaceae	unclassified F Muribaculaceae	unclassified F Muribaculaceae
SV1296	K Bacteria	P Firmicutes	C Clostridia	O Lachnospirales	F Lachnospiraceae	G Lachnoclostridium	unclassified G Lachnoclostridium
SV1297	K Bacteria	P Bacteroidota	C Bacteroidia	O Bacteroidales	F Muribaculaceae	unclassified F Muribaculaceae	unclassified F Muribaculaceae
SV1298	K Bacteria	P Firmicutes	C Clostridia	O Lachnospirales	F Lachnospiraceae	G [Eubacterium] xylanophilum group	unclassified G [Eubacterium] xylanophilum group
SV1299	K_Bacteria	P_Firmicutes	C_Clostridia	O_Lachnospirales	F_Lachnospiraceae	G_Lachnospiraceae NK4A136 group	unclassified_G_Lachnospiraceae NK4A136 group
SV1300	K Bacteria	P Firmicutes	C Clostridia	O Lachnospirales	F Lachnospiraceae	G Lachnoclostridium	unclassified G Lachnoclostridium
SV1301	K Bacteria	P Firmicutes	C Clostridia	O Oscillospirales	F Ruminococcaceae	unclassified F Ruminococcaceae	unclassified F Ruminococcaceae
SV1302	K Bacteria	P Firmicutes	C Clostridia	O Lachnospirales	F Lachnospiraceae	G Lachnospiraceae UCG-001	unclassified G Lachnospiraceae UCG-001
SV1303	K Bacteria	P Firmicutes	C Clostridia	O Clostridia UCG-014	unclassified O Clostridia UCG-014	unclassified O Clostridia UCG-014	unclassified O Clostridia UCG-014
SV1304	K Bacteria	P Firmicutes	C Clostridia	O Clostridiales	F Clostridiaceae	G Clostridium sensu stricto 1	unclassified G Clostridium sensu stricto 1
SV1305	K_Bacteria	P_Bacteroidota	C_Bacteroidia	O_Bacteroidales	F_Rikenellaceae	G_Alistipes	unclassified_G_Alistipes
SV1306	K Bacteria	P Firmicutes	C Clostridia	O Clostridia UCG-014	unclassified O Clostridia UCG-014	unclassified O Clostridia UCG-014	unclassified O Clostridia UCG-014
SV1307	K Bacteria	P Proteobacteria	C Gammaproteobacteria	O Burkholderiales	F Sutterellaceae	G Parasutterella	unclassified G Parasutterella
SV1308	K Bacteria	P Firmicutes	C Clostridia	O Oscillospirales	F Ruminococcaceae	unclassified F Ruminococcaceae	unclassified F Ruminococcaceae
SV1309	K Bacteria	P Firmicutes	C Clostridia	O Lachnospirales	F Lachnospiraceae	G GCA-900066575	unclassified G GCA-900066575
SV1310	K Bacteria	P Firmicutes	C Clostridia	O Oscillospirales	F Ruminococcaceae	G Incertae Sedis	unclassified G Incertae Sedis
SV1311	K_Bacteria	P_Firmicutes	C_Clostridia	O_Lachnospirales	F_Lachnospiraceae	G_Lachnospiraceae NK4A136 group	unclassified_G_Lachnospiraceae NK4A136 group
SV1312	K Bacteria	P Firmicutes	C Clostridia	O Clostridia UCG-014	unclassified O Clostridia UCG-014	unclassified O Clostridia UCG-014	unclassified O Clostridia UCG-014
SV1313	K Bacteria	P Firmicutes	C Clostridia	O Oscillospirales	F Oscillospiraceae	G Colidextribacter	unclassified G Colidextribacter
SV1314	K Bacteria	P Firmicutes	C Clostridia	O Peptostreptococcales-Tissierellales	F Anaerovoracaceae	G Family XIII AD3011 group	unclassified G Family XIII AD3011 group
SV1315	K Bacteria	P Firmicutes	C Clostridia	O Lachnospirales	F Lachnospiraceae	unclassified F Lachnospiraceae	unclassified F Lachnospiraceae
SV1316	K Bacteria	P Firmicutes	C Clostridia	O Lachnospirales	F Lachnospiraceae	G [Eubacterium] xylanophilum group	unclassified G [Eubacterium] xylanophilum group
SV1317	K Bacteria	P Firmicutes	C Clostridia	O Oscillospirales	F Ruminococcaceae	G Incertae Sedis	unclassified G Incertae Sedis
SV1318	K Bacteria	P Firmicutes	C Clostridia	O Oscillospirales	F Oscillospiraceae	G Oscillibacter	unclassified G Oscillibacter
SV1319	K Bacteria	P Bacteroidota	C Bacteroidia	O Bacteroidales	F Muribaculaceae	unclassified F Muribaculaceae	unclassified F Muribaculaceae
SV1320	K Bacteria	P Firmicutes	C Bacilli	O RF39	unclassified O RF39	unclassified O RF39	unclassified O RF39
SV1321	K Bacteria	P Firmicutes	C Clostridia	O Lachnospirales	F Lachnospiraceae	G Roseburia	unclassified G Roseburia
SV1322	K Bacteria	P Firmicutes	C Clostridia	O Oscillospirales	F Oscillospiraceae	G Colidextribacter	unclassified G Colidextribacter
SV1323	K Bacteria	P Firmicutes	C Clostridia	O Clostridia UCG-014	unclassified O Clostridia UCG-014	unclassified O Clostridia UCG-014	unclassified O Clostridia UCG-014
SV1324	K Bacteria	P Bacteroidota	C Bacteroidia	O Bacteroidales	F Bacteroidaceae	G Bacteroides	unclassified G Bacteroides
SV1325	K Bacteria	P Bacteroidota	C Bacteroidia	O Bacteroidales	F Bacteroidaceae	G Bacteroides	unclassified G Bacteroides
SV1326	K Bacteria	P Firmicutes	C Clostridia	O Oscillospirales	F Oscillospiraceae	unclassified F Oscillospiraceae	unclassified F Oscillospiraceae
SV1327	K Bacteria	P Firmicutes	C Clostridia	O Lachnospirales	F Lachnospiraceae	G A2	unclassified G A2
SV1328	K Bacteria	P Firmicutes	C Clostridia	O Lachnospirales	F Lachnospiraceae	G [Eubacterium] ruminantium group	unclassified G [Eubacterium] ruminantium group
SV1329	K Bacteria	P Firmicutes	C Clostridia	O Lachnospirales	F Lachnospiraceae	G GCA-900066575	unclassified G GCA-900066575
SV1330	K Bacteria	P Cyanobacteria	C Vampirivibrionia	O Gastranaerophilales	unclassified O Gastranaerophilales	unclassified O Gastranaerophilales	unclassified O Gastranaerophilales
SV1331	K Bacteria	P Firmicutes	C Clostridia	O Lachnospirales	F Lachnospiraceae	unclassified F Lachnospiraceae	unclassified F Lachnospiraceae
SV1332	K Bacteria	P Firmicutes	C Clostridia	O Lachnospirales	F Lachnospiraceae	G A2	unclassified G A2
SV1333	K Bacteria	P Firmicutes	C Clostridia	O Clostridia vadinBB60 group	unclassified O Clostridia vadinBB60 group	unclassified O Clostridia vadinBB60 group	unclassified O Clostridia vadinBB60 group
SV1334	K Bacteria	P Firmicutes	C Clostridia	O Lachnospirales	F Lachnospiraceae	G Lachnospiraceae NK4A136 group	unclassified G Lachnospiraceae NK4A136 group
SV1335	K Bacteria	P Firmicutes	C Clostridia	O Oscillospirales	F Butyricicoccaceae	G Butyricicoccus	unclassified G Butyricicoccus
SV1336	K Bacteria	P Firmicutes	C Clostridia	O Lachnospirales	F Lachnospiraceae	unclassified F Lachnospiraceae	unclassified F Lachnospiraceae
SV1337	K Bacteria	P Firmicutes	C Clostridia	O Oscillospirales	F Ruminococcaceae	unclassified F Ruminococcaceae	unclassified F Ruminococcaceae
SV1338	K Bacteria	P Firmicutes	C Bacilli	O Lactobacillales	F Lactobacillaceae	unclassified F Lactobacillaceae	unclassified F Lactobacillaceae
SV1339	K Bacteria	P Firmicutes	C Clostridia	O Oscillospirales	F Oscillospiraceae	G Oscillibacter	unclassified G Oscillibacter
SV1340	K Bacteria	P Firmicutes	C Clostridia	O Clostridia UCG-014	unclassified O Clostridia UCG-014	unclassified O Clostridia UCG-014	unclassified O Clostridia UCG-014
SV1341	K Bacteria	P Firmicutes	C Clostridia	O Lachnospirales	F Lachnospiraceae	G Lachnospiraceae NK4A136 group	unclassified G Lachnospiraceae NK4A136 group
SV1342	K Bacteria	P Firmicutes	C Clostridia	O Clostridia UCG-014	unclassified O Clostridia UCG-014	unclassified O Clostridia UCG-014	unclassified O Clostridia UCG-014
SV1343	K Bacteria	P Firmicutes	C Clostridia	O Peptostreptococcales-Tissierellales	F Anaerovoracaceae	G Anaerovorax	unclassified G Anaerovorax
SV1344	K Bacteria	P Bacteroidota	C Bacteroidia	O Bacteroidales	F Rikenellaceae	G Alistipes	unclassified G Alistipes
SV1345	K Bacteria	P Firmicutes	C Clostridia	O Clostridiales	F Clostridiaceae	G Clostridium sensu stricto 1	unclassified G Clostridium sensu stricto 1
SV1346	K Bacteria	P Firmicutes	C Clostridia	O Lachnospirales	F Lachnospiraceae	unclassified F Lachnospiraceae	unclassified F Lachnospiraceae
SV1347	K Bacteria	P Firmicutes	C Bacilli	O Erysipelotrichales	F Erysipelatoclostridiaceae	unclassified F Erysipelatoclostridiaceae	unclassified F Erysipelatoclostridiaceae
SV1348	K Bacteria	P Firmicutes	C Clostridia	O Oscillospirales	F Oscillospiraceae	G Colidextribacter	unclassified G Colidextribacter
SV1349	K Bacteria	P Firmicutes	C Clostridia	O Oscillospirales	F Oscillospiraceae	unclassified F Oscillospiraceae	unclassified F Oscillospiraceae
SV1350	K Bacteria	P Actinobacteriota	C Coriobacteriia	O Coriobacteriales	F Eggerthellaceae	G Enterorhabdus	unclassified G Enterorhabdus
SV1351	K Bacteria	P Firmicutes	C Clostridia	O Clostridia UCG-014	unclassified O Clostridia UCG-014	unclassified O Clostridia UCG-014	unclassified O Clostridia UCG-014
SV1352	K_Bacteria	P_Firmicutes	C_Clostridia	O_Lachnospirales	F_Lachnospiraceae	G_Marvinbryantia	unclassified_G_Marvinbryantia
SV1353	K Bacteria	P Firmicutes	C Clostridia	O Clostridia UCG-014	unclassified O Clostridia UCG-014	unclassified O Clostridia UCG-014	unclassified O Clostridia UCG-014
SV1354	K Bacteria	P Firmicutes	C Clostridia	O Clostridia UCG-014	unclassified O Clostridia UCG-014	unclassified O Clostridia UCG-014	unclassified O Clostridia UCG-014
SV1355	K Bacteria	P Firmicutes	C Clostridia	O Lachnospirales	F Lachnospiraceae	G Roseburia	unclassified G Roseburia
SV1356	K Bacteria	P Firmicutes	C Clostridia	O Oscillospirales	F Oscillospiraceae	unclassified F Oscillospiraceae	unclassified F Oscillospiraceae
SV1357	K Bacteria	P Firmicutes	C Clostridia	O Lachnospirales	F Lachnospiraceae	G Roseburia	unclassified G Roseburia
SV1358	K_Bacteria	P_Firmicutes	C_Clostridia	O_Lachnospirales	F_Lachnospiraceae	G_ASF356	unclassified_G_ASF356
SV1359	K Bacteria	P Firmicutes	C Clostridia	O Lachnospirales	F Lachnospiraceae	unclassified F Lachnospiraceae	unclassified F Lachnospiraceae
SV1360	K Bacteria	P Firmicutes	C Bacilli	O Lactobacillales	F Lactobacillaceae	G Ligilactobacillus	unclassified G Ligilactobacillus
SV1361	K Bacteria	P Firmicutes	C Clostridia	O Lachnospirales	F Lachnospiraceae	unclassified F Lachnospiraceae	unclassified F Lachnospiraceae
SV1362	K Bacteria	P Firmicutes	C Clostridia	O Oscillospirales	F Oscillospiraceae	unclassified F Oscillospiraceae	unclassified F Oscillospiraceae
SV1363	K Bacteria	P Firmicutes	C Clostridia	O Oscillospirales	F Oscillospiraceae	G Oscillibacter	unclassified G Oscillibacter
SV1364	K_Bacteria	P_Firmicutes	C_Clostridia	O_Lachnospirales	F_Lachnospiraceae	G_Lachnospiraceae NK4A136 group	S_bacterium
SV1365	K Bacteria	P Actinobacteriota	C Coriobacteriia	O Coriobacteriales	F Eggerthellaceae	G Enterorhabdus	unclassified G Enterorhabdus
SV1366	K Bacteria	P Firmicutes	C Clostridia	O Clostridia vadinBB60 group	unclassified O Clostridia vadinBB60 group	unclassified O Clostridia vadinBB60 group	unclassified O Clostridia vadinBB60 group
SV1367	K Bacteria	P Firmicutes	C Bacilli	O Erysipelotrichales	F Erysipelotrichaceae	G Turicibacter	unclassified G Turicibacter
SV1368	K Bacteria	P Firmicutes	C Clostridia	O Lachnospirales	F Lachnospiraceae	G Lachnospiraceae UCG-001	unclassified G Lachnospiraceae UCG-001
SV1369	K Bacteria	P Firmicutes	C Clostridia	O Oscillospirales	F Oscillospiraceae	G Intestinimonas	unclassified G Intestinimonas
SV1370	K_Bacteria	P_Firmicutes	C_Clostridia	O_Lachnospirales	F_Lachnospiraceae	unclassified_F_Lachnospiraceae	unclassified_F_Lachnospiraceae
SV1371	K Bacteria	P Firmicutes	C Clostridia	O Oscillospirales	F Ruminococcaceae	G Paludicola	unclassified G Paludicola
SV1372	K Bacteria	P Firmicutes	C Clostridia	O Oscillospirales	F Oscillospiraceae	unclassified F Oscillospiraceae	unclassified F Oscillospiraceae
SV1373	K Bacteria	P Firmicutes	C Clostridia	O Lachnospirales	F Lachnospiraceae	unclassified F Lachnospiraceae	unclassified F Lachnospiraceae
SV1374	K Bacteria	P Firmicutes	C Clostridia	O Lachnospirales	F Lachnospiraceae	G Lachnospiraceae FCS020 group	unclassified G Lachnospiraceae FCS020 group
SV1375	K Bacteria	P Bacteroidota	C Bacteroidia	O Bacteroidales	F Muribaculaceae	unclassified F Muribaculaceae	unclassified F Muribaculaceae
SV1376	K Bacteria	P Firmicutes	C Clostridia	O Lachnospirales	F Lachnospiraceae	G Dorea	unclassified G Dorea
SV1377	K Bacteria	P Firmicutes	C Clostridia	O Oscillospirales	F Oscillospiraceae	G Colidextribacter	unclassified G Colidextribacter
SV1378	K Bacteria	P Firmicutes	C Clostridia	O Lachnospirales	F Lachnospiraceae	unclassified F Lachnospiraceae	unclassified F Lachnospiraceae

SV1379	K_Bacteria	P_Firmicutes	C_Clostridia	O_Lachnospirales	F_Lachnospiraceae	G_Lachnospiraceae NK4A136 group	unclassified_G_Lachnospiraceae NK4A136 group
SV1380	K_Bacteria	P_Bacteroidota	C_Bacteroidia	O_Bacteroidales	F_Muribaculaceae	unclassified_F_Muribaculaceae	unclassified_F_Muribaculaceae
SV1381	K_Bacteria	P_Firmicutes	C_Clostridia	O_Lachnospirales	F_Lachnospiraceae	unclassified_F_Lachnospiraceae	unclassified_F_Lachnospiraceae
SV1382	K_Bacteria	P_Firmicutes	C_Clostridia	O_Lachnospirales	F_Lachnospiraceae	G_Lachnospiraceae NK4A136 group	unclassified_G_Lachnospiraceae NK4A136 group
SV1383	K_Bacteria	P_Bacteroidota	C_Bacteroidia	O_Bacteroidales	F_Muribaculaceae	unclassified_F_Muribaculaceae	unclassified_F_Muribaculaceae
SV1384	K_Bacteria	P_Firmicutes	C_Clostridia	O_Lachnospirales	F_Lachnospiraceae	unclassified_F_Lachnospiraceae	unclassified_F_Lachnospiraceae
SV1385	K_Bacteria	P_Actinobacteriota	C_Coriobacteriia	O_Coriobacteriales	F_Eggerthellaceae	G_Enterorhabdus	unclassified_G_Enterorhabdus
SV1386	K_Bacteria	P_Firmicutes	C_Clostridia	O_Lachnospirales	F_Lachnospiraceae	G_Blautia	unclassified_G_Blautia
SV1387	K_Bacteria	P_Firmicutes	C_Clostridia	O_Clostridia vadinBB60 group	unclassified_O_Clostridia vadinBB60 group	unclassified_O_Clostridia vadinBB60 group	unclassified_O_Clostridia vadinBB60 group
SV1388	K_Bacteria	P_Actinobacteriota	C_Coriobacteriia	O_Coriobacteriales	F_Eggerthellaceae	G_Enterorhabdus	unclassified_G_Enterorhabdus
SV1389	K_Bacteria	P_Firmicutes	C_Clostridia	O_Lachnospirales	F_Lachnospiraceae	G_Roseburia	unclassified_G_Roseburia
SV1390	K_Bacteria	P_Bacteroidota	C_Bacteroidia	O_Bacteroidales	F_Muribaculaceae	unclassified_F_Muribaculaceae	unclassified_F_Muribaculaceae
SV1391	K_Bacteria	P_Firmicutes	C_Clostridia	O_Lachnospirales	F_Lachnospiraceae	G_Lachnospiraceae UCG-001	unclassified_G_Lachnospiraceae UCG-001
SV1392	K_Bacteria	P_Firmicutes	C_Clostridia	O_Lachnospirales	F_Lachnospiraceae	G_Lachnospiraceae UCG-001	unclassified_G_Lachnospiraceae UCG-001
SV1393	K_Bacteria	P_Firmicutes	C_Clostridia	O_Lachnospirales	F_Lachnospiraceae	unclassified_F_Lachnospiraceae	unclassified_F_Lachnospiraceae
SV1394	K_Bacteria	P_Firmicutes	C_Clostridia	O_Clostridiales	F_Clostridiaceae	G_Clostridium sensu stricto 1	unclassified_G_Clostridium sensu stricto 1
SV1395	K_Bacteria	P_Firmicutes	C_Clostridia	O_Lachnospirales	F_Lachnospiraceae	unclassified_F_Lachnospiraceae	unclassified_F_Lachnospiraceae
SV1396	K_Bacteria	P_Firmicutes	C_Clostridia	O_Lachnospirales	F_Lachnospiraceae	G_Roseburia	unclassified_G_Roseburia
SV1397	K_Bacteria	P_Firmicutes	C_Clostridia	O_Lachnospirales	F_Lachnospiraceae	G_Lachnospiraceae UCG-001	unclassified_G_Lachnospiraceae UCG-001
SV1398	K_Bacteria	P_Bacteroidota	C_Bacteroidia	O_Bacteroidales	F_Bacteroidaceae	G_Bacteroides	unclassified_G_Bacteroides
SV1399	K_Bacteria	P_Bacteroidota	C_Bacteroidia	O_Bacteroidales	F_Muribaculaceae	unclassified_F_Muribaculaceae	unclassified_F_Muribaculaceae
SV1400	K_Bacteria	P_Firmicutes	C_Clostridia	O_Oscillospirales	F_Oscillospiraceae	unclassified_F_Oscillospiraceae	unclassified_F_Oscillospiraceae
SV1401	K_Bacteria	P_Firmicutes	C_Clostridia	O_Clostridia UCG-014	unclassified_O_Clostridia UCG-014	unclassified_O_Clostridia UCG-014	unclassified_O_Clostridia UCG-014
SV1402	K_Bacteria	P_Bacteroidota	C_Bacteroidia	O_Bacteroidales	F_Muribaculaceae	unclassified_F_Muribaculaceae	unclassified_F_Muribaculaceae
SV1403	K_Bacteria	P_Firmicutes	C_Clostridia	O_Lachnospirales	F_Lachnospiraceae	unclassified_F_Lachnospiraceae	unclassified_F_Lachnospiraceae
SV1404	K_Bacteria	P_Firmicutes	C_Clostridia	O_Lachnospirales	F_Lachnospiraceae	G_Lachnospiraceae UCG-001	unclassified_G_Lachnospiraceae UCG-001
SV1405	K_Bacteria	P_Firmicutes	unclassified_P_Firmicutes	unclassified_P_Firmicutes	unclassified_P_Firmicutes	unclassified_P_Firmicutes	unclassified_P_Firmicutes
SV1406	K_Bacteria	P_Firmicutes	C_Clostridia	O_Oscillospirales	F_Oscillospiraceae	unclassified_F_Oscillospiraceae	unclassified_F_Oscillospiraceae
SV1407	K_Bacteria	P_Firmicutes	C_Clostridia	O_Lachnospirales	F_Lachnospiraceae	G_Lachnoclostridium	unclassified_G_Lachnoclostridium
SV1408	K_Bacteria	P_Firmicutes	C_Clostridia	O_Clostridia UCG-014	unclassified_O_Clostridia UCG-014	unclassified_O_Clostridia UCG-014	unclassified_O_Clostridia UCG-014
SV1409	K_Bacteria	P_Bacteroidota	C_Bacteroidia	O_Bacteroidales	F_Muribaculaceae	unclassified_F_Muribaculaceae	unclassified_F_Muribaculaceae
SV1410	K_Bacteria	P_Firmicutes	C_Clostridia	O_Lachnospirales	F_Lachnospiraceae	G_Lachnospiraceae NK4A136 group	unclassified_G_Lachnospiraceae NK4A136 group
SV1411	K_Bacteria	P_Firmicutes	C_Clostridia	O_Peptostreptococcales-Tissierellales	F_Anaerovoracaceae	G_Family XIII UCG-001	unclassified_G_Family XIII UCG-001
SV1412	K_Bacteria	P_Firmicutes	C_Clostridia	O_Lachnospirales	F_Lachnospiraceae	G_Lachnospiraceae NK4A136 group	unclassified_G_Lachnospiraceae NK4A136 group
SV1413	K_Bacteria	P_Firmicutes	C_Clostridia	O_Clostridia UCG-014	unclassified_O_Clostridia UCG-014	unclassified_O_Clostridia UCG-014	unclassified_O_Clostridia UCG-014
SV1414	K_Bacteria	P_Firmicutes	C_Clostridia	O_Oscillospirales	F_Oscillospiraceae	unclassified_F_Oscillospiraceae	unclassified_F_Oscillospiraceae
SV1415	K_Bacteria	P_Firmicutes	C_Clostridia	O_Clostridia UCG-014	unclassified_O_Clostridia UCG-014	unclassified_O_Clostridia UCG-014	unclassified_O_Clostridia UCG-014
SV1416	K_Bacteria	P_Proteobacteria	C_Gammaproteobacteria	O_Enterobacterales	F_Enterobacteriaceae	G_Escherichia-Shigella	unclassified_G_Escherichia-Shigella
SV1417	K_Bacteria	P_Firmicutes	C_Clostridia	O_Lachnospirales	F_Lachnospiraceae	G_Lachnospiraceae NK4A136 group	unclassified_G_Lachnospiraceae NK4A136 group
SV1418	K_Bacteria	P_Firmicutes	C_Clostridia	O_Lachnospirales	F_Lachnospiraceae	G_Lachnospiraceae NK4A136 group	unclassified_G_Lachnospiraceae NK4A136 group
SV1419	K_Bacteria	P_Firmicutes	C_Clostridia	O_Lachnospirales	F_Lachnospiraceae	unclassified_F_Lachnospiraceae	unclassified_F_Lachnospiraceae
SV1420	K_Bacteria	P_Firmicutes	C_Clostridia	O_Lachnospirales	F_Lachnospiraceae	unclassified_F_Lachnospiraceae	unclassified_F_Lachnospiraceae
SV1421	K_Bacteria	P_Firmicutes	C_Clostridia	O_Lachnospirales	F_Lachnospiraceae	G_Lachnospiraceae UCG-001	unclassified_G_Lachnospiraceae UCG-001
SV1422	K_Bacteria	P_Bacteroidota	C_Bacteroidia	O_Bacteroidales	F_Muribaculaceae	unclassified_F_Muribaculaceae	unclassified_F_Muribaculaceae
SV1423	K_Bacteria	P_Firmicutes	C_Clostridia	O_Lachnospirales	F_Lachnospiraceae	unclassified_F_Lachnospiraceae	unclassified_F_Lachnospiraceae
SV1424	K_Bacteria	P_Firmicutes	C_Clostridia	O_Lachnospirales	F_Lachnospiraceae	G_GCA-900066575	unclassified_G_GCA-900066575
SV1425	K_Bacteria	P_Firmicutes	C_Clostridia	O_Oscillospirales	F_Oscillospiraceae	G_Oscillibacter	unclassified_G_Oscillibacter
SV1426	K_Bacteria	P_Firmicutes	C_Clostridia	O_Oscillospirales	F_Oscillospiraceae	G_Colidextribacter	unclassified_G_Colidextribacter
SV1427	K_Bacteria	P_Firmicutes	C_Clostridia	O_Oscillospirales	F_Butyricicoccaceae	G_Butyricicoccus	unclassified_G_Butyricicoccus
SV1428	K_Bacteria	P_Firmicutes	C_Clostridia	O_Clostridia UCG-014	unclassified_O_Clostridia UCG-014	unclassified_O_Clostridia UCG-014	unclassified_O_Clostridia UCG-014
SV1429	K_Bacteria	P_Bacteroidota	C_Bacteroidia	O_Bacteroidales	F_Muribaculaceae	unclassified_F_Muribaculaceae	unclassified_F_Muribaculaceae
SV1430	K_Bacteria	P_Firmicutes	C_Clostridia	O_Lachnospirales	F_Lachnospiraceae	unclassified_F_Lachnospiraceae	unclassified_F_Lachnospiraceae
SV1431	K_Bacteria	P_Firmicutes	C_Clostridia	O_Lachnospirales	F_Lachnospiraceae	unclassified_F_Lachnospiraceae	unclassified_F_Lachnospiraceae
SV1432	K_Bacteria	P_Firmicutes	C_Clostridia	O_Oscillospirales	F_Ruminococcaceae	unclassified_F_Ruminococcaceae	unclassified_F_Ruminococcaceae
SV1433	K_Bacteria	P_Firmicutes	C_Clostridia	O_Lachnospirales	F_Lachnospiraceae	G_A2	unclassified_G_A2
SV1434	K_Bacteria	P_Firmicutes	C_Clostridia	O_Lachnospirales	F_Lachnospiraceae	G_Eisenbergiella	unclassified_G_Eisenbergiella
SV1435	K_Bacteria	P_Bacteroidota	C_Bacteroidia	O_Bacteroidales	F_Rikenellaceae	G_Alistipes	unclassified_G_Alistipes
SV1436	K_Bacteria	P_Cyanobacteria	C_Cyanobacteriia	O_Chloroplast	unclassified_O_Chloroplast	unclassified_O_Chloroplast	unclassified_O_Chloroplast
SV1437	K_Bacteria	P_Firmicutes	C_Clostridia	O_Oscillospirales	F_Oscillospiraceae	G_Intestinimonas	unclassified_G_Intestinimonas
SV1438	K_Bacteria	P_Firmicutes	C_Clostridia	O_Peptostreptococcales-Tissierellales	F_Anaerovoracaceae	G_Family XIII AD3011 group	unclassified_G_Family XIII AD3011 group
SV1439	K_Bacteria	P_Firmicutes	C_Clostridia	O_Oscillospirales	F_Oscillospiraceae	unclassified_F_Oscillospiraceae	unclassified_F_Oscillospiraceae
SV1440	K_Bacteria	P_Bacteroidota	C_Bacteroidia	O_Bacteroidales	F_Bacteroidaceae	G_Bacteroides	unclassified_G_Bacteroides
SV1441	K_Bacteria	P_Firmicutes	C_Clostridia	O_Lachnospirales	F_Lachnospiraceae	unclassified_F_Lachnospiraceae	unclassified_F_Lachnospiraceae
SV1442	K_Bacteria	P_Firmicutes	C_Clostridia	O_Lachnospirales	F_Lachnospiraceae	G_Roseburia	unclassified_G_Roseburia
SV1443	K_Bacteria	P_Firmicutes	C_Clostridia	O_Clostridia UCG-014	unclassified_O_Clostridia UCG-014	unclassified_O_Clostridia UCG-014	unclassified_O_Clostridia UCG-014
SV1444	K_Bacteria	P_Firmicutes	C_Clostridia	O_Lachnospirales	F_Lachnospiraceae	G_Roseburia	unclassified_G_Roseburia
SV1445	K_Bacteria	P_Firmicutes	C_Clostridia	O_Lachnospirales	F_Lachnospiraceae	G_Lachnospiraceae UCG-001	unclassified_G_Lachnospiraceae UCG-001
SV1446	K_Bacteria	P_Firmicutes	C_Clostridia	O_Oscillospirales	F_Ruminococcaceae	G_Paludicola	unclassified_G_Paludicola
SV1447	K_Bacteria	P_Firmicutes	C_Clostridia	O_Lachnospirales	F_Lachnospiraceae	G_Roseburia	unclassified_G_Roseburia
SV1448	K_Bacteria	P_Firmicutes	C_Clostridia	O_Lachnospirales	F_Lachnospiraceae	unclassified_F_Lachnospiraceae	unclassified_F_Lachnospiraceae
SV1449	K_Bacteria	P_Bacteroidota	C_Bacteroidia	O_Bacteroidales	F_Muribaculaceae	unclassified_F_Muribaculaceae	unclassified_F_Muribaculaceae
SV1450	K_Bacteria	P_Bacteroidota	C_Bacteroidia	O_Bacteroidales	F_Muribaculaceae	unclassified_F_Muribaculaceae	unclassified_F_Muribaculaceae
SV1451	K_Bacteria	P_Firmicutes	C_Clostridia	O_Christensenellales	F_Christensenellaceae	G_Christensenellaceae R-7 group	unclassified_G_Christensenellaceae R-7 group
SV1452	K_Bacteria	P_Firmicutes	C_Clostridia	O_Lachnospirales	F_Lachnospiraceae	unclassified_F_Lachnospiraceae	unclassified_F_Lachnospiraceae
SV1453	K_Bacteria	P_Firmicutes	C_Clostridia	O_Lachnospirales	F_Lachnospiraceae	G_Lachnospiraceae FCS020 group	unclassified_G_Lachnospiraceae FCS020 group
SV1454	K_Bacteria	P_Firmicutes	C_Clostridia	O_Lachnospirales	F_Lachnospiraceae	G_Marvinbryantia	unclassified_G_Marvinbryantia
SV1455	K_Bacteria	P_Firmicutes	C_Bacilli	O_Erysipelotrichales	F_Erysipelatoclostridiaceae	G_Erysipelatoclostridium	unclassified_G_Erysipelatoclostridium
SV1456	K_Bacteria	P_Firmicutes	C_Clostridia	O_Clostridia vadinBB60 group	unclassified_O_Clostridia vadinBB60 group	unclassified_O_Clostridia vadinBB60 group	unclassified_O_Clostridia vadinBB60 group
SV1457	K_Bacteria	P_Firmicutes	C_Clostridia	O_Lachnospirales	F_Lachnospiraceae	G_[Eubacterium] ventriosum group	unclassified_G_[Eubacterium] ventriosum group
SV1458	K_Bacteria	P_Bacteroidota	C_Bacteroidia	O_Bacteroidales	F_Muribaculaceae	unclassified_F_Muribaculaceae	unclassified_F_Muribaculaceae
SV1459	K_Bacteria	P_Bacteroidota	C_Bacteroidia	O_Bacteroidales	F_Bacteroidaceae	G_Bacteroides	unclassified_G_Bacteroides
SV1460	K_Bacteria	P_Firmicutes	C_Clostridia	O_Christensenellales	F_Christensenellaceae	G_Christensenellaceae R-7 group	unclassified_G_Christensenellaceae R-7 group
SV1461	K_Bacteria	P_Firmicutes	C_Clostridia	O_Lachnospirales	F_Lachnospiraceae	unclassified_F_Lachnospiraceae	unclassified_F_Lachnospiraceae
SV1462	K_Bacteria	P_Firmicutes	C_Clostridia	O_Lachnospirales	F_Lachnospiraceae	G_GCA-900066575	unclassified_G_GCA-900066575
SV1463	K_Bacteria	P_Bacteroidota	C_Bacteroidia	O_Bacteroidales	F_Rikenellaceae	G_Rikenellaceae RC9 gut group	unclassified_G_Rikenellaceae RC9 gut group
SV1464	K_Bacteria	P_Firmicutes	C_Clostridia	O_Lachnospirales	F_Lachnospiraceae	G_GCA-900066575	unclassified_G_GCA-900066575
SV1465	K_Bacteria	P_Firmicutes	C_Clostridia	O_Oscillospirales	F_Ruminococcaceae	G_Incertae Sedis	unclassified_G_Incertae Sedis
SV1466	K_Bacteria	P_Firmicutes	C_Bacilli	O_Erysipelotrichales	F_Erysipelotrichaceae	G_Turicibacter	unclassified_G_Turicibacter
SV1467	K_Bacteria	P_Bacteroidota	C_Bacteroidia	O_Bacteroidales	F_Muribaculaceae	unclassified_F_Muribaculaceae	unclassified_F_Muribaculaceae
SV1468	K_Bacteria	P_Firmicutes	C_Clostridia	O_Lachnospirales	F_Lachnospiraceae	unclassified_F_Lachnospiraceae	unclassified_F_Lachnospiraceae
SV1469	K_Bacteria	P_Firmicutes	C_Clostridia	O_Lachnospirales	F_Lachnospiraceae	G_Lachnospiraceae FCS020 group	unclassified_G_Lachnospiraceae FCS020 group
SV1470	K_Bacteria	P_Firmicutes	C_Clostridia	O_Clostridia UCG-014	unclassified_O_Clostridia UCG-014	unclassified_O_Clostridia UCG-014	unclassified_O_Clostridia UCG-014
SV1471	K_Bacteria	P_Firmicutes	C_Clostridia	O_Peptostreptococcales-Tissierellales	F_Anaerovoracaceae	G_Family XIII AD3011 group	unclassified_G_Family XIII AD3011 group
SV1472	K_Bacteria	P_Firmicutes	C_Clostridia	O_Oscillospirales	F_Ruminococcaceae	G_Anaerotruncus	unclassified_G_Anaerotruncus
SV1473	K_Bacteria	P_Firmicutes	C_Clostridia	O_Lachnospirales	F_Lachnospiraceae	unclassified_F_Lachnospiraceae	unclassified_F_Lachnospiraceae
SV1474	K_Bacteria	P_Firmicutes	C_Clostridia	O_Lachnospirales	F_Lachnospiraceae	G_A2	unclassified_G_A2
SV1475	K_Bacteria	P_Firmicutes	C_Clostridia	O_Lachnospirales	F_Lachnospiraceae	unclassified_F_Lachnospiraceae	unclassified_F_Lachnospiraceae
SV1476	K_Bacteria	P_Firmicutes	C_Clostridia	O_Lachnospirales	F_Lachnospiraceae	unclassified_F_Lachnospiraceae	unclassified_F_Lachnospiraceae
SV1477	K_Bacteria	P_Firmicutes	C_Clostridia	O_Lachnospirales	F_Lachnospiraceae	G_Lachnoclostridium	unclassified_G_Lachnoclostridium
SV1478	K_Bacteria	P_Bacteroidota	C_Bacteroidia	O_Bacteroidales	F_Muribaculaceae	unclassified_F_Muribaculaceae	unclassified_F_Muribaculaceae
SV1479	K_Bacteria	P_Firmicutes	C_Clostridia	O_Lachnospirales	F_Lachnospiraceae	G_Blautia	unclassified_G_Blautia
SV1480	K_Bacteria	P_Firmicutes	C_Clostridia	O_Oscillospirales	F_Ruminococcaceae	G_Incertae Sedis	unclassified_G_Incertae Sedis
SV1481	K_Bacteria	P_Firmicutes	C_Clostridia	O_Clostridia UCG-014	unclassified_O_Clostridia UCG-014	unclassified_O_Clostridia UCG-014	unclassified_O_Clostridia UCG-014
SV1482	K_Bacteria	P_Firmicutes	C_Clostridia	O_Lachnospirales	F_Lachnospiraceae	G_Lachnospiraceae UCG-001	unclassified_G_Lachnospiraceae UCG-001
SV1483	K_Bacteria	P_Bacteroidota	C_Bacteroidia	O_Bacteroidales	F_Bacteroidaceae	G_Bacteroides	unclassified_G_Bacteroides
SV1484	K_Bacteria	P_Bacteroidota	C_Bacteroidia	O_Bacteroidales	F_Rikenellaceae	G_Alistipes	unclassified_G_Alistipes
SV1485	K_Bacteria	P_Firmicutes	C_Clostridia	O_Lachnospirales	F_Lachnospiraceae	unclassified_F_Lachnospiraceae	unclassified_F_Lachnospiraceae
SV1486	K_Bacteria	P_Firmicutes	C_Clostridia	O_Lachnospirales	F_Lachnospiraceae	unclassified_F_Lachnospiraceae	unclassified_F_Lachnospiraceae
SV1487	K_Bacteria	P_Firmicutes	C_Clostridia	O_Clostridia vadinBB60 group	unclassified_O_Clostridia vadinBB60 group	unclassified_O_Clostridia vadinBB60 group	unclassified_O_Clostridia vadinBB60 group
SV1488	K_Bacteria	P_Firmicutes	C_Clostridia	O_Oscillospirales	F_Oscillospiraceae	unclassified_F_Oscillospiraceae	unclassified_F_Oscillospiraceae
SV1489	K_Bacteria	P_Firmicutes	C_Clostridia	O_Oscillospirales	F_Oscillospiraceae	G_Colidextribacter	unclassified_G_Colidextribacter
SV1490	K_Bacteria	P_Firmicutes	C_Clostridia	O_Lachnospirales	F_Lachnospiraceae	G_Lachnoclostridium	unclassified_G_Lachnoclostridium
SV1491	K_Bacteria	P_Actinobacteriota	C_Coriobacteriia	O_Coriobacteriales	F_Eggerthellaceae	G_Enterorhabdus	unclassified_G_Enterorhabdus
SV1492	K_Bacteria	P_Firmicutes	C_Clostridia	O_Oscillospirales	F_Ruminococcaceae	unclassified_F_Ruminococcaceae	unclassified_F_Ruminococcaceae
SV1493	K_Bacteria	P_Firmicutes	C_Clostridia	O_Clostridia vadinBB60 group	unclassified_O_Clostridia vadinBB60 group	unclassified_O_Clostridia vadinBB60 group	unclassified_O_Clostridia vadinBB60 group
SV1494	K_Bacteria	P_Firmicutes	C_Clostridia	O_Lachnospirales	F_Lachnospiraceae	unclassified_F_Lachnospiraceae	unclassified_F_Lachnospiraceae
SV1495	K_Bacteria	P_Firmicutes	C_Clostridia	O_Clostridiales	F_Clostridiaceae	G_Clostridium sensu stricto 1	unclassified_G_Clostridium sensu stricto 1
SV1496	K_Bacteria	P_Firmicutes	C_Bacilli	O_Lactobacillales	F_Lactobacillaceae	G_HT002	unclassified_G_HT002
SV1497	K_Bacteria	P_Firmicutes	C_Clostridia	O_Clostridiales	F_Clostridiaceae	G_Clostridium sensu stricto 1	unclassified_G_Clostridium sensu stricto 1
SV1498	K_Bacteria	P_Firmicutes	C_Clostridia	O_Oscillospirales	F_Butyricicoccaceae	G_Butyricicoccus	unclassified_G_Butyricicoccus
SV1499	K_Bacteria	P_Firmicutes	C_Clostridia	O_Clostridia UCG-014	unclassified_O_Clostridia UCG-014	unclassified_O_Clostridia UCG-014	unclassified_O_Clostridia UCG-014
SV1500	K_Bacteria	P_Firmicutes	C_Clostridia	O_Oscillospirales	F_Oscillospiraceae	G_Oscillibacter	unclassified_G_Oscillibacter
SV1501	K_Bacteria	P_Firmicutes	C_Clostridia	O_Lachnospirales	F_Lachnospiraceae	G_Marvinbryantia	unclassified_G_Marvinbryantia
SV1502	K_Bacteria	P_Firmicutes	C_Clostridia	O_Lachnospirales	F_Lachnospiraceae	unclassified_F_Lachnospiraceae	unclassified_F_Lachnospiraceae
SV1503	K_Bacteria	P_Firmicutes	C_Clostridia	O_Oscillospirales	F_Ruminococcaceae	unclassified_F_Ruminococcaceae	unclassified_F_Ruminococcaceae
SV1504	K_Bacteria	P_Firmicutes	C_Clostridia	O_Oscillospirales	F_Ruminococcaceae	G_Incertae Sedis	unclassified_G_Incertae Sedis
SV1505	K_Bacteria	P_Firmicutes	C_Clostridia	O_Oscillospirales	F_Ruminococcaceae	unclassified_F_Ruminococcaceae	unclassified_F_Ruminococcaceae
SV1506	K_Bacteria	P_Proteobacteria	C_Gammaproteobacteria	O_Enterobacterales	F_Enterobacteriaceae	G_Enterobacter	unclassified_G_Enterobacter
SV1507	K_Bacteria	P_Actinobacteriota	C_Coriobacteriia	O_Coriobacteriales	F_Eggerthellaceae	G_Enterorhabdus	unclassified_G_Enterorhabdus
SV1508	K_Bacteria	P_Firmicutes	C_Clostridia	O_Oscillospirales	F_Oscillospiraceae	G_Colidextribacter	unclassified_G_Colidextribacter
SV1509	K_Bacteria	P_Firmicutes	C_Clostridia	O_Lachnospirales	F_Lachnospiraceae	G_Lachnospiraceae UCG-006	unclassified_G_Lachnospiraceae UCG-006
SV1510	K_Bacteria	P_Firmicutes	C_Clostridia	O_Oscillospirales	F_[Eubacterium] coprostanoligenes group	unclassified_F_[Eubacterium] coprostanoligenes grou	unclassified_F_[Eubacterium] coprostanoligenes group
SV1511	K_Bacteria	P_Firmicutes	C_Clostridia	O_Lachnospirales	F_Lachnospiraceae	G_Lachnospiraceae UCG-001	unclassified_G_Lachnospiraceae UCG-001
SV1512	K_Bacteria	P_Firmicutes	C_Clostridia	O_Oscillospirales	F_Oscillospiraceae	G_Colidextribacter	unclassified_G_Colidextribacter
SV1513	K_Bacteria	P_Firmicutes	C_Clostridia	O_Clostridia UCG-014	unclassified_O_Clostridia UCG-014	unclassified_O_Clostridia UCG-014	unclassified_O_Clostridia UCG-014
SV1514	K_Bacteria	P_Firmicutes	C_Clostridia	O_Lachnospirales	F_Lachnospiraceae	G_Lachnospiraceae NK4A136 group	unclassified_G_Lachnospiraceae NK4A136 group
SV1515	K_Bacteria	P_Firmicutes	C_Clostridia	O_Lachnospirales	F_Lachnospiraceae	G_Lachnospiraceae NK4A136 group	unclassified_G_Lachnospiraceae NK4A136 group
SV1516	K_Bacteria	P_Firmicutes	C_Clostridia	O_Lachnospirales	F_Lachnospiraceae	unclassified_F_Lachnospiraceae	unclassified_F_Lachnospiraceae

SV1517	K Bacteria	P Firmicutes	C Clostridia	O Oscillospirales	F Ruminococcaceae	unclassified F Ruminococcaceae	unclassified F Ruminococcaceae	
SV1518	K Bacteria	P Bacteroidota	C Bacteroidia	O Bacteroidales	F Muribaculaceae	unclassified F Muribaculaceae	unclassified F Muribaculaceae	
SV1519	K Bacteria	P Firmicutes	C Clostridia	O Clostridia UCG-014	unclassified O Clostridia UCG-014	unclassified O Clostridia UCG-014	unclassified O Clostridia UCG-014	
SV1520	K Bacteria	P Firmicutes	C Clostridia	O Peptostreptococcales-Tissierellales	F Peptostreptococcaceae	G Romboutsia	unclassified G Romboutsia	
SV1521	K Bacteria	P Firmicutes	C Clostridia	O Oscillospirales	F [Eubacterium] coprostanoligenes group	unclassified F [Eubacterium] coprostanoligenes group	unclassified F [Eubacterium] coprostanoligenes group	
SV1522	K Bacteria	P Firmicutes	C Clostridia	O Lachnospirales	F Lachnospiraceae	G Lachnospiraceae NK4A136 group	unclassified G Lachnospiraceae NK4A136 group	
SV1523	K Bacteria	P Firmicutes	C Clostridia	O Lachnospirales	F Lachnospiraceae	unclassified F Lachnospiraceae	unclassified F Lachnospiraceae	
SV1524	K Bacteria	P Firmicutes	C Clostridia	O Oscillospirales	F Oscillospiraceae	G Colidextribacter	unclassified G Colidextribacter	
SV1525	K Bacteria	P Firmicutes	C Clostridia	O Oscillospirales	F Ruminococcaceae	G Incertae Sedis	unclassified G Incertae Sedis	
SV1526	K Bacteria	P Firmicutes	C Clostridia	O Lachnospirales	F Lachnospiraceae	unclassified F Lachnospiraceae	unclassified F Lachnospiraceae	
SV1527	K Bacteria	P Firmicutes	C Clostridia	O Clostridia UCG-014	unclassified O Clostridia UCG-014	unclassified O Clostridia UCG-014	unclassified O Clostridia UCG-014	
SV1528	K Bacteria	P Firmicutes	C Clostridia	O Lachnospirales	F Lachnospiraceae	G A2	unclassified G A2	
SV1529	K Bacteria	P Firmicutes	C Clostridia	O Oscillospirales	F [Eubacterium] coprostanoligenes group	unclassified F [Eubacterium] coprostanoligenes group	unclassified F [Eubacterium] coprostanoligenes group	
SV1530	K Bacteria	P Firmicutes	C Clostridia	O Clostridia UCG-014	unclassified O Clostridia UCG-014	unclassified O Clostridia UCG-014	unclassified O Clostridia UCG-014	
SV1531	K Bacteria	P Firmicutes	C Clostridia	O Lachnospirales	F Lachnospiraceae	G Lachnospiraceae NK4A136 group	unclassified G Lachnospiraceae NK4A136 group	
SV1532	K Bacteria	P Bacteroidota	C Bacteroidia	O Bacteroidales	F Muribaculaceae	unclassified F Muribaculaceae	unclassified F Muribaculaceae	
SV1533	K Bacteria	P Firmicutes	C Clostridia	O Oscillospirales	F Oscillospiraceae	G Colidextribacter	unclassified G Colidextribacter	
SV1534	K Bacteria	P Firmicutes	C Clostridia	O Oscillospirales	F Oscillospiraceae	G UCG-005	unclassified G UCG-005	
SV1535	K Bacteria	P Firmicutes	C Clostridia	O Oscillospirales	F Oscillospiraceae	G UCG-005	unclassified G UCG-005	
SV1536	K Bacteria	P Bacteroidota	C Bacteroidia	O Bacteroidales	F Muribaculaceae	unclassified F Muribaculaceae	unclassified F Muribaculaceae	
SV1537	K Bacteria	P Firmicutes	C Clostridia	O Clostridia UCG-014	unclassified O Clostridia UCG-014	unclassified O Clostridia UCG-014	unclassified O Clostridia UCG-014	
SV1538	K Bacteria	P Firmicutes	C Clostridia	O Lachnospirales	F Lachnospiraceae	unclassified F Lachnospiraceae	unclassified F Lachnospiraceae	
SV1539	K Bacteria	P Firmicutes	C Clostridia	O Lachnospirales	F Lachnospiraceae	G Lachnospiraceae NK4A136 group	unclassified G Lachnospiraceae NK4A136 group	
SV1540	K Bacteria	P Firmicutes	C Clostridia	O Clostridia UCG-014	unclassified O Clostridia UCG-014	unclassified O Clostridia UCG-014	unclassified O Clostridia UCG-014	
SV1541	K Bacteria	P Firmicutes	C Clostridia	O Oscillospirales	F Oscillospiraceae	unclassified F Oscillospiraceae	unclassified F Oscillospiraceae	
SV1542	K Bacteria	P Firmicutes	C Clostridia	O Lachnospirales	F Lachnospiraceae	unclassified F Lachnospiraceae	unclassified F Lachnospiraceae	
SV1543	K Bacteria	P Firmicutes	C Clostridia	O Clostridia UCG-014	unclassified O Clostridia UCG-014	unclassified O Clostridia UCG-014	unclassified O Clostridia UCG-014	
SV1544	K Bacteria	P Firmicutes	C Clostridia	O Lachnospirales	F Lachnospiraceae	G Blautia	unclassified G Blautia	
SV1545	K Bacteria	P Firmicutes	C Clostridia	O Clostridia UCG-014	unclassified O Clostridia UCG-014	unclassified O Clostridia UCG-014	unclassified O Clostridia UCG-014	
SV1546	K Bacteria	P Firmicutes	C Clostridia	O Lachnospirales	F Lachnospiraceae	G Roseburia	unclassified G Roseburia	
SV1547	K Bacteria	P Firmicutes	C Clostridia	O Lachnospirales	F Lachnospiraceae	G Blautia	unclassified G Blautia	
SV1548	K Bacteria	P Firmicutes	C Clostridia	O Clostridiales	F Clostridiaceae	G Clostridium sensu stricto 1	unclassified G Clostridium sensu stricto 1	
SV1549	K Bacteria	P Firmicutes	C Clostridia	O Lachnospirales	F Lachnospiraceae	G [Ruminococcus] gnavus group	unclassified G [Ruminococcus] gnavus group	
SV1550	K Bacteria	P Firmicutes	C Clostridia	O Lachnospirales	F Lachnospiraceae	G Lachnospiraceae UCG-006	unclassified G Lachnospiraceae UCG-006	
SV1551	K Bacteria	P Firmicutes	C Clostridia	O Oscillospirales	F Ruminococcaceae	G Anaerotruncus	S colihominis	
SV1552	K Bacteria	P Firmicutes	C Clostridia	O Oscillospirales	F Ruminococcaceae	unclassified F Ruminococcaceae	unclassified F Ruminococcaceae	
SV1553	K Bacteria	P Firmicutes	C Clostridia	O Oscillospirales	F Ruminococcaceae	G Incertae Sedis	unclassified G Incertae Sedis	
SV1554	K Bacteria	P Firmicutes	C Clostridia	O Lachnospirales	F Lachnospiraceae	G Lachnospiraceae NK4A136 group	unclassified G Lachnospiraceae NK4A136 group	
SV1555	K Bacteria	P Firmicutes	C Clostridia	O Clostridiales	F Clostridiaceae	G Clostridium sensu stricto 1	unclassified G Clostridium sensu stricto 1	
SV1556	K Bacteria	P Firmicutes	C Clostridia	O Lachnospirales	F Lachnospiraceae	unclassified F Lachnospiraceae	unclassified F Lachnospiraceae	
SV1557	K Bacteria	P Firmicutes	C Clostridia	O Clostridia UCG-014	unclassified O Clostridia UCG-014	unclassified O Clostridia UCG-014	unclassified O Clostridia UCG-014	
SV1558	K Bacteria	P Firmicutes	C Clostridia	O Lachnospirales	F Lachnospiraceae	G Lachnospiraceae NK4A136 group	unclassified G Lachnospiraceae NK4A136 group	
SV1559	K Bacteria	P Firmicutes	C Clostridia	O Lachnospirales	F Lachnospiraceae	G Roseburia	unclassified G Roseburia	
SV1560	K Bacteria	P Firmicutes	C Clostridia	O Clostridiales	F Clostridiaceae	G Clostridium sensu stricto 1	unclassified G Clostridium sensu stricto 1	
SV1561	K Bacteria	P Firmicutes	C Clostridia	O Oscillospirales	F Oscillospiraceae	G NK4A214 group	unclassified G NK4A214 group	
SV1562	K Bacteria	P Firmicutes	C Clostridia	O Monoglobales	F Monoglobaceae	G Monoglobus	unclassified G Monoglobus	
SV1563	K_Bacteria	P_Firmicutes	C_Clostridia	O_Lachnospirales	F_Lachnospiraceae	unclassified_F_Lachnospiraceae	unclassified_F_Lachnospiraceae	
SV1564	K Bacteria	P Firmicutes	C Clostridia	O Lachnospirales	F Lachnospiraceae	G Lachnospiraceae UCG-001	unclassified G Lachnospiraceae UCG-001	
SV1565	K Bacteria	P Cyanobacteria	C Vampirivibrionia	O Gastranaerophilales	unclassified O Gastranaerophilales	unclassified O Gastranaerophilales	unclassified O Gastranaerophilales	
SV1566	K Bacteria	P Firmicutes	C Clostridia	O Lachnospirales	F Lachnospiraceae	G Lachnoclostridium	unclassified G Lachnoclostridium	
SV1567	K Bacteria	P Firmicutes	C Clostridia	O Lachnospirales	F Lachnospiraceae	G Lachnoclostridium	unclassified G Lachnoclostridium	
SV1568	K Bacteria	P Firmicutes	C Clostridia	O Lachnospirales	F Lachnospiraceae	unclassified F Lachnospiraceae	unclassified F Lachnospiraceae	
SV1569	K_Bacteria	P_Firmicutes	C_Clostridia	O_Oscillospirales	F_Oscillospiraceae	G_Colidextribacter	unclassified_G_Colidextribacter	
SV1570	K Bacteria	P Firmicutes	C Clostridia	O Lachnospirales	F Lachnospiraceae	G Roseburia	unclassified G Roseburia	
SV1571	K Bacteria	P Firmicutes	C Clostridia	O Oscillospirales	F Oscillospiraceae	G Intestinimonas	unclassified G Intestinimonas	
SV1572	K Bacteria	P Firmicutes	C Bacilli	O Lactobacillales	F Lactobacillaceae	unclassified F Lactobacillaceae	unclassified F Lactobacillaceae	
SV1573	K Bacteria	P Firmicutes	C Clostridia	O Lachnospirales	F Lachnospiraceae	unclassified F Lachnospiraceae	unclassified F Lachnospiraceae	
SV1574	K Bacteria	P Firmicutes	C Clostridia	O Lachnospirales	F Lachnospiraceae	G GCA-900066575	unclassified G GCA-900066575	
SV1575	K_Bacteria	P_Firmicutes	C_Clostridia	O_Oscillospirales	F_Oscillospiraceae	G_Colidextribacter	unclassified_G_Colidextribacter	
SV1576	K Bacteria	P Firmicutes	C Clostridia	O Lachnospirales	F Lachnospiraceae	G Eisenbergiella	S massiliensis	
SV1577	K Bacteria	P Bacteroidota	C Bacteroidia	O Bacteroidales	F Bacteroidaceae	G Bacteroides	unclassified G Bacteroides	
SV1578	K Bacteria	P Firmicutes	C Clostridia	O Lachnospirales	F Lachnospiraceae	G Lachnoclostridium	unclassified G Lachnoclostridium	
SV1579	K Bacteria	P Firmicutes	C Clostridia	O Peptostreptococcales-Tissierellales	F Anaerovoracaceae	G [Eubacterium] nodatum group	unclassified G [Eubacterium] nodatum group	
SV1580	K Bacteria	P Firmicutes	C Clostridia	O Lachnospirales	F Lachnospiraceae	G Lachnoclostridium	unclassified G Lachnoclostridium	
SV1581	K_Bacteria	P_Firmicutes	C_Clostridia	O_Lachnospirales	F_Lachnospiraceae	unclassified_F_Lachnospiraceae	unclassified_F_Lachnospiraceae	
SV1582	K Bacteria	P Bacteroidota	C Bacteroidia	O Bacteroidales	F Muribaculaceae	unclassified F Muribaculaceae	unclassified F Muribaculaceae	
SV1583	K Bacteria	P Bacteroidota	C Bacteroidia	O Bacteroidales	F Muribaculaceae	unclassified F Muribaculaceae	unclassified F Muribaculaceae	
SV1584	K Bacteria	P Firmicutes	C Clostridia	O Lachnospirales	F Lachnospiraceae	G Lachnospiraceae NK4A136 group	unclassified G Lachnospiraceae NK4A136 group	
SV1585	K Bacteria	P Firmicutes	C Clostridia	O Oscillospirales	F Ruminococcaceae	unclassified F Ruminococcaceae	unclassified F Ruminococcaceae	
SV1586	K Bacteria	P Firmicutes	C Clostridia	O Peptostreptococcales-Tissierellales	F Peptostreptococcaceae	unclassified F Peptostreptococcaceae	unclassified F Peptostreptococcaceae	
SV1587	K_Bacteria	P_Firmicutes	C_Clostridia	O_Lachnospirales	F_Lachnospiraceae	G_Acetatifactor	unclassified_G_Acetatifactor	
SV1588	K Bacteria	P Firmicutes	C Clostridia	O Lachnospirales	F Lachnospiraceae	unclassified F Lachnospiraceae	unclassified F Lachnospiraceae	
SV1589	K Bacteria	P Firmicutes	C Clostridia	O Lachnospirales	F Lachnospiraceae	unclassified F Lachnospiraceae	unclassified F Lachnospiraceae	
SV1590	K Bacteria	P Firmicutes	C Clostridia	O Oscillospirales	F Butyricicoccaceae	G Butyricicoccus	unclassified G Butyricicoccus	
SV1591	K Bacteria	P Bacteroidota	C Bacteroidia	O Bacteroidales	F Prevotellaceae	G Prevotellaceae UCG-001	unclassified G Prevotellaceae UCG-001	
SV1592	K Bacteria	P Firmicutes	C Clostridia	O Clostridia UCG-014	unclassified O Clostridia UCG-014	unclassified O Clostridia UCG-014	unclassified O Clostridia UCG-014	
SV1593	K Bacteria	P Firmicutes	C Clostridia	O Clostridia UCG-014	unclassified O Clostridia UCG-014	unclassified O Clostridia UCG-014	unclassified O Clostridia UCG-014	
SV1594	K Bacteria	P Firmicutes	C Clostridia	O Lachnospirales	F Lachnospiraceae	G Blautia	unclassified G Blautia	
SV1595	K Bacteria	P Bacteroidota	C Bacteroidia	O Bacteroidales	F Muribaculaceae	unclassified F Muribaculaceae	unclassified F Muribaculaceae	
SV1596	K Bacteria	P Firmicutes	C Clostridia	O Lachnospirales	F Lachnospiraceae	G ASF356	unclassified G ASF356	
SV1597	K Bacteria	P Firmicutes	C Clostridia	O Lachnospirales	F Lachnospiraceae	G GCA-900066575	unclassified G GCA-900066575	
SV1598	K Bacteria	P Firmicutes	C Clostridia	O Lachnospirales	F Lachnospiraceae	G Roseburia	unclassified G Roseburia	
SV1599	K Bacteria	P Firmicutes	C Clostridia	O Lachnospirales	F Lachnospiraceae	G Tuzzerella	unclassified G Tuzzerella	
SV1600	K Bacteria	P Firmicutes	C Clostridia	O Lachnospirales	F Lachnospiraceae	unclassified F Lachnospiraceae	unclassified F Lachnospiraceae	
SV1601	K Bacteria	P Firmicutes	C Clostridia	O Oscillospirales	F Ruminococcaceae	unclassified F Ruminococcaceae	unclassified F Ruminococcaceae	
SV1602	K Bacteria	P Firmicutes	C Clostridia	O Lachnospirales	F Lachnospiraceae	unclassified F Lachnospiraceae	unclassified F Lachnospiraceae	
SV1603	K Bacteria	P Firmicutes	C Clostridia	O Lachnospirales	F Lachnospiraceae	unclassified F Lachnospiraceae	unclassified F Lachnospiraceae	
SV1604	K Bacteria	P Firmicutes	C Bacilli	O RF39	unclassified O RF39	unclassified O RF39	unclassified O RF39	
SV1605	K Bacteria	P Firmicutes	C Clostridia	O Lachnospirales	F Lachnospiraceae	unclassified F Lachnospiraceae	unclassified F Lachnospiraceae	
SV1606	K Bacteria	P Firmicutes	C Clostridia	O Lachnospirales	F Lachnospiraceae	G Lachnoclostridium	unclassified G Lachnoclostridium	
SV1607	K Bacteria	P Firmicutes	C Clostridia	O Lachnospirales	F Lachnospiraceae	unclassified F Lachnospiraceae	unclassified F Lachnospiraceae	
SV1608	K Bacteria	P Firmicutes	C Clostridia	O Lachnospirales	F Lachnospiraceae	G Lachnospiraceae FCS020 group	unclassified G Lachnospiraceae FCS020 group	
SV1609	K Bacteria	P Firmicutes	C Clostridia	O Lachnospirales	F Lachnospiraceae	G [Eubacterium] fissicatena group	unclassified G [Eubacterium] fissicatena group	
SV1610	K Bacteria	P Firmicutes	C Clostridia	O Oscillospirales	F Oscillospiraceae	G Intestinimonas	unclassified G Intestinimonas	
SV1611	K Bacteria	P Firmicutes	C Clostridia	O Lachnospirales	F Lachnospiraceae	G Marvinbryantia	unclassified G Marvinbryantia	
SV1612	K Bacteria	P Firmicutes	C Clostridia	O Lachnospirales	F Lachnospiraceae	G Lachnospiraceae UCG-001	unclassified G Lachnospiraceae UCG-001	
SV1613	K Bacteria	P Firmicutes	C Clostridia	O Clostridia UCG-014	unclassified O Clostridia UCG-014	unclassified O Clostridia UCG-014	unclassified O Clostridia UCG-014	
SV1614	K Bacteria	P Firmicutes	C Clostridia	O Oscillospirales	F Butyricicoccaceae	G Butyricicoccus	unclassified G Butyricicoccus	
SV1615	K Bacteria	P Bacteroidota	C Bacteroidia	O Bacteroidales	F Prevotellaceae	G Prevotellaceae UCG-001	unclassified G Prevotellaceae UCG-001	
SV1616	K Bacteria	P Bacteroidota	C Bacteroidia	O Bacteroidales	F Muribaculaceae	unclassified F Muribaculaceae	unclassified F Muribaculaceae	
SV1617	K Bacteria	P Firmicutes	C Bacilli	O Erysipelotrichales	F Erysipelotrichaceae	G Turicibacter	unclassified G Turicibacter	
SV1618	K Bacteria	P Firmicutes	C Clostridia	O Oscillospirales	F Ruminococcaceae	unclassified F Ruminococcaceae	unclassified F Ruminococcaceae	
SV1619	K Bacteria	P Bacteroidota	C Bacteroidia	O Bacteroidales	F Prevotellaceae	G Prevotellaceae UCG-001	unclassified G Prevotellaceae UCG-001	
SV1620	K Bacteria	P Firmicutes	C Clostridia	O Clostridiales	F Clostridiaceae	G Clostridium sensu stricto 1	unclassified G Clostridium sensu stricto 1	
SV1621	K Bacteria	P Firmicutes	C Clostridia	O Lachnospirales	F Lachnospiraceae	unclassified F Lachnospiraceae	unclassified F Lachnospiraceae	
SV1622	K Bacteria	P Firmicutes	C Clostridia	O Oscillospirales	F Oscillospiraceae	G Colidextribacter	unclassified G Colidextribacter	
SV1623	K Bacteria	P Firmicutes	C Clostridia	O Lachnospirales	F Lachnospiraceae	G Lachnospiraceae NK4A136 group	unclassified G Lachnospiraceae NK4A136 group	
SV1624	K Bacteria	P Firmicutes	C Clostridia	O Lachnospirales	F Lachnospiraceae	unclassified F Lachnospiraceae	unclassified F Lachnospiraceae	
SV1625	K Bacteria	P Firmicutes	C Clostridia	O Oscillospirales	F UCG-010	unclassified F UCG-010	unclassified F UCG-010	
SV1626	K Bacteria	P Firmicutes	C Clostridia	O Lachnospirales	F Lachnospiraceae	unclassified F Lachnospiraceae	unclassified F Lachnospiraceae	
SV1627	K Bacteria	P Firmicutes	C Clostridia	O Christensenellales	F Christensenellaceae	G Christensenellaceae R-7 group	unclassified G Christensenellaceae R-7 group	
SV1628	K_Bacteria	P_Firmicutes	C_Clostridia	O_Lachnospirales	F_Lachnospiraceae	unclassified_F_Lachnospiraceae	unclassified_F_Lachnospiraceae	
SV1629	K Bacteria	P Firmicutes	C Clostridia	O Clostridia UCG-014	unclassified O Clostridia UCG-014	unclassified O Clostridia UCG-014	unclassified O Clostridia UCG-014	
SV1630	K Bacteria	P Firmicutes	C Clostridia	O Lachnospirales	F Lachnospiraceae	unclassified F Lachnospiraceae	unclassified F Lachnospiraceae	
SV1631	K Bacteria	P Bacteroidota	C Bacteroidia	O Bacteroidales	F Marinifilaceae	G Odoribacter	unclassified G Odoribacter	
SV1632	K Bacteria	P Firmicutes	C Clostridia	O Lachnospirales	F Lachnospiraceae	G Lachnospiraceae NK4A136 group	unclassified G Lachnospiraceae NK4A136 group	
SV1633	K Bacteria	P Bacteroidota	C Bacteroidia	O Bacteroidales	F Muribaculaceae	unclassified F Muribaculaceae	unclassified F Muribaculaceae	
SV1634	K_Bacteria	P_Firmicutes	C_Clostridia	O_Lachnospirales	F_Lachnospiraceae	G_Lachnospiraceae NK4A136 group	unclassified_G_Lachnospiraceae NK4A136 group	
SV1635	K Bacteria	P Firmicutes	C Clostridia	O Lachnospirales	F Lachnospiraceae	G Lachnospiraceae FCS020 group	unclassified G Lachnospiraceae FCS020 group	
SV1636	K Bacteria	P Firmicutes	C Clostridia	O Oscillospirales	F Oscillospiraceae	G Intestinimonas	unclassified G Intestinimonas	
SV1637	K Bacteria	P Firmicutes	C Clostridia	O Lachnospirales	F Lachnospiraceae	G Roseburia	unclassified G Roseburia	
SV1638	K Bacteria	P Firmicutes	C Clostridia	O Lachnospirales	F Lachnospiraceae	G Lachnoclostridium	unclassified G Lachnoclostridium	
SV1639	K Bacteria	P Firmicutes	C Clostridia	O Oscillospirales	F Ruminococcaceae	G Paludicola	unclassified G Paludicola	
SV1640	K_Bacteria	P_Firmicutes	C_Clostridia	O_Lachnospirales	F_Lachnospiraceae	unclassified_F_Lachnospiraceae	unclassified_F_Lachnospiraceae	
SV1641	K Bacteria	P Firmicutes	C Clostridia	O Clostridia vadinBB60 group	unclassified O Clostridia vadinBB60 group	unclassified O Clostridia vadinBB60 group	unclassified O Clostridia vadinBB60 group	
SV1642	K Bacteria	P Firmicutes	C Bacilli	O RF39	unclassified O RF39	unclassified O RF39	unclassified O RF39	
SV1643	K Bacteria	P Firmicutes	C Clostridia	O Oscillospirales	F Ruminococcaceae	G Anaerotruncus	unclassified G Anaerotruncus	
SV1644	K Bacteria	P Firmicutes	C Clostridia	O Peptostreptococcales-Tissierellales	F Peptostreptococcaceae	G Romboutsia	unclassified G Romboutsia	
SV1645	K Bacteria	P Firmicutes	C Bacilli	O Lactobacillales	F Lactobacillaceae	G Ligilactobacillus	unclassified G Ligilactobacillus	
SV1646	K_Bacteria	P_Firmicutes	C_Clostridia	O_Oscillospirales	F_Ruminococcaceae	unclassified_F_Ruminococcaceae	unclassified_F_Ruminococcaceae	
SV1647	K Bacteria	P Firmicutes	C Clostridia	O Oscillospirales	F UCG-010	unclassified F UCG-010	unclassified F UCG-010	
SV1648	K Bacteria	P Firmicutes	C Clostridia	O Lachnospirales	F Lachnospiraceae	unclassified F Lachnospiraceae	unclassified F Lachnospiraceae	
SV1649	K Bacteria	P Firmicutes	C Clostridia	O Lachnospirales	F Lachnospiraceae	G Lachnoclostridium	unclassified G Lachnoclostridium	
SV1650	K Bacteria	P Firmicutes	C Clostridia	O Oscillospirales	F Oscillospiraceae	G Oscillibacter	unclassified G Oscillibacter	
SV1651	K Bacteria	P Firmicutes	C Clostridia	O Oscillospirales	F Oscillospiraceae	unclassified F Oscillospiraceae	unclassified F Oscillospiraceae	
SV1652	K Bacteria	P Firmicutes	C Clostridia	O Clostridia vadinBB60 group	unclassified O Clostridia vadinBB60 group	unclassified O Clostridia vadinBB60 group	unclassified O Clostridia vadinBB60 group	
SV1653	K Bacteria	P Firmicutes	C Bacilli	O Lactobacillales	F Lactobacillaceae	unclassified F Lactobacillaceae	unclassified F Lactobacillaceae	
SV1654	K Bacteria	P Firmicutes	C Clostridia	O Lachnospirales	F Lachnospiraceae	unclassified F Lachnospiraceae	unclassified F Lachnospiraceae	

SV1655	K Bacteria	P Firmicutes	C Clostridia	O Oscillospirales	F Oscillospiraceae	G Oscillibacter	unclassified G Oscillibacter
SV1656	K Bacteria	P Firmicutes	C Clostridia	O Lachnospirales	F Lachnospiraceae	unclassified F Lachnospiraceae	unclassified F Lachnospiraceae
SV1657	K Bacteria	P Firmicutes	C Clostridia	O Clostridia UCG-014	unclassified O Clostridia UCG-014	unclassified O Clostridia UCG-014	unclassified O Clostridia UCG-014
SV1658	K Bacteria	P Firmicutes	C Clostridia	O Peptostreptococcales-Tissierellales	F Anaerovoracaceae	G [Eubacterium] brachy group	unclassified G [Eubacterium] brachy group
SV1659	K Bacteria	P Firmicutes	C Clostridia	O Oscillospirales	F Ruminococcaceae	G UBA1819	unclassified G UBA1819
SV1660	K Bacteria	P Bacteroidota	C Bacteroidia	O Bacteroidales	F Muribaculaceae	unclassified F Muribaculaceae	unclassified F Muribaculaceae
SV1661	K Bacteria	P Bacteroidota	C Bacteroidia	O Bacteroidales	F Muribaculaceae	unclassified F Muribaculaceae	unclassified F Muribaculaceae
SV1662	K Bacteria	P Firmicutes	C Clostridia	O Lachnospirales	F Lachnospiraceae	G GCA-900066575	unclassified G GCA-900066575
SV1663	K Bacteria	P Firmicutes	C Clostridia	O Lachnospirales	F Lachnospiraceae	G Lachnoclostridium	unclassified G Lachnoclostridium
SV1664	K Bacteria	P Firmicutes	C Clostridia	O Lachnospirales	F Lachnospiraceae	G GCA-900066575	unclassified G GCA-900066575
SV1665	K Bacteria	P Firmicutes	C Clostridia	O Lachnospirales	F Lachnospiraceae	G GCA-900066575	unclassified G GCA-900066575
SV1666	K Bacteria	P Firmicutes	C Clostridia	O Lachnospirales	F Lachnospiraceae	G GCA-900066575	unclassified G GCA-900066575
SV1667	K Bacteria	P Firmicutes	C Clostridia	O Lachnospirales	F Lachnospiraceae	G Lachnoclostridium	unclassified G Lachnoclostridium
SV1668	K Bacteria	P Firmicutes	C Clostridia	O Oscillospirales	F Oscillospiraceae	unclassified F Oscillospiraceae	unclassified F Oscillospiraceae
SV1669	K Bacteria	P Firmicutes	C Clostridia	O Lachnospirales	F Lachnospiraceae	G Lachnospiraceae UCG-001	unclassified G Lachnospiraceae UCG-001
SV1670	K Bacteria	P Firmicutes	C Clostridia	O Clostridia UCG-014	unclassified O Clostridia UCG-014	unclassified O Clostridia UCG-014	unclassified O Clostridia UCG-014
SV1671	K Bacteria	P Firmicutes	C Clostridia	O Oscillospirales	F Ruminococcaceae	G Harryflintia	unclassified G Harryflintia
SV1672	K Bacteria	P Firmicutes	C Clostridia	O Lachnospirales	F Lachnospiraceae	G Lachnospiraceae NK4A136 group	unclassified G Lachnospiraceae NK4A136 group
SV1673	K Bacteria	P Firmicutes	C Clostridia	O Peptostreptococcales-Tissierellales	F Anaerovoracaceae	G Family XIII AD3011 group	unclassified G Family XIII AD3011 group
SV1674	K Bacteria	P Firmicutes	C Clostridia	O Clostridia UCG-014	unclassified O Clostridia UCG-014	unclassified O Clostridia UCG-014	unclassified O Clostridia UCG-014
SV1675	K Bacteria	P Firmicutes	C Clostridia	O Lachnospirales	F Lachnospiraceae	G Lachnospiraceae NK4A136 group	unclassified G Lachnospiraceae NK4A136 group
SV1676	K Bacteria	P Firmicutes	C Bacilli	O Staphylococcales	F Staphylococcaceae	unclassified F Staphylococcaceae	unclassified F Staphylococcaceae
SV1677	K Bacteria	P Firmicutes	C Clostridia	O Lachnospirales	F Lachnospiraceae	unclassified F Lachnospiraceae	unclassified F Lachnospiraceae
SV1678	K Bacteria	P Firmicutes	C Clostridia	O Lachnospirales	F Lachnospiraceae	G Lachnospiraceae NK4A136 group	unclassified G Lachnospiraceae NK4A136 group
SV1679	K Bacteria	P Firmicutes	C Clostridia	O Clostridia UCG-014	unclassified O Clostridia UCG-014	unclassified O Clostridia UCG-014	unclassified O Clostridia UCG-014
SV1680	K Bacteria	P Firmicutes	C Clostridia	O Oscillospirales	F Oscillospiraceae	G Colidextribacter	unclassified G Colidextribacter
SV1681	K Bacteria	P Firmicutes	C Clostridia	O Oscillospirales	F Ruminococcaceae	G Candidatus Soleaferrea	unclassified G Candidatus Soleaferrea
SV1682	K Bacteria	P Firmicutes	C Clostridia	O Lachnospirales	F Lachnospiraceae	unclassified F Lachnospiraceae	unclassified F Lachnospiraceae
SV1683	K Bacteria	P Firmicutes	C Clostridia	O Lachnospirales	F Lachnospiraceae	G Lachnospiraceae NK4A136 group	unclassified G Lachnospiraceae NK4A136 group
SV1684	K Bacteria	P Firmicutes	C Bacilli	O Erysipelotrichales	F Erysipelatoclostridiaceae	G Erysipelatoclostridium	unclassified G Erysipelatoclostridium
SV1685	K Bacteria	P Firmicutes	C Clostridia	O Lachnospirales	F Lachnospiraceae	unclassified F Lachnospiraceae	unclassified F Lachnospiraceae
SV1686	K Bacteria	P Firmicutes	C Clostridia	O Lachnospirales	F Lachnospiraceae	unclassified F Lachnospiraceae	unclassified F Lachnospiraceae
SV1687	K Bacteria	P Firmicutes	C Clostridia	O Oscillospirales	F Oscillospiraceae	G Colidextribacter	unclassified G Colidextribacter
SV1688	K Bacteria	P Firmicutes	C Clostridia	O Lachnospirales	F Lachnospiraceae	G Tuzzerella	unclassified G Tuzzerella
SV1689	K Bacteria	P Firmicutes	C Clostridia	O Lachnospirales	F Lachnospiraceae	G Lachnospiraceae UCG-010	unclassified G Lachnospiraceae UCG-010
SV1690	K Bacteria	P Bacteroidota	C Bacteroidia	O Bacteroidales	F Bacteroidaceae	G Bacteroides	unclassified G Bacteroides
SV1691	K Bacteria	P Firmicutes	C Clostridia	O Lachnospirales	F Lachnospiraceae	G Roseburia	unclassified G Roseburia
SV1692	K Bacteria	P Bacteroidota	C Bacteroidia	O Bacteroidales	F Rikenellaceae	G Alistipes	unclassified G Alistipes
SV1693	K Bacteria	P Firmicutes	C Clostridia	O Oscillospirales	F Oscillospiraceae	unclassified F Oscillospiraceae	unclassified F Oscillospiraceae
SV1694	K Bacteria	P Firmicutes	C Clostridia	O Lachnospirales	F Lachnospiraceae	unclassified F Lachnospiraceae	unclassified F Lachnospiraceae
SV1695	K Bacteria	P Firmicutes	C Clostridia	O Lachnospirales	F Lachnospiraceae	G Roseburia	unclassified G Roseburia
SV1696	K Bacteria	P Firmicutes	C Clostridia	O Oscillospirales	F Oscillospiraceae	unclassified F Oscillospiraceae	unclassified F Oscillospiraceae
SV1697	K Bacteria	P Firmicutes	C Bacilli	O Lactobacillales	F Lactobacillaceae	G Ligilactobacillus	unclassified G Ligilactobacillus
SV1698	K Bacteria	P Firmicutes	C Clostridia	O Oscillospirales	F Oscillospiraceae	G UCG-007	unclassified G UCG-007
SV1699	K Bacteria	P Firmicutes	C Clostridia	O Clostridiales	F Clostridiaceae	G Clostridium sensu stricto 1	unclassified G Clostridium sensu stricto 1
SV1700	K Bacteria	P Firmicutes	C Clostridia	O Oscillospirales	F Oscillospiraceae	unclassified F Oscillospiraceae	unclassified F Oscillospiraceae
SV1701	K_Bacteria	P_Firmicutes	C_Clostridia	O_Lachnospirales	F_Lachnospiraceae	unclassified_F_Lachnospiraceae	unclassified_F_Lachnospiraceae
SV1702	K Bacteria	P Bacteroidota	C Bacteroidia	O Bacteroidales	F Muribaculaceae	unclassified F Muribaculaceae	unclassified F Muribaculaceae
SV1703	K Bacteria	P Firmicutes	C Clostridia	O Eubacteriales	F Anaerofustaceae	G Anaerofustis	unclassified G Anaerofustis
SV1704	K Bacteria	P Firmicutes	C Clostridia	O Lachnospirales	F Lachnospiraceae	G [Eubacterium] xylanophilum group	unclassified G [Eubacterium] xylanophilum group
SV1705	K Bacteria	P Firmicutes	C Clostridia	O Clostridia UCG-014	unclassified O Clostridia UCG-014	unclassified O Clostridia UCG-014	unclassified O Clostridia UCG-014
SV1706	K Bacteria	P Firmicutes	C Clostridia	O Lachnospirales	F Lachnospiraceae	G Roseburia	unclassified G Roseburia
SV1707	K_Bacteria	P_Firmicutes	C_Clostridia	O_Lachnospirales	F_Lachnospiraceae	G_Lachnoclostridium	unclassified_G_Lachnoclostridium
SV1708	K Bacteria	P Firmicutes	C Clostridia	O Lachnospirales	F Lachnospiraceae	unclassified F Lachnospiraceae	unclassified F Lachnospiraceae
SV1709	K Bacteria	P Firmicutes	C Clostridia	O Oscillospirales	F Ruminococcaceae	unclassified F Ruminococcaceae	unclassified F Ruminococcaceae
SV1710	K Bacteria	P Verrucomicrobiota	C Verrucomicrobiae	O Verrucomicrobiales	F Akkermansiaceae	G Akkermansia	unclassified G Akkermansia
SV1711	K Bacteria	P Firmicutes	C Clostridia	O Lachnospirales	F Lachnospiraceae	G Lachnoclostridium	unclassified G Lachnoclostridium
SV1712	K Bacteria	P Firmicutes	C Bacilli	O Lactobacillales	F Lactobacillaceae	unclassified F Lactobacillaceae	unclassified F Lactobacillaceae
SV1713	K_Bacteria	P_Firmicutes	C_Clostridia	O_Lachnospirales	F_Lachnospiraceae	unclassified_F_Lachnospiraceae	unclassified_F_Lachnospiraceae
SV1714	K Bacteria	P Firmicutes	C Clostridia	O Lachnospirales	F Lachnospiraceae	G Lachnospiraceae FCS020 group	unclassified G Lachnospiraceae FCS020 group
SV1715	K Bacteria	P Firmicutes	C Bacilli	O Staphylococcales	F Staphylococcaceae	unclassified F Staphylococcaceae	unclassified F Staphylococcaceae
SV1716	K Bacteria	P Firmicutes	C Clostridia	O Oscillospirales	F Oscillospiraceae	G UCG-005	unclassified G UCG-005
SV1717	K Bacteria	P Firmicutes	C Clostridia	O Oscillospirales	F Oscillospiraceae	unclassified F Oscillospiraceae	unclassified F Oscillospiraceae
SV1718	K Bacteria	P Firmicutes	C Clostridia	O Peptostreptococcales-Tissierellales	F Peptostreptococcaceae	unclassified F Peptostreptococcaceae	unclassified F Peptostreptococcaceae
SV1719	K_Bacteria	P_Firmicutes	C_Clostridia	O_Lachnospirales	F_Lachnospiraceae	unclassified_F_Lachnospiraceae	unclassified_F_Lachnospiraceae
SV1720	K Bacteria	P Firmicutes	C Clostridia	O Lachnospirales	F Lachnospiraceae	G GCA-900066575	unclassified G GCA-900066575
SV1721	K Bacteria	P Firmicutes	C Clostridia	O Oscillospirales	F Oscillospiraceae	G Colidextribacter	unclassified G Colidextribacter
SV1722	K Bacteria	P Firmicutes	C Clostridia	O Lachnospirales	F Lachnospiraceae	unclassified F Lachnospiraceae	unclassified F Lachnospiraceae
SV1723	K Bacteria	P Firmicutes	C Clostridia	O Lachnospirales	F Lachnospiraceae	G Lachnoclostridium	unclassified G Lachnoclostridium
SV1724	K Bacteria	P Firmicutes	C Clostridia	O Lachnospirales	F Lachnospiraceae	G Lachnospiraceae UCG-001	unclassified G Lachnospiraceae UCG-001
SV1725	K_Bacteria	P_Firmicutes	C_Bacilli	O_Lactobacillales	F_Lactobacillaceae	G_Ligilactobacillus	unclassified_G_Ligilactobacillus
SV1726	K Bacteria	P Firmicutes	C Clostridia	O Peptostreptococcales-Tissierellales	F Anaerovoracaceae	unclassified F Anaerovoracaceae	unclassified F Anaerovoracaceae
SV1727	K Bacteria	P Firmicutes	C Clostridia	O Lachnospirales	F Lachnospiraceae	unclassified F Lachnospiraceae	unclassified F Lachnospiraceae
SV1728	K Bacteria	P Firmicutes	C Clostridia	O Clostridia vadinBB60 group	unclassified O Clostridia vadinBB60 group	unclassified O Clostridia vadinBB60 group	unclassified O Clostridia vadinBB60 group
SV1729	K Bacteria	P Firmicutes	C Clostridia	O Oscillospirales	F Oscillospiraceae	G Flavonifractor	S plautii
SV1730	K Bacteria	P Proteobacteria	C Alphaproteobacteria	O Rhodospirillales	unclassified O Rhodospirillales	unclassified O Rhodospirillales	unclassified O Rhodospirillales
SV1731	K Bacteria	P Firmicutes	C Clostridia	O Oscillospirales	F Ruminococcaceae	unclassified F Ruminococcaceae	unclassified F Ruminococcaceae
SV1732	K Bacteria	P Firmicutes	C Clostridia	O Lachnospirales	F Lachnospiraceae	G Acetatifactor	unclassified G Acetatifactor
SV1733	K Bacteria	P Firmicutes	C Clostridia	O Lachnospirales	F Lachnospiraceae	G Lachnospiraceae NK4A136 group	unclassified G Lachnospiraceae NK4A136 group
SV1734	K Bacteria	P Bacteroidota	C Bacteroidia	O Bacteroidales	F Muribaculaceae	unclassified F Muribaculaceae	unclassified F Muribaculaceae
SV1735	K Bacteria	P Firmicutes	C Clostridia	O Peptostreptococcales-Tissierellales	F Peptostreptococcaceae	unclassified F Peptostreptococcaceae	unclassified F Peptostreptococcaceae
SV1736	K Bacteria	P Firmicutes	C Bacilli	O Erysipelotrichales	F Erysipelotrichaceae	G Turicibacter	unclassified G Turicibacter
SV1737	K Bacteria	P Firmicutes	C Clostridia	O Lachnospirales	F Lachnospiraceae	G Lachnoclostridium	unclassified G Lachnoclostridium
SV1738	K Bacteria	P Firmicutes	C Clostridia	O Lachnospirales	F Lachnospiraceae	unclassified F Lachnospiraceae	unclassified F Lachnospiraceae
SV1739	K Bacteria	P Firmicutes	C Bacilli	O Erysipelotrichales	F Erysipelotrichaceae	unclassified F Erysipelotrichaceae	unclassified F Erysipelotrichaceae
SV1740	K Bacteria	P Firmicutes	C Clostridia	O Lachnospirales	F Lachnospiraceae	unclassified F Lachnospiraceae	unclassified F Lachnospiraceae
SV1741	K Bacteria	P Firmicutes	C Clostridia	O Oscillospirales	F Ruminococcaceae	G Ruminococcus	unclassified G Ruminococcus
SV1742	K Bacteria	P Firmicutes	C Clostridia	O Oscillospirales	F Ruminococcaceae	unclassified F Ruminococcaceae	unclassified F Ruminococcaceae
SV1743	K Bacteria	P Firmicutes	C Clostridia	O Lachnospirales	F Lachnospiraceae	unclassified F Lachnospiraceae	unclassified F Lachnospiraceae
SV1744	K Bacteria	P Firmicutes	C Clostridia	O Lachnospirales	F Lachnospiraceae	G Lachnospiraceae NK4A136 group	unclassified G Lachnospiraceae NK4A136 group
SV1745	K Bacteria	P Firmicutes	C Clostridia	O Lachnospirales	F Lachnospiraceae	unclassified F Lachnospiraceae	unclassified F Lachnospiraceae
SV1746	K Bacteria	P Firmicutes	C Clostridia	O Lachnospirales	F Lachnospiraceae	G Lachnoclostridium	unclassified G Lachnoclostridium
SV1747	K Bacteria	P Firmicutes	C Clostridia	O Oscillospirales	F Oscillospiraceae	G Colidextribacter	unclassified G Colidextribacter
SV1748	K Bacteria	P Firmicutes	C Clostridia	O Clostridia UCG-014	unclassified O Clostridia UCG-014	unclassified O Clostridia UCG-014	unclassified O Clostridia UCG-014
SV1749	K Bacteria	P Firmicutes	C Clostridia	O Lachnospirales	F Lachnospiraceae	G Lachnospiraceae UCG-006	unclassified G Lachnospiraceae UCG-006
SV1750	K Bacteria	P Firmicutes	C Clostridia	O Oscillospirales	F Oscillospiraceae	G Intestinimonas	unclassified G Intestinimonas
SV1751	K Bacteria	P Firmicutes	C Clostridia	O Lachnospirales	F Lachnospiraceae	G A2	unclassified G A2
SV1752	K Bacteria	P Firmicutes	C Clostridia	O Oscillospirales	F Oscillospiraceae	unclassified F Oscillospiraceae	unclassified F Oscillospiraceae
SV1753	K Bacteria	P Firmicutes	C Clostridia	O Lachnospirales	F Lachnospiraceae	G Lachnospiraceae NK4A136 group	unclassified G Lachnospiraceae NK4A136 group
SV1754	K Bacteria	P Firmicutes	C Clostridia	O Oscillospirales	F Oscillospiraceae	G Oscillibacter	unclassified G Oscillibacter
SV1755	K Bacteria	P Firmicutes	C Clostridia	O Oscillospirales	F Oscillospiraceae	G Colidextribacter	unclassified G Colidextribacter
SV1756	K Bacteria	P Bacteroidota	C Bacteroidia	O Bacteroidales	F Rikenellaceae	G Alistipes	unclassified G Alistipes
SV1757	K Bacteria	P Bacteroidota	C Bacteroidia	O Bacteroidales	F Muribaculaceae	G Muribaculum	unclassified G Muribaculum
SV1758	K Bacteria	P Firmicutes	C Clostridia	O Oscillospirales	F Ruminococcaceae	G UBA1819	unclassified G UBA1819
SV1759	K Bacteria	P Firmicutes	C Bacilli	O RF39	unclassified O RF39	unclassified O RF39	unclassified O RF39
SV1760	K Bacteria	P Bacteroidota	C Bacteroidia	O Bacteroidales	F Muribaculaceae	unclassified F Muribaculaceae	unclassified F Muribaculaceae
SV1761	K Bacteria	P Firmicutes	C Clostridia	O Lachnospirales	F Lachnospiraceae	unclassified F Lachnospiraceae	unclassified F Lachnospiraceae
SV1762	K Bacteria	P Firmicutes	C Clostridia	O Clostridiales	F Clostridiaceae	G Clostridium sensu stricto 1	unclassified G Clostridium sensu stricto 1
SV1763	K Bacteria	P Bacteroidota	C Bacteroidia	O Bacteroidales	F Prevotellaceae	G Prevotellaceae UCG-001	unclassified G Prevotellaceae UCG-001
SV1764	K Bacteria	P Firmicutes	C Clostridia	O Lachnospirales	F Lachnospiraceae	G Lachnospiraceae FCS020 group	unclassified G Lachnospiraceae FCS020 group
SV1765	K Bacteria	P Firmicutes	C Clostridia	O Clostridia UCG-014	unclassified O Clostridia UCG-014	unclassified O Clostridia UCG-014	unclassified O Clostridia UCG-014
SV1766	K_Bacteria	P_Firmicutes	C_Clostridia	O_Lachnospirales	F_Lachnospiraceae	G_Lachnoclostridium	unclassified_G_Lachnoclostridium
SV1767	K Bacteria	P Bacteroidota	C Bacteroidia	O Bacteroidales	F Muribaculaceae	unclassified F Muribaculaceae	unclassified F Muribaculaceae
SV1768	K Bacteria	P Firmicutes	C Clostridia	O Clostridia UCG-014	unclassified O Clostridia UCG-014	unclassified O Clostridia UCG-014	unclassified O Clostridia UCG-014
SV1769	K Bacteria	P Firmicutes	C Clostridia	O Oscillospirales	F Oscillospiraceae	unclassified F Oscillospiraceae	unclassified F Oscillospiraceae
SV1770	K Bacteria	P Firmicutes	C Clostridia	O Lachnospirales	F Lachnospiraceae	G GCA-900066575	unclassified G GCA-900066575
SV1771	K Bacteria	P Bacteroidota	C Bacteroidia	O Bacteroidales	F Rikenellaceae	G Alistipes	unclassified G Alistipes
SV1772	K_Bacteria	P_Firmicutes	C_Clostridia	O_Lachnospirales	F_Lachnospiraceae	G_Lachnospiraceae NK4A136 group	unclassified_G_Lachnospiraceae NK4A136 group
SV1773	K Bacteria	P Firmicutes	C Clostridia	O Lachnospirales	F Lachnospiraceae	G Lachnospiraceae NK4A136 group	unclassified G Lachnospiraceae NK4A136 group
SV1774	K Bacteria	P Firmicutes	C Clostridia	O Lachnospirales	F Lachnospiraceae	G GCA-900066575	unclassified G GCA-900066575
SV1775	K Bacteria	P Firmicutes	C Bacilli	O Lactobacillales	F Lactobacillaceae	G HT002	unclassified G HT002
SV1776	K Bacteria	P Firmicutes	C Clostridia	O Lachnospirales	F Lachnospiraceae	G GCA-900066575	unclassified G GCA-900066575
SV1777	K Bacteria	P Firmicutes	C Clostridia	O Lachnospirales	F Lachnospiraceae	unclassified F Lachnospiraceae	unclassified F Lachnospiraceae
SV1778	K_Bacteria	P_Firmicutes	C_Clostridia	O_Oscillospirales	F_Oscillospiraceae	G_Oscillibacter	unclassified_G_Oscillibacter
SV1779	K Bacteria	P Firmicutes	C Clostridia	O Oscillospirales	F Oscillospiraceae	unclassified F Oscillospiraceae	unclassified F Oscillospiraceae
SV1780	K Bacteria	P Firmicutes	C Bacilli	O Erysipelotrichales	F Erysipelotrichaceae	unclassified F Erysipelotrichaceae	unclassified F Erysipelotrichaceae
SV1781	K Bacteria	P Firmicutes	C Clostridia	O Peptococcales	F Peptococcaceae	unclassified F Peptococcaceae	unclassified F Peptococcaceae
SV1782	K Bacteria	P Firmicutes	C Clostridia	O Peptostreptococcales-Tissierellales	F Anaerovoracaceae	G Family XIII AD3011 group	unclassified G Family XIII AD3011 group
SV1783	K Bacteria	P Firmicutes	C Clostridia	O Oscillospirales	F Oscillospiraceae	G Oscillibacter	unclassified G Oscillibacter
SV1784	K_Bacteria	P_Firmicutes	C_Clostridia	O_Oscillospirales	F_Ruminococcaceae	G_Incertae Sedis	unclassified_G_Incertae Sedis
SV1785	K Bacteria	P Firmicutes	C Bacilli	O Erysipelotrichales	F Erysipelotrichaceae	G Faecalibaculum	unclassified G Faecalibaculum
SV1786	K Bacteria	P Firmicutes	C Clostridia	O Lachnospirales	F Lachnospiraceae	unclassified F Lachnospiraceae	unclassified F Lachnospiraceae
SV1787	K Bacteria	P Bacteroidota	C Bacteroidia	O Bacteroidales	F Marinifilaceae	G Odoribacter	unclassified G Odoribacter
SV1788	K Bacteria	P Firmicutes	C Bacilli	O Erysipelotrichales	F Erysipelotrichaceae	G Turicibacter	unclassified G Turicibacter
SV1789	K Bacteria	P Firmicutes	C Clostridia	O Lachnospirales	F Lachnospiraceae	G Lachnoclostridium	unclassified G Lachnoclostridium
SV1790	K Bacteria	P Firmicutes	C Clostridia	O Lachnospirales	F Lachnospiraceae	G Lachnospiraceae UCG-001	unclassified G Lachnospiraceae UCG-001
SV1791	K Bacteria	P Firmicutes	C Clostridia	O Lachnospirales	F Lachnospiraceae	G GCA-900066575	unclassified G GCA-900066575
SV1792	K Bacteria	P Firmicutes	C Clostridia	O Oscillospirales	F Ruminococcaceae	unclassified F Ruminococcaceae	unclassified F Ruminococcaceae

SV1793	K Bacteria	P Firmicutes	C Clostridia	O Lachnospirales	F Lachnospiraceae	unclassified F Lachnospiraceae	unclassified F Lachnospiraceae
SV1794	K Bacteria	P Bacteroidota	C Bacteroidia	O Bacteroidales	F Prevotellaceae	G Prevotellaceae UCG-001	unclassified G Prevotellaceae UCG-001
SV1795	K Bacteria	P Firmicutes	C Clostridia	O Oscillospirales	F Oscillospiraceae	G NK4A214 group	unclassified G NK4A214 group
SV1796	K Bacteria	P Firmicutes	C Clostridia	O Clostridia UCG-014	unclassified O Clostridia UCG-014	unclassified O Clostridia UCG-014	unclassified O Clostridia UCG-014
SV1797	K Bacteria	P Firmicutes	C Clostridia	O Oscillospirales	F Oscillospiraceae	G Colidextribacter	unclassified G Colidextribacter
SV1798	K Bacteria	P Firmicutes	C Clostridia	O Clostridiales	F Clostridiaceae	G Clostridium sensu stricto 1	unclassified G Clostridium sensu stricto 1
SV1799	K Bacteria	P Firmicutes	C Clostridia	O Oscillospirales	F [Eubacterium] coprostanoligenes group	unclassified F [Eubacterium] coprostanoligenes group	unclassified F [Eubacterium] coprostanoligenes group
SV1800	K Bacteria	P Firmicutes	C Clostridia	O Oscillospirales	F Oscillospiraceae	unclassified F Oscillospiraceae	unclassified F Oscillospiraceae
SV1801	K Bacteria	P Firmicutes	C Clostridia	O Lachnospirales	F Lachnospiraceae	G Lachnospiraceae NK4A136 group	unclassified G Lachnospiraceae NK4A136 group
SV1802	K Bacteria	P Firmicutes	C Clostridia	O Lachnospirales	F Lachnospiraceae	G GCA-900066575	unclassified G GCA-900066575
SV1803	K Bacteria	P Firmicutes	C Clostridia	O Lachnospirales	F Lachnospiraceae	G Lachnospiraceae NK4A136 group	unclassified G Lachnospiraceae NK4A136 group
SV1804	K Bacteria	P Firmicutes	C Clostridia	O Clostridia vadinBB60 group	unclassified O Clostridia vadinBB60 group	unclassified O Clostridia vadinBB60 group	unclassified O Clostridia vadinBB60 group
SV1805	K Bacteria	P Firmicutes	C Clostridia	O Lachnospirales	F Lachnospiraceae	G Roseburia	unclassified G Roseburia
SV1806	K Bacteria	P Firmicutes	C Clostridia	O Oscillospirales	F Ruminococcaceae	unclassified F Ruminococcaceae	unclassified F Ruminococcaceae
SV1807	K Bacteria	P Bacteroidota	C Bacteroidia	O Bacteroidales	F Rikenellaceae	G Alistipes	unclassified G Alistipes
SV1808	K Bacteria	P Firmicutes	C Clostridia	O Oscillospirales	F Oscillospiraceae	G Oscillibacter	unclassified G Oscillibacter
SV1809	K Bacteria	P Firmicutes	C Bacilli	O Erysipelotrichales	F Erysipelotrichaceae	G Turicibacter	unclassified G Turicibacter
SV1810	K Bacteria	P Firmicutes	C Clostridia	O Oscillospirales	F Oscillospiraceae	unclassified F Oscillospiraceae	unclassified F Oscillospiraceae
SV1811	K Bacteria	P Firmicutes	C Clostridia	O Lachnospirales	F Lachnospiraceae	unclassified F Lachnospiraceae	unclassified F Lachnospiraceae
SV1812	K Bacteria	P Firmicutes	C Clostridia	O Lachnospirales	F Lachnospiraceae	unclassified F Lachnospiraceae	unclassified F Lachnospiraceae
SV1813	K Bacteria	P Firmicutes	C Clostridia	O Lachnospirales	F Lachnospiraceae	G Lachnospiraceae NK4A136 group	unclassified G Lachnospiraceae NK4A136 group
SV1814	K Bacteria	P Firmicutes	C Clostridia	O Lachnospirales	F Lachnospiraceae	unclassified F Lachnospiraceae	unclassified F Lachnospiraceae
SV1815	K Bacteria	P Firmicutes	C Clostridia	O Oscillospirales	F Oscillospiraceae	unclassified F Oscillospiraceae	unclassified F Oscillospiraceae
SV1816	K Bacteria	P Firmicutes	C Clostridia	O Lachnospirales	F Lachnospiraceae	unclassified F Lachnospiraceae	unclassified F Lachnospiraceae
SV1817	K Bacteria	P Firmicutes	C Clostridia	O Clostridia UCG-014	unclassified O Clostridia UCG-014	unclassified O Clostridia UCG-014	unclassified O Clostridia UCG-014
SV1818	K Bacteria	P Firmicutes	C Clostridia	O Lachnospirales	F Lachnospiraceae	unclassified F Lachnospiraceae	unclassified F Lachnospiraceae
SV1819	K Bacteria	P Firmicutes	C Clostridia	O Oscillospirales	F Oscillospiraceae	unclassified F Oscillospiraceae	unclassified F Oscillospiraceae
SV1820	K Bacteria	P Firmicutes	C Clostridia	O Clostridia UCG-014	unclassified O Clostridia UCG-014	unclassified O Clostridia UCG-014	unclassified O Clostridia UCG-014
SV1821	K Bacteria	P Bacteroidota	C Bacteroidia	O Bacteroidales	F Muribaculaceae	unclassified F Muribaculaceae	unclassified F Muribaculaceae
SV1822	K Bacteria	P Firmicutes	C Clostridia	O Lachnospirales	F Lachnospiraceae	G Lachnospiraceae UCG-001	unclassified G Lachnospiraceae UCG-001
SV1823	K Bacteria	P Firmicutes	C Clostridia	O Lachnospirales	F Lachnospiraceae	G Lachnospiraceae UCG-001	unclassified G Lachnospiraceae UCG-001
SV1824	K Bacteria	P Firmicutes	C Clostridia	O Clostridiales	F Clostridiaceae	G Clostridium sensu stricto 1	unclassified G Clostridium sensu stricto 1
SV1825	K Bacteria	P Firmicutes	C Clostridia	O Lachnospirales	F Lachnospiraceae	G Lachnospiraceae NK4A136 group	unclassified G Lachnospiraceae NK4A136 group
SV1826	K Bacteria	P Firmicutes	C Clostridia	O Oscillospirales	F Oscillospiraceae	G Colidextribacter	unclassified G Colidextribacter
SV1827	K Bacteria	P Firmicutes	C Clostridia	O Oscillospirales	F Oscillospiraceae	G Colidextribacter	unclassified G Colidextribacter
SV1828	K Bacteria	P Firmicutes	C Clostridia	O Oscillospirales	F Ruminococcaceae	G Incertae Sedis	unclassified G Incertae Sedis
SV1829	K Bacteria	P Bacteroidota	C Bacteroidia	O Bacteroidales	F Muribaculaceae	unclassified F Muribaculaceae	unclassified F Muribaculaceae
SV1830	K Bacteria	P Firmicutes	C Clostridia	O Oscillospirales	F Oscillospiraceae	G Oscillibacter	unclassified G Oscillibacter
SV1831	K Bacteria	P Firmicutes	C Clostridia	O Oscillospirales	F Ruminococcaceae	G UBA1819	unclassified G UBA1819
SV1832	K Bacteria	P Firmicutes	C Clostridia	O Lachnospirales	F Lachnospiraceae	G Acetatifactor	unclassified G Acetatifactor
SV1833	K Bacteria	P Bacteroidota	C Bacteroidia	O Bacteroidales	F Muribaculaceae	unclassified F Muribaculaceae	unclassified F Muribaculaceae
SV1834	K Bacteria	P Firmicutes	C Clostridia	O Oscillospirales	F Oscillospiraceae	unclassified F Oscillospiraceae	unclassified F Oscillospiraceae
SV1835	K Bacteria	P Firmicutes	C Clostridia	O Lachnospirales	F Lachnospiraceae	G Lachnospiraceae UCG-006	unclassified G Lachnospiraceae UCG-006
SV1836	K Bacteria	P Bacteroidota	C Bacteroidia	O Bacteroidales	F Muribaculaceae	unclassified F Muribaculaceae	unclassified F Muribaculaceae
SV1837	K Bacteria	P Firmicutes	C Clostridia	O Lachnospirales	F Lachnospiraceae	G A2	unclassified G A2
SV1838	K Bacteria	P Firmicutes	C Bacilli	O Erysipelotrichales	F Erysipelotrichaceae	G Faecalibaculum	unclassified G Faecalibaculum
SV1839	K_Bacteria	P_Bacteroidota	C_Bacteroidia	O_Bacteroidales	F_Rikenellaceae	G_Alistipes	unclassified_G_Alistipes
SV1840	K Bacteria	P Firmicutes	C Clostridia	O Clostridia UCG-014	unclassified O Clostridia UCG-014	unclassified O Clostridia UCG-014	unclassified O Clostridia UCG-014
SV1841	K Bacteria	P Bacteroidota	C Bacteroidia	O Bacteroidales	F Marinifilaceae	G Odoribacter	unclassified G Odoribacter
SV1842	K Bacteria	P Firmicutes	C Clostridia	O Clostridia UCG-014	unclassified O Clostridia UCG-014	unclassified O Clostridia UCG-014	unclassified O Clostridia UCG-014
SV1843	K Bacteria	P Firmicutes	C Clostridia	O Lachnospirales	F Lachnospiraceae	G Lachnospiraceae NK4A136 group	unclassified G Lachnospiraceae NK4A136 group
SV1844	K Bacteria	P Firmicutes	C Clostridia	O Lachnospirales	F Lachnospiraceae	G Murimonas	S intestini
SV1845	K_Bacteria	P_Bacteroidota	C_Bacteroidia	O_Bacteroidales	F_Muribaculaceae	unclassified_F_Muribaculaceae	unclassified_F_Muribaculaceae
SV1846	K Bacteria	P Firmicutes	C Clostridia	O Oscillospirales	F Ruminococcaceae	G Incertae Sedis	unclassified G Incertae Sedis
SV1847	K Bacteria	P Firmicutes	C Clostridia	O Oscillospirales	F Ruminococcaceae	G Ruminococcus	unclassified G Ruminococcus
SV1848	K Bacteria	P Firmicutes	C Clostridia	O Lachnospirales	F Lachnospiraceae	G Roseburia	unclassified G Roseburia
SV1849	K Bacteria	P Firmicutes	C Clostridia	O Lachnospirales	F Lachnospiraceae	G Lachnospiraceae UCG-006	unclassified G Lachnospiraceae UCG-006
SV1850	K Bacteria	P Cyanobacteria	C Vampirivibrionia	O Gastranaerophilales	unclassified O Gastranaerophilales	unclassified O Gastranaerophilales	unclassified O Gastranaerophilales
SV1851	K_Bacteria	P_Firmicutes	C_Clostridia	O_Lachnospirales	F_Lachnospiraceae	G_Roseburia	unclassified_G_Roseburia
SV1852	K Bacteria	P Firmicutes	C Clostridia	unclassified C Clostridia	unclassified C Clostridia	unclassified C Clostridia	unclassified C Clostridia
SV1853	K Bacteria	P Firmicutes	C Clostridia	O Clostridia UCG-014	unclassified O Clostridia UCG-014	unclassified O Clostridia UCG-014	unclassified O Clostridia UCG-014
SV1854	K Bacteria	P Firmicutes	C Clostridia	O Lachnospirales	F Lachnospiraceae	G Lachnospiraceae FCS020 group	unclassified G Lachnospiraceae FCS020 group
SV1855	K Bacteria	P Firmicutes	C Clostridia	O Lachnospirales	F Lachnospiraceae	unclassified F Lachnospiraceae	unclassified F Lachnospiraceae
SV1856	K Bacteria	P Firmicutes	C Clostridia	O Lachnospirales	F Lachnospiraceae	unclassified F Lachnospiraceae	unclassified F Lachnospiraceae
SV1857	K_Bacteria	P_Bacteroidota	C_Bacteroidia	O_Bacteroidales	F_Muribaculaceae	unclassified_F_Muribaculaceae	unclassified_F_Muribaculaceae
SV1858	K Bacteria	P Firmicutes	C Clostridia	O Lachnospirales	F Lachnospiraceae	unclassified F Lachnospiraceae	unclassified F Lachnospiraceae
SV1859	K Bacteria	P Firmicutes	C Clostridia	O Peptostreptococcales-Tissierellales	F Anaerovoracaceae	G Family XIII AD3011 group	unclassified G Family XIII AD3011 group
SV1860	K Bacteria	P Firmicutes	C Clostridia	O Peptostreptococcales-Tissierellales	F Peptostreptococcaceae	unclassified F Peptostreptococcaceae	unclassified F Peptostreptococcaceae
SV1861	K Bacteria	P Firmicutes	C Clostridia	O Peptostreptococcales-Tissierellales	F Anaerovoracaceae	G [Eubacterium] brachy group	unclassified G [Eubacterium] brachy group
SV1862	K Bacteria	P Firmicutes	C Bacilli	O Erysipelotrichales	F Erysipelatoclostridiaceae	G Erysipelatoclostridium	unclassified G Erysipelatoclostridium
SV1863	K_Bacteria	P_Firmicutes	C_Clostridia	O_Lachnospirales	F_Lachnospiraceae	unclassified_F_Lachnospiraceae	unclassified_F_Lachnospiraceae
SV1864	K Bacteria	P Firmicutes	C Clostridia	O Oscillospirales	F Oscillospiraceae	G UCG-003	unclassified G UCG-003
SV1865	K Bacteria	P Firmicutes	C Clostridia	O Lachnospirales	F Lachnospiraceae	unclassified F Lachnospiraceae	unclassified F Lachnospiraceae
SV1866	K Bacteria	P Firmicutes	C Clostridia	O Lachnospirales	F Lachnospiraceae	unclassified F Lachnospiraceae	unclassified F Lachnospiraceae
SV1867	K Bacteria	P Firmicutes	C Clostridia	O Lachnospirales	F Lachnospiraceae	G Lachnoclostridium	unclassified G Lachnoclostridium
SV1868	K Bacteria	P Firmicutes	C Clostridia	O Peptostreptococcales-Tissierellales	F Peptostreptococcaceae	unclassified F Peptostreptococcaceae	unclassified F Peptostreptococcaceae
SV1869	K Bacteria	P Bacteroidota	C Bacteroidia	O Bacteroidales	F Muribaculaceae	unclassified F Muribaculaceae	unclassified F Muribaculaceae
SV1870	K Bacteria	P Firmicutes	C Clostridia	O Lachnospirales	F Lachnospiraceae	G Lachnospiraceae UCG-006	unclassified G Lachnospiraceae UCG-006
SV1871	K Bacteria	P Firmicutes	C Clostridia	O Lachnospirales	F Lachnospiraceae	G Lachnospiraceae NK4A136 group	unclassified G Lachnospiraceae NK4A136 group
SV1872	K Bacteria	P Firmicutes	C Clostridia	O Clostridia UCG-014	unclassified O Clostridia UCG-014	unclassified O Clostridia UCG-014	unclassified O Clostridia UCG-014
SV1873	K Bacteria	P Firmicutes	C Bacilli	O Staphylococcales	F Staphylococcaceae	G Staphylococcus	unclassified G Staphylococcus
SV1874	K Bacteria	P Firmicutes	C Clostridia	O Lachnospirales	F Lachnospiraceae	G [Ruminococcus] gauvreauii group	unclassified G [Ruminococcus] gauvreauii group
SV1875	K Bacteria	P Firmicutes	C Clostridia	O Oscillospirales	F Oscillospiraceae	G UCG-003	unclassified G UCG-003
SV1876	K Bacteria	P Desulfobacterota	C Desulfovibrionia	O Desulfovibrionales	F Desulfovibrionaceae	G Bilophila	unclassified G Bilophila
SV1877	K Bacteria	P Firmicutes	C Clostridia	O Lachnospirales	F Lachnospiraceae	G Dorea	unclassified G Dorea
SV1878	K Bacteria	P Firmicutes	C Clostridia	O Oscillospirales	F Ruminococcaceae	unclassified F Ruminococcaceae	unclassified F Ruminococcaceae
SV1879	K Bacteria	P Firmicutes	C Clostridia	O Lachnospirales	F Lachnospiraceae	unclassified F Lachnospiraceae	unclassified F Lachnospiraceae
SV1880	K Bacteria	P Bacteroidota	C Bacteroidia	O Bacteroidales	F Rikenellaceae	G Alistipes	unclassified G Alistipes
SV1881	K Bacteria	P Bacteroidota	C Bacteroidia	O Bacteroidales	F Muribaculaceae	unclassified F Muribaculaceae	unclassified F Muribaculaceae
SV1882	K Bacteria	P Firmicutes	C Clostridia	O Clostridia vadinBB60 group	unclassified O Clostridia vadinBB60 group	unclassified O Clostridia vadinBB60 group	unclassified O Clostridia vadinBB60 group
SV1883	K Bacteria	P Bacteroidota	C Bacteroidia	O Bacteroidales	F Muribaculaceae	unclassified F Muribaculaceae	unclassified F Muribaculaceae
SV1884	K Bacteria	P Firmicutes	C Bacilli	O Lactobacillales	F Lactobacillaceae	unclassified F Lactobacillaceae	unclassified F Lactobacillaceae
SV1885	K Bacteria	P Firmicutes	C Clostridia	O Lachnospirales	F Lachnospiraceae	G Lachnospiraceae NK4A136 group	unclassified G Lachnospiraceae NK4A136 group
SV1886	K Bacteria	P Firmicutes	C Clostridia	O Lachnospirales	F Lachnospiraceae	G Lachnoclostridium	unclassified G Lachnoclostridium
SV1887	K Bacteria	P Firmicutes	C Clostridia	O Lachnospirales	F Lachnospiraceae	G Tuzzerella	unclassified G Tuzzerella
SV1888	K Bacteria	P Firmicutes	C Clostridia	O Oscillospirales	F Oscillospiraceae	G Oscillibacter	unclassified G Oscillibacter
SV1889	K Bacteria	P Firmicutes	C Clostridia	O Lachnospirales	F Lachnospiraceae	G Lachnoclostridium	unclassified G Lachnoclostridium
SV1890	K Bacteria	P Firmicutes	C Bacilli	O Lactobacillales	F Lactobacillaceae	unclassified F Lactobacillaceae	unclassified F Lactobacillaceae
SV1891	K Bacteria	P Firmicutes	C Clostridia	O Lachnospirales	F Lachnospiraceae	G Lachnospiraceae NK4A136 group	unclassified G Lachnospiraceae NK4A136 group
SV1892	K Bacteria	P Firmicutes	C Clostridia	O Clostridia UCG-014	unclassified O Clostridia UCG-014	unclassified O Clostridia UCG-014	unclassified O Clostridia UCG-014
SV1893	K Bacteria	P Firmicutes	C Clostridia	O Lachnospirales	F Lachnospiraceae	G Lachnospiraceae NK4A136 group	unclassified G Lachnospiraceae NK4A136 group
SV1894	K Bacteria	P Firmicutes	C Clostridia	O Lachnospirales	F Lachnospiraceae	G [Eubacterium] oxidoreducens group	unclassified G [Eubacterium] oxidoreducens group
SV1895	K Bacteria	P Firmicutes	C Clostridia	O Lachnospirales	F Lachnospiraceae	unclassified F Lachnospiraceae	unclassified F Lachnospiraceae
SV1896	K Bacteria	P Firmicutes	C Clostridia	O Lachnospirales	F Lachnospiraceae	G Roseburia	unclassified G Roseburia
SV1897	K Bacteria	P Firmicutes	C Clostridia	O Christensenellales	F Christensenellaceae	unclassified F Christensenellaceae	unclassified F Christensenellaceae
SV1898	K Bacteria	P Firmicutes	C Bacilli	O Lactobacillales	F Lactobacillaceae	G Ligilactobacillus	unclassified G Ligilactobacillus
SV1899	K Bacteria	P Actinobacteriota	C Coriobacteriia	O Coriobacteriales	F Eggerthellaceae	G Enterorhabdus	unclassified G Enterorhabdus
SV1900	K Bacteria	P Firmicutes	C Bacilli	O Lactobacillales	F Lactobacillaceae	unclassified F Lactobacillaceae	unclassified F Lactobacillaceae
SV1901	K Bacteria	P Firmicutes	C Clostridia	O Oscillospirales	F Ruminococcaceae	unclassified F Ruminococcaceae	unclassified F Ruminococcaceae
SV1902	K Bacteria	P Firmicutes	C Clostridia	O Lachnospirales	F Lachnospiraceae	G Lachnospiraceae UCG-001	unclassified G Lachnospiraceae UCG-001
SV1903	K Bacteria	P Firmicutes	C Clostridia	O Oscillospirales	F Oscillospiraceae	G Colidextribacter	unclassified G Colidextribacter
SV1904	K_Bacteria	P_Firmicutes	C_Clostridia	O_Oscillospirales	F_Ruminococcaceae	G_[Eubacterium] siraeum group	unclassified_G_[Eubacterium] siraeum group
SV1905	K Bacteria	P Firmicutes	C Clostridia	O Oscillospirales	F Oscillospiraceae	G Intestinimonas	unclassified G Intestinimonas
SV1906	K Bacteria	P Firmicutes	C Clostridia	O Lachnospirales	F Lachnospiraceae	unclassified F Lachnospiraceae	unclassified F Lachnospiraceae
SV1907	K Bacteria	P Firmicutes	C Clostridia	O Oscillospirales	F Ruminococcaceae	G Incertae Sedis	unclassified G Incertae Sedis
SV1908	K Bacteria	P Firmicutes	C Clostridia	O Clostridiales	F Clostridiaceae	G Clostridium sensu stricto 1	unclassified G Clostridium sensu stricto 1
SV1909	K Bacteria	P Firmicutes	C Clostridia	O Lachnospirales	F Lachnospiraceae	unclassified F Lachnospiraceae	unclassified F Lachnospiraceae
SV1910	K_Bacteria	P_Firmicutes	C_Clostridia	O_Lachnospirales	F_Lachnospiraceae	unclassified_F_Lachnospiraceae	unclassified_F_Lachnospiraceae
SV1911	K Bacteria	P Firmicutes	C Clostridia	O Oscillospirales	F Ruminococcaceae	G Incertae Sedis	unclassified G Incertae Sedis
SV1912	K Bacteria	P Firmicutes	C Clostridia	O Lachnospirales	F Lachnospiraceae	unclassified F Lachnospiraceae	unclassified F Lachnospiraceae
SV1913	K Bacteria	P Firmicutes	C Clostridia	O Lachnospirales	F Lachnospiraceae	G Acetatifactor	unclassified G Acetatifactor
SV1914	K Bacteria	P Firmicutes	C Clostridia	O Oscillospirales	F Oscillospiraceae	unclassified F Oscillospiraceae	unclassified F Oscillospiraceae
SV1915	K Bacteria	P Firmicutes	C Clostridia	O Lachnospirales	F Lachnospiraceae	G ASF356	unclassified G ASF356
SV1916	K_Bacteria	P_Firmicutes	C_Clostridia	O_Lachnospirales	F_Lachnospiraceae	G_Roseburia	unclassified_G_Roseburia
SV1917	K Bacteria	P Firmicutes	C Clostridia	O Clostridiales	F Clostridiaceae	unclassified F Clostridiaceae	unclassified F Clostridiaceae
SV1918	K Bacteria	P Firmicutes	C Clostridia	O Lachnospirales	F Lachnospiraceae	unclassified F Lachnospiraceae	unclassified F Lachnospiraceae
SV1919	K Bacteria	P Firmicutes	C Clostridia	O Peptostreptococcales-Tissierellales	F Anaerovoracaceae	G [Eubacterium] brachy group	unclassified G [Eubacterium] brachy group
SV1920	K Bacteria	P Firmicutes	C Clostridia	O Oscillospirales	F Ruminococcaceae	G Incertae Sedis	unclassified G Incertae Sedis
SV1921	K Bacteria	P Firmicutes	C Clostridia	O Lachnospirales	F Lachnospiraceae	unclassified F Lachnospiraceae	unclassified F Lachnospiraceae
SV1922	K_Bacteria	P_Firmicutes	C_Clostridia	O_Oscillospirales	F_Oscillospiraceae	unclassified_F_Oscillospiraceae	unclassified_F_Oscillospiraceae
SV1923	K Bacteria	P Firmicutes	C Clostridia	O Lachnospirales	F Lachnospiraceae	G Lachnospiraceae NK4A136 group	unclassified G Lachnospiraceae NK4A136 group
SV1924	K Bacteria	P Firmicutes	C Clostridia	O Oscillospirales	F Ruminococcaceae	unclassified F Ruminococcaceae	unclassified F Ruminococcaceae
SV1925	K Bacteria	P Firmicutes	C Clostridia	O Oscillospirales	F Ruminococcaceae	G Incertae Sedis	unclassified G Incertae Sedis
SV1926	K Bacteria	P Firmicutes	C Clostridia	O Lachnospirales	F Lachnospiraceae	unclassified F Lachnospiraceae	unclassified F Lachnospiraceae
SV1927	K Bacteria	P Firmicutes	C Clostridia	O Lachnospirales	F Lachnospiraceae	G Roseburia	unclassified G Roseburia
SV1928	K Bacteria	P Firmicutes	C Clostridia	O Clostridia UCG-014	unclassified O Clostridia UCG-014	unclassified O Clostridia UCG-014	unclassified O Clostridia UCG-014
SV1929	K Bacteria	P Firmicutes	C Clostridia	O Oscillospirales	F Ruminococcaceae	unclassified F Ruminococcaceae	unclassified F Ruminococcaceae
SV1930	K Bacteria	P Firmicutes	C Clostridia	O Peptostreptococcales-Tissierellales	F Anaerovoracaceae	G [Eubacterium] nodatum group	unclassified G [Eubacterium] nodatum group

SV1931	K Bacteria	P Firmicutes	C Clostridia	unclassified C Clostridia	unclassified C Clostridia	unclassified C Clostridia	unclassified C Clostridia
SV1932	K Bacteria	P Firmicutes	C Clostridia	O Lachnospirales	F Lachnospiraceae	G Marvinbryantia	unclassified G Marvinbryantia
SV1933	K Bacteria	P Firmicutes	C Clostridia	O Clostridia UCG-014	unclassified O Clostridia UCG-014	unclassified O Clostridia UCG-014	unclassified O Clostridia UCG-014
SV1934	K Bacteria	P Firmicutes	C Clostridia	O Lachnospirales	F Lachnospiraceae	G Lachnospiraceae NK4A136 group	unclassified G Lachnospiraceae NK4A136 group
SV1935	K Bacteria	P Firmicutes	C Clostridia	O Oscillospirales	F Oscillospiraceae	unclassified F Oscillospiraceae	unclassified F Oscillospiraceae
SV1936	K Bacteria	P Firmicutes	C Clostridia	O Oscillospirales	F Oscillospiraceae	G Oscillibacter	unclassified G Oscillibacter
SV1937	K Bacteria	P Firmicutes	C Clostridia	O Lachnospirales	F Lachnospiraceae	unclassified F Lachnospiraceae	unclassified F Lachnospiraceae
SV1938	K Bacteria	P Firmicutes	C Clostridia	O Lachnospirales	F Lachnospiraceae	unclassified F Lachnospiraceae	unclassified F Lachnospiraceae
SV1939	K Bacteria	P Firmicutes	C Clostridia	O Peptostreptococcales-Tissierellales	F Peptostreptococcaceae	unclassified F Peptostreptococcaceae	unclassified F Peptostreptococcaceae
SV1940	K Bacteria	P Firmicutes	C Clostridia	O Oscillospirales	F Oscillospiraceae	G Oscillospira	unclassified G Oscillospira
SV1941	K Bacteria	P Firmicutes	C Clostridia	O Clostridia UCG-014	unclassified O Clostridia UCG-014	unclassified O Clostridia UCG-014	unclassified O Clostridia UCG-014
SV1942	K Bacteria	P Firmicutes	C Clostridia	O Clostridia UCG-014	unclassified O Clostridia UCG-014	unclassified O Clostridia UCG-014	unclassified O Clostridia UCG-014
SV1943	K Bacteria	P Firmicutes	C Clostridia	O Lachnospirales	F Lachnospiraceae	unclassified F Lachnospiraceae	unclassified F Lachnospiraceae
SV1944	K Bacteria	P Firmicutes	C Bacilli	O Erysipelotrichales	F Erysipelotrichaceae	G [Clostridium] innocuum group	unclassified G [Clostridium] innocuum group
SV1945	K Bacteria	P Firmicutes	C Clostridia	O Lachnospirales	F Lachnospiraceae	G Lachnoclostridium	unclassified G Lachnoclostridium
SV1946	K Bacteria	P Firmicutes	C Clostridia	O Clostridia UCG-014	unclassified O Clostridia UCG-014	unclassified O Clostridia UCG-014	unclassified O Clostridia UCG-014
SV1947	K Bacteria	P Firmicutes	C Clostridia	O Lachnospirales	F Lachnospiraceae	unclassified F Lachnospiraceae	unclassified F Lachnospiraceae
SV1948	K Bacteria	P Firmicutes	C Clostridia	O Clostridiales	F Clostridiaceae	G Clostridium sensu stricto 1	unclassified G Clostridium sensu stricto 1
SV1949	K Bacteria	P Firmicutes	C Clostridia	O Peptostreptococcales-Tissierellales	F Peptostreptococcaceae	unclassified F Peptostreptococcaceae	unclassified F Peptostreptococcaceae
SV1950	K Bacteria	P Firmicutes	C Clostridia	O Clostridia vadinBB60 group	unclassified O Clostridia vadinBB60 group	unclassified O Clostridia vadinBB60 group	unclassified O Clostridia vadinBB60 group
SV1951	K Bacteria	P Firmicutes	C Clostridia	O Oscillospirales	F Oscillospiraceae	unclassified F Oscillospiraceae	unclassified F Oscillospiraceae
SV1952	K Bacteria	P Firmicutes	C Clostridia	O Oscillospirales	F Ruminococcaceae	unclassified F Ruminococcaceae	unclassified F Ruminococcaceae
SV1953	K Bacteria	P Firmicutes	C Clostridia	O Clostridia UCG-014	unclassified O Clostridia UCG-014	unclassified O Clostridia UCG-014	unclassified O Clostridia UCG-014
SV1954	K Bacteria	P Firmicutes	C Clostridia	O Lachnospirales	F Lachnospiraceae	G Lachnospiraceae FCS020 group	unclassified G Lachnospiraceae FCS020 group
SV1955	K Bacteria	P Firmicutes	C Clostridia	O Lachnospirales	F Lachnospiraceae	G Lachnospiraceae UCG-001	unclassified G Lachnospiraceae UCG-001
SV1956	K Bacteria	P Firmicutes	C Clostridia	O Oscillospirales	F Ruminococcaceae	G Incertae Sedis	unclassified G Incertae Sedis
SV1957	K Bacteria	P Firmicutes	C Clostridia	O Oscillospirales	F Ruminococcaceae	unclassified F Ruminococcaceae	unclassified F Ruminococcaceae
SV1958	K Bacteria	P Firmicutes	C Clostridia	O Lachnospirales	F Lachnospiraceae	G [Ruminococcus] gnavus group	unclassified G [Ruminococcus] gnavus group
SV1959	K Bacteria	P Firmicutes	C Clostridia	O Lachnospirales	F Lachnospiraceae	G Lachnospiraceae UCG-001	unclassified G Lachnospiraceae UCG-001
SV1960	K Bacteria	P Firmicutes	C Clostridia	O Lachnospirales	F Lachnospiraceae	G Lachnospiraceae NK4A136 group	unclassified G Lachnospiraceae NK4A136 group
SV1961	K Bacteria	P Firmicutes	C Clostridia	O Lachnospirales	F Lachnospiraceae	unclassified F Lachnospiraceae	unclassified F Lachnospiraceae
SV1962	K Bacteria	P Firmicutes	C Clostridia	O Oscillospirales	F Oscillospiraceae	G Colidextribacter	unclassified G Colidextribacter
SV1963	K Bacteria	P Firmicutes	C Clostridia	O Lachnospirales	F Lachnospiraceae	G Lachnospiraceae UCG-001	unclassified G Lachnospiraceae UCG-001
SV1964	K Bacteria	P Firmicutes	C Clostridia	O Oscillospirales	F Oscillospiraceae	unclassified F Oscillospiraceae	unclassified F Oscillospiraceae
SV1965	K Bacteria	P Firmicutes	C Clostridia	O Oscillospirales	F Ruminococcaceae	unclassified F Ruminococcaceae	unclassified F Ruminococcaceae
SV1966	K Bacteria	P Firmicutes	C Clostridia	O Oscillospirales	F Ruminococcaceae	G Ruminococcus	unclassified G Ruminococcus
SV1967	K Bacteria	P Patescibacteria	C Saccharimonadia	O Saccharimonadales	F Saccharimonadaceae	G Candidatus Saccharimonas	unclassified G Candidatus Saccharimonas
SV1968	K Bacteria	P Firmicutes	C Clostridia	O Lachnospirales	F Lachnospiraceae	unclassified F Lachnospiraceae	unclassified F Lachnospiraceae
SV1969	K Bacteria	P Firmicutes	C Clostridia	O Lachnospirales	F Lachnospiraceae	G Dorea	unclassified G Dorea
SV1970	K Bacteria	P Firmicutes	C Clostridia	O Oscillospirales	F Ruminococcaceae	G Ruminococcus	unclassified G Ruminococcus
SV1971	K Bacteria	P Firmicutes	C Bacilli	O RF39	unclassified O RF39	unclassified O RF39	unclassified O RF39
SV1972	K Bacteria	P Actinobacteriota	C Coriobacteriia	O Coriobacteriales	F Eggerthellaceae	G Enterorhabdus	unclassified G Enterorhabdus
SV1973	K Bacteria	P Firmicutes	C Clostridia	O Oscillospirales	F Butyricicoccaceae	G Butyricicoccus	unclassified G Butyricicoccus
SV1974	K Bacteria	P Firmicutes	C Clostridia	O Lachnospirales	F Lachnospiraceae	G Roseburia	unclassified G Roseburia
SV1975	K Bacteria	P Firmicutes	C Clostridia	O Lachnospirales	F Lachnospiraceae	unclassified F Lachnospiraceae	unclassified F Lachnospiraceae
SV1976	K Bacteria	P Firmicutes	C Clostridia	O Oscillospirales	F [Eubacterium] coprostanoligenes group	unclassified F [Eubacterium] coprostanoligenes group	unclassified F [Eubacterium] coprostanoligenes group
SV1977	K_Bacteria	P_Firmicutes	C_Clostridia	O_Oscillospirales	F_Ruminococcaceae	G_UBA1819	unclassified_G_UBA1819
SV1978	K Bacteria	P Firmicutes	C Clostridia	O Lachnospirales	F Lachnospiraceae	G Lachnospiraceae NK4A136 group	unclassified G Lachnospiraceae NK4A136 group
SV1979	K Bacteria	P Firmicutes	C Clostridia	O Oscillospirales	F Ruminococcaceae	G Incertae Sedis	unclassified G Incertae Sedis
SV1980	K Bacteria	P Firmicutes	C Bacilli	O Erysipelotrichales	F Erysipelotrichaceae	G Dubosiella	unclassified G Dubosiella
SV1981	K Bacteria	P Firmicutes	C Clostridia	O Oscillospirales	F Ruminococcaceae	G Ruminococcus	unclassified G Ruminococcus
SV1982	K Bacteria	P Firmicutes	C Clostridia	O Lachnospirales	F Lachnospiraceae	G Lachnospiraceae NK4A136 group	unclassified G Lachnospiraceae NK4A136 group
SV1983	K_Bacteria	P_Bacteroidota	C_Bacteroidia	O_Bacteroidales	F_Muribaculaceae	unclassified_F_Muribaculaceae	unclassified_F_Muribaculaceae
SV1984	K Bacteria	P Bacteroidota	C Bacteroidia	O Bacteroidales	F Muribaculaceae	unclassified F Muribaculaceae	unclassified F Muribaculaceae
SV1985	K Bacteria	P Firmicutes	C Clostridia	O Lachnospirales	F Lachnospiraceae	G A2	unclassified G A2
SV1986	K Bacteria	P Bacteroidota	C Bacteroidia	O Bacteroidales	F Muribaculaceae	unclassified F Muribaculaceae	unclassified F Muribaculaceae
SV1987	K Bacteria	P Firmicutes	C Clostridia	O Lachnospirales	F Lachnospiraceae	G Tuzzerella	unclassified G Tuzzerella
SV1988	K Bacteria	P Bacteroidota	C Bacteroidia	O Bacteroidales	F Muribaculaceae	unclassified F Muribaculaceae	unclassified F Muribaculaceae
SV1989	K_Bacteria	P_Firmicutes	C_Clostridia	O_Oscillospirales	F_Ruminococcaceae	G_Ruminococcus	unclassified_G_Ruminococcus
SV1990	K Bacteria	P Firmicutes	C Clostridia	O Lachnospirales	F Lachnospiraceae	G Acetatifactor	unclassified G Acetatifactor
SV1991	K Bacteria	P Firmicutes	C Clostridia	O Oscillospirales	F Ruminococcaceae	unclassified F Ruminococcaceae	unclassified F Ruminococcaceae
SV1992	K Bacteria	P Bacteroidota	C Bacteroidia	O Bacteroidales	F Bacteroidaceae	G Bacteroides	unclassified G Bacteroides
SV1993	K Bacteria	P Firmicutes	C Clostridia	O Oscillospirales	F Oscillospiraceae	G UCG-005	unclassified G UCG-005
SV1994	K Bacteria	P Firmicutes	C Clostridia	O Oscillospirales	F Oscillospiraceae	unclassified F Oscillospiraceae	unclassified F Oscillospiraceae
SV1995	K_Bacteria	P_Firmicutes	C_Bacilli	O_Erysipelotrichales	F_Erysipelotrichaceae	unclassified_F_Erysipelotrichaceae	unclassified_F_Erysipelotrichaceae
SV1996	K Bacteria	P Firmicutes	C Clostridia	O Lachnospirales	F Lachnospiraceae	G Lachnoclostridium	unclassified G Lachnoclostridium
SV1997	K Bacteria	P Firmicutes	C Clostridia	O Lachnospirales	F Lachnospiraceae	unclassified F Lachnospiraceae	unclassified F Lachnospiraceae
SV1998	K Bacteria	P Bacteroidota	C Bacteroidia	O Bacteroidales	F Muribaculaceae	unclassified F Muribaculaceae	unclassified F Muribaculaceae
SV1999	K Bacteria	P Firmicutes	C Clostridia	O Lachnospirales	F Lachnospiraceae	G [Eubacterium] oxidoreducens group	unclassified G [Eubacterium] oxidoreducens group
SV2000	K Bacteria	P Bacteroidota	C Bacteroidia	O Bacteroidales	F Prevotellaceae	G Prevotellaceae UCG-001	unclassified G Prevotellaceae UCG-001
SV2001	K_Bacteria	P_Firmicutes	C_Clostridia	O_Lachnospirales	F_Lachnospiraceae	unclassified_F_Lachnospiraceae	unclassified_F_Lachnospiraceae
SV2002	K Bacteria	P Firmicutes	C Clostridia	O Clostridia UCG-014	unclassified O Clostridia UCG-014	unclassified O Clostridia UCG-014	unclassified O Clostridia UCG-014
SV2003	K Bacteria	P Firmicutes	C Clostridia	O Oscillospirales	F Oscillospiraceae	G Oscillospira	unclassified G Oscillospira
SV2004	K Bacteria	P Firmicutes	C Clostridia	O Lachnospirales	F Lachnospiraceae	G Tuzzerella	unclassified G Tuzzerella
SV2005	K Bacteria	P Firmicutes	C Clostridia	O Peptostreptococcales-Tissierellales	F Anaerovoracaceae	G [Eubacterium] brachy group	unclassified G [Eubacterium] brachy group
SV2006	K Bacteria	P Firmicutes	C Clostridia	O Oscillospirales	F Oscillospiraceae	G Colidextribacter	unclassified G Colidextribacter
SV2007	K Bacteria	P Firmicutes	C Clostridia	O Clostridia UCG-014	unclassified O Clostridia UCG-014	unclassified O Clostridia UCG-014	unclassified O Clostridia UCG-014
SV2008	K Bacteria	P Firmicutes	C Clostridia	O Oscillospirales	F Oscillospiraceae	G UCG-005	unclassified G UCG-005
SV2009	K Bacteria	P Firmicutes	C Clostridia	O Lachnospirales	F Lachnospiraceae	unclassified F Lachnospiraceae	unclassified F Lachnospiraceae
SV2010	K Bacteria	P Firmicutes	C Clostridia	O Oscillospirales	F Oscillospiraceae	G Intestinimonas	S butyriciproducens
SV2011	K Bacteria	P Firmicutes	C Clostridia	O Oscillospirales	F Oscillospiraceae	G Colidextribacter	unclassified G Colidextribacter
SV2012	K Bacteria	P Firmicutes	C Bacilli	O Lactobacillales	F Lactobacillaceae	G Ligilactobacillus	unclassified G Ligilactobacillus
SV2013	K Bacteria	P Firmicutes	C Bacilli	O Lactobacillales	F Lactobacillaceae	unclassified F Lactobacillaceae	unclassified F Lactobacillaceae
SV2014	K Bacteria	P Desulfobacterota	C Desulfovibrionia	O Desulfovibrionales	F Desulfovibrionaceae	unclassified F Desulfovibrionaceae	unclassified F Desulfovibrionaceae
SV2015	K Bacteria	P Firmicutes	C Clostridia	O Lachnospirales	F Lachnospiraceae	G GCA-900066575	unclassified G GCA-900066575
SV2016	K Bacteria	P Bacteroidota	C Bacteroidia	O Bacteroidales	F Muribaculaceae	unclassified F Muribaculaceae	unclassified F Muribaculaceae
SV2017	K Bacteria	P Firmicutes	C Clostridia	O Oscillospirales	F Ruminococcaceae	G UBA1819	unclassified G UBA1819
SV2018	K Bacteria	P Bacteroidota	C Bacteroidia	O Bacteroidales	F Muribaculaceae	unclassified F Muribaculaceae	unclassified F Muribaculaceae
SV2019	K Bacteria	P Firmicutes	C Clostridia	O Lachnospirales	F Lachnospiraceae	G Lachnospiraceae NK4A136 group	unclassified G Lachnospiraceae NK4A136 group
SV2020	K Bacteria	P Firmicutes	C Clostridia	O Lachnospirales	F Lachnospiraceae	G Lachnospiraceae NK4A136 group	unclassified G Lachnospiraceae NK4A136 group
SV2021	K Bacteria	P Bacteroidota	C Bacteroidia	O Bacteroidales	F Muribaculaceae	unclassified F Muribaculaceae	unclassified F Muribaculaceae
SV2022	K Bacteria	P Firmicutes	C Clostridia	O Lachnospirales	F Lachnospiraceae	G GCA-900066575	unclassified G GCA-900066575
SV2023	K Bacteria	P Firmicutes	C Clostridia	O Lachnospirales	F Lachnospiraceae	unclassified F Lachnospiraceae	unclassified F Lachnospiraceae
SV2024	K Bacteria	P Firmicutes	C Clostridia	O Lachnospirales	F Lachnospiraceae	G Lachnospiraceae UCG-006	unclassified G Lachnospiraceae UCG-006
SV2025	K Bacteria	P Firmicutes	C Clostridia	O Christensenellales	F Christensenellaceae	unclassified F Christensenellaceae	unclassified F Christensenellaceae
SV2026	K Bacteria	P Firmicutes	C Clostridia	O Clostridia vadinBB60 group	unclassified O Clostridia vadinBB60 group	unclassified O Clostridia vadinBB60 group	unclassified O Clostridia vadinBB60 group
SV2027	K Bacteria	P Bacteroidota	C Bacteroidia	O Bacteroidales	F Marinifilaceae	G Odoribacter	unclassified G Odoribacter
SV2028	K Bacteria	P Firmicutes	C Clostridia	O Oscillospirales	F Ruminococcaceae	G Harryflintia	unclassified G Harryflintia
SV2029	K Bacteria	P Firmicutes	C Clostridia	unclassified C Clostridia	unclassified C Clostridia	unclassified C Clostridia	unclassified C Clostridia
SV2030	K Bacteria	P Proteobacteria	C Alphaproteobacteria	O Rhodospirillales	unclassified O Rhodospirillales	unclassified O Rhodospirillales	unclassified O Rhodospirillales
SV2031	K Bacteria	P Firmicutes	C Bacilli	O Erysipelotrichales	F Erysipelotrichaceae	G [Clostridium] innocuum group	unclassified G [Clostridium] innocuum group
SV2032	K Bacteria	P Firmicutes	C Clostridia	O Lachnospirales	F Lachnospiraceae	G Lachnospiraceae NK4A136 group	unclassified G Lachnospiraceae NK4A136 group
SV2033	K Bacteria	P Firmicutes	C Clostridia	O Oscillospirales	F Ruminococcaceae	unclassified F Ruminococcaceae	unclassified F Ruminococcaceae
SV2034	K Bacteria	P Firmicutes	C Clostridia	O Lachnospirales	F Lachnospiraceae	unclassified F Lachnospiraceae	unclassified F Lachnospiraceae
SV2035	K Bacteria	P Firmicutes	C Clostridia	O Oscillospirales	F Ruminococcaceae	G Incertae Sedis	unclassified G Incertae Sedis
SV2036	K Bacteria	P Firmicutes	C Clostridia	O Clostridia UCG-014	unclassified O Clostridia UCG-014	unclassified O Clostridia UCG-014	unclassified O Clostridia UCG-014
SV2037	K Bacteria	P Firmicutes	C Clostridia	O Lachnospirales	F Lachnospiraceae	G Lachnospiraceae NK4A136 group	unclassified G Lachnospiraceae NK4A136 group
SV2038	K Bacteria	P Firmicutes	C Clostridia	O Lachnospirales	F Lachnospiraceae	G ASF356	unclassified G ASF356
SV2039	K Bacteria	P Firmicutes	C Clostridia	O Lachnospirales	F Lachnospiraceae	unclassified F Lachnospiraceae	unclassified F Lachnospiraceae
SV2040	K Bacteria	P Firmicutes	C Clostridia	O Lachnospirales	F Lachnospiraceae	unclassified F Lachnospiraceae	unclassified F Lachnospiraceae
SV2041	K Bacteria	P Firmicutes	C Clostridia	O Clostridia UCG-014	unclassified O Clostridia UCG-014	unclassified O Clostridia UCG-014	unclassified O Clostridia UCG-014
SV2042	K_Bacteria	P_Firmicutes	C_Clostridia	O_Oscillospirales	F_Ruminococcaceae	G_Paludicola	unclassified_G_Paludicola
SV2043	K Bacteria	P Firmicutes	C Clostridia	O Oscillospirales	F Ruminococcaceae	G Negativibacillus	unclassified G Negativibacillus
SV2044	K Bacteria	P Firmicutes	C Clostridia	O Lachnospirales	F Lachnospiraceae	unclassified F Lachnospiraceae	unclassified F Lachnospiraceae
SV2045	K Bacteria	P Firmicutes	C Clostridia	O Lachnospirales	F Lachnospiraceae	G Lachnospiraceae UCG-001	unclassified G Lachnospiraceae UCG-001
SV2046	K Bacteria	P Firmicutes	C Clostridia	O Lachnospirales	F Lachnospiraceae	unclassified F Lachnospiraceae	unclassified F Lachnospiraceae
SV2047	K Bacteria	P Bacteroidota	C Bacteroidia	O Bacteroidales	F Muribaculaceae	unclassified F Muribaculaceae	unclassified F Muribaculaceae
SV2048	K_Bacteria	P_Firmicutes	C_Clostridia	O_Oscillospirales	F_Oscillospiraceae	G_Colidextribacter	unclassified_G_Colidextribacter
SV2049	K Bacteria	P Firmicutes	C Clostridia	O Lachnospirales	F Lachnospiraceae	G Lachnospiraceae NK4A136 group	unclassified G Lachnospiraceae NK4A136 group
SV2050	K Bacteria	P Firmicutes	C Clostridia	O Oscillospirales	F Ruminococcaceae	unclassified F Ruminococcaceae	unclassified F Ruminococcaceae
SV2051	K Bacteria	P Firmicutes	C Clostridia	O Lachnospirales	F Lachnospiraceae	unclassified F Lachnospiraceae	unclassified F Lachnospiraceae
SV2052	K Bacteria	P Firmicutes	C Clostridia	O Oscillospirales	F Oscillospiraceae	G Intestinimonas	unclassified G Intestinimonas
SV2053	K Bacteria	P Firmicutes	C Clostridia	O Lachnospirales	F Lachnospiraceae	unclassified F Lachnospiraceae	unclassified F Lachnospiraceae
SV2054	K_Bacteria	P_Firmicutes	C_Clostridia	O_Clostridia UCG-014	unclassified_O_Clostridia UCG-014	unclassified_O_Clostridia UCG-014	unclassified_O_Clostridia UCG-014
SV2055	K Bacteria	P Bacteroidota	C Bacteroidia	O Bacteroidales	F Muribaculaceae	unclassified F Muribaculaceae	unclassified F Muribaculaceae
SV2056	K Bacteria	P Firmicutes	C Clostridia	O Lachnospirales	F Lachnospiraceae	G Marvinbryantia	unclassified G Marvinbryantia
SV2057	K Bacteria	P Firmicutes	C Clostridia	O Oscillospirales	F Oscillospiraceae	unclassified F Oscillospiraceae	unclassified F Oscillospiraceae
SV2058	K Bacteria	P Bacteroidota	C Bacteroidia	O Bacteroidales	F Muribaculaceae	unclassified F Muribaculaceae	unclassified F Muribaculaceae
SV2059	K Bacteria	P Firmicutes	C Clostridia	O Oscillospirales	F Oscillospiraceae	G Oscillibacter	unclassified G Oscillibacter
SV2060	K_Bacteria	P_Bacteroidota	C_Bacteroidia	O_Bacteroidales	F_Muribaculaceae	unclassified_F_Muribaculaceae	unclassified_F_Muribaculaceae
SV2061	K Bacteria	P Firmicutes	C Clostridia	O Lachnospirales	F Lachnospiraceae	G Lachnospiraceae UCG-001	unclassified G Lachnospiraceae UCG-001
SV2062	K Bacteria	P Firmicutes	C Clostridia	O Lachnospirales	F Lachnospiraceae	G A2	unclassified G A2
SV2063	K Bacteria	P Firmicutes	C Clostridia	O Lachnospirales	F Lachnospiraceae	G Marvinbryantia	unclassified G Marvinbryantia
SV2064	K Bacteria	P Bacteroidota	C Bacteroidia	O Bacteroidales	F Prevotellaceae	G Prevotellaceae UCG-001	unclassified G Prevotellaceae UCG-001
SV2065	K Bacteria	P Verrucomicrobiota	C Verrucomicrobiae	O Verrucomicrobiales	F Akkermansiaceae	G Akkermansia	unclassified G Akkermansia
SV2066	K Bacteria	P Firmicutes	C Clostridia	O Lachnospirales	F Lachnospiraceae	unclassified F Lachnospiraceae	unclassified F Lachnospiraceae
SV2067	K Bacteria	P Firmicutes	C Clostridia	O Lachnospirales	F Lachnospiraceae	G A2	unclassified G A2
SV2068	K Bacteria	P Firmicutes	C Clostridia	O Lachnospirales	F Lachnospiraceae	unclassified F Lachnospiraceae	unclassified F Lachnospiraceae

SV2069	K Bacteria	P Firmicutes	C Clostridia	O Oscillospirales	F Ruminococcaceae	unclassified F Ruminococcaceae	unclassified F Ruminococcaceae
SV2070	K Bacteria	P Firmicutes	C Clostridia	O Lachnospirales	F Lachnospiraceae	G Lachnospiraceae NK4A136 group	unclassified G Lachnospiraceae NK4A136 group
SV2071	K Bacteria	P Bacteroidota	C Bacteroidia	O Bacteroidales	F Bacteroidaceae	G Bacteroides	unclassified G Bacteroides
SV2072	K Bacteria	P Firmicutes	C Bacilli	O Lactobacillales	F Lactobacillaceae	G HT002	unclassified G HT002
SV2073	K Bacteria	P Bacteroidota	C Bacteroidia	O Bacteroidales	F Prevotellaceae	G Prevotellaceae UCG-001	unclassified G Prevotellaceae UCG-001
SV2074	K Bacteria	P Firmicutes	C Clostridia	O Lachnospirales	F Lachnospiraceae	G Roseburia	unclassified G Roseburia
SV2075	K Bacteria	P Firmicutes	C Clostridia	O Lachnospirales	F Lachnospiraceae	G GCA-900066575	unclassified G GCA-900066575
SV2076	K Bacteria	P Firmicutes	C Clostridia	O Lachnospirales	F Lachnospiraceae	G Lachnospiraceae UCG-001	unclassified G Lachnospiraceae UCG-001
SV2077	K Bacteria	P Bacteroidota	C Bacteroidia	O Bacteroidales	F Bacteroidaceae	G Bacteroides	unclassified G Bacteroides
SV2078	K Bacteria	P Firmicutes	C Clostridia	O Lachnospirales	F Lachnospiraceae	G Roseburia	unclassified G Roseburia
SV2079	K Bacteria	P Firmicutes	C Clostridia	O Lachnospirales	F Lachnospiraceae	G Eisenbergiella	S massiliensis
SV2080	K Bacteria	P Firmicutes	C Clostridia	O Oscillospirales	F Ruminococcaceae	G Incertae Sedis	unclassified G Incertae Sedis
SV2081	K Bacteria	P Firmicutes	C Bacilli	O Lactobacillales	F Lactobacillaceae	unclassified F Lactobacillaceae	unclassified F Lactobacillaceae
SV2082	K Bacteria	P Firmicutes	C Clostridia	O Lachnospirales	F Lachnospiraceae	G GCA-900066575	unclassified G GCA-900066575
SV2083	K Bacteria	P Firmicutes	C Clostridia	O Lachnospirales	F Lachnospiraceae	G Blautia	unclassified G Blautia
SV2084	K Bacteria	P Firmicutes	C Clostridia	O Lachnospirales	F Lachnospiraceae	G [Eubacterium] oxidoreducens group	unclassified G [Eubacterium] oxidoreducens group
SV2085	K Bacteria	P Firmicutes	C Clostridia	O Oscillospirales	F Oscillospiraceae	unclassified F Oscillospiraceae	unclassified F Oscillospiraceae
SV2086	K Bacteria	P Firmicutes	C Clostridia	O Oscillospirales	F Ruminococcaceae	G Ruminococcus	unclassified G Ruminococcus
SV2087	K Bacteria	P Firmicutes	C Clostridia	O Clostridia UCG-014	unclassified O Clostridia UCG-014	unclassified O Clostridia UCG-014	unclassified O Clostridia UCG-014
SV2088	K Bacteria	P Bacteroidota	C Bacteroidia	O Bacteroidales	F Muribaculaceae	unclassified F Muribaculaceae	unclassified F Muribaculaceae
SV2089	K Bacteria	P Firmicutes	C Clostridia	O Clostridia UCG-014	unclassified O Clostridia UCG-014	unclassified O Clostridia UCG-014	unclassified O Clostridia UCG-014
SV2090	K Bacteria	P Firmicutes	C Clostridia	O Lachnospirales	F Lachnospiraceae	G Dorea	unclassified G Dorea
SV2091	K Bacteria	P Firmicutes	C Clostridia	O Oscillospirales	F Ruminococcaceae	unclassified F Ruminococcaceae	unclassified F Ruminococcaceae
SV2092	K Bacteria	P Firmicutes	C Clostridia	O Lachnospirales	F Lachnospiraceae	unclassified F Lachnospiraceae	unclassified F Lachnospiraceae
SV2093	K Bacteria	P Firmicutes	C Clostridia	O Oscillospirales	F UCG-010	unclassified F UCG-010	unclassified F UCG-010
SV2094	K Bacteria	P Firmicutes	C Clostridia	unclassified C Clostridia	unclassified C Clostridia	unclassified C Clostridia	unclassified C Clostridia
SV2095	K Bacteria	P Firmicutes	C Clostridia	O Oscillospirales	F Ruminococcaceae	G Ruminococcus	unclassified G Ruminococcus
SV2096	K Bacteria	P Firmicutes	C Bacilli	O Lactobacillales	F Lactobacillaceae	unclassified F Lactobacillaceae	unclassified F Lactobacillaceae
SV2097	K Bacteria	P Bacteroidota	C Bacteroidia	O Bacteroidales	F Muribaculaceae	unclassified F Muribaculaceae	unclassified F Muribaculaceae
SV2098	K Bacteria	P Firmicutes	C Clostridia	O Eubacteriales	F Anaerofustaceae	G Anaerofustis	unclassified G Anaerofustis
SV2099	K Bacteria	P Firmicutes	C Clostridia	O Oscillospirales	F [Eubacterium] coprostanoligenes group	unclassified F [Eubacterium] coprostanoligenes group	unclassified F [Eubacterium] coprostanoligenes group
SV2100	K Bacteria	P Firmicutes	C Clostridia	O Lachnospirales	F Lachnospiraceae	G Lachnoclostridium	unclassified G Lachnoclostridium
SV2101	K Bacteria	P Firmicutes	C Clostridia	O Lachnospirales	F Lachnospiraceae	G Lachnospiraceae FCS020 group	unclassified G Lachnospiraceae FCS020 group
SV2102	K Bacteria	P Firmicutes	C Clostridia	O Lachnospirales	F Lachnospiraceae	unclassified F Lachnospiraceae	unclassified F Lachnospiraceae
SV2103	K Bacteria	P Firmicutes	C Clostridia	O Lachnospirales	F Lachnospiraceae	G Marvinbryantia	unclassified G Marvinbryantia
SV2104	K Bacteria	P Firmicutes	C Clostridia	O Christensenellales	F Christensenellaceae	G Christensenellaceae R-7 group	unclassified G Christensenellaceae R-7 group
SV2105	K Bacteria	P Firmicutes	C Bacilli	O Lactobacillales	F Lactobacillaceae	unclassified F Lactobacillaceae	unclassified F Lactobacillaceae
SV2106	K Bacteria	P Firmicutes	C Clostridia	O Lachnospirales	F Lachnospiraceae	G Lachnospiraceae NK4A136 group	unclassified G Lachnospiraceae NK4A136 group
SV2107	K Bacteria	P Firmicutes	C Clostridia	O Oscillospirales	F Butyricicoccaceae	G Butyricicoccus	unclassified G Butyricicoccus
SV2108	K Bacteria	P Firmicutes	C Bacilli	O Erysipelotrichales	F Erysipelotrichaceae	G Turicibacter	unclassified G Turicibacter
SV2109	K Bacteria	P Firmicutes	C Clostridia	O Oscillospirales	F Oscillospiraceae	G Colidextribacter	unclassified G Colidextribacter
SV2110	K Bacteria	P Firmicutes	C Clostridia	O Oscillospirales	F Ruminococcaceae	G Incertae Sedis	unclassified G Incertae Sedis
SV2111	K Bacteria	P Firmicutes	C Clostridia	O Peptostreptococcales-Tissierellales	F Anaerovoracaceae	G [Eubacterium] nodatum group	unclassified G [Eubacterium] nodatum group
SV2112	K Bacteria	P Firmicutes	C Clostridia	O Lachnospirales	F Lachnospiraceae	G Lachnospiraceae NK4A136 group	unclassified G Lachnospiraceae NK4A136 group
SV2113	K Bacteria	P Firmicutes	C Clostridia	O Lachnospirales	F Lachnospiraceae	unclassified F Lachnospiraceae	unclassified F Lachnospiraceae
SV2114	K Bacteria	P Firmicutes	C Clostridia	O Lachnospirales	F Lachnospiraceae	G [Eubacterium] oxidoreducens group	unclassified G [Eubacterium] oxidoreducens group
SV2115	K_Bacteria	P_Firmicutes	C_Clostridia	O_Christensenellales	F_Christensenellaceae	G_Christensenellaceae R-7 group	unclassified_G_Christensenellaceae R-7 group
SV2116	K Bacteria	P Firmicutes	C Clostridia	O Lachnospirales	F Lachnospiraceae	G Lachnospiraceae FCS020 group	unclassified G Lachnospiraceae FCS020 group
SV2117	K Bacteria	P Bacteroidota	C Bacteroidia	O Bacteroidales	F Bacteroidaceae	G Bacteroides	unclassified G Bacteroides
SV2118	K Bacteria	P Firmicutes	C Clostridia	O Clostridia UCG-014	unclassified O Clostridia UCG-014	unclassified O Clostridia UCG-014	unclassified O Clostridia UCG-014
SV2119	K Bacteria	P Firmicutes	C Clostridia	O Lachnospirales	F Lachnospiraceae	G Lachnospiraceae UCG-006	unclassified G Lachnospiraceae UCG-006
SV2120	K Bacteria	P Firmicutes	C Clostridia	O Lachnospirales	F Lachnospiraceae	G Lachnospiraceae UCG-001	unclassified G Lachnospiraceae UCG-001
SV2121	K_Bacteria	P_Firmicutes	C_Clostridia	O_Lachnospirales	F_Lachnospiraceae	G_Lachnospiraceae UCG-001	unclassified_G_Lachnospiraceae UCG-001
SV2122	K Bacteria	P Firmicutes	C Clostridia	O Peptostreptococcales-Tissierellales	F Anaerovoracaceae	G Family XIII AD3011 group	unclassified G Family XIII AD3011 group
SV2123	K Bacteria	P Actinobacteriota	C Coriobacteriia	O Coriobacteriales	F Eggerthellaceae	G Enterorhabdus	unclassified G Enterorhabdus
SV2124	K Bacteria	P Firmicutes	C Clostridia	O Clostridia vadinBB60 group	unclassified O Clostridia vadinBB60 group	unclassified O Clostridia vadinBB60 group	unclassified O Clostridia vadinBB60 group
SV2125	K Bacteria	P Firmicutes	C Clostridia	O Oscillospirales	F Butyricicoccaceae	G Butyricicoccus	unclassified G Butyricicoccus
SV2126	K Bacteria	P Firmicutes	C Clostridia	O Lachnospirales	F Lachnospiraceae	unclassified F Lachnospiraceae	unclassified F Lachnospiraceae
SV2127	K_Bacteria	P_Firmicutes	C_Clostridia	O_Oscillospirales	F_Oscillospiraceae	unclassified_F_Oscillospiraceae	unclassified_F_Oscillospiraceae
SV2128	K Bacteria	P Firmicutes	C Clostridia	O Oscillospirales	F Oscillospiraceae	unclassified F Oscillospiraceae	unclassified F Oscillospiraceae
SV2129	K Bacteria	P Firmicutes	C Clostridia	O Lachnospirales	F Lachnospiraceae	G Murimonas	S intestini
SV2130	K Bacteria	P Firmicutes	C Clostridia	O Clostridia UCG-014	unclassified O Clostridia UCG-014	unclassified O Clostridia UCG-014	unclassified O Clostridia UCG-014
SV2131	K Bacteria	P Firmicutes	C Clostridia	O Oscillospirales	F Oscillospiraceae	G Colidextribacter	unclassified G Colidextribacter
SV2132	K Bacteria	P Firmicutes	C Clostridia	O Lachnospirales	F Lachnospiraceae	G Lachnospiraceae NK4A136 group	unclassified G Lachnospiraceae NK4A136 group
SV2133	K_Bacteria	P_Firmicutes	C_Clostridia	O_Oscillospirales	F_Ruminococcaceae	G_Incertae Sedis	unclassified_G_Incertae Sedis
SV2134	K Bacteria	P Firmicutes	C Clostridia	O Lachnospirales	F Lachnospiraceae	G Lachnoclostridium	unclassified G Lachnoclostridium
SV2135	K Bacteria	P Firmicutes	C Clostridia	O Lachnospirales	F Lachnospiraceae	unclassified F Lachnospiraceae	unclassified F Lachnospiraceae
SV2136	K Bacteria	P Firmicutes	C Clostridia	O Oscillospirales	F Ruminococcaceae	unclassified F Ruminococcaceae	unclassified F Ruminococcaceae
SV2137	K Bacteria	P Bacteroidota	C Bacteroidia	O Bacteroidales	F Marinifilaceae	G Odoribacter	unclassified G Odoribacter
SV2138	K Bacteria	P Firmicutes	C Clostridia	O Lachnospirales	F Lachnospiraceae	G Lachnospiraceae FCS020 group	unclassified G Lachnospiraceae FCS020 group
SV2139	K_Bacteria	P_Firmicutes	C_Clostridia	O_Lachnospirales	F_Lachnospiraceae	G_Lachnoclostridium	unclassified_G_Lachnoclostridium
SV2140	K Bacteria	P Firmicutes	C Clostridia	O Clostridia UCG-014	unclassified O Clostridia UCG-014	unclassified O Clostridia UCG-014	unclassified O Clostridia UCG-014
SV2141	K Bacteria	P Firmicutes	C Clostridia	O Lachnospirales	F Lachnospiraceae	G A2	unclassified G A2
SV2142	K Bacteria	P Bacteroidota	C Bacteroidia	O Bacteroidales	F Muribaculaceae	unclassified F Muribaculaceae	unclassified F Muribaculaceae
SV2143	K Bacteria	P Bacteroidota	C Bacteroidia	O Bacteroidales	F Muribaculaceae	unclassified F Muribaculaceae	unclassified F Muribaculaceae
SV2144	K Bacteria	P Firmicutes	C Clostridia	O Lachnospirales	F Lachnospiraceae	G Lachnospiraceae NK4A136 group	unclassified G Lachnospiraceae NK4A136 group
SV2145	K Bacteria	P Firmicutes	C Clostridia	O Oscillospirales	F Oscillospiraceae	unclassified F Oscillospiraceae	unclassified F Oscillospiraceae
SV2146	K Bacteria	P Bacteroidota	C Bacteroidia	O Bacteroidales	F Muribaculaceae	unclassified F Muribaculaceae	unclassified F Muribaculaceae
SV2147	K Bacteria	P Firmicutes	C Clostridia	O Oscillospirales	F Oscillospiraceae	G Oscillibacter	unclassified G Oscillibacter
SV2148	K Bacteria	P Firmicutes	C Clostridia	O Oscillospirales	F Ruminococcaceae	unclassified F Ruminococcaceae	unclassified F Ruminococcaceae
SV2149	K Bacteria	P Firmicutes	C Clostridia	O Clostridia vadinBB60 group	unclassified O Clostridia vadinBB60 group	unclassified O Clostridia vadinBB60 group	unclassified O Clostridia vadinBB60 group
SV2150	K Bacteria	P Firmicutes	C Clostridia	O Lachnospirales	F Lachnospiraceae	unclassified F Lachnospiraceae	unclassified F Lachnospiraceae
SV2151	K Bacteria	P Firmicutes	C Clostridia	O Oscillospirales	F Ruminococcaceae	G Candidatus Soleaferrea	unclassified G Candidatus Soleaferrea
SV2152	K Bacteria	P Firmicutes	C Clostridia	O Lachnospirales	F Lachnospiraceae	G Frisingicoccus	S caecimuris
SV2153	K Bacteria	P Firmicutes	C Clostridia	O Lachnospirales	F Lachnospiraceae	G Lachnospiraceae FCS020 group	unclassified G Lachnospiraceae FCS020 group
SV2154	K Bacteria	P Firmicutes	C Clostridia	O Lachnospirales	F Lachnospiraceae	G Hungatella	S hathewayi
SV2155	K Bacteria	P Firmicutes	C Clostridia	O Lachnospirales	F Lachnospiraceae	unclassified F Lachnospiraceae	unclassified F Lachnospiraceae
SV2156	K Bacteria	P Firmicutes	C Bacilli	O RF39	unclassified O RF39	unclassified O RF39	unclassified O RF39
SV2157	K Bacteria	P Firmicutes	C Clostridia	O Lachnospirales	F Lachnospiraceae	G ASF356	unclassified G ASF356
SV2158	K Bacteria	P Firmicutes	C Clostridia	O Oscillospirales	F [Eubacterium] coprostanoligenes group	unclassified F [Eubacterium] coprostanoligenes group	unclassified F [Eubacterium] coprostanoligenes group
SV2159	K Bacteria	P Firmicutes	C Clostridia	O Lachnospirales	F Lachnospiraceae	unclassified F Lachnospiraceae	unclassified F Lachnospiraceae
SV2160	K Bacteria	P Firmicutes	C Clostridia	O Lachnospirales	F Lachnospiraceae	unclassified F Lachnospiraceae	unclassified F Lachnospiraceae
SV2161	K Bacteria	P Firmicutes	C Clostridia	O Lachnospirales	F Lachnospiraceae	G Acetitomaculum	unclassified G Acetitomaculum
SV2162	K Bacteria	P Firmicutes	C Clostridia	O Clostridia UCG-014	unclassified O Clostridia UCG-014	unclassified O Clostridia UCG-014	unclassified O Clostridia UCG-014
SV2163	K Bacteria	P Firmicutes	C Clostridia	O Lachnospirales	F Lachnospiraceae	G Marvinbryantia	unclassified G Marvinbryantia
SV2164	K Bacteria	P Firmicutes	C Clostridia	O Clostridia UCG-014	unclassified O Clostridia UCG-014	unclassified O Clostridia UCG-014	unclassified O Clostridia UCG-014
SV2165	K Bacteria	P Bacteroidota	C Bacteroidia	O Bacteroidales	F Marinifilaceae	G Odoribacter	unclassified G Odoribacter
SV2166	K Bacteria	P Actinobacteriota	C Coriobacteriia	O Coriobacteriales	F Eggerthellaceae	G Enterorhabdus	unclassified G Enterorhabdus
SV2167	K Bacteria	P Firmicutes	C Clostridia	O Oscillospirales	F Oscillospiraceae	unclassified F Oscillospiraceae	unclassified F Oscillospiraceae
SV2168	K Bacteria	P Verrucomicrobiota	C Verrucomicrobiae	O Verrucomicrobiales	F Akkermansiaceae	G Akkermansia	unclassified G Akkermansia
SV2169	K Bacteria	P Firmicutes	C Clostridia	O Lachnospirales	F Lachnospiraceae	unclassified F Lachnospiraceae	unclassified F Lachnospiraceae
SV2170	K Bacteria	P Firmicutes	C Clostridia	O Clostridia UCG-014	unclassified O Clostridia UCG-014	unclassified O Clostridia UCG-014	unclassified O Clostridia UCG-014
SV2171	K Bacteria	P Firmicutes	C Clostridia	O Clostridia vadinBB60 group	unclassified O Clostridia vadinBB60 group	unclassified O Clostridia vadinBB60 group	unclassified O Clostridia vadinBB60 group
SV2172	K Bacteria	P Bacteroidota	C Bacteroidia	O Bacteroidales	F Muribaculaceae	unclassified F Muribaculaceae	unclassified F Muribaculaceae
SV2173	K Bacteria	P Bacteroidota	C Bacteroidia	O Bacteroidales	F Muribaculaceae	unclassified F Muribaculaceae	unclassified F Muribaculaceae
SV2174	K Bacteria	P Actinobacteriota	C Coriobacteriia	O Coriobacteriales	F Eggerthellaceae	G Enterorhabdus	unclassified G Enterorhabdus
SV2175	K Bacteria	P Firmicutes	C Clostridia	O Oscillospirales	F Ruminococcaceae	unclassified F Ruminococcaceae	unclassified F Ruminococcaceae
SV2176	K Bacteria	P Firmicutes	C Clostridia	O Clostridia UCG-014	unclassified O Clostridia UCG-014	unclassified O Clostridia UCG-014	unclassified O Clostridia UCG-014
SV2177	K Bacteria	P Firmicutes	C Clostridia	O Oscillospirales	F Ruminococcaceae	G Incertae Sedis	unclassified G Incertae Sedis
SV2178	K Bacteria	P Firmicutes	C Clostridia	O Lachnospirales	F Lachnospiraceae	unclassified F Lachnospiraceae	unclassified F Lachnospiraceae
SV2179	K Bacteria	P Firmicutes	C Clostridia	O Clostridia vadinBB60 group	unclassified O Clostridia vadinBB60 group	unclassified O Clostridia vadinBB60 group	unclassified O Clostridia vadinBB60 group
SV2180	K_Bacteria	P_Firmicutes	C_Clostridia	O_Clostridia vadinBB60 group	unclassified_O_Clostridia vadinBB60 group	unclassified_O_Clostridia vadinBB60 group	unclassified_O_Clostridia vadinBB60 group
SV2181	K Bacteria	P Firmicutes	C Clostridia	O Lachnospirales	F Lachnospiraceae	G Lachnospiraceae NK4A136 group	unclassified G Lachnospiraceae NK4A136 group
SV2182	K Bacteria	P Firmicutes	C Clostridia	O Oscillospirales	F Oscillospiraceae	G Colidextribacter	unclassified G Colidextribacter
SV2183	K Bacteria	P Firmicutes	C Clostridia	O Lachnospirales	F Lachnospiraceae	unclassified F Lachnospiraceae	unclassified F Lachnospiraceae
SV2184	K Bacteria	P Firmicutes	C Clostridia	O Clostridia UCG-014	unclassified O Clostridia UCG-014	unclassified O Clostridia UCG-014	unclassified O Clostridia UCG-014
SV2185	K Bacteria	P Bacteroidota	C Bacteroidia	O Bacteroidales	F Muribaculaceae	unclassified F Muribaculaceae	unclassified F Muribaculaceae
SV2186	K_Bacteria	P_Firmicutes	C_Clostridia	O_Lachnospirales	F_Lachnospiraceae	unclassified_F_Lachnospiraceae	unclassified_F_Lachnospiraceae
SV2187	K Bacteria	P Firmicutes	C Clostridia	O Oscillospirales	F Ruminococcaceae	unclassified F Ruminococcaceae	unclassified F Ruminococcaceae
SV2188	K Bacteria	P Firmicutes	C Clostridia	O Oscillospirales	F Ruminococcaceae	G UBA1819	unclassified G UBA1819
SV2189	K Bacteria	P Firmicutes	C Clostridia	O Lachnospirales	F Lachnospiraceae	G GCA-900066575	unclassified G GCA-900066575
SV2190	K Bacteria	P Firmicutes	C Bacilli	O Lactobacillales	F Lactobacillaceae	unclassified F Lactobacillaceae	unclassified F Lactobacillaceae
SV2191	K Bacteria	P Firmicutes	C Clostridia	O Lachnospirales	F Lachnospiraceae	unclassified F Lachnospiraceae	unclassified F Lachnospiraceae
SV2192	K_Bacteria	P_Firmicutes	C_Clostridia	O_Lachnospirales	F_Lachnospiraceae	G_Blautia	unclassified_G_Blautia
SV2193	K Bacteria	P Firmicutes	C Clostridia	O Oscillospirales	F Ruminococcaceae	unclassified F Ruminococcaceae	unclassified F Ruminococcaceae
SV2194	K Bacteria	P Firmicutes	C Clostridia	O Peptostreptococcales-Tissierellales	F Peptostreptococcaceae	unclassified F Peptostreptococcaceae	unclassified F Peptostreptococcaceae
SV2195	K Bacteria	P Firmicutes	C Clostridia	O Lachnospirales	F Lachnospiraceae	G Lachnoclostridium	unclassified G Lachnoclostridium
SV2196	K Bacteria	P Firmicutes	C Clostridia	O Oscillospirales	F Ruminococcaceae	unclassified F Ruminococcaceae	unclassified F Ruminococcaceae
SV2197	K Bacteria	P Firmicutes	C Clostridia	O Lachnospirales	F Lachnospiraceae	unclassified F Lachnospiraceae	unclassified F Lachnospiraceae
SV2198	K_Bacteria	P_Firmicutes	C_Clostridia	O_Oscillospirales	F_Ruminococcaceae	unclassified_F_Ruminococcaceae	unclassified_F_Ruminococcaceae
SV2199	K Bacteria	P Firmicutes	C Clostridia	O Clostridia UCG-014	unclassified O Clostridia UCG-014	unclassified O Clostridia UCG-014	unclassified O Clostridia UCG-014
SV2200	K Bacteria	P Firmicutes	C Clostridia	O Oscillospirales	F Ruminococcaceae	G Incertae Sedis	unclassified G Incertae Sedis
SV2201	K Bacteria	P Firmicutes	C Clostridia	O Lachnospirales	F Lachnospiraceae	G Acetatifactor	S muris
SV2202	K Bacteria	P Firmicutes	C Clostridia	O Oscillospirales	F Oscillospiraceae	unclassified F Oscillospiraceae	unclassified F Oscillospiraceae
SV2203	K Bacteria	P Firmicutes	C Clostridia	O Lachnospirales	F Lachnospiraceae	G Roseburia	unclassified G Roseburia
SV2204	K Bacteria	P Firmicutes	C Clostridia	O Lachnospirales	F Lachnospiraceae	G Lachnospiraceae UCG-001	unclassified G Lachnospiraceae UCG-001
SV2205	K Bacteria	P Firmicutes	C Clostridia	O Oscillospirales	F UCG-010	unclassified F UCG-010	unclassified F UCG-010
SV2206	K Bacteria	P Firmicutes	C Clostridia	O Oscillospirales	F Ruminococcaceae	G Anaerotruncus	unclassified G Anaerotruncus

SV2207	K Bacteria	P Firmicutes	C Clostridia	O Lachnospirales	F Lachnospiraceae	G Lachnoclostridium	unclassified G Lachnoclostridium
SV2208	K Bacteria	P Firmicutes	C Clostridia	O Clostridia UCG-014	unclassified O Clostridia UCG-014	unclassified O Clostridia UCG-014	unclassified O Clostridia UCG-014
SV2209	K Bacteria	P Firmicutes	C Clostridia	O Oscillospirales	F Oscillospiraceae	unclassified F Oscillospiraceae	unclassified F Oscillospiraceae
SV2210	K Bacteria	P Firmicutes	C Clostridia	O Oscillospirales	F Ruminococcaceae	unclassified F Ruminococcaceae	unclassified F Ruminococcaceae
SV2211	K Bacteria	P Bacteroidota	C Bacteroidia	O Bacteroidales	F Bacteroidaceae	G Bacteroides	unclassified G Bacteroides
SV2212	K Bacteria	P Firmicutes	C Clostridia	O Clostridia UCG-014	unclassified O Clostridia UCG-014	unclassified O Clostridia UCG-014	unclassified O Clostridia UCG-014
SV2213	K Bacteria	P Firmicutes	C Clostridia	O Lachnospirales	F Lachnospiraceae	unclassified F Lachnospiraceae	unclassified F Lachnospiraceae
SV2214	K Bacteria	P Bacteroidota	C Bacteroidia	O Bacteroidales	F Muribaculaceae	unclassified F Muribaculaceae	unclassified F Muribaculaceae
SV2215	K Bacteria	P Firmicutes	C Clostridia	O Oscillospirales	F Ruminococcaceae	G Ruminococcus	unclassified G Ruminococcus
SV2216	K Bacteria	P Firmicutes	C Clostridia	O Lachnospirales	F Lachnospiraceae	G GCA-900066575	unclassified G GCA-900066575
SV2217	K Bacteria	P Firmicutes	C Clostridia	O Oscillospirales	F Ruminococcaceae	unclassified F Ruminococcaceae	unclassified F Ruminococcaceae
SV2218	K Bacteria	P Firmicutes	C Clostridia	O Lachnospirales	F Lachnospiraceae	unclassified F Lachnospiraceae	unclassified F Lachnospiraceae
SV2219	K Bacteria	P Firmicutes	C Clostridia	O Clostridia vadinBB60 group	unclassified O Clostridia vadinBB60 group	unclassified O Clostridia vadinBB60 group	unclassified O Clostridia vadinBB60 group
SV2220	K Bacteria	P Firmicutes	C Clostridia	O Clostridia UCG-014	unclassified O Clostridia UCG-014	unclassified O Clostridia UCG-014	unclassified O Clostridia UCG-014
SV2221	K Bacteria	P Firmicutes	C Clostridia	O Oscillospirales	F Oscillospiraceae	G Colidextribacter	unclassified G Colidextribacter
SV2222	K Bacteria	P Firmicutes	C Clostridia	O Lachnospirales	F Lachnospiraceae	unclassified F Lachnospiraceae	unclassified F Lachnospiraceae
SV2223	K Bacteria	P Firmicutes	C Clostridia	O Lachnospirales	F Lachnospiraceae	G Lachnospiraceae NK4A136 group	unclassified G Lachnospiraceae NK4A136 group
SV2224	K Bacteria	P Bacteroidota	C Bacteroidia	O Bacteroidales	F Muribaculaceae	unclassified F Muribaculaceae	unclassified F Muribaculaceae
SV2225	K Bacteria	P Firmicutes	C Clostridia	O Oscillospirales	F Oscillospiraceae	G Intestinimonas	unclassified G Intestinimonas
SV2226	K Bacteria	P Firmicutes	C Clostridia	O Oscillospirales	F Ruminococcaceae	G Harryflintia	unclassified G Harryflintia
SV2227	K Bacteria	P Bacteroidota	C Bacteroidia	O Bacteroidales	F Rikenellaceae	G Rikenellaceae RC9 gut group	unclassified G Rikenellaceae RC9 gut group
SV2228	K Bacteria	P Firmicutes	C Clostridia	O Lachnospirales	F Lachnospiraceae	unclassified F Lachnospiraceae	unclassified F Lachnospiraceae
SV2229	K Bacteria	P Firmicutes	C Clostridia	O Clostridia vadinBB60 group	unclassified O Clostridia vadinBB60 group	unclassified O Clostridia vadinBB60 group	unclassified O Clostridia vadinBB60 group
SV2230	K Bacteria	P Firmicutes	C Clostridia	O Lachnospirales	F Lachnospiraceae	G Lachnospiraceae UCG-008	unclassified G Lachnospiraceae UCG-008
SV2231	K Bacteria	P Firmicutes	C Clostridia	O Oscillospirales	F Ruminococcaceae	unclassified F Ruminococcaceae	unclassified F Ruminococcaceae
SV2232	K Bacteria	P Bacteroidota	C Bacteroidia	O Bacteroidales	F Muribaculaceae	unclassified F Muribaculaceae	unclassified F Muribaculaceae
SV2233	K Bacteria	P Firmicutes	C Clostridia	O Clostridia UCG-014	unclassified O Clostridia UCG-014	unclassified O Clostridia UCG-014	unclassified O Clostridia UCG-014
SV2234	K Bacteria	P Actinobacteriota	C Coriobacteriia	O Coriobacteriales	F Eggerthellaceae	G Enterorhabdus	unclassified G Enterorhabdus
SV2235	K Bacteria	P Firmicutes	C Clostridia	O Clostridia vadinBB60 group	unclassified O Clostridia vadinBB60 group	unclassified O Clostridia vadinBB60 group	unclassified O Clostridia vadinBB60 group
SV2236	K Bacteria	P Bacteroidota	C Bacteroidia	O Bacteroidales	F Muribaculaceae	unclassified F Muribaculaceae	unclassified F Muribaculaceae
SV2237	K Bacteria	P Firmicutes	C Clostridia	O Oscillospirales	F Oscillospiraceae	unclassified F Oscillospiraceae	unclassified F Oscillospiraceae
SV2238	K Bacteria	P Firmicutes	C Bacilli	O RF39	unclassified O RF39	unclassified O RF39	unclassified O RF39
SV2239	K Bacteria	P Firmicutes	C Bacilli	O Erysipelotrichales	F Erysipelotrichaceae	G Turicibacter	unclassified G Turicibacter
SV2240	K Bacteria	P Bacteroidota	C Bacteroidia	O Bacteroidales	F Muribaculaceae	unclassified F Muribaculaceae	unclassified F Muribaculaceae
SV2241	K Bacteria	P Firmicutes	C Clostridia	O Oscillospirales	F Oscillospiraceae	unclassified F Oscillospiraceae	unclassified F Oscillospiraceae
SV2242	K Bacteria	P Firmicutes	C Clostridia	O Oscillospirales	F Ruminococcaceae	unclassified F Ruminococcaceae	unclassified F Ruminococcaceae
SV2243	K Bacteria	P Firmicutes	C Clostridia	O Oscillospirales	F Butyricicoccaceae	G Butyricicoccus	unclassified G Butyricicoccus
SV2244	K Bacteria	P Firmicutes	C Clostridia	O Lachnospirales	F Lachnospiraceae	G [Eubacterium] ventriosum group	unclassified G [Eubacterium] ventriosum group
SV2245	K Bacteria	P Firmicutes	C Clostridia	O Clostridia vadinBB60 group	unclassified O Clostridia vadinBB60 group	unclassified O Clostridia vadinBB60 group	unclassified O Clostridia vadinBB60 group
SV2246	K Bacteria	P Firmicutes	C Clostridia	O Clostridia UCG-014	unclassified O Clostridia UCG-014	unclassified O Clostridia UCG-014	unclassified O Clostridia UCG-014
SV2247	K Bacteria	P Firmicutes	C Clostridia	O Oscillospirales	F Butyricicoccaceae	G Butyricicoccus	unclassified G Butyricicoccus
SV2248	K Bacteria	P Firmicutes	C Clostridia	O Clostridia UCG-014	unclassified O Clostridia UCG-014	unclassified O Clostridia UCG-014	unclassified O Clostridia UCG-014
SV2249	K Bacteria	P Firmicutes	C Clostridia	O Peptococcales	F Peptococcaceae	unclassified F Peptococcaceae	unclassified F Peptococcaceae
SV2250	K Bacteria	P Firmicutes	C Clostridia	O Lachnospirales	F Lachnospiraceae	G Lachnospiraceae NK4A136 group	unclassified G Lachnospiraceae NK4A136 group
SV2251	K Bacteria	P Firmicutes	C Clostridia	O Lachnospirales	F Lachnospiraceae	unclassified F Lachnospiraceae	unclassified F Lachnospiraceae
SV2252	K Bacteria	P Firmicutes	C Clostridia	O Oscillospirales	F [Eubacterium] coprostanoligenes group	unclassified F [Eubacterium] coprostanoligenes group	unclassified F [Eubacterium] coprostanoligenes group
SV2253	K_Bacteria	P_Firmicutes	C_Clostridia	O_Lachnospirales	F_Lachnospiraceae	unclassified_F_Lachnospiraceae	unclassified_F_Lachnospiraceae
SV2254	K Bacteria	P Firmicutes	C Clostridia	O Lachnospirales	F Lachnospiraceae	G [Eubacterium] oxidoreducens group	unclassified G [Eubacterium] oxidoreducens group
SV2255	K Bacteria	P Firmicutes	C Clostridia	O Oscillospirales	F Oscillospiraceae	G Colidextribacter	unclassified G Colidextribacter
SV2256	K Bacteria	P Firmicutes	C Clostridia	O Oscillospirales	F Oscillospiraceae	G Colidextribacter	unclassified G Colidextribacter
SV2257	K Bacteria	P Firmicutes	C Clostridia	O Peptostreptococcales-Tissierellales	F Anaerovoracaceae	G Family XIII UCG-001	unclassified G Family XIII UCG-001
SV2258	K Bacteria	P Firmicutes	C Clostridia	O Oscillospirales	F Oscillospiraceae	unclassified F Oscillospiraceae	unclassified F Oscillospiraceae
SV2259	K_Bacteria	P_Firmicutes	C_Clostridia	O_Lachnospirales	F_Lachnospiraceae	G_Tuzzerella	unclassified_G_Tuzzerella
SV2260	K Bacteria	P Firmicutes	C Clostridia	O Peptostreptococcales-Tissierellales	F Anaerovoracaceae	G [Eubacterium] nodatum group	unclassified G [Eubacterium] nodatum group
SV2261	K Bacteria	P Actinobacteriota	C Actinobacteria	O Bifidobacteriales	F Bifidobacteriaceae	G Bifidobacterium	S bifidum
SV2262	K Bacteria	P Firmicutes	C Clostridia	O Christensenellales	F Christensenellaceae	G Christensenellaceae R-7 group	unclassified G Christensenellaceae R-7 group
SV2263	K Bacteria	P Firmicutes	C Bacilli	O Erysipelotrichales	F Erysipelotrichaceae	G Turicibacter	unclassified G Turicibacter
SV2264	K Bacteria	P Firmicutes	C Clostridia	O Oscillospirales	F Ruminococcaceae	unclassified F Ruminococcaceae	unclassified F Ruminococcaceae
SV2265	K_Bacteria	P_Firmicutes	C_Clostridia	O_Lachnospirales	F_Lachnospiraceae	unclassified_F_Lachnospiraceae	unclassified_F_Lachnospiraceae
SV2266	K Bacteria	P Firmicutes	C Clostridia	O Clostridiales	F Clostridiaceae	unclassified F Clostridiaceae	unclassified F Clostridiaceae
SV2267	K Bacteria	P Firmicutes	C Clostridia	O Peptostreptococcales-Tissierellales	F Peptostreptococcaceae	unclassified F Peptostreptococcaceae	unclassified F Peptostreptococcaceae
SV2268	K Bacteria	P Bacteroidota	C Bacteroidia	O Bacteroidales	F Rikenellaceae	G Alistipes	unclassified G Alistipes
SV2269	K Bacteria	P Firmicutes	C Clostridia	O Clostridia UCG-014	unclassified O Clostridia UCG-014	unclassified O Clostridia UCG-014	unclassified O Clostridia UCG-014
SV2270	K Bacteria	P Bacteroidota	C Bacteroidia	O Bacteroidales	F Rikenellaceae	G Rikenellaceae RC9 gut group	unclassified G Rikenellaceae RC9 gut group
SV2271	K_Bacteria	P_Firmicutes	C_Clostridia	O_Clostridia UCG-014	unclassified_O_Clostridia UCG-014	unclassified_O_Clostridia UCG-014	unclassified_O_Clostridia UCG-014
SV2272	K Bacteria	P Firmicutes	C Clostridia	O Oscillospirales	F Ruminococcaceae	unclassified F Ruminococcaceae	unclassified F Ruminococcaceae
SV2273	K Bacteria	P Bacteroidota	C Bacteroidia	O Bacteroidales	F Bacteroidaceae	G Bacteroides	unclassified G Bacteroides
SV2274	K Bacteria	P Firmicutes	C Clostridia	O Lachnospirales	F Lachnospiraceae	unclassified F Lachnospiraceae	unclassified F Lachnospiraceae
SV2275	K Bacteria	P Firmicutes	C Clostridia	O Peptostreptococcales-Tissierellales	F Anaerovoracaceae	G Family XIII UCG-001	unclassified G Family XIII UCG-001
SV2276	K Bacteria	P Firmicutes	C Clostridia	O Oscillospirales	F Oscillospiraceae	G Colidextribacter	unclassified G Colidextribacter
SV2277	K_Bacteria	P_Firmicutes	C_Clostridia	O_Lachnospirales	F_Lachnospiraceae	G_Lachnoclostridium	unclassified_G_Lachnoclostridium
SV2278	K Bacteria	P Firmicutes	C Clostridia	O Clostridia UCG-014	unclassified O Clostridia UCG-014	unclassified O Clostridia UCG-014	unclassified O Clostridia UCG-014
SV2279	K Bacteria	P Firmicutes	C Clostridia	O Lachnospirales	F Lachnospiraceae	G Roseburia	unclassified G Roseburia
SV2280	K Bacteria	P Firmicutes	C Clostridia	O Oscillospirales	F Ruminococcaceae	unclassified F Ruminococcaceae	unclassified F Ruminococcaceae
SV2281	K Bacteria	P Firmicutes	C Clostridia	O Lachnospirales	F Lachnospiraceae	G Lachnospiraceae UCG-006	unclassified G Lachnospiraceae UCG-006
SV2282	K Bacteria	P Firmicutes	C Clostridia	O Lachnospirales	F Lachnospiraceae	unclassified F Lachnospiraceae	unclassified F Lachnospiraceae
SV2283	K Bacteria	P Firmicutes	C Clostridia	O Clostridiales	F Clostridiaceae	G Clostridium sensu stricto 1	unclassified G Clostridium sensu stricto 1
SV2284	K Bacteria	P Firmicutes	C Clostridia	O Lachnospirales	F Lachnospiraceae	G Lachnoclostridium	unclassified G Lachnoclostridium
SV2285	K Bacteria	P Firmicutes	C Clostridia	O Lachnospirales	F Lachnospiraceae	G [Eubacterium] oxidoreducens group	unclassified G [Eubacterium] oxidoreducens group
SV2286	K Bacteria	P Firmicutes	C Clostridia	O Oscillospirales	F Oscillospiraceae	unclassified F Oscillospiraceae	unclassified F Oscillospiraceae
SV2287	K Bacteria	P Bacteroidota	C Bacteroidia	O Bacteroidales	F Rikenellaceae	G Alistipes	unclassified G Alistipes
SV2288	K Bacteria	P Firmicutes	C Bacilli	O Erysipelotrichales	F Erysipelotrichaceae	G Turicibacter	unclassified G Turicibacter
SV2289	K Bacteria	P Firmicutes	C Clostridia	O Lachnospirales	F Lachnospiraceae	G Lachnospiraceae NK4A136 group	unclassified G Lachnospiraceae NK4A136 group
SV2290	K Bacteria	P Firmicutes	C Clostridia	O Peptostreptococcales-Tissierellales	F Anaerovoracaceae	G Anaerovorax	unclassified G Anaerovorax
SV2291	K Bacteria	P Firmicutes	C Clostridia	O Oscillospirales	F Ruminococcaceae	unclassified F Ruminococcaceae	unclassified F Ruminococcaceae
SV2292	K Bacteria	P Firmicutes	C Clostridia	O Lachnospirales	F Lachnospiraceae	G Murimonas	unclassified G Murimonas
SV2293	K Bacteria	P Firmicutes	C Clostridia	O Lachnospirales	F Lachnospiraceae	G ASF356	unclassified G ASF356
SV2294	K Bacteria	P Firmicutes	C Clostridia	O Lachnospirales	F Lachnospiraceae	unclassified F Lachnospiraceae	unclassified F Lachnospiraceae
SV2295	K Bacteria	P Firmicutes	C Clostridia	O Clostridia UCG-014	unclassified O Clostridia UCG-014	unclassified O Clostridia UCG-014	unclassified O Clostridia UCG-014
SV2296	K Bacteria	P Firmicutes	C Clostridia	O Clostridia UCG-014	unclassified O Clostridia UCG-014	unclassified O Clostridia UCG-014	unclassified O Clostridia UCG-014
SV2297	K Bacteria	P Firmicutes	C Clostridia	O Clostridia UCG-014	unclassified O Clostridia UCG-014	unclassified O Clostridia UCG-014	unclassified O Clostridia UCG-014
SV2298	K Bacteria	P Firmicutes	C Clostridia	O Oscillospirales	F Oscillospiraceae	G Oscillibacter	unclassified G Oscillibacter
SV2299	K Bacteria	P Firmicutes	C Clostridia	O Oscillospirales	F Butyricicoccaceae	G Butyricicoccus	unclassified G Butyricicoccus
SV2300	K Bacteria	P Firmicutes	C Clostridia	O Lachnospirales	F Lachnospiraceae	G Lachnospiraceae UCG-008	unclassified G Lachnospiraceae UCG-008
SV2301	K Bacteria	P Firmicutes	C Clostridia	O Lachnospirales	F Lachnospiraceae	G Acetatifactor	S muris
SV2302	K Bacteria	P Firmicutes	C Clostridia	O Oscillospirales	F Ruminococcaceae	unclassified F Ruminococcaceae	unclassified F Ruminococcaceae
SV2303	K Bacteria	P Firmicutes	C Bacilli	O Lactobacillales	F Lactobacillaceae	G Ligilactobacillus	unclassified G Ligilactobacillus
SV2304	K Bacteria	P Firmicutes	C Clostridia	O Lachnospirales	F Lachnospiraceae	unclassified F Lachnospiraceae	unclassified F Lachnospiraceae
SV2305	K Bacteria	P Firmicutes	C Clostridia	O Oscillospirales	F UCG-010	unclassified F UCG-010	unclassified F UCG-010
SV2306	K Bacteria	P Firmicutes	C Bacilli	unclassified C Bacilli	unclassified C Bacilli	unclassified C Bacilli	unclassified C Bacilli
SV2307	K Bacteria	P Firmicutes	C Clostridia	O Lachnospirales	F Lachnospiraceae	G Marvinbryantia	unclassified G Marvinbryantia
SV2308	K Bacteria	P Firmicutes	C Clostridia	O Lachnospirales	F Lachnospiraceae	G Lachnospiraceae UCG-006	unclassified G Lachnospiraceae UCG-006
SV2309	K Bacteria	P Firmicutes	C Clostridia	O Lachnospirales	F Lachnospiraceae	unclassified F Lachnospiraceae	unclassified F Lachnospiraceae
SV2310	K Bacteria	P Bacteroidota	C Bacteroidia	O Bacteroidales	F Muribaculaceae	unclassified F Muribaculaceae	unclassified F Muribaculaceae
SV2311	K Bacteria	P Firmicutes	C Clostridia	O Lachnospirales	F Lachnospiraceae	G Roseburia	unclassified G Roseburia
SV2312	K Bacteria	P Firmicutes	C Clostridia	O Oscillospirales	F Ruminococcaceae	unclassified F Ruminococcaceae	unclassified F Ruminococcaceae
SV2313	K Bacteria	P Firmicutes	C Clostridia	O Oscillospirales	F Oscillospiraceae	G Colidextribacter	unclassified G Colidextribacter
SV2314	K Bacteria	P Firmicutes	C Clostridia	O Peptostreptococcales-Tissierellales	F Anaerovoracaceae	G Family XIII UCG-001	unclassified G Family XIII UCG-001
SV2315	K Bacteria	P Firmicutes	C Clostridia	O Oscillospirales	F Ruminococcaceae	unclassified F Ruminococcaceae	unclassified F Ruminococcaceae
SV2316	K Bacteria	P Bacteroidota	C Bacteroidia	O Bacteroidales	F Muribaculaceae	unclassified F Muribaculaceae	unclassified F Muribaculaceae
SV2317	K Bacteria	P Firmicutes	C Clostridia	O Clostridia UCG-014	unclassified O Clostridia UCG-014	unclassified O Clostridia UCG-014	unclassified O Clostridia UCG-014
SV2318	K_Bacteria	P_Bacteroidota	C_Bacteroidia	O_Bacteroidales	F_Muribaculaceae	unclassified_F_Muribaculaceae	unclassified_F_Muribaculaceae
SV2319	K Bacteria	P Bacteroidota	C Bacteroidia	O Bacteroidales	F Muribaculaceae	unclassified F Muribaculaceae	unclassified F Muribaculaceae
SV2320	K Bacteria	P Firmicutes	C Clostridia	O Oscillospirales	F Oscillospiraceae	G Colidextribacter	unclassified G Colidextribacter
SV2321	K Bacteria	P Firmicutes	C Clostridia	O Lachnospirales	F Lachnospiraceae	G Marvinbryantia	unclassified G Marvinbryantia
SV2322	K Bacteria	P Bacteroidota	C Bacteroidia	O Bacteroidales	F Muribaculaceae	unclassified F Muribaculaceae	unclassified F Muribaculaceae
SV2323	K Bacteria	P Firmicutes	C Clostridia	O Lachnospirales	F Lachnospiraceae	G Acetatifactor	unclassified G Acetatifactor
SV2324	K_Bacteria	P_Firmicutes	C_Clostridia	O_Clostridia UCG-014	unclassified_O_Clostridia UCG-014	unclassified_O_Clostridia UCG-014	unclassified_O_Clostridia UCG-014
SV2325	K Bacteria	P Firmicutes	C Clostridia	O Lachnospirales	F Lachnospiraceae	G Lachnospiraceae NK4A136 group	unclassified G Lachnospiraceae NK4A136 group
SV2326	K Bacteria	P Firmicutes	C Clostridia	O Lachnospirales	F Lachnospiraceae	unclassified F Lachnospiraceae	unclassified F Lachnospiraceae
SV2327	K Bacteria	P Bacteroidota	C Bacteroidia	O Bacteroidales	F Muribaculaceae	unclassified F Muribaculaceae	unclassified F Muribaculaceae
SV2328	K Bacteria	P Firmicutes	C Clostridia	O Oscillospirales	F Ruminococcaceae	unclassified F Ruminococcaceae	unclassified F Ruminococcaceae
SV2329	K Bacteria	P Firmicutes	C Bacilli	O Erysipelotrichales	F Erysipelatoclostridiaceae	G Erysipelatoclostridium	S ramosum
SV2330	K_Bacteria	P_Firmicutes	C_Clostridia	O_Oscillospirales	F_Hydrogenoanaerobacterium	unclassified_F_Hydrogenoanaerobacterium	unclassified_F_Hydrogenoanaerobacterium
SV2331	K Bacteria	P Firmicutes	C Clostridia	O Lachnospirales	F Lachnospiraceae	G Blautia	unclassified G Blautia
SV2332	K Bacteria	P Firmicutes	C Clostridia	O Clostridia UCG-014	unclassified O Clostridia UCG-014	unclassified O Clostridia UCG-014	unclassified O Clostridia UCG-014
SV2333	K Bacteria	P Firmicutes	C Clostridia	O Lachnospirales	F Lachnospiraceae	unclassified F Lachnospiraceae	unclassified F Lachnospiraceae
SV2334	K Bacteria	P Firmicutes	C Clostridia	O Oscillospirales	F Ruminococcaceae	G Paludicola	unclassified G Paludicola
SV2335	K Bacteria	P Bacteroidota	C Bacteroidia	O Bacteroidales	F Prevotellaceae	G Prevotellaceae UCG-001	unclassified G Prevotellaceae UCG-001
SV2336	K_Bacteria	P_Firmicutes	C_Bacilli	O_Erysipelotrichales	F_Erysipelatoclostridiaceae	G_Candidatus Stoquefichus	unclassified_G_Candidatus Stoquefichus
SV2337	K Bacteria	P Actinobacteriota	C Coriobacteriia	O Coriobacteriales	F Eggerthellaceae	G Enterorhabdus	unclassified G Enterorhabdus
SV2338	K Bacteria	P Firmicutes	C Bacilli	O Staphylococcales	F Staphylococcaceae	G Staphylococcus	unclassified G Staphylococcus
SV2339	K Bacteria	P Firmicutes	C Clostridia	O Lachnospirales	F Lachnospiraceae	G Tyzzerella	unclassified G Tyzzerella
SV2340	K Bacteria	P Bacteroidota	C Bacteroidia	O Bacteroidales	F Muribaculaceae	unclassified F Muribaculaceae	unclassified F Muribaculaceae
SV2341	K Bacteria	P Bacteroidota	C Bacteroidia	O Bacteroidales	F Muribaculaceae	unclassified F Muribaculaceae	unclassified F Muribaculaceae
SV2342	K Bacteria	P Bacteroidota	C Bacteroidia	O Bacteroidales	F Muribaculaceae	unclassified F Muribaculaceae	unclassified F Muribaculaceae
SV2343	K Bacteria	P Firmicutes	C Clostridia	O Lachnospirales	F Lachnospiraceae	G Lachnospiraceae UCG-006	unclassified G Lachnospiraceae UCG-006
SV2344	K Bacteria	P Firmicutes	C Clostridia	O Oscillospirales	F Oscillospiraceae	unclassified F Oscillospiraceae	unclassified F Oscillospiraceae

SV2345	K_Bacteria	P_Firmicutes	C_Clostridia	O_Clostridia UCG-014	unclassified_O_Clostridia UCG-014	unclassified_O_Clostridia UCG-014	unclassified_O_Clostridia UCG-014
SV2346	K_Bacteria	P_Bacteroidota	C_Bacteroidia	O_Bacteroidales	F_Muribaculaceae	unclassified_F_Muribaculaceae	unclassified_F_Muribaculaceae
SV2347	K_Bacteria	P_Bacteroidota	C_Bacteroidia	O_Bacteroidales	F_Muribaculaceae	unclassified_F_Muribaculaceae	unclassified_F_Muribaculaceae
SV2348	K_Bacteria	P_Firmicutes	C_Clostridia	O_Lachnospirales	F_Lachnospiraceae	unclassified_F_Lachnospiraceae	unclassified_F_Lachnospiraceae
SV2349	K_Bacteria	P_Firmicutes	C_Clostridia	O_Oscillospirales	F_Ruminococcaceae	G_[Eubacterium] siraeum group	unclassified_G_[Eubacterium] siraeum group
SV2350	K_Bacteria	P_Firmicutes	C_Clostridia	O_Oscillospirales	F_Ruminococcaceae	unclassified_F_Ruminococcaceae	unclassified_F_Ruminococcaceae
SV2351	K_Bacteria	P_Firmicutes	C_Clostridia	O_Oscillospirales	F_Oscillospiraceae	G_Colidextribacter	unclassified_G_Colidextribacter
SV2352	K_Bacteria	P_Firmicutes	C_Clostridia	O_Peptostreptococcales-Tissierellales	F_Peptostreptococcaceae	unclassified_F_Peptostreptococcaceae	unclassified_F_Peptostreptococcaceae
SV2353	K_Bacteria	P_Bacteroidota	C_Bacteroidia	O_Bacteroidales	F_Rikenellaceae	G_Rikenellaceae RC9 gut group	unclassified_G_Rikenellaceae RC9 gut group
SV2354	K_Bacteria	P_Firmicutes	C_Clostridia	O_Lachnospirales	F_Lachnospiraceae	unclassified_F_Lachnospiraceae	unclassified_F_Lachnospiraceae
SV2355	K_Bacteria	P_Bacteroidota	C_Bacteroidia	O_Bacteroidales	F_Muribaculaceae	unclassified_F_Muribaculaceae	unclassified_F_Muribaculaceae
SV2356	K_Bacteria	P_Firmicutes	C_Clostridia	O_Oscillospirales	F_Ruminococcaceae	unclassified_F_Ruminococcaceae	unclassified_F_Ruminococcaceae
SV2357	K_Bacteria	P_Firmicutes	C_Clostridia	O_Clostridia UCG-014	unclassified_O_Clostridia UCG-014	unclassified_O_Clostridia UCG-014	unclassified_O_Clostridia UCG-014
SV2358	K_Bacteria	P_Bacteroidota	C_Bacteroidia	O_Bacteroidales	F_Rikenellaceae	G_Alistipes	unclassified_G_Alistipes
SV2359	K_Bacteria	P_Firmicutes	C_Clostridia	O_Lachnospirales	F_Lachnospiraceae	G_Roseburia	unclassified_G_Roseburia
SV2360	K_Bacteria	P_Firmicutes	C_Clostridia	O_Peptostreptococcales-Tissierellales	F_Peptostreptococcaceae	unclassified_F_Peptostreptococcaceae	unclassified_F_Peptostreptococcaceae
SV2361	K_Bacteria	P_Firmicutes	C_Clostridia	O_Christensenellales	F_Christensenellaceae	G_Christensenellaceae R-7 group	unclassified_G_Christensenellaceae R-7 group
SV2362	K_Bacteria	P_Firmicutes	C_Clostridia	O_Clostridia UCG-014	unclassified_O_Clostridia UCG-014	unclassified_O_Clostridia UCG-014	unclassified_O_Clostridia UCG-014
SV2363	K_Bacteria	P_Firmicutes	C_Clostridia	O_Lachnospirales	F_Lachnospiraceae	unclassified_F_Lachnospiraceae	unclassified_F_Lachnospiraceae
SV2364	K_Bacteria	P_Firmicutes	C_Clostridia	O_Lachnospirales	F_Lachnospiraceae	G_[Eubacterium] oxidoreducens group	unclassified_G_[Eubacterium] oxidoreducens group
SV2365	K_Bacteria	P_Firmicutes	C_Bacilli	O_Staphylococcales	F_Staphylococcaceae	G_Staphylococcus	unclassified_G_Staphylococcus
SV2366	K_Bacteria	P_Firmicutes	C_Clostridia	O_Oscillospirales	F_Ruminococcaceae	G_Incertae Sedis	unclassified_G_Incertae Sedis
SV2367	K_Bacteria	P_Firmicutes	C_Clostridia	O_Lachnospirales	F_Lachnospiraceae	G_Lachnoclostridium	unclassified_G_Lachnoclostridium
SV2368	K_Bacteria	P_Firmicutes	C_Clostridia	O_Clostridia UCG-014	unclassified_O_Clostridia UCG-014	unclassified_O_Clostridia UCG-014	unclassified_O_Clostridia UCG-014
SV2369	K_Bacteria	P_Firmicutes	C_Clostridia	O_Lachnospirales	F_Lachnospiraceae	G_ASF356	unclassified_G_ASF356
SV2370	K_Bacteria	P_Firmicutes	C_Clostridia	O_Oscillospirales	F_Oscillospiraceae	G_Oscillibacter	unclassified_G_Oscillibacter
SV2371	K_Bacteria	P_Firmicutes	C_Clostridia	O_Oscillospirales	F_Ruminococcaceae	G_Ruminococcus	unclassified_G_Ruminococcus
SV2372	K_Bacteria	P_Firmicutes	C_Clostridia	O_Lachnospirales	F_Lachnospiraceae	G_Lachnospiraceae NK4A136 group	unclassified_G_Lachnospiraceae NK4A136 group
SV2373	K_Bacteria	P_Firmicutes	C_Clostridia	O_Oscillospirales	F_Ruminococcaceae	unclassified_F_Ruminococcaceae	unclassified_F_Ruminococcaceae
SV2374	K_Bacteria	P_Firmicutes	C_Clostridia	O_Oscillospirales	F_Ruminococcaceae	unclassified_F_Ruminococcaceae	unclassified_F_Ruminococcaceae
SV2375	K_Bacteria	P_Firmicutes	C_Clostridia	O_Monoglobales	F_Monoglobaceae	G_Monoglobus	unclassified_G_Monoglobus
SV2376	K_Bacteria	P_Firmicutes	C_Bacilli	O_Erysipelotrichales	F_Erysipelatoclostridiaceae	G_Candidatus Stoquefichus	unclassified_G_Candidatus Stoquefichus
SV2377	K_Bacteria	P_Bacteroidota	C_Bacteroidia	O_Bacteroidales	F_Muribaculaceae	unclassified_F_Muribaculaceae	unclassified_F_Muribaculaceae
SV2378	K_Bacteria	P_Firmicutes	C_Clostridia	O_Oscillospirales	F_Oscillospiraceae	G_Colidextribacter	unclassified_G_Colidextribacter
SV2379	K_Bacteria	P_Firmicutes	C_Clostridia	O_Oscillospirales	F_Ruminococcaceae	G_Anaerotruncus	unclassified_G_Anaerotruncus
SV2380	K_Bacteria	P_Actinobacteriota	C_Coriobacteriia	O_Coriobacteriales	F_Eggerthellaceae	G_Enterorhabdus	unclassified_G_Enterorhabdus
SV2381	K_Bacteria	P_Firmicutes	C_Clostridia	O_Lachnospirales	F_Lachnospiraceae	G_Marvinbryantia	unclassified_G_Marvinbryantia
SV2382	K_Bacteria	P_Firmicutes	C_Clostridia	O_Lachnospirales	F_Lachnospiraceae	G_GCA-900066575	unclassified_G_GCA-900066575
SV2383	K_Bacteria	P_Firmicutes	C_Clostridia	O_Oscillospirales	F_Oscillospiraceae	G_UCG-005	unclassified_G_UCG-005
SV2384	K_Bacteria	P_Firmicutes	C_Clostridia	O_Oscillospirales	F_Ruminococcaceae	G_Paludicola	unclassified_G_Paludicola
SV2385	K_Bacteria	P_Firmicutes	C_Clostridia	O_Lachnospirales	F_Lachnospiraceae	G_[Eubacterium] oxidoreducens group	unclassified_G_[Eubacterium] oxidoreducens group
SV2386	K_Bacteria	P_Firmicutes	C_Clostridia	O_Peptococcales	F_Peptococcaceae	unclassified_F_Peptococcaceae	unclassified_F_Peptococcaceae
SV2387	K_Bacteria	P_Actinobacteriota	C_Coriobacteriia	O_Coriobacteriales	F_Eggerthellaceae	G_Enterorhabdus	unclassified_G_Enterorhabdus
SV2388	K_Bacteria	P_Firmicutes	C_Clostridia	O_Lachnospirales	F_Lachnospiraceae	G_[Eubacterium] oxidoreducens group	unclassified_G_[Eubacterium] oxidoreducens group
SV2389	K_Bacteria	P_Firmicutes	C_Clostridia	O_Oscillospirales	F_Oscillospiraceae	G_Colidextribacter	unclassified_G_Colidextribacter
SV2390	K_Bacteria	P_Firmicutes	C_Clostridia	O_Lachnospirales	F_Lachnospiraceae	unclassified_F_Lachnospiraceae	unclassified_F_Lachnospiraceae
SV2391	K_Bacteria	P_Firmicutes	C_Clostridia	O_Lachnospirales	F_Lachnospiraceae	unclassified_F_Lachnospiraceae	unclassified_F_Lachnospiraceae
SV2392	K_Bacteria	P_Firmicutes	C_Clostridia	O_Lachnospirales	F_Lachnospiraceae	G_Eisenbergiella	unclassified_G_Eisenbergiella
SV2393	K_Bacteria	P_Firmicutes	C_Clostridia	O_Oscillospirales	F_Ruminococcaceae	G_Pygmaiobacter	unclassified_G_Pygmaiobacter
SV2394	K_Bacteria	P_Firmicutes	C_Clostridia	O_Lachnospirales	F_Lachnospiraceae	G_Acetatifactor	unclassified_G_Acetatifactor
SV2395	K_Bacteria	P_Bacteroidota	C_Bacteroidia	O_Bacteroidales	F_Rikenellaceae	G_Alistipes	unclassified_G_Alistipes
SV2396	K_Bacteria	P_Firmicutes	C_Clostridia	O_Oscillospirales	F_Ruminococcaceae	unclassified_F_Ruminococcaceae	unclassified_F_Ruminococcaceae
SV2397	K_Bacteria	P_Firmicutes	C_Clostridia	O_Peptostreptococcales-Tissierellales	F_Peptostreptococcaceae	unclassified_F_Peptostreptococcaceae	unclassified_F_Peptostreptococcaceae
SV2398	K_Bacteria	P_Bacteroidota	C_Bacteroidia	O_Bacteroidales	F_Muribaculaceae	unclassified_F_Muribaculaceae	unclassified_F_Muribaculaceae
SV2399	K_Bacteria	P_Firmicutes	C_Clostridia	O_Lachnospirales	F_Lachnospiraceae	G_Lachnospiraceae NK4A136 group	unclassified_G_Lachnospiraceae NK4A136 group
SV2400	K_Bacteria	P_Firmicutes	C_Clostridia	O_Peptostreptococcales-Tissierellales	F_Peptostreptococcaceae	G_Romboutsia	unclassified_G_Romboutsia
SV2401	K_Bacteria	P_Firmicutes	C_Clostridia	O_Oscillospirales	F_Ruminococcaceae	unclassified_F_Ruminococcaceae	unclassified_F_Ruminococcaceae
SV2402	K_Bacteria	P_Firmicutes	C_Clostridia	O_Lachnospirales	F_Lachnospiraceae	unclassified_F_Lachnospiraceae	unclassified_F_Lachnospiraceae
SV2403	K_Bacteria	P_Proteobacteria	C_Alphaproteobacteria	O_Rhodospirillales	unclassified_O_Rhodospirillales	unclassified_O_Rhodospirillales	unclassified_O_Rhodospirillales
SV2404	K_Bacteria	P_Firmicutes	C_Clostridia	O_Oscillospirales	F_Oscillospiraceae	unclassified_F_Oscillospiraceae	unclassified_F_Oscillospiraceae
SV2405	K_Bacteria	P_Firmicutes	C_Clostridia	O_Peptostreptococcales-Tissierellales	F_Peptostreptococcaceae	unclassified_F_Peptostreptococcaceae	unclassified_F_Peptostreptococcaceae
SV2406	K_Bacteria	P_Firmicutes	C_Clostridia	O_Oscillospirales	F_Oscillospiraceae	G_Colidextribacter	unclassified_G_Colidextribacter
SV2407	K_Bacteria	P_Firmicutes	C_Bacilli	O_Erysipelotrichales	F_Erysipelotrichaceae	G_Turicibacter	unclassified_G_Turicibacter
SV2408	K_Bacteria	P_Firmicutes	C_Clostridia	O_Peptostreptococcales-Tissierellales	F_Anaerovoracaceae	G_Family XIII AD3011 group	unclassified_G_Family XIII AD3011 group
SV2409	K_Bacteria	P_Firmicutes	C_Clostridia	O_Lachnospirales	F_Lachnospiraceae	unclassified_F_Lachnospiraceae	unclassified_F_Lachnospiraceae
SV2410	K_Bacteria	P_Firmicutes	C_Bacilli	O_Erysipelotrichales	F_Erysipelotrichaceae	G_Faecalibaculum	unclassified_G_Faecalibaculum
SV2411	K_Bacteria	P_Firmicutes	C_Clostridia	O_Clostridiales	F_Clostridiaceae	G_Clostridium sensu stricto 1	unclassified_G_Clostridium sensu stricto 1
SV2412	K_Bacteria	P_Firmicutes	C_Clostridia	unclassified_C_Clostridia	unclassified_C_Clostridia	unclassified_C_Clostridia	unclassified_C_Clostridia
SV2413	K_Bacteria	P_Firmicutes	C_Clostridia	O_Oscillospirales	F_Ruminococcaceae	G_Incertae Sedis	unclassified_G_Incertae Sedis
SV2414	K_Bacteria	P_Bacteroidota	C_Bacteroidia	O_Bacteroidales	F_Bacteroidaceae	G_Bacteroides	unclassified_G_Bacteroides
SV2415	K_Bacteria	P_Firmicutes	C_Clostridia	O_Peptostreptococcales-Tissierellales	F_Peptostreptococcaceae	unclassified_F_Peptostreptococcaceae	unclassified_F_Peptostreptococcaceae
SV2416	K_Bacteria	P_Firmicutes	C_Clostridia	O_Lachnospirales	F_Lachnospiraceae	G_Lachnospiraceae NK4A136 group	unclassified_G_Lachnospiraceae NK4A136 group
SV2417	K_Bacteria	P_Bacteroidota	C_Bacteroidia	O_Bacteroidales	F_Muribaculaceae	unclassified_F_Muribaculaceae	unclassified_F_Muribaculaceae
SV2418	K_Bacteria	P_Firmicutes	C_Clostridia	O_Oscillospirales	F_Oscillospiraceae	unclassified_F_Oscillospiraceae	unclassified_F_Oscillospiraceae
SV2419	K_Bacteria	P_Firmicutes	C_Clostridia	O_Lachnospirales	F_Lachnospiraceae	unclassified_F_Lachnospiraceae	unclassified_F_Lachnospiraceae
SV2420	K_Bacteria	P_Firmicutes	C_Bacilli	O_Lactobacillales	F_Lactobacillaceae	unclassified_F_Lactobacillaceae	unclassified_F_Lactobacillaceae
SV2421	K_Bacteria	P_Firmicutes	C_Bacilli	O_Lactobacillales	F_Lactobacillaceae	unclassified_F_Lactobacillaceae	unclassified_F_Lactobacillaceae
SV2422	K_Bacteria	P_Bacteroidota	C_Bacteroidia	O_Bacteroidales	F_Muribaculaceae	unclassified_F_Muribaculaceae	unclassified_F_Muribaculaceae
SV2423	K_Bacteria	P_Firmicutes	C_Clostridia	O_Clostridia UCG-014	unclassified_O_Clostridia UCG-014	unclassified_O_Clostridia UCG-014	unclassified_O_Clostridia UCG-014
SV2424	K_Bacteria	P_Firmicutes	C_Clostridia	O_Monoglobales	F_Monoglobaceae	G_Monoglobus	unclassified_G_Monoglobus
SV2425	K_Bacteria	P_Firmicutes	C_Clostridia	O_Lachnospirales	F_Lachnospiraceae	G_Lachnoclostridium	unclassified_G_Lachnoclostridium
SV2426	K_Bacteria	P_Bacteroidota	C_Bacteroidia	O_Bacteroidales	F_Bacteroidaceae	G_Bacteroides	unclassified_G_Bacteroides
SV2427	K_Bacteria	P_Firmicutes	C_Clostridia	O_Peptostreptococcales-Tissierellales	F_Peptostreptococcaceae	unclassified_F_Peptostreptococcaceae	unclassified_F_Peptostreptococcaceae
SV2428	K_Bacteria	P_Firmicutes	C_Clostridia	O_Oscillospirales	F_Oscillospiraceae	G_Oscillibacter	unclassified_G_Oscillibacter
SV2429	K_Bacteria	P_Firmicutes	C_Clostridia	O_Oscillospirales	F_Ruminococcaceae	unclassified_F_Ruminococcaceae	unclassified_F_Ruminococcaceae
SV2430	K_Bacteria	P_Firmicutes	C_Clostridia	O_Clostridia UCG-014	unclassified_O_Clostridia UCG-014	unclassified_O_Clostridia UCG-014	unclassified_O_Clostridia UCG-014
SV2431	K_Bacteria	P_Firmicutes	C_Clostridia	O_Lachnospirales	F_Lachnospiraceae	unclassified_F_Lachnospiraceae	unclassified_F_Lachnospiraceae
SV2432	K_Bacteria	P_Firmicutes	C_Clostridia	O_Lachnospirales	F_Lachnospiraceae	unclassified_F_Lachnospiraceae	unclassified_F_Lachnospiraceae
SV2433	K_Bacteria	P_Proteobacteria	C_Gammaproteobacteria	O_Burkholderiales	F_Sutterellaceae	G_Parasutterella	unclassified_G_Parasutterella
SV2434	K_Bacteria	P_Firmicutes	C_Clostridia	O_Lachnospirales	F_Lachnospiraceae	G_[Eubacterium] ventriosum group	unclassified_G_[Eubacterium] ventriosum group
SV2435	K_Bacteria	P_Firmicutes	C_Clostridia	O_Lachnospirales	F_Lachnospiraceae	unclassified_F_Lachnospiraceae	unclassified_F_Lachnospiraceae
SV2436	K_Bacteria	P_Firmicutes	C_Clostridia	O_Lachnospirales	F_Lachnospiraceae	G_Roseburia	unclassified_G_Roseburia
SV2437	K_Bacteria	P_Bacteroidota	C_Bacteroidia	O_Bacteroidales	F_Muribaculaceae	unclassified_F_Muribaculaceae	unclassified_F_Muribaculaceae
SV2438	K_Bacteria	P_Firmicutes	C_Bacilli	O_Erysipelotrichales	F_Erysipelotrichaceae	G_Faecalibaculum	unclassified_G_Faecalibaculum
SV2439	K_Bacteria	P_Firmicutes	C_Clostridia	O_Peptostreptococcales-Tissierellales	F_Anaerovoracaceae	G_Family XIII AD3011 group	unclassified_G_Family XIII AD3011 group
SV2440	K_Bacteria	P_Firmicutes	C_Clostridia	O_Oscillospirales	F_Oscillospiraceae	G_Oscillibacter	unclassified_G_Oscillibacter
SV2441	K_Bacteria	P_Firmicutes	C_Clostridia	O_Oscillospirales	F_Oscillospiraceae	G_Oscillibacter	unclassified_G_Oscillibacter
SV2442	K_Bacteria	P_Firmicutes	C_Bacilli	O_Erysipelotrichales	F_Erysipelatoclostridiaceae	G_Candidatus Stoquefichus	unclassified_G_Candidatus Stoquefichus
SV2443	K_Bacteria	P_Firmicutes	C_Clostridia	O_Lachnospirales	F_Lachnospiraceae	unclassified_F_Lachnospiraceae	unclassified_F_Lachnospiraceae
SV2444	K_Bacteria	P_Firmicutes	C_Clostridia	O_Oscillospirales	F_Oscillospiraceae	G_Intestinimonas	unclassified_G_Intestinimonas
SV2445	K_Bacteria	P_Firmicutes	C_Clostridia	O_Lachnospirales	F_Lachnospiraceae	G_Lachnospiraceae UCG-006	unclassified_G_Lachnospiraceae UCG-006
SV2446	K_Bacteria	P_Bacteroidota	C_Bacteroidia	O_Bacteroidales	F_Muribaculaceae	unclassified_F_Muribaculaceae	unclassified_F_Muribaculaceae
SV2447	K_Bacteria	P_Firmicutes	C_Clostridia	O_Lachnospirales	F_Lachnospiraceae	G_Lachnospiraceae FCS020 group	unclassified_G_Lachnospiraceae FCS020 group
SV2448	K_Bacteria	P_Bacteroidota	C_Bacteroidia	O_Bacteroidales	F_Muribaculaceae	unclassified_F_Muribaculaceae	unclassified_F_Muribaculaceae
SV2449	K_Bacteria	P_Firmicutes	C_Bacilli	O_Erysipelotrichales	F_Erysipelotrichaceae	G_Dubosiella	unclassified_G_Dubosiella
SV2450	K_Bacteria	P_Firmicutes	C_Clostridia	O_Oscillospirales	F_Ruminococcaceae	unclassified_F_Ruminococcaceae	unclassified_F_Ruminococcaceae
SV2451	K_Bacteria	P_Firmicutes	C_Clostridia	O_Lachnospirales	F_Lachnospiraceae	G_[Eubacterium] ventriosum group	unclassified_G_[Eubacterium] ventriosum group
SV2452	K_Bacteria	P_Firmicutes	C_Clostridia	O_Lachnospirales	F_Lachnospiraceae	G_GCA-900066575	unclassified_G_GCA-900066575
SV2453	K_Bacteria	P_Firmicutes	C_Clostridia	O_Lachnospirales	F_Lachnospiraceae	G_Lachnospiraceae NK4A136 group	unclassified_G_Lachnospiraceae NK4A136 group
SV2454	K_Bacteria	P_Firmicutes	C_Bacilli	O_Erysipelotrichales	F_Erysipelotrichaceae	G_Turicibacter	unclassified_G_Turicibacter
SV2455	K_Bacteria	P_Firmicutes	C_Clostridia	O_Oscillospirales	F_Ruminococcaceae	G_Negativibacillus	unclassified_G_Negativibacillus
SV2456	K_Bacteria	P_Firmicutes	C_Clostridia	O_Oscillospirales	F_Oscillospiraceae	G_Intestinimonas	unclassified_G_Intestinimonas
SV2457	K_Bacteria	P_Firmicutes	C_Clostridia	O_Oscillospirales	F_Oscillospiraceae	unclassified_F_Oscillospiraceae	unclassified_F_Oscillospiraceae
SV2458	K_Bacteria	P_Bacteroidota	C_Bacteroidia	O_Bacteroidales	F_Muribaculaceae	unclassified_F_Muribaculaceae	unclassified_F_Muribaculaceae
SV2459	K_Bacteria	P_Firmicutes	C_Clostridia	O_Lachnospirales	F_Lachnospiraceae	G_Lachnospiraceae NK4A136 group	unclassified_G_Lachnospiraceae NK4A136 group
SV2460	K_Bacteria	P_Firmicutes	C_Clostridia	O_Oscillospirales	F_Ruminococcaceae	unclassified_F_Ruminococcaceae	unclassified_F_Ruminococcaceae
SV2461	K_Bacteria	P_Firmicutes	C_Clostridia	O_Peptostreptococcales-Tissierellales	F_Anaerovoracaceae	unclassified_F_Anaerovoracaceae	unclassified_F_Anaerovoracaceae
SV2462	K_Bacteria	P_Firmicutes	C_Clostridia	O_Lachnospirales	F_Lachnospiraceae	G_GCA-900066575	unclassified_G_GCA-900066575
SV2463	K_Bacteria	P_Firmicutes	C_Clostridia	O_Lachnospirales	F_Lachnospiraceae	G_Lachnospiraceae NK4A136 group	unclassified_G_Lachnospiraceae NK4A136 group
SV2464	K_Bacteria	P_Firmicutes	C_Bacilli	O_Erysipelotrichales	F_Erysipelotrichaceae	G_Faecalibaculum	unclassified_G_Faecalibaculum
SV2465	K_Bacteria	P_Firmicutes	C_Clostridia	O_Lachnospirales	F_Lachnospiraceae	G_Roseburia	unclassified_G_Roseburia
SV2466	K_Bacteria	P_Firmicutes	C_Clostridia	O_Lachnospirales	F_Lachnospiraceae	unclassified_F_Lachnospiraceae	unclassified_F_Lachnospiraceae
SV2467	K_Bacteria	P_Firmicutes	C_Clostridia	O_Oscillospirales	F_Oscillospiraceae	G_Colidextribacter	unclassified_G_Colidextribacter
SV2468	K_Bacteria	P_Firmicutes	C_Clostridia	O_Oscillospirales	F_Ruminococcaceae	G_Ruminococcus	unclassified_G_Ruminococcus
SV2469	K_Bacteria	P_Firmicutes	C_Bacilli	O_RF39	unclassified_O_RF39	unclassified_O_RF39	unclassified_O_RF39
SV2470	K_Bacteria	P_Actinobacteriota	C_Coriobacteriia	O_Coriobacteriales	F_Eggerthellaceae	G_Enterorhabdus	S_mucosicola
SV2471	K_Bacteria	P_Firmicutes	C_Clostridia	O_Oscillospirales	F_Oscillospiraceae	G_Colidextribacter	unclassified_G_Colidextribacter
SV2472	K_Bacteria	P_Bacteroidota	C_Bacteroidia	O_Bacteroidales	F_Prevotellaceae	G_Prevotellaceae UCG-001	unclassified_G_Prevotellaceae UCG-001
SV2473	K_Bacteria	P_Firmicutes	C_Clostridia	O_Lachnospirales	F_Lachnospiraceae	G_ASF356	unclassified_G_ASF356
SV2474	K_Bacteria	P_Firmicutes	C_Clostridia	O_Peptostreptococcales-Tissierellales	F_Anaerovoracaceae	G_[Eubacterium] brachy group	unclassified_G_[Eubacterium] brachy group
SV2475	K_Bacteria	P_Firmicutes	C_Clostridia	O_Lachnospirales	F_Lachnospiraceae	G_Lachnospiraceae UCG-008	unclassified_G_Lachnospiraceae UCG-008
SV2476	K_Bacteria	P_Firmicutes	C_Clostridia	O_Oscillospirales	F_Ruminococcaceae	G_Incertae Sedis	unclassified_G_Incertae Sedis
SV2477	K_Bacteria	P_Firmicutes	C_Clostridia	O_Lachnospirales	F_Lachnospiraceae	unclassified_F_Lachnospiraceae	unclassified_F_Lachnospiraceae
SV2478	K_Bacteria	P_Firmicutes	C_Clostridia	O_Oscillospirales	F_Ruminococcaceae	G_Harryflintia	unclassified_G_Harryflintia
SV2479	K_Bacteria	P_Firmicutes	C_Clostridia	O_Oscillospirales	F_Ruminococcaceae	unclassified_F_Ruminococcaceae	unclassified_F_Ruminococcaceae
SV2480	K_Bacteria	P_Firmicutes	C_Bacilli	O_Erysipelotrichales	F_Erysipelotrichaceae	unclassified_F_Erysipelotrichaceae	unclassified_F_Erysipelotrichaceae
SV2481	K_Bacteria	P_Firmicutes	C_Clostridia	O_Lachnospirales	F_Lachnospiraceae	G_Anaerostipes	unclassified_G_Anaerostipes
SV2482	K_Bacteria	P_Firmicutes	C_Bacilli	O_Erysipelotrichales	F_Erysipelotrichaceae	G_Turicibacter	unclassified_G_Turicibacter

SV2483	K Bacteria	P Actinobacteriota	C Coriobacteriia	O Coriobacteriales	F Eggerthellaceae	G Enterorhabdus	unclassified G Enterorhabdus
SV2484	K Bacteria	P Cyanobacteria	C Vampirivibrionia	O Gastranaerophilales	unclassified O Gastranaerophilales	unclassified O Gastranaerophilales	unclassified O Gastranaerophilales
SV2485	K Bacteria	P Firmicutes	C Clostridia	O Lachnospirales	F Lachnospiraceae	G [Eubacterium] oxidoreducens group	unclassified G [Eubacterium] oxidoreducens group
SV2486	K Bacteria	P Firmicutes	C Clostridia	O Lachnospirales	F Lachnospiraceae	G Lachnospiraceae NK4A136 group	unclassified G Lachnospiraceae NK4A136 group
SV2487	K Bacteria	P Firmicutes	C Clostridia	O Peptostreptococcales-Tissierellales	F Anaerovoracaceae	G Anaerovorax	unclassified G Anaerovorax
SV2488	K Bacteria	P Firmicutes	C Clostridia	O Oscillospirales	F Oscillospiraceae	G Intestinimonas	unclassified G Intestinimonas
SV2489	K Bacteria	P Firmicutes	C Clostridia	O Peptostreptococcales-Tissierellales	F Peptostreptococcaceae	unclassified F Peptostreptococcaceae	unclassified F Peptostreptococcaceae
SV2490	K Bacteria	P Firmicutes	C Bacilli	O Lactobacillales	F Lactobacillaceae	unclassified F Lactobacillaceae	unclassified F Lactobacillaceae
SV2491	K Bacteria	P Firmicutes	C Bacilli	O Lactobacillales	F Lactobacillaceae	unclassified F Lactobacillaceae	unclassified F Lactobacillaceae
SV2492	K Bacteria	P Firmicutes	C Clostridia	O Clostridia UCG-014	unclassified O Clostridia UCG-014	unclassified O Clostridia UCG-014	unclassified O Clostridia UCG-014
SV2493	K Bacteria	P Firmicutes	C Clostridia	O Clostridia UCG-014	unclassified O Clostridia UCG-014	unclassified O Clostridia UCG-014	unclassified O Clostridia UCG-014
SV2494	K Bacteria	P Firmicutes	C Clostridia	O Lachnospirales	F Lachnospiraceae	G ASF356	unclassified G ASF356
SV2495	K Bacteria	P Firmicutes	C Clostridia	O Peptostreptococcales-Tissierellales	F Peptostreptococcaceae	unclassified F Peptostreptococcaceae	unclassified F Peptostreptococcaceae
SV2496	K Bacteria	P Bacteroidota	C Bacteroidia	O Bacteroidales	F Rikenellaceae	unclassified F Rikenellaceae	unclassified F Rikenellaceae
SV2497	K Bacteria	P Firmicutes	C Clostridia	O Oscillospirales	F Oscillospiraceae	G Intestinimonas	unclassified G Intestinimonas
SV2498	K Bacteria	P Firmicutes	C Clostridia	O Oscillospirales	F Oscillospiraceae	unclassified F Oscillospiraceae	unclassified F Oscillospiraceae
SV2499	K Bacteria	P Firmicutes	C Clostridia	O Clostridia UCG-014	unclassified O Clostridia UCG-014	unclassified O Clostridia UCG-014	unclassified O Clostridia UCG-014
SV2500	K Bacteria	P Firmicutes	C Bacilli	O Lactobacillales	F Lactobacillaceae	G HT002	unclassified G HT002
SV2501	K Bacteria	P Firmicutes	C Clostridia	O Peptostreptococcales-Tissierellales	F Anaerovoracaceae	G Family XIII UCG-001	unclassified G Family XIII UCG-001
SV2502	K Bacteria	P Firmicutes	C Clostridia	O Lachnospirales	F Lachnospiraceae	G Lachnoclostridium	unclassified G Lachnoclostridium
SV2503	K Bacteria	P Firmicutes	C Clostridia	O Lachnospirales	F Lachnospiraceae	unclassified F Lachnospiraceae	unclassified F Lachnospiraceae
SV2504	K Bacteria	P Firmicutes	C Clostridia	O Lachnospirales	F Lachnospiraceae	G Tyzzerella	unclassified G Tyzzerella
SV2505	K Bacteria	P Bacteroidota	C Bacteroidia	O Bacteroidales	F Rikenellaceae	G Alistipes	unclassified G Alistipes
SV2506	K Bacteria	P Firmicutes	C Clostridia	O Lachnospirales	F Lachnospiraceae	G [Eubacterium] fissicatena group	unclassified G [Eubacterium] fissicatena group
SV2507	K Bacteria	P Firmicutes	C Clostridia	O Lachnospirales	F Lachnospiraceae	unclassified F Lachnospiraceae	unclassified F Lachnospiraceae
SV2508	K Bacteria	P Firmicutes	C Clostridia	O Christensenellales	F Christensenellaceae	G Christensenellaceae R-7 group	unclassified G Christensenellaceae R-7 group
SV2509	K Bacteria	P Firmicutes	C Clostridia	O Lachnospirales	F Lachnospiraceae	G Lachnospiraceae UCG-006	unclassified G Lachnospiraceae UCG-006
SV2510	K Bacteria	P Firmicutes	C Clostridia	O Lachnospirales	F Lachnospiraceae	G Lachnospiraceae NK4A136 group	unclassified G Lachnospiraceae NK4A136 group
SV2511	K Bacteria	P Firmicutes	C Clostridia	O Oscillospirales	F Oscillospiraceae	G Colidextribacter	unclassified G Colidextribacter
SV2512	K Bacteria	P Firmicutes	C Clostridia	O Lachnospirales	F Lachnospiraceae	unclassified F Lachnospiraceae	unclassified F Lachnospiraceae
SV2513	K Bacteria	P Firmicutes	C Clostridia	O Lachnospirales	F Lachnospiraceae	G Lachnospiraceae UCG-008	unclassified G Lachnospiraceae UCG-008
SV2514	K Bacteria	P Firmicutes	C Clostridia	O Lachnospirales	F Lachnospiraceae	unclassified F Lachnospiraceae	unclassified F Lachnospiraceae
SV2515	K Bacteria	P Bacteroidota	C Bacteroidia	O Bacteroidales	F Muribaculaceae	unclassified F Muribaculaceae	unclassified F Muribaculaceae
SV2516	K Bacteria	P Firmicutes	C Clostridia	O Clostridia vadinBB60 group	unclassified O Clostridia vadinBB60 group	unclassified O Clostridia vadinBB60 group	unclassified O Clostridia vadinBB60 group
SV2517	K Bacteria	P Cyanobacteria	C Vampirivibrionia	O Gastranaerophilales	unclassified O Gastranaerophilales	unclassified O Gastranaerophilales	unclassified O Gastranaerophilales
SV2518	K Bacteria	P Bacteroidota	C Bacteroidia	unclassified C Bacteroidia	unclassified C Bacteroidia	unclassified C Bacteroidia	unclassified C Bacteroidia
SV2519	K Bacteria	P Firmicutes	C Clostridia	O Peptostreptococcales-Tissierellales	F Peptostreptococcaceae	unclassified F Peptostreptococcaceae	unclassified F Peptostreptococcaceae
SV2520	K Bacteria	P Firmicutes	C Bacilli	O RF39	unclassified O RF39	unclassified O RF39	unclassified O RF39
SV2521	K Bacteria	P Firmicutes	C Clostridia	O Oscillospirales	F Ruminococcaceae	G DTU089	unclassified G DTU089
SV2522	K Bacteria	P Firmicutes	C Bacilli	O RF39	unclassified O RF39	unclassified O RF39	unclassified O RF39
SV2523	K Bacteria	P Firmicutes	C Bacilli	O Erysipelotrichales	F Erysipelatoclostridiaceae	G Candidatus Stoquefichus	unclassified G Candidatus Stoquefichus
SV2524	K Bacteria	P Firmicutes	C Clostridia	O Lachnospirales	F Lachnospiraceae	G Lachnospiraceae UCG-008	unclassified G Lachnospiraceae UCG-008
SV2525	K Bacteria	P Bacteroidota	C Bacteroidia	O Bacteroidales	F Muribaculaceae	unclassified F Muribaculaceae	unclassified F Muribaculaceae
SV2526	K Bacteria	P Firmicutes	C Clostridia	O Lachnospirales	F Lachnospiraceae	G Lachnospiraceae NK4A136 group	unclassified G Lachnospiraceae NK4A136 group
SV2527	K Bacteria	P Bacteroidota	C Bacteroidia	O Bacteroidales	F Muribaculaceae	unclassified F Muribaculaceae	unclassified F Muribaculaceae
SV2528	K Bacteria	P Firmicutes	C Clostridia	O Oscillospirales	F Oscillospiraceae	unclassified F Oscillospiraceae	unclassified F Oscillospiraceae
SV2529	K_Bacteria	P_Firmicutes	C_Clostridia	O_Peptostreptococcales-Tissierellales	F_Peptostreptococcaceae	unclassified_F_Peptostreptococcaceae	unclassified_F_Peptostreptococcaceae
SV2530	K Bacteria	P Firmicutes	C Clostridia	O Lachnospirales	F Lachnospiraceae	unclassified F Lachnospiraceae	unclassified F Lachnospiraceae
SV2531	K Bacteria	P Bacteroidota	C Bacteroidia	O Bacteroidales	F Prevotellaceae	G Prevotellaceae UCG-001	unclassified G Prevotellaceae UCG-001
SV2532	K Bacteria	P Firmicutes	C Clostridia	O Clostridia UCG-014	unclassified O Clostridia UCG-014	unclassified O Clostridia UCG-014	unclassified O Clostridia UCG-014
SV2533	K Bacteria	P Firmicutes	C Clostridia	O Lachnospirales	F Lachnospiraceae	unclassified F Lachnospiraceae	unclassified F Lachnospiraceae
SV2534	K Bacteria	P Bacteroidota	C Bacteroidia	O Bacteroidales	F Muribaculaceae	unclassified F Muribaculaceae	unclassified F Muribaculaceae
SV2535	K_Bacteria	P_Firmicutes	C_Clostridia	O_Lachnospirales	F_Lachnospiraceae	G_Roseburia	unclassified_G_Roseburia
SV2536	K Bacteria	P Bacteroidota	C Bacteroidia	O Bacteroidales	F Prevotellaceae	G Prevotellaceae UCG-001	unclassified G Prevotellaceae UCG-001
SV2537	K Bacteria	P Bacteroidota	C Bacteroidia	O Bacteroidales	F Muribaculaceae	unclassified F Muribaculaceae	unclassified F Muribaculaceae
SV2538	K Bacteria	P Firmicutes	C Clostridia	O Clostridia UCG-014	unclassified O Clostridia UCG-014	unclassified O Clostridia UCG-014	unclassified O Clostridia UCG-014
SV2539	K Bacteria	P Firmicutes	C Clostridia	O Lachnospirales	F Lachnospiraceae	G Lachnospiraceae NK4A136 group	unclassified G Lachnospiraceae NK4A136 group
SV2540	K Bacteria	P Actinobacteriota	C Coriobacteriia	O Coriobacteriales	F Eggerthellaceae	G Enterorhabdus	unclassified G Enterorhabdus
SV2541	K_Bacteria	P_Bacteroidota	C_Bacteroidia	O_Bacteroidales	F_Muribaculaceae	unclassified_F_Muribaculaceae	unclassified_F_Muribaculaceae
SV2542	K Bacteria	P Firmicutes	C Clostridia	O Peptostreptococcales-Tissierellales	F Anaerovoracaceae	G [Eubacterium] brachy group	unclassified G [Eubacterium] brachy group
SV2543	K Bacteria	P Firmicutes	C Clostridia	O Oscillospirales	F Oscillospiraceae	unclassified F Oscillospiraceae	unclassified F Oscillospiraceae
SV2544	K Bacteria	P Firmicutes	C Clostridia	O Oscillospirales	F Oscillospiraceae	unclassified F Oscillospiraceae	unclassified F Oscillospiraceae
SV2545	K Bacteria	P Firmicutes	C Clostridia	O Lachnospirales	F Lachnospiraceae	unclassified F Lachnospiraceae	unclassified F Lachnospiraceae
SV2546	K Bacteria	P Firmicutes	C Clostridia	O Lachnospirales	F Lachnospiraceae	unclassified F Lachnospiraceae	unclassified F Lachnospiraceae
SV2547	K_Bacteria	P_Firmicutes	C_Bacilli	O_Erysipelotrichales	F_Erysipelotrichaceae	G_Faecalibaculum	unclassified_G_Faecalibaculum
SV2548	K Bacteria	P Firmicutes	C Clostridia	O Oscillospirales	F Ruminococcaceae	unclassified F Ruminococcaceae	unclassified F Ruminococcaceae
SV2549	K Bacteria	P Bacteroidota	C Bacteroidia	O Bacteroidales	F Bacteroidaceae	G Bacteroides	unclassified G Bacteroides
SV2550	K Bacteria	P Firmicutes	C Clostridia	O Peptostreptococcales-Tissierellales	F Peptostreptococcaceae	unclassified F Peptostreptococcaceae	unclassified F Peptostreptococcaceae
SV2551	K Bacteria	P Firmicutes	C Clostridia	O Lachnospirales	F Lachnospiraceae	unclassified F Lachnospiraceae	unclassified F Lachnospiraceae
SV2552	K Bacteria	P Bacteroidota	C Bacteroidia	O Bacteroidales	F Rikenellaceae	G Alistipes	unclassified G Alistipes
SV2553	K_Bacteria	P_Firmicutes	C_Clostridia	O_Lachnospirales	F_Lachnospiraceae	unclassified_F_Lachnospiraceae	unclassified_F_Lachnospiraceae
SV2554	K Bacteria	P Firmicutes	C Clostridia	O Oscillospirales	F Ruminococcaceae	G Harryflintia	unclassified G Harryflintia
SV2555	K Bacteria	P Firmicutes	C Bacilli	O Erysipelotrichales	F Erysipelatoclostridiaceae	G Erysipelatoclostridium	unclassified G Erysipelatoclostridium
SV2556	K Bacteria	P Firmicutes	C Clostridia	O Clostridia UCG-014	unclassified O Clostridia UCG-014	unclassified O Clostridia UCG-014	unclassified O Clostridia UCG-014
SV2557	K Bacteria	P Firmicutes	C Clostridia	O Oscillospirales	F Hungateiclostridiaceae	G Ruminiclostridium	unclassified G Ruminiclostridium
SV2558	K Bacteria	P Firmicutes	C Bacilli	O Erysipelotrichales	F Erysipelatoclostridiaceae	G Erysipelatoclostridium	unclassified G Erysipelatoclostridium
SV2559	K Bacteria	P Firmicutes	C Clostridia	O Oscillospirales	F Ruminococcaceae	unclassified F Ruminococcaceae	unclassified F Ruminococcaceae
SV2560	K Bacteria	unclassified K Bacteria	unclassified K Bacteria	unclassified K Bacteria	unclassified K Bacteria	unclassified K Bacteria	unclassified K Bacteria
SV2561	K Bacteria	P Firmicutes	C Clostridia	O Oscillospirales	F Oscillospiraceae	G Colidextribacter	unclassified G Colidextribacter
SV2562	K Bacteria	P Firmicutes	C Clostridia	O Clostridia UCG-014	unclassified O Clostridia UCG-014	unclassified O Clostridia UCG-014	unclassified O Clostridia UCG-014
SV2563	K Bacteria	P Firmicutes	C Clostridia	O Clostridia UCG-014	unclassified O Clostridia UCG-014	unclassified O Clostridia UCG-014	unclassified O Clostridia UCG-014
SV2564	K Bacteria	P Firmicutes	C Clostridia	O Peptostreptococcales-Tissierellales	F Peptostreptococcaceae	unclassified F Peptostreptococcaceae	unclassified F Peptostreptococcaceae
SV2565	K Bacteria	P Firmicutes	C Clostridia	O Oscillospirales	F Ruminococcaceae	G Anaerotruncus	unclassified G Anaerotruncus
SV2566	K Bacteria	P Firmicutes	C Clostridia	O Oscillospirales	F Ruminococcaceae	G Ruminococcus	unclassified G Ruminococcus
SV2567	K Bacteria	P Firmicutes	C Clostridia	O Lachnospirales	F Lachnospiraceae	unclassified F Lachnospiraceae	unclassified F Lachnospiraceae
SV2568	K Bacteria	P Firmicutes	C Clostridia	O Monoglobales	F Monoglobaceae	G Monoglobus	unclassified G Monoglobus
SV2569	K Bacteria	P Bacteroidota	C Bacteroidia	O Bacteroidales	F Muribaculaceae	unclassified F Muribaculaceae	unclassified F Muribaculaceae
SV2570	K Bacteria	P Firmicutes	C Bacilli	O Lactobacillales	F Lactobacillaceae	G Ligilactobacillus	unclassified G Ligilactobacillus
SV2571	K Bacteria	P Firmicutes	C Clostridia	O Lachnospirales	F Lachnospiraceae	G GCA-900066575	unclassified G GCA-900066575
SV2572	K Bacteria	P Firmicutes	C Bacilli	O Erysipelotrichales	F Erysipelotrichaceae	G Turicibacter	unclassified G Turicibacter
SV2573	K Bacteria	P Firmicutes	C Clostridia	O Peptostreptococcales-Tissierellales	F Peptostreptococcaceae	unclassified F Peptostreptococcaceae	unclassified F Peptostreptococcaceae
SV2574	K Bacteria	P Firmicutes	C Clostridia	O Clostridiales	F Clostridiaceae	G Clostridium sensu stricto 1	unclassified G Clostridium sensu stricto 1
SV2575	K Bacteria	P Firmicutes	C Clostridia	O Peptostreptococcales-Tissierellales	F Anaerovoracaceae	G Family XIII UCG-001	unclassified G Family XIII UCG-001
SV2576	K Bacteria	P Firmicutes	C Clostridia	O Lachnospirales	F Defluviitaleaceae	G Defluviitaleaceae UCG-011	unclassified G Defluviitaleaceae UCG-011
SV2577	K Bacteria	P Firmicutes	C Clostridia	O Lachnospirales	F Lachnospiraceae	G Lachnoclostridium	unclassified G Lachnoclostridium
SV2578	K Bacteria	P Firmicutes	C Clostridia	O Oscillospirales	F Ruminococcaceae	G Incertae Sedis	unclassified G Incertae Sedis
SV2579	K Bacteria	P Firmicutes	C Clostridia	O Oscillospirales	F Oscillospiraceae	G Oscillibacter	unclassified G Oscillibacter
SV2580	K Bacteria	P Firmicutes	C Clostridia	O Lachnospirales	F Lachnospiraceae	unclassified F Lachnospiraceae	unclassified F Lachnospiraceae
SV2581	K Bacteria	P Bacteroidota	C Bacteroidia	O Bacteroidales	F Bacteroidaceae	G Bacteroides	unclassified G Bacteroides
SV2582	K Bacteria	P Bacteroidota	C Bacteroidia	O Bacteroidales	F Muribaculaceae	unclassified F Muribaculaceae	unclassified F Muribaculaceae
SV2583	K Bacteria	P Firmicutes	C Clostridia	O Lachnospirales	F Lachnospiraceae	unclassified F Lachnospiraceae	unclassified F Lachnospiraceae
SV2584	K Bacteria	P Firmicutes	C Bacilli	O Erysipelotrichales	F Erysipelatoclostridiaceae	G Erysipelatoclostridium	unclassified G Erysipelatoclostridium
SV2585	K Bacteria	P Actinobacteriota	C Coriobacteriia	O Coriobacteriales	F Eggerthellaceae	G Enterorhabdus	unclassified G Enterorhabdus
SV2586	K Bacteria	P Firmicutes	C Clostridia	O Lachnospirales	F Lachnospiraceae	G Dorea	unclassified G Dorea
SV2587	K Bacteria	P Firmicutes	C Clostridia	O Oscillospirales	F Ruminococcaceae	G Incertae Sedis	unclassified G Incertae Sedis
SV2588	K Bacteria	P Bacteroidota	C Bacteroidia	O Bacteroidales	F Prevotellaceae	G Prevotellaceae UCG-001	unclassified G Prevotellaceae UCG-001
SV2589	K Bacteria	P Firmicutes	C Clostridia	O Oscillospirales	F Ruminococcaceae	unclassified F Ruminococcaceae	unclassified F Ruminococcaceae
SV2590	K Bacteria	P Firmicutes	C Bacilli	O RF39	unclassified O RF39	unclassified O RF39	unclassified O RF39
SV2591	K Bacteria	P Proteobacteria	C Alphaproteobacteria	O Rhodospirillales	unclassified O Rhodospirillales	unclassified O Rhodospirillales	unclassified O Rhodospirillales
SV2592	K Bacteria	P Firmicutes	C Bacilli	O RF39	unclassified O RF39	unclassified O RF39	unclassified O RF39
SV2593	K Bacteria	P Firmicutes	C Clostridia	O Clostridia vadinBB60 group	unclassified O Clostridia vadinBB60 group	unclassified O Clostridia vadinBB60 group	unclassified O Clostridia vadinBB60 group
SV2594	K_Bacteria	P_Bacteroidota	C_Bacteroidia	O_Bacteroidales	F_Muribaculaceae	unclassified_F_Muribaculaceae	unclassified_F_Muribaculaceae
SV2595	K Bacteria	P Firmicutes	C Clostridia	O Clostridia UCG-014	unclassified O Clostridia UCG-014	unclassified O Clostridia UCG-014	unclassified O Clostridia UCG-014
SV2596	K Bacteria	P Firmicutes	C Clostridia	O Clostridia UCG-014	unclassified O Clostridia UCG-014	unclassified O Clostridia UCG-014	unclassified O Clostridia UCG-014
SV2597	K Bacteria	P Firmicutes	C Clostridia	O Lachnospirales	F Lachnospiraceae	unclassified F Lachnospiraceae	unclassified F Lachnospiraceae
SV2598	K Bacteria	P Firmicutes	C Clostridia	O Peptostreptococcales-Tissierellales	F Peptostreptococcaceae	unclassified F Peptostreptococcaceae	unclassified F Peptostreptococcaceae
SV2599	K Bacteria	P Bacteroidota	C Bacteroidia	O Bacteroidales	F Rikenellaceae	G Alistipes	unclassified G Alistipes
SV2600	K_Bacteria	P_Firmicutes	C_Clostridia	O_Lachnospirales	F_Lachnospiraceae	G_Lachnospiraceae NK4A136 group	unclassified_G_Lachnospiraceae NK4A136 group
SV2601	K Bacteria	P Firmicutes	C Clostridia	O Lachnospirales	F Lachnospiraceae	G Lachnospiraceae NK4A136 group	unclassified G Lachnospiraceae NK4A136 group
SV2602	K Bacteria	P Firmicutes	C Clostridia	O Lachnospirales	F Lachnospiraceae	unclassified F Lachnospiraceae	unclassified F Lachnospiraceae
SV2603	K Bacteria	P Firmicutes	C Clostridia	O Peptostreptococcales-Tissierellales	F Peptostreptococcaceae	unclassified F Peptostreptococcaceae	unclassified F Peptostreptococcaceae
SV2604	K Bacteria	P Bacteroidota	C Bacteroidia	O Bacteroidales	F Muribaculaceae	unclassified F Muribaculaceae	unclassified F Muribaculaceae
SV2605	K Bacteria	P Firmicutes	C Bacilli	O Lactobacillales	F Lactobacillaceae	G HT002	unclassified G HT002
SV2606	K_Bacteria	P_Desulfobacterota	C_Desulfovibrionia	O_Desulfovibrionales	F_Desulfovibrionaceae	G_Bilophila	unclassified_G_Bilophila
SV2607	K Bacteria	P Actinobacteriota	C Coriobacteriia	O Coriobacteriales	F Eggerthellaceae	G Enterorhabdus	unclassified G Enterorhabdus
SV2608	K Bacteria	P Firmicutes	C Clostridia	O Oscillospirales	F Oscillospiraceae	G Flavonifractor	unclassified G Flavonifractor
SV2609	K Bacteria	P Firmicutes	C Clostridia	O Lachnospirales	F Lachnospiraceae	G [Eubacterium] oxidoreducens group	unclassified G [Eubacterium] oxidoreducens group
SV2610	K Bacteria	P Bacteroidota	C Bacteroidia	O Bacteroidales	F Prevotellaceae	G Prevotellaceae UCG-001	unclassified G Prevotellaceae UCG-001
SV2611	K Bacteria	P Firmicutes	C Clostridia	O Lachnospirales	F Lachnospiraceae	G [Eubacterium] oxidoreducens group	unclassified G [Eubacterium] oxidoreducens group
SV2612	K_Bacteria	P_Bacteroidota	C_Bacteroidia	O_Bacteroidales	F_Rikenellaceae	G_Alistipes	unclassified_G_Alistipes
SV2613	K Bacteria	P Firmicutes	C Clostridia	O Oscillospirales	F Oscillospiraceae	unclassified F Oscillospiraceae	unclassified F Oscillospiraceae
SV2614	K Bacteria	P Firmicutes	C Clostridia	O Oscillospirales	F [Eubacterium] coprostanoligenes group	unclassified F [Eubacterium] coprostanoligenes group	unclassified F [Eubacterium] coprostanoligenes group
SV2615	K Bacteria	P Firmicutes	C Clostridia	O Lachnospirales	F Defluviitaleaceae	G Defluviitaleaceae UCG-011	unclassified G Defluviitaleaceae UCG-011
SV2616	K Bacteria	P Firmicutes	C Clostridia	O Peptostreptococcales-Tissierellales	F Anaerovoracaceae	G [Eubacterium] brachy group	unclassified G [Eubacterium] brachy group
SV2617	K Bacteria	P Firmicutes	C Clostridia	O Clostridia vadinBB60 group	unclassified O Clostridia vadinBB60 group	unclassified O Clostridia vadinBB60 group	unclassified O Clostridia vadinBB60 group
SV2618	K Bacteria	P Bacteroidota	C Bacteroidia	O Bacteroidales	F Rikenellaceae	G Alistipes	unclassified G Alistipes
SV2619	K Bacteria	P Cyanobacteria	C Vampirivibrionia	O Gastranaerophilales	unclassified O Gastranaerophilales	unclassified O Gastranaerophilales	unclassified O Gastranaerophilales
SV2620	K Bacteria	P Firmicutes	C Clostridia	O Clostridiales	F Clostridiaceae	G Clostridium sensu stricto 1	unclassified G Clostridium sensu stricto 1

SV2621	K_Bacteria	P_Firmicutes	C_Clostridia	O_Lachnospirales	F_Lachnospiraceae	G_Lachnospiraceae NK4A136 group	unclassified_G_Lachnospiraceae NK4A136 group
SV2622	K_Bacteria	P_Firmicutes	C_Clostridia	O_Christensenellales	F_Christensenellaceae	G_Christensenellaceae R-7 group	unclassified_G_Christensenellaceae R-7 group
SV2623	K_Bacteria	P_Firmicutes	C_Clostridia	O_Oscillospirales	F_Oscillospiraceae	unclassified_F_Oscillospiraceae	unclassified_F_Oscillospiraceae
SV2624	K_Bacteria	P_Bacteroidota	C_Bacteroidia	O_Bacteroidales	F_Marinifilaceae	G_Odoribacter	unclassified_G_Odoribacter
SV2625	K_Bacteria	P_Bacteroidota	C_Bacteroidia	O_Bacteroidales	F_Bacteroidaceae	G_Bacteroides	unclassified_G_Bacteroides
SV2626	K_Bacteria	P_Firmicutes	C_Clostridia	O_Oscillospirales	F_Ruminococcaceae	G_Candidatus Soleaferrea	unclassified_G_Candidatus Soleaferrea
SV2627	K_Bacteria	P_Firmicutes	C_Clostridia	O_Lachnospirales	F_Lachnospiraceae	unclassified_F_Lachnospiraceae	unclassified_F_Lachnospiraceae
SV2628	K_Bacteria	P_Firmicutes	C_Clostridia	O_Lachnospirales	F_Lachnospiraceae	unclassified_F_Lachnospiraceae	unclassified_F_Lachnospiraceae
SV2629	K_Bacteria	P_Bacteroidota	C_Bacteroidia	O_Bacteroidales	F_Muribaculaceae	unclassified_F_Muribaculaceae	unclassified_F_Muribaculaceae
SV2630	K_Bacteria	P_Firmicutes	C_Bacilli	O_Staphylococcales	F_Staphylococcaceae	G_Staphylococcus	unclassified_G_Staphylococcus
SV2631	K_Bacteria	P_Firmicutes	C_Clostridia	O_Lachnospirales	F_Lachnospiraceae	G_Marvinbryantia	unclassified_G_Marvinbryantia
SV2632	K_Bacteria	P_Bacteroidota	C_Bacteroidia	O_Bacteroidales	F_Muribaculaceae	unclassified_F_Muribaculaceae	unclassified_F_Muribaculaceae
SV2633	K_Bacteria	P_Firmicutes	C_Clostridia	O_Lachnospirales	F_Lachnospiraceae	G_Roseburia	unclassified_G_Roseburia
SV2634	K_Bacteria	P_Firmicutes	C_Clostridia	O_Clostridia UCG-014	unclassified_O_Clostridia UCG-014	unclassified_O_Clostridia UCG-014	unclassified_O_Clostridia UCG-014
SV2635	K_Bacteria	P_Firmicutes	C_Bacilli	O_Lactobacillales	F_Lactobacillaceae	G_Ligilactobacillus	unclassified_G_Ligilactobacillus
SV2636	K_Bacteria	P_Firmicutes	C_Clostridia	O_Oscillospirales	F_Ruminococcaceae	unclassified_F_Ruminococcaceae	unclassified_F_Ruminococcaceae
SV2637	K_Bacteria	P_Cyanobacteria	C_Vampirivibrionia	O_Gastranaerophilales	unclassified_O_Gastranaerophilales	unclassified_O_Gastranaerophilales	unclassified_O_Gastranaerophilales
SV2638	K_Bacteria	P_Firmicutes	C_Bacilli	O_Lactobacillales	F_Lactobacillaceae	unclassified_F_Lactobacillaceae	unclassified_F_Lactobacillaceae
SV2639	K_Bacteria	P_Firmicutes	C_Clostridia	O_Lachnospirales	F_Lachnospiraceae	G_[Ruminococcus] gauvreauii group	unclassified_G_[Ruminococcus] gauvreauii group
SV2640	K_Bacteria	P_Firmicutes	C_Clostridia	O_Oscillospirales	F_Oscillospiraceae	unclassified_F_Oscillospiraceae	unclassified_F_Oscillospiraceae
SV2641	K_Bacteria	P_Bacteroidota	C_Bacteroidia	O_Bacteroidales	unclassified_O_Bacteroidales	unclassified_O_Bacteroidales	unclassified_O_Bacteroidales
SV2642	K_Bacteria	P_Bacteroidota	C_Bacteroidia	O_Bacteroidales	F_Muribaculaceae	unclassified_F_Muribaculaceae	unclassified_F_Muribaculaceae
SV2643	K_Bacteria	P_Firmicutes	C_Bacilli	O_Erysipelotrichales	F_Erysipelotrichaceae	G_Faecalibaculum	unclassified_G_Faecalibaculum
SV2644	K_Bacteria	P_Firmicutes	C_Clostridia	O_Oscillospirales	F_Ruminococcaceae	G_Ruminococcus	unclassified_G_Ruminococcus
SV2645	K_Bacteria	P_Firmicutes	C_Clostridia	O_Clostridiales	F_Clostridiaceae	G_Clostridium sensu stricto 1	unclassified_G_Clostridium sensu stricto 1
SV2646	K_Bacteria	P_Firmicutes	C_Bacilli	O_Erysipelotrichales	F_Erysipelotrichaceae	unclassified_F_Erysipelotrichaceae	unclassified_F_Erysipelotrichaceae
SV2647	K_Bacteria	P_Proteobacteria	C_Gammaproteobacteria	O_Enterobacterales	unclassified_O_Enterobacterales	unclassified_O_Enterobacterales	unclassified_O_Enterobacterales
SV2648	K_Bacteria	P_Firmicutes	C_Clostridia	O_Lachnospirales	F_Lachnospiraceae	G_ASF356	unclassified_G_ASF356
SV2649	K_Bacteria	P_Firmicutes	C_Clostridia	O_Clostridia UCG-014	unclassified_O_Clostridia UCG-014	unclassified_O_Clostridia UCG-014	unclassified_O_Clostridia UCG-014
SV2650	K_Bacteria	P_Firmicutes	C_Clostridia	O_Clostridia vadinBB60 group	unclassified_O_Clostridia vadinBB60 group	unclassified_O_Clostridia vadinBB60 group	unclassified_O_Clostridia vadinBB60 group
SV2651	K_Bacteria	P_Firmicutes	C_Bacilli	O_Lactobacillales	F_Lactobacillaceae	unclassified_F_Lactobacillaceae	unclassified_F_Lactobacillaceae
SV2652	K_Bacteria	P_Firmicutes	C_Bacilli	O_Erysipelotrichales	F_Erysipelotrichaceae	G_Turicibacter	unclassified_G_Turicibacter
SV2653	K_Bacteria	P_Firmicutes	C_Clostridia	O_Oscillospirales	F_[Eubacterium] coprostanoligenes group	unclassified_F_[Eubacterium] coprostanoligenes group	unclassified_F_[Eubacterium] coprostanoligenes group
SV2654	K_Bacteria	P_Bacteroidota	C_Bacteroidia	O_Bacteroidales	F_Muribaculaceae	unclassified_F_Muribaculaceae	unclassified_F_Muribaculaceae
SV2655	K_Bacteria	P_Firmicutes	C_Clostridia	O_Clostridia UCG-014	unclassified_O_Clostridia UCG-014	unclassified_O_Clostridia UCG-014	unclassified_O_Clostridia UCG-014
SV2656	K_Bacteria	P_Bacteroidota	C_Bacteroidia	O_Bacteroidales	F_Prevotellaceae	G_Prevotellaceae UCG-001	unclassified_G_Prevotellaceae UCG-001
SV2657	K_Bacteria	P_Firmicutes	C_Clostridia	O_Clostridia vadinBB60 group	unclassified_O_Clostridia vadinBB60 group	unclassified_O_Clostridia vadinBB60 group	unclassified_O_Clostridia vadinBB60 group
SV2658	K_Bacteria	P_Firmicutes	C_Clostridia	O_Lachnospirales	F_Lachnospiraceae	unclassified_F_Lachnospiraceae	unclassified_F_Lachnospiraceae
SV2659	K_Bacteria	P_Firmicutes	C_Clostridia	O_Peptostreptococcales-Tissierellales	F_Peptostreptococcaceae	unclassified_F_Peptostreptococcaceae	unclassified_F_Peptostreptococcaceae
SV2660	K_Bacteria	P_Firmicutes	C_Clostridia	O_Lachnospirales	F_Lachnospiraceae	G_[Eubacterium] oxidoreducens group	unclassified_G_[Eubacterium] oxidoreducens group
SV2661	K_Bacteria	P_Firmicutes	C_Clostridia	O_Oscillospirales	F_Ruminococcaceae	unclassified_F_Ruminococcaceae	unclassified_F_Ruminococcaceae
SV2662	K_Bacteria	P_Firmicutes	C_Clostridia	O_Oscillospirales	F_Oscillospiraceae	unclassified_F_Oscillospiraceae	unclassified_F_Oscillospiraceae
SV2663	K_Bacteria	P_Firmicutes	C_Clostridia	O_Lachnospirales	F_Lachnospiraceae	G_ASF356	unclassified_G_ASF356
SV2664	K_Bacteria	P_Firmicutes	C_Clostridia	O_Clostridia UCG-014	unclassified_O_Clostridia UCG-014	unclassified_O_Clostridia UCG-014	unclassified_O_Clostridia UCG-014
SV2665	K_Bacteria	P_Verrucomicrobiota	C_Verrucomicrobiae	O_Verrucomicrobiales	F_Akkermansiaceae	G_Akkermansia	unclassified_G_Akkermansia
SV2666	K_Bacteria	P_Verrucomicrobiota	C_Verrucomicrobiae	O_Verrucomicrobiales	F_Akkermansiaceae	G_Akkermansia	unclassified_G_Akkermansia
SV2667	K_Bacteria	P_Bacteroidota	C_Bacteroidia	O_Bacteroidales	F_Muribaculaceae	unclassified_F_Muribaculaceae	unclassified_F_Muribaculaceae
SV2668	K_Bacteria	P_Firmicutes	C_Clostridia	O_Oscillospirales	F_Ruminococcaceae	G_Pygmaiobacter	unclassified_G_Pygmaiobacter
SV2669	K_Bacteria	P_Firmicutes	C_Clostridia	O_Oscillospirales	F_Ruminococcaceae	unclassified_F_Ruminococcaceae	unclassified_F_Ruminococcaceae
SV2670	K_Bacteria	P_Firmicutes	C_Clostridia	O_Clostridia UCG-014	unclassified_O_Clostridia UCG-014	unclassified_O_Clostridia UCG-014	unclassified_O_Clostridia UCG-014
SV2671	K_Bacteria	P_Actinobacteriota	C_Actinobacteria	O_Corynebacteriales	F_Corynebacteriaceae	G_Corynebacterium	S_stationis
SV2672	K_Bacteria	P_Firmicutes	C_Clostridia	O_Oscillospirales	F_Ruminococcaceae	G_Ruminococcus	unclassified_G_Ruminococcus
SV2673	K_Bacteria	P_Firmicutes	C_Clostridia	O_Lachnospirales	F_Lachnospiraceae	G_Roseburia	unclassified_G_Roseburia
SV2674	K_Bacteria	P_Firmicutes	C_Clostridia	O_Oscillospirales	F_Ruminococcaceae	G_Ruminococcus	unclassified_G_Ruminococcus
SV2675	K_Bacteria	P_Firmicutes	C_Clostridia	O_Oscillospirales	F_Ruminococcaceae	G_Incertae Sedis	unclassified_G_Incertae Sedis
SV2676	K_Bacteria	P_Firmicutes	C_Bacilli	O_Lactobacillales	F_Lactobacillaceae	unclassified_F_Lactobacillaceae	unclassified_F_Lactobacillaceae
SV2677	K_Bacteria	P_Bacteroidota	C_Bacteroidia	O_Bacteroidales	F_Muribaculaceae	unclassified_F_Muribaculaceae	unclassified_F_Muribaculaceae
SV2678	K_Bacteria	P_Firmicutes	C_Bacilli	O_Lactobacillales	F_Lactobacillaceae	unclassified_F_Lactobacillaceae	unclassified_F_Lactobacillaceae
SV2679	K_Bacteria	P_Firmicutes	C_Clostridia	O_Oscillospirales	F_Butyricicoccaceae	G_Butyricicoccus	unclassified_G_Butyricicoccus
SV2680	K_Bacteria	P_Firmicutes	C_Clostridia	O_Peptostreptococcales-Tissierellales	F_Anaerovoracaceae	unclassified_F_Anaerovoracaceae	unclassified_F_Anaerovoracaceae
SV2681	K_Bacteria	P_Firmicutes	C_Clostridia	O_Clostridia vadinBB60 group	unclassified_O_Clostridia vadinBB60 group	unclassified_O_Clostridia vadinBB60 group	unclassified_O_Clostridia vadinBB60 group
SV2682	K_Bacteria	P_Firmicutes	C_Clostridia	O_Oscillospirales	F_Ruminococcaceae	G_Anaerotruncus	unclassified_G_Anaerotruncus
SV2683	K_Bacteria	P_Cyanobacteria	C_Vampirivibrionia	O_Gastranaerophilales	unclassified_O_Gastranaerophilales	unclassified_O_Gastranaerophilales	unclassified_O_Gastranaerophilales
SV2684	K_Bacteria	P_Firmicutes	C_Clostridia	O_Oscillospirales	F_Ruminococcaceae	G_Harryflintia	unclassified_G_Harryflintia
SV2685	K_Bacteria	P_Firmicutes	C_Clostridia	O_Clostridia vadinBB60 group	unclassified_O_Clostridia vadinBB60 group	unclassified_O_Clostridia vadinBB60 group	unclassified_O_Clostridia vadinBB60 group
SV2686	K_Bacteria	P_Firmicutes	C_Clostridia	O_Oscillospirales	F_Oscillospiraceae	G_UCG-005	unclassified_G_UCG-005
SV2687	K_Bacteria	P_Bacteroidota	C_Bacteroidia	O_Bacteroidales	F_Muribaculaceae	unclassified_F_Muribaculaceae	unclassified_F_Muribaculaceae
SV2688	K_Bacteria	P_Firmicutes	C_Clostridia	O_Lachnospirales	F_Lachnospiraceae	G_Roseburia	unclassified_G_Roseburia
SV2689	K_Bacteria	P_Cyanobacteria	C_Vampirivibrionia	O_Gastranaerophilales	unclassified_O_Gastranaerophilales	unclassified_O_Gastranaerophilales	unclassified_O_Gastranaerophilales
SV2690	K_Bacteria	P_Firmicutes	C_Clostridia	O_Oscillospirales	F_[Eubacterium] coprostanoligenes group	unclassified_F_[Eubacterium] coprostanoligenes group	unclassified_F_[Eubacterium] coprostanoligenes group
SV2691	K_Bacteria	P_Firmicutes	C_Clostridia	O_Oscillospirales	F_Oscillospiraceae	G_Colidextribacter	unclassified_G_Colidextribacter
SV2692	K_Bacteria	P_Firmicutes	C_Clostridia	O_Lachnospirales	F_Lachnospiraceae	G_Lachnoclostridium	unclassified_G_Lachnoclostridium
SV2693	K_Bacteria	P_Firmicutes	C_Bacilli	O_Lactobacillales	F_Lactobacillaceae	G_Lactobacillus	unclassified_G_Lactobacillus
SV2694	K_Bacteria	P_Firmicutes	C_Clostridia	O_Clostridiales	F_Clostridiaceae	G_Clostridium sensu stricto 1	unclassified_G_Clostridium sensu stricto 1
SV2695	K_Bacteria	P_Firmicutes	C_Clostridia	O_Oscillospirales	F_Ruminococcaceae	G_Ruminococcus	unclassified_G_Ruminococcus
SV2696	K_Bacteria	P_Firmicutes	C_Clostridia	O_Oscillospirales	F_Ruminococcaceae	unclassified_F_Ruminococcaceae	unclassified_F_Ruminococcaceae
SV2697	K_Bacteria	P_Firmicutes	C_Clostridia	O_Oscillospirales	F_Ruminococcaceae	unclassified_F_Ruminococcaceae	unclassified_F_Ruminococcaceae
SV2698	K_Bacteria	P_Firmicutes	C_Bacilli	O_Lactobacillales	F_Lactobacillaceae	unclassified_F_Lactobacillaceae	unclassified_F_Lactobacillaceae
SV2699	K_Bacteria	P_Firmicutes	C_Clostridia	O_Oscillospirales	F_Oscillospiraceae	G_Intestinimonas	unclassified_G_Intestinimonas
SV2700	K_Bacteria	P_Firmicutes	C_Clostridia	O_Peptostreptococcales-Tissierellales	F_Anaerovoracaceae	unclassified_F_Anaerovoracaceae	unclassified_F_Anaerovoracaceae
SV2701	K_Bacteria	P_Firmicutes	C_Bacilli	unclassified_C_Bacilli	unclassified_C_Bacilli	unclassified_C_Bacilli	unclassified_C_Bacilli
SV2702	K_Bacteria	P_Firmicutes	C_Clostridia	O_Clostridia UCG-014	unclassified_O_Clostridia UCG-014	unclassified_O_Clostridia UCG-014	unclassified_O_Clostridia UCG-014
SV2703	K_Bacteria	P_Firmicutes	C_Clostridia	O_Oscillospirales	F_[Eubacterium] coprostanoligenes group	unclassified_F_[Eubacterium] coprostanoligenes group	unclassified_F_[Eubacterium] coprostanoligenes group
SV2704	K_Bacteria	P_Bacteroidota	C_Bacteroidia	O_Bacteroidales	F_Rikenellaceae	G_Alistipes	unclassified_G_Alistipes
SV2705	K_Bacteria	P_Bacteroidota	C_Bacteroidia	O_Bacteroidales	F_Muribaculaceae	unclassified_F_Muribaculaceae	unclassified_F_Muribaculaceae
SV2706	K_Bacteria	P_Actinobacteriota	C_Coriobacteriia	O_Coriobacteriales	F_Eggerthellaceae	G_Enterorhabdus	unclassified_G_Enterorhabdus
SV2707	K_Bacteria	P_Firmicutes	C_Bacilli	O_RF39	unclassified_O_RF39	unclassified_O_RF39	unclassified_O_RF39
SV2708	K_Bacteria	P_Firmicutes	C_Clostridia	O_Oscillospirales	F_Butyricicoccaceae	G_Butyricicoccus	unclassified_G_Butyricicoccus
SV2709	K_Bacteria	P_Firmicutes	C_Clostridia	O_Peptostreptococcales-Tissierellales	F_Anaerovoracaceae	G_Family XIII AD3011 group	unclassified_G_Family XIII AD3011 group
SV2710	K_Bacteria	P_Firmicutes	C_Bacilli	O_Erysipelotrichales	F_Erysipelotrichaceae	G_Turicibacter	unclassified_G_Turicibacter
SV2711	K_Bacteria	P_Firmicutes	C_Clostridia	O_Oscillospirales	F_[Eubacterium] coprostanoligenes group	unclassified_F_[Eubacterium] coprostanoligenes group	unclassified_F_[Eubacterium] coprostanoligenes group
SV2712	K_Bacteria	P_Firmicutes	C_Clostridia	O_Lachnospirales	F_Lachnospiraceae	G_GCA-900066755	unclassified_G_GCA-900066755
SV2713	K_Bacteria	P_Firmicutes	C_Clostridia	O_Monoglobales	F_Monoglobaceae	G_Monoglobus	unclassified_G_Monoglobus
SV2714	K_Bacteria	P_Firmicutes	C_Clostridia	O_Eubacteriales	F_Anaerofustaceae	G_Anaerofustis	unclassified_G_Anaerofustis
SV2715	K_Bacteria	P_Firmicutes	C_Clostridia	O_Oscillospirales	F_Ruminococcaceae	unclassified_F_Ruminococcaceae	unclassified_F_Ruminococcaceae
SV2716	K_Bacteria	P_Bacteroidota	C_Bacteroidia	O_Bacteroidales	F_Muribaculaceae	unclassified_F_Muribaculaceae	unclassified_F_Muribaculaceae
SV2717	K_Bacteria	P_Deinococcota	C_Deinococci	O_Deinococcales	F_Deinococcaceae	G_Deinococcus	unclassified_G_Deinococcus
SV2718	K_Bacteria	P_Firmicutes	C_Clostridia	O_Oscillospirales	F_[Eubacterium] coprostanoligenes group	unclassified_F_[Eubacterium] coprostanoligenes group	unclassified_F_[Eubacterium] coprostanoligenes group
SV2719	K_Bacteria	P_Firmicutes	C_Clostridia	O_Oscillospirales	F_Ruminococcaceae	G_Ruminococcus	unclassified_G_Ruminococcus
SV2720	K_Bacteria	P_Firmicutes	C_Clostridia	O_Oscillospirales	F_Oscillospiraceae	G_NK4A214 group	unclassified_G_NK4A214 group
SV2721	K_Bacteria	P_Cyanobacteria	C_Vampirivibrionia	O_Gastranaerophilales	unclassified_O_Gastranaerophilales	unclassified_O_Gastranaerophilales	unclassified_O_Gastranaerophilales
SV2722	K_Bacteria	P_Bacteroidota	C_Bacteroidia	O_Bacteroidales	F_Prevotellaceae	G_Prevotellaceae UCG-001	unclassified_G_Prevotellaceae UCG-001
SV2723	K_Bacteria	P_Actinobacteriota	C_Coriobacteriia	O_Coriobacteriales	F_Eggerthellaceae	G_Enterorhabdus	unclassified_G_Enterorhabdus
SV2724	K_Bacteria	P_Firmicutes	C_Clostridia	O_Lachnospirales	F_Lachnospiraceae	G_Lachnospiraceae UCG-001	unclassified_G_Lachnospiraceae UCG-001
SV2725	K_Bacteria	P_Firmicutes	C_Clostridia	O_Lachnospirales	F_Lachnospiraceae	G_Acetatifactor	unclassified_G_Acetatifactor
SV2726	K_Bacteria	P_Actinobacteriota	C_Coriobacteriia	O_Coriobacteriales	F_Eggerthellaceae	G_Enterorhabdus	unclassified_G_Enterorhabdus
SV2727	K_Bacteria	P_Bacteroidota	C_Bacteroidia	O_Bacteroidales	F_Muribaculaceae	unclassified_F_Muribaculaceae	unclassified_F_Muribaculaceae
SV2728	K_Bacteria	P_Firmicutes	C_Clostridia	O_Oscillospirales	F_Ruminococcaceae	G_Anaerotruncus	unclassified_G_Anaerotruncus
SV2729	K_Bacteria	P_Firmicutes	C_Bacilli	O_Lactobacillales	F_Lactobacillaceae	unclassified_F_Lactobacillaceae	unclassified_F_Lactobacillaceae
SV2730	K_Bacteria	P_Firmicutes	C_Clostridia	O_Oscillospirales	F_Oscillospiraceae	unclassified_F_Oscillospiraceae	unclassified_F_Oscillospiraceae
SV2731	K_Bacteria	P_Firmicutes	C_Bacilli	O_Erysipelotrichales	F_Erysipelotrichaceae	G_[Clostridium] innocuum group	unclassified_G_[Clostridium] innocuum group
SV2732	K_Bacteria	P_Firmicutes	C_Clostridia	O_Oscillospirales	F_Ruminococcaceae	unclassified_F_Ruminococcaceae	unclassified_F_Ruminococcaceae
SV2733	K_Bacteria	P_Bacteroidota	C_Bacteroidia	O_Bacteroidales	F_Bacteroidaceae	G_Bacteroides	unclassified_G_Bacteroides
SV2734	K_Bacteria	P_Firmicutes	C_Bacilli	O_Lactobacillales	F_Lactobacillaceae	unclassified_F_Lactobacillaceae	unclassified_F_Lactobacillaceae
SV2735	K_Bacteria	P_Firmicutes	C_Clostridia	O_Oscillospirales	F_Ruminococcaceae	G_Anaerofilum	unclassified_G_Anaerofilum
SV2736	K_Bacteria	P_Bacteroidota	C_Bacteroidia	O_Bacteroidales	F_Prevotellaceae	G_Prevotellaceae UCG-001	unclassified_G_Prevotellaceae UCG-001
SV2737	K_Bacteria	P_Firmicutes	C_Clostridia	O_Clostridia vadinBB60 group	unclassified_O_Clostridia vadinBB60 group	unclassified_O_Clostridia vadinBB60 group	unclassified_O_Clostridia vadinBB60 group
SV2738	K_Bacteria	P_Firmicutes	C_Bacilli	O_Erysipelotrichales	F_Erysipelatoclostridiaceae	G_Candidatus Stoquefichus	unclassified_G_Candidatus Stoquefichus
SV2739	K_Bacteria	P_Firmicutes	C_Clostridia	O_Oscillospirales	F_Ruminococcaceae	G_Ruminococcus	unclassified_G_Ruminococcus
SV2740	K_Bacteria	P_Firmicutes	C_Clostridia	O_Peptostreptococcales-Tissierellales	F_Peptostreptococcaceae	unclassified_F_Peptostreptococcaceae	unclassified_F_Peptostreptococcaceae
SV2741	K_Bacteria	P_Actinobacteriota	C_Coriobacteriia	O_Coriobacteriales	F_Eggerthellaceae	G_Enterorhabdus	unclassified_G_Enterorhabdus
SV2742	K_Bacteria	P_Firmicutes	C_Bacilli	O_Lactobacillales	F_Lactobacillaceae	unclassified_F_Lactobacillaceae	unclassified_F_Lactobacillaceae
SV2743	K_Bacteria	P_Firmicutes	C_Clostridia	O_Clostridia vadinBB60 group	unclassified_O_Clostridia vadinBB60 group	unclassified_O_Clostridia vadinBB60 group	unclassified_O_Clostridia vadinBB60 group
SV2744	K_Bacteria	P_Firmicutes	C_Clostridia	O_Lachnospirales	F_Lachnospiraceae	G_Lachnospiraceae NK4A136 group	unclassified_G_Lachnospiraceae NK4A136 group
SV2745	K_Bacteria	P_Firmicutes	C_Bacilli	unclassified_C_Bacilli	unclassified_C_Bacilli	unclassified_C_Bacilli	unclassified_C_Bacilli
SV2746	K_Bacteria	P_Firmicutes	C_Clostridia	O_Clostridia vadinBB60 group	unclassified_O_Clostridia vadinBB60 group	unclassified_O_Clostridia vadinBB60 group	unclassified_O_Clostridia vadinBB60 group
SV2747	K_Bacteria	P_Firmicutes	C_Clostridia	O_Lachnospirales	F_Lachnospiraceae	G_Lachnospiraceae NC2004 group	unclassified_G_Lachnospiraceae NC2004 group
SV2748	K_Bacteria	P_Firmicutes	C_Clostridia	O_Oscillospirales	F_Ruminococcaceae	unclassified_F_Ruminococcaceae	unclassified_F_Ruminococcaceae
SV2749	K_Bacteria	P_Firmicutes	C_Clostridia	O_Peptostreptococcales-Tissierellales	F_Anaerovoracaceae	G_Family XIII AD3011 group	unclassified_G_Family XIII AD3011 group
SV2750	K_Bacteria	P_Firmicutes	C_Clostridia	O_Oscillospirales	F_Ruminococcaceae	unclassified_F_Ruminococcaceae	unclassified_F_Ruminococcaceae
SV2751	K_Bacteria	P_Desulfobacterota	C_Desulfovibrionia	O_Desulfovibrionales	F_Desulfovibrionaceae	G_Bilophila	unclassified_G_Bilophila
SV2752	K_Bacteria	P_Firmicutes	C_Clostridia	O_Peptostreptococcales-Tissierellales	F_Anaerovoracaceae	unclassified_F_Anaerovoracaceae	unclassified_F_Anaerovoracaceae
SV2753	K_Bacteria	P_Cyanobacteria	C_Vampirivibrionia	O_Gastranaerophilales	unclassified_O_Gastranaerophilales	unclassified_O_Gastranaerophilales	unclassified_O_Gastranaerophilales
SV2754	K_Bacteria	P_Cyanobacteria	C_Vampirivibrionia	O_Gastranaerophilales	unclassified_O_Gastranaerophilales	unclassified_O_Gastranaerophilales	unclassified_O_Gastranaerophilales
SV2755	K_Bacteria	P_Firmicutes	C_Clostridia	O_Peptostreptococcales-Tissierellales	F_Anaerovoracaceae	G_Family XIII AD3011 group	unclassified_G_Family XIII AD3011 group
SV2756	K_Bacteria	P_Firmicutes	C_Clostridia	O_Clostridia UCG-014	unclassified_O_Clostridia UCG-014	unclassified_O_Clostridia UCG-014	unclassified_O_Clostridia UCG-014
SV2757	K_Bacteria	P_Firmicutes	C_Clostridia	O_Oscillospirales	F_Oscillospiraceae	unclassified_F_Oscillospiraceae	unclassified_F_Oscillospiraceae
SV2758	K_Bacteria	P_Bacteroidota	C_Bacteroidia	O_Bacteroidales	unclassified_O_Bacteroidales	unclassified_O_Bacteroidales	unclassified_O_Bacteroidales

SV2759	K Bacteria	P Firmicutes	C Clostridia	O Peptostreptococcales-Tissierellales	F Peptostreptococcaceae	unclassified F Peptostreptococcaceae	unclassified F Peptostreptococcaceae
SV2760	K Bacteria	P Firmicutes	C Clostridia	O Lachnospirales	F Lachnospiraceae	unclassified F Lachnospiraceae	unclassified F Lachnospiraceae
SV2761	K Bacteria	P Actinobacteriota	C Actinobacteria	O Bifidobacteriales	F Bifidobacteriaceae	unclassified F Bifidobacteriaceae	unclassified F Bifidobacteriaceae
SV2762	K Bacteria	P Actinobacteriota	C Coriobacteriia	O Coriobacteriales	F Eggerthellaceae	G Enterorhabdus	unclassified G Enterorhabdus
SV2763	K Bacteria	P Firmicutes	C Clostridia	O Oscillospirales	F Ruminococcaceae	G Anaerotruncus	unclassified G Anaerotruncus
SV2764	K Bacteria	P Firmicutes	C Clostridia	O Oscillospirales	F Ruminococcaceae	unclassified F Ruminococcaceae	unclassified F Ruminococcaceae
SV2765	K Bacteria	P Firmicutes	unclassified P Firmicutes	unclassified P Firmicutes	unclassified P Firmicutes	unclassified P Firmicutes	unclassified P Firmicutes
SV2766	K Bacteria	P Firmicutes	C Clostridia	O Lachnospirales	F Lachnospiraceae	unclassified F Lachnospiraceae	unclassified F Lachnospiraceae
SV2767	K Bacteria	P Firmicutes	C Bacilli	O Erysipelotrichales	F Erysipelotrichaceae	G Turicibacter	unclassified G Turicibacter
SV2768	K Bacteria	P Firmicutes	C Bacilli	O Erysipelotrichales	F Erysipelotrichaceae	G Faecalibaculum	unclassified G Faecalibaculum
SV2769	K Bacteria	P Firmicutes	C Clostridia	O Oscillospirales	F Ruminococcaceae	G Ruminococcus	unclassified G Ruminococcus
SV2770	K Bacteria	P Firmicutes	C Clostridia	O Peptococcales	F Peptococcaceae	unclassified F Peptococcaceae	unclassified F Peptococcaceae
SV2771	K Bacteria	P Firmicutes	unclassified P Firmicutes	unclassified P Firmicutes	unclassified P Firmicutes	unclassified P Firmicutes	unclassified P Firmicutes
SV2772	K Bacteria	P Firmicutes	C Clostridia	O Oscillospirales	F Ruminococcaceae	G Ruminococcus	unclassified G Ruminococcus
SV2773	K Bacteria	P Firmicutes	C Clostridia	O Oscillospirales	F Ruminococcaceae	G Ruminococcus	unclassified G Ruminococcus
SV2774	K Bacteria	P Firmicutes	C Bacilli	O Lactobacillales	F Lactobacillaceae	unclassified F Lactobacillaceae	unclassified F Lactobacillaceae
SV2775	K Bacteria	P Firmicutes	C Clostridia	O Oscillospirales	F Ruminococcaceae	G Ruminococcus	unclassified G Ruminococcus
SV2776	K Bacteria	P Firmicutes	C Clostridia	O Oscillospirales	F Oscillospiraceae	G UCG-005	unclassified G UCG-005
SV2777	K Bacteria	P Firmicutes	C Bacilli	O Lactobacillales	F Lactobacillaceae	G HT002	unclassified G HT002
SV2778	K Bacteria	P Firmicutes	C Clostridia	O Clostridiales	F Clostridiaceae	unclassified F Clostridiaceae	unclassified F Clostridiaceae
SV2779	K Bacteria	P Desulfobacterota	C Desulfovibrionia	O Desulfovibrionales	F Desulfovibrionaceae	unclassified F Desulfovibrionaceae	unclassified F Desulfovibrionaceae
SV2780	K Bacteria	P Firmicutes	C Clostridia	O Peptostreptococcales-Tissierellales	F Peptostreptococcaceae	unclassified F Peptostreptococcaceae	unclassified F Peptostreptococcaceae
SV2781	K Bacteria	P Firmicutes	C Clostridia	O Clostridiales	F Clostridiaceae	G Clostridium sensu stricto 1	unclassified G Clostridium sensu stricto 1
SV2782	K Bacteria	P Bacteroidota	C Bacteroidia	O Bacteroidales	F Prevotellaceae	G Prevotellaceae UCG-001	unclassified G Prevotellaceae UCG-001
SV2783	K Bacteria	P Firmicutes	C Bacilli	O Erysipelotrichales	F Erysipelotrichaceae	G Faecalibaculum	unclassified G Faecalibaculum
SV2784	K Bacteria	P Firmicutes	C Clostridia	O Oscillospirales	F Oscillospiraceae	G Oscillibacter	unclassified G Oscillibacter
SV2785	K Bacteria	P Firmicutes	C Bacilli	O Lactobacillales	F Lactobacillaceae	unclassified F Lactobacillaceae	unclassified F Lactobacillaceae
SV2786	K Bacteria	P Firmicutes	C Bacilli	O Erysipelotrichales	F Erysipelatoclostridiaceae	G Candidatus Stoquefichus	unclassified G Candidatus Stoquefichus
SV2787	K Bacteria	P Firmicutes	C Clostridia	O Lachnospirales	F Lachnospiraceae	G Murimonas	unclassified G Murimonas
SV2788	K Bacteria	P Firmicutes	C Clostridia	O Peptostreptococcales-Tissierellales	F Anaerovoracaceae	G Anaerovorax	unclassified G Anaerovorax
SV2789	K Bacteria	P Actinobacteriota	C Coriobacteriia	O Coriobacteriales	F Eggerthellaceae	G Enterorhabdus	unclassified G Enterorhabdus
SV2790	K Bacteria	P Verrucomicrobiota	C Verrucomicrobiae	O Verrucomicrobiales	F Akkermansiaceae	G Akkermansia	unclassified G Akkermansia
SV2791	K Bacteria	P Firmicutes	C Clostridia	O Peptostreptococcales-Tissierellales	unclassified O Peptostreptococcales-Tissierellales	unclassified O Peptostreptococcales-Tissierellales	unclassified O Peptostreptococcales-Tissierellales
SV2792	K Bacteria	P Proteobacteria	C Alphaproteobacteria	O Rhodospirillales	unclassified O Rhodospirillales	unclassified O Rhodospirillales	unclassified O Rhodospirillales
SV2793	K Bacteria	P Firmicutes	C Clostridia	O Oscillospirales	F Ruminococcaceae	G Candidatus Soleaferrea	unclassified G Candidatus Soleaferrea
SV2794	K Bacteria	P Firmicutes	C Clostridia	unclassified C Clostridia	unclassified C Clostridia	unclassified C Clostridia	unclassified C Clostridia
SV2795	K Bacteria	P Firmicutes	C Clostridia	O Oscillospirales	F [Eubacterium] coprostanoligenes group	unclassified F [Eubacterium] coprostanoligenes grou	unclassified F [Eubacterium] coprostanoligenes group
SV2796	K Bacteria	P Firmicutes	C Clostridia	O Lachnospirales	F Lachnospiraceae	unclassified F Lachnospiraceae	unclassified F Lachnospiraceae
SV2797	K Bacteria	P Firmicutes	unclassified P Firmicutes	unclassified P Firmicutes	unclassified P Firmicutes	unclassified P Firmicutes	unclassified P Firmicutes
SV2798	K Bacteria	P Firmicutes	C Bacilli	O Lactobacillales	F Lactobacillaceae	G HT002	unclassified G HT002
SV2799	K Bacteria	P Firmicutes	C Clostridia	O Peptostreptococcales-Tissierellales	F Peptostreptococcaceae	unclassified F Peptostreptococcaceae	unclassified F Peptostreptococcaceae
SV2800	K Bacteria	P Firmicutes	C Clostridia	O Lachnospirales	F Lachnospiraceae	unclassified F Lachnospiraceae	unclassified F Lachnospiraceae
SV2801	K Bacteria	P Firmicutes	C Clostridia	O Eubacteriales	F Anaerofustaceae	G Anaerofustis	unclassified G Anaerofustis
SV2802	K Bacteria	P Bacteroidota	C Bacteroidia	O Bacteroidales	F Prevotellaceae	G Prevotellaceae UCG-001	unclassified G Prevotellaceae UCG-001
SV2803	K Bacteria	P Firmicutes	C Clostridia	O Oscillospirales	F Ruminococcaceae	G Ruminococcus	unclassified G Ruminococcus
SV2804	K Bacteria	P Firmicutes	C Clostridia	O Oscillospirales	F Ruminococcaceae	unclassified F Ruminococcaceae	unclassified F Ruminococcaceae
SV2805	K_Bacteria	P_Bacteroidota	C_Bacteroidia	O_Bacteroidales	F_Rikenellaceae	G_Alistipes	unclassified_G_Alistipes
SV2806	K Bacteria	P Firmicutes	C Clostridia	O Oscillospirales	F Ruminococcaceae	unclassified F Ruminococcaceae	unclassified F Ruminococcaceae
SV2807	K Bacteria	P Firmicutes	C Clostridia	O Oscillospirales	F Butyricicoccaceae	G UCG-009	unclassified G UCG-009
SV2808	K Bacteria	P Firmicutes	C Clostridia	O Monoglobales	F Monoglobaceae	G Monoglobus	unclassified G Monoglobus
SV2809	K Bacteria	P Firmicutes	C Clostridia	O Lachnospirales	F Lachnospiraceae	G Murimonas	unclassified G Murimonas
SV2810	K Bacteria	P Firmicutes	C Clostridia	O Lachnospirales	F Lachnospiraceae	G GCA-900066575	unclassified G GCA-900066575
SV2811	K_Bacteria	P_Firmicutes	C_Bacilli	O_Lactobacillales	F_Lactobacillaceae	unclassified_F_Lactobacillaceae	unclassified_F_Lactobacillaceae
SV2812	K Bacteria	P Proteobacteria	C Gammaproteobacteria	O Enterobacterales	F Enterobacteriaceae	G Enterobacter	unclassified G Enterobacter
SV2813	K Bacteria	P Firmicutes	C Clostridia	O Christensenellales	F Christensenellaceae	unclassified F Christensenellaceae	unclassified F Christensenellaceae
SV2814	K Bacteria	P Firmicutes	C Bacilli	unclassified C Bacilli	unclassified C Bacilli	unclassified C Bacilli	unclassified C Bacilli
SV2815	K Bacteria	P Bacteroidota	C Bacteroidia	O Bacteroidales	F Muribaculaceae	unclassified F Muribaculaceae	unclassified F Muribaculaceae
SV2816	K Bacteria	P Firmicutes	C Clostridia	O Clostridia UCG-014	unclassified O Clostridia UCG-014	unclassified O Clostridia UCG-014	unclassified O Clostridia UCG-014
SV2817	K_Bacteria	P_Proteobacteria	C_Gammaproteobacteria	O_Burkholderiales	F_Sutterellaceae	G_Parasutterella	unclassified_G_Parasutterella
SV2818	K Bacteria	P Firmicutes	C Clostridia	O Oscillospirales	F [Eubacterium] coprostanoligenes group	unclassified F [Eubacterium] coprostanoligenes grou	unclassified F [Eubacterium] coprostanoligenes group
SV2819	K Bacteria	P Bacteroidota	C Bacteroidia	O Bacteroidales	F Muribaculaceae	unclassified F Muribaculaceae	unclassified F Muribaculaceae
SV2820	K Bacteria	P Firmicutes	C Bacilli	O Erysipelotrichales	F Erysipelotrichaceae	G Turicibacter	unclassified G Turicibacter
SV2821	K Bacteria	P Firmicutes	C Clostridia	O Lachnospirales	F Lachnospiraceae	G Murimonas	unclassified G Murimonas
SV2822	K Bacteria	P Firmicutes	C Clostridia	O Lachnospirales	F Lachnospiraceae	unclassified F Lachnospiraceae	unclassified F Lachnospiraceae
SV2823	K_Bacteria	P_Firmicutes	C_Clostridia	O_Lachnospirales	F_Lachnospiraceae	G_ASF356	unclassified_G_ASF356
SV2824	K Bacteria	P Firmicutes	C Clostridia	unclassified C Clostridia	unclassified C Clostridia	unclassified C Clostridia	unclassified C Clostridia
SV2825	K Bacteria	P Firmicutes	C Bacilli	O Lactobacillales	F Lactobacillaceae	unclassified F Lactobacillaceae	unclassified F Lactobacillaceae
SV2826	K Bacteria	P Firmicutes	C Bacilli	O Erysipelotrichales	F Erysipelotrichaceae	G Faecalibaculum	unclassified G Faecalibaculum
SV2827	K Bacteria	P Firmicutes	C Clostridia	O Oscillospirales	F [Eubacterium] coprostanoligenes group	unclassified F [Eubacterium] coprostanoligenes grou	unclassified F [Eubacterium] coprostanoligenes group
SV2828	K Bacteria	P Firmicutes	C Clostridia	O Peptostreptococcales-Tissierellales	F Peptostreptococcaceae	G Romboutsia	unclassified G Romboutsia
SV2829	K_Bacteria	P_Firmicutes	C_Bacilli	unclassified_C_Bacilli	unclassified_C_Bacilli	unclassified_C_Bacilli	unclassified_C_Bacilli
SV2830	K Bacteria	P Firmicutes	C Bacilli	O Lactobacillales	F Lactobacillaceae	G HT002	unclassified G HT002
SV2831	K Bacteria	P Firmicutes	C Clostridia	O Lachnospirales	F Lachnospiraceae	G Lachnospiraceae NK4A136 group	unclassified G Lachnospiraceae NK4A136 group
SV2832	K Bacteria	P Firmicutes	C Clostridia	O Lachnospirales	F Lachnospiraceae	G Lachnospiraceae NK4A136 group	unclassified G Lachnospiraceae NK4A136 group
SV2833	K Bacteria	P Firmicutes	C Clostridia	O Oscillospirales	F Oscillospiraceae	G UCG-005	unclassified G UCG-005
SV2834	K Bacteria	P Firmicutes	C Clostridia	O Peptostreptococcales-Tissierellales	unclassified O Peptostreptococcales-Tissierellales	unclassified O Peptostreptococcales-Tissierellales	unclassified O Peptostreptococcales-Tissierellales
SV2835	K Bacteria	P Firmicutes	C Clostridia	O Peptostreptococcales-Tissierellales	F Peptostreptococcaceae	unclassified F Peptostreptococcaceae	unclassified F Peptostreptococcaceae
SV2836	K Bacteria	P Firmicutes	C Bacilli	O Erysipelotrichales	F Erysipelotrichaceae	unclassified F Erysipelotrichaceae	unclassified F Erysipelotrichaceae
SV2837	K Bacteria	P Firmicutes	C Bacilli	O Lactobacillales	F Lactobacillaceae	G Holzapfelia	unclassified G Holzapfelia
SV2838	K Bacteria	P Firmicutes	C Bacilli	O Erysipelotrichales	F Erysipelatoclostridiaceae	G Erysipelatoclostridium	unclassified G Erysipelatoclostridium
SV2839	K Bacteria	P Firmicutes	C Clostridia	O Oscillospirales	F Oscillospiraceae	G Intestinimonas	unclassified G Intestinimonas
SV2840	K Bacteria	P Bacteroidota	C Bacteroidia	O Bacteroidales	F Rikenellaceae	G Alistipes	unclassified G Alistipes
SV2841	K Bacteria	P Firmicutes	C Clostridia	O Oscillospirales	F Oscillospiraceae	unclassified F Oscillospiraceae	unclassified F Oscillospiraceae
SV2842	K Bacteria	P Firmicutes	C Clostridia	O Oscillospirales	F Ruminococcaceae	unclassified F Ruminococcaceae	unclassified F Ruminococcaceae
SV2843	K Bacteria	P Firmicutes	C Clostridia	O Oscillospirales	F Ruminococcaceae	G Incertae Sedis	unclassified G Incertae Sedis
SV2844	K Bacteria	P Firmicutes	C Clostridia	O Oscillospirales	F Butyricicoccaceae	G Butyricicoccus	unclassified G Butyricicoccus
SV2845	K Bacteria	P Desulfobacterota	C Desulfovibrionia	O Desulfovibrionales	F Desulfovibrionaceae	G Bilophila	unclassified G Bilophila
SV2846	K Bacteria	P Firmicutes	C Clostridia	O Peptostreptococcales-Tissierellales	F Peptostreptococcaceae	unclassified F Peptostreptococcaceae	unclassified F Peptostreptococcaceae
SV2847	K Bacteria	P Bacteroidota	C Bacteroidia	O Bacteroidales	F Rikenellaceae	G Alistipes	unclassified G Alistipes
SV2848	K Bacteria	P Firmicutes	C Bacilli	O Erysipelotrichales	F Erysipelotrichaceae	G Faecalibaculum	unclassified G Faecalibaculum
SV2849	K Bacteria	P Firmicutes	C Clostridia	O Oscillospirales	F Oscillospiraceae	G Colidextribacter	unclassified G Colidextribacter
SV2850	K Bacteria	P Firmicutes	C Clostridia	O Oscillospirales	F [Eubacterium] coprostanoligenes group	unclassified F [Eubacterium] coprostanoligenes grou	unclassified F [Eubacterium] coprostanoligenes group
SV2851	K Bacteria	P Firmicutes	C Clostridia	O Peptostreptococcales-Tissierellales	F Peptostreptococcaceae	unclassified F Peptostreptococcaceae	unclassified F Peptostreptococcaceae
SV2852	K Bacteria	P Firmicutes	C Clostridia	O Oscillospirales	F Butyricicoccaceae	G Butyricicoccus	unclassified G Butyricicoccus
SV2853	K Bacteria	P Firmicutes	C Bacilli	unclassified C Bacilli	unclassified C Bacilli	unclassified C Bacilli	unclassified C Bacilli
SV2854	K Bacteria	P Bacteroidota	C Bacteroidia	O Bacteroidales	F Rikenellaceae	G Alistipes	unclassified G Alistipes
SV2855	K Bacteria	P Firmicutes	C Clostridia	O Lachnospirales	F Lachnospiraceae	G Lachnospiraceae UCG-006	unclassified G Lachnospiraceae UCG-006
SV2856	K Bacteria	P Firmicutes	C Clostridia	O Peptostreptococcales-Tissierellales	F Peptostreptococcaceae	unclassified F Peptostreptococcaceae	unclassified F Peptostreptococcaceae
SV2857	K Bacteria	P Firmicutes	C Clostridia	O Clostridia UCG-014	unclassified O Clostridia UCG-014	unclassified O Clostridia UCG-014	unclassified O Clostridia UCG-014
SV2858	K Bacteria	P Bacteroidota	C Bacteroidia	O Bacteroidales	F Muribaculaceae	unclassified F Muribaculaceae	unclassified F Muribaculaceae
SV2859	K Bacteria	P Bacteroidota	C Bacteroidia	O Bacteroidales	F Muribaculaceae	unclassified F Muribaculaceae	unclassified F Muribaculaceae
SV2860	K Bacteria	P Firmicutes	C Clostridia	O Oscillospirales	F Oscillospiraceae	G UCG-007	unclassified G UCG-007
SV2861	K Bacteria	P Firmicutes	C Bacilli	O Erysipelotrichales	F Erysipelatoclostridiaceae	G Erysipelatoclostridium	unclassified G Erysipelatoclostridium
SV2862	K Bacteria	P Firmicutes	C Clostridia	O Clostridia vadinBB60 group	unclassified O Clostridia vadinBB60 group	unclassified O Clostridia vadinBB60 group	unclassified O Clostridia vadinBB60 group
SV2863	K Bacteria	P Bacteroidota	C Bacteroidia	O Bacteroidales	F Muribaculaceae	G Muribaculum	unclassified G Muribaculum
SV2864	K Bacteria	P Firmicutes	C Clostridia	O Lachnospirales	F Lachnospiraceae	G Murimonas	unclassified G Murimonas
SV2865	K Bacteria	P Actinobacteriota	C Actinobacteria	O Corynebacteriales	F Corynebacteriaceae	G Corynebacterium	unclassified G Corynebacterium
SV2866	K Bacteria	P Firmicutes	C Bacilli	unclassified C Bacilli	unclassified C Bacilli	unclassified C Bacilli	unclassified C Bacilli
SV2867	K Bacteria	P Firmicutes	C Clostridia	O Peptostreptococcales-Tissierellales	F Peptostreptococcaceae	unclassified F Peptostreptococcaceae	unclassified F Peptostreptococcaceae
SV2868	K Bacteria	P Firmicutes	C Bacilli	O Lactobacillales	F Lactobacillaceae	unclassified F Lactobacillaceae	unclassified F Lactobacillaceae
SV2869	K Bacteria	P Bacteroidota	C Bacteroidia	O Bacteroidales	F Muribaculaceae	unclassified F Muribaculaceae	unclassified F Muribaculaceae
SV2870	K_Bacteria	P_Bacteroidota	C_Bacteroidia	O_Bacteroidales	F_Muribaculaceae	unclassified_F_Muribaculaceae	unclassified_F_Muribaculaceae
SV2871	K Bacteria	P Firmicutes	C Clostridia	O Lachnospirales	F Lachnospiraceae	G Murimonas	unclassified G Murimonas
SV2872	K Bacteria	P Firmicutes	C Clostridia	O Monoglobales	F Monoglobaceae	G Monoglobus	unclassified G Monoglobus
SV2873	K Bacteria	P Bacteroidota	C Bacteroidia	O Bacteroidales	F Prevotellaceae	G Prevotellaceae UCG-001	unclassified G Prevotellaceae UCG-001
SV2874	K Bacteria	P Bacteroidota	C Bacteroidia	O Bacteroidales	F Muribaculaceae	unclassified F Muribaculaceae	unclassified F Muribaculaceae
SV2875	K Bacteria	P Firmicutes	C Clostridia	O Lachnospirales	F Lachnospiraceae	unclassified F Lachnospiraceae	unclassified F Lachnospiraceae
SV2876	K_Bacteria	P_Firmicutes	C_Bacilli	O_Lactobacillales	F_Lactobacillaceae	unclassified_F_Lactobacillaceae	unclassified_F_Lactobacillaceae
SV2877	K Bacteria	P Firmicutes	C Bacilli	O Lactobacillales	F Lactobacillaceae	unclassified F Lactobacillaceae	unclassified F Lactobacillaceae
SV2878	K Bacteria	P Firmicutes	unclassified P Firmicutes	unclassified P Firmicutes	unclassified P Firmicutes	unclassified P Firmicutes	unclassified P Firmicutes
SV2879	K Bacteria	P Firmicutes	C Clostridia	O Eubacteriales	F Anaerofustaceae	G Anaerofustis	unclassified G Anaerofustis
SV2880	K Bacteria	P Firmicutes	C Clostridia	O Lachnospirales	F Lachnospiraceae	unclassified F Lachnospiraceae	unclassified F Lachnospiraceae
SV2881	K Bacteria	P Firmicutes	C Clostridia	O Peptostreptococcales-Tissierellales	F Peptostreptococcaceae	unclassified F Peptostreptococcaceae	unclassified F Peptostreptococcaceae
SV2882	K_Bacteria	P_Firmicutes	C_Bacilli	O_RF39	unclassified_O_RF39	unclassified_O_RF39	unclassified_O_RF39
SV2883	K Bacteria	P Firmicutes	C Clostridia	O Peptostreptococcales-Tissierellales	F Peptostreptococcaceae	unclassified F Peptostreptococcaceae	unclassified F Peptostreptococcaceae
SV2884	K Bacteria	P Bacteroidota	C Bacteroidia	O Bacteroidales	F Muribaculaceae	unclassified F Muribaculaceae	unclassified F Muribaculaceae
SV2885	K Bacteria	P Firmicutes	C Clostridia	O Oscillospirales	F Ruminococcaceae	unclassified F Ruminococcaceae	unclassified F Ruminococcaceae
SV2886	K Bacteria	P Bacteroidota	C Bacteroidia	O Bacteroidales	F Marinifilaceae	G Odoribacter	unclassified G Odoribacter
SV2887	K Bacteria	P Firmicutes	C Bacilli	O Lactobacillales	F Lactobacillaceae	unclassified F Lactobacillaceae	unclassified F Lactobacillaceae
SV2888	K_Bacteria	P_Firmicutes	C_Clostridia	O_Oscillospirales	F_[Eubacterium] coprostanoligenes group	unclassified_F_[Eubacterium] coprostanoligenes grou	unclassified_F_[Eubacterium] coprostanoligenes group
SV2889	K Bacteria	P Firmicutes	C Clostridia	O Lachnospirales	F Lachnospiraceae	G Murimonas	unclassified G Murimonas
SV2890	K Bacteria	P Firmicutes	C Clostridia	O Lachnospirales	F Lachnospiraceae	G Murimonas	unclassified G Murimonas
SV2891	K Bacteria	P Firmicutes	C Clostridia	O Oscillospirales	F Ruminococcaceae	G Ruminococcus	unclassified G Ruminococcus
SV2892	K Bacteria	P Firmicutes	C Clostridia	O Lachnospirales	F Lachnospiraceae	G Lachnospiraceae UCG-004	unclassified G Lachnospiraceae UCG-004
SV2893	K Bacteria	P Firmicutes	C Bacilli	O Lactobacillales	F Lactobacillaceae	G HT002	unclassified G HT002
SV2894	K Bacteria	P Firmicutes	C Clostridia	O Oscillospirales	F Ruminococcaceae	G Ruminococcus	unclassified G Ruminococcus
SV2895	K Bacteria	P Firmicutes	C Clostridia	O Oscillospirales	F Ruminococcaceae	G Ruminococcus	unclassified G Ruminococcus
SV2896	K Bacteria	P Firmicutes	C Clostridia	O Oscillospirales	F Ruminococcaceae	G Ruminococcus	unclassified G Ruminococcus

SV2897	K_Bacteria	P_Firmicutes	C_Clostridia	O_Peptostreptococcales-Tissierellales	F_Peptostreptococcaceae	unclassified_F_Peptostreptococcaceae	unclassified_F_Peptostreptococcaceae
SV2898	K_Bacteria	P_Firmicutes	unclassified_P_Firmicutes	unclassified_P_Firmicutes	unclassified_P_Firmicutes	unclassified_P_Firmicutes	unclassified_P_Firmicutes
SV2899	K_Bacteria	P_Firmicutes	C_Clostridia	O_Clostridia UCG-014	unclassified_O_Clostridia UCG-014	unclassified_O_Clostridia UCG-014	unclassified_O_Clostridia UCG-014
SV2900	K_Bacteria	P_Firmicutes	C_Clostridia	O_Lachnospirales	F_Lachnospiraceae	unclassified_F_Lachnospiraceae	unclassified_F_Lachnospiraceae
SV2901	K_Bacteria	P_Firmicutes	C_Clostridia	O_Peptostreptococcales-Tissierellales	F_Anaerovoracaceae	G_[Eubacterium] brachy group	unclassified_G_[Eubacterium] brachy group
SV2902	K_Bacteria	P_Firmicutes	C_Clostridia	O_Lachnospirales	F_Lachnospiraceae	unclassified_F_Lachnospiraceae	unclassified_F_Lachnospiraceae
SV2903	K_Bacteria	P_Firmicutes	C_Clostridia	O_Peptostreptococcales-Tissierellales	F_Peptostreptococcaceae	unclassified_F_Peptostreptococcaceae	unclassified_F_Peptostreptococcaceae
SV2904	K_Bacteria	P_Firmicutes	C_Bacilli	O_Erysipelotrichales	F_Erysipelotrichaceae	G_Turicibacter	unclassified_G_Turicibacter
SV2905	K_Bacteria	P_Firmicutes	C_Bacilli	O_Lactobacillales	unclassified_O_Lactobacillales	unclassified_O_Lactobacillales	unclassified_O_Lactobacillales
SV2906	K_Bacteria	P_Firmicutes	C_Clostridia	O_Lachnospirales	F_Lachnospiraceae	unclassified_F_Lachnospiraceae	unclassified_F_Lachnospiraceae
SV2907	K_Bacteria	P_Bacteroidota	C_Bacteroidia	O_Bacteroidales	F_Muribaculaceae	unclassified_F_Muribaculaceae	unclassified_F_Muribaculaceae
SV2908	K_Bacteria	P_Firmicutes	C_Clostridia	O_Lachnospirales	F_Lachnospiraceae	unclassified_F_Lachnospiraceae	unclassified_F_Lachnospiraceae
SV2909	K_Bacteria	P_Firmicutes	C_Clostridia	O_Peptostreptococcales-Tissierellales	F_Anaerovoracaceae	G_[Eubacterium] nodatum group	unclassified_G_[Eubacterium] nodatum group
SV2910	K_Bacteria	P_Firmicutes	C_Clostridia	O_Oscillospirales	F_Ruminococcaceae	G_Ruminococcus	unclassified_G_Ruminococcus
SV2911	K_Bacteria	P_Firmicutes	C_Clostridia	O_Lachnospirales	F_Lachnospiraceae	unclassified_F_Lachnospiraceae	unclassified_F_Lachnospiraceae
SV2912	K_Bacteria	P_Firmicutes	C_Clostridia	O_Lachnospirales	F_Lachnospiraceae	unclassified_F_Lachnospiraceae	unclassified_F_Lachnospiraceae
SV2913	K_Bacteria	P_Firmicutes	C_Bacilli	O_Lactobacillales	F_Lactobacillaceae	G_HT002	unclassified_G_HT002
SV2914	K_Bacteria	P_Firmicutes	C_Bacilli	O_Erysipelotrichales	F_Erysipelotrichaceae	G_Faecalibaculum	unclassified_G_Faecalibaculum
SV2915	K_Bacteria	P_Firmicutes	C_Clostridia	O_Lachnospirales	F_Lachnospiraceae	unclassified_F_Lachnospiraceae	unclassified_F_Lachnospiraceae
SV2916	K_Bacteria	P_Firmicutes	C_Bacilli	O_Lactobacillales	unclassified_O_Lactobacillales	unclassified_O_Lactobacillales	unclassified_O_Lactobacillales
SV2917	K_Bacteria	P_Firmicutes	C_Bacilli	O_Lactobacillales	F_Lactobacillaceae	G_HT002	unclassified_G_HT002
SV2918	K_Bacteria	P_Firmicutes	C_Clostridia	O_Oscillospirales	F_Ruminococcaceae	unclassified_F_Ruminococcaceae	unclassified_F_Ruminococcaceae
SV2919	K_Bacteria	P_Firmicutes	C_Clostridia	O_Lachnospirales	F_Lachnospiraceae	G_[Eubacterium] oxidoreducens group	unclassified_G_[Eubacterium] oxidoreducens group
SV2920	K_Bacteria	P_Firmicutes	C_Clostridia	O_Lachnospirales	F_Lachnospiraceae	G_Lachnospiraceae NC2004 group	unclassified_G_Lachnospiraceae NC2004 group
SV2921	K_Bacteria	P_Bacteroidota	C_Bacteroidia	O_Bacteroidales	F_Bacteroidaceae	G_Bacteroides	unclassified_G_Bacteroides
SV2922	K_Bacteria	P_Bacteroidota	C_Bacteroidia	O_Bacteroidales	F_Bacteroidaceae	G_Bacteroides	unclassified_G_Bacteroides
SV2923	K_Bacteria	P_Bacteroidota	C_Bacteroidia	O_Bacteroidales	F_Prevotellaceae	G_Prevotellaceae UCG-001	unclassified_G_Prevotellaceae UCG-001
SV2924	K_Bacteria	P_Firmicutes	C_Clostridia	O_Peptostreptococcales-Tissierellales	F_Peptostreptococcaceae	unclassified_F_Peptostreptococcaceae	unclassified_F_Peptostreptococcaceae
SV2925	K_Bacteria	P_Firmicutes	C_Bacilli	O_Lactobacillales	F_Lactobacillaceae	unclassified_F_Lactobacillaceae	unclassified_F_Lactobacillaceae
SV2926	K_Bacteria	P_Bacteroidota	C_Bacteroidia	O_Bacteroidales	F_Muribaculaceae	unclassified_F_Muribaculaceae	unclassified_F_Muribaculaceae
SV2927	K_Bacteria	P_Firmicutes	C_Bacilli	O_Erysipelotrichales	F_Erysipelotrichaceae	G_Holdemania	unclassified_G_Holdemania
SV2928	K_Bacteria	P_Firmicutes	C_Bacilli	O_Erysipelotrichales	F_Erysipelotrichaceae	G_Turicibacter	unclassified_G_Turicibacter
SV2929	K_Bacteria	P_Bacteroidota	C_Bacteroidia	O_Bacteroidales	F_Muribaculaceae	unclassified_F_Muribaculaceae	unclassified_F_Muribaculaceae
SV2930	K_Bacteria	P_Bacteroidota	C_Bacteroidia	O_Bacteroidales	F_Marinifilaceae	G_Odoribacter	unclassified_G_Odoribacter
SV2931	K_Bacteria	P_Bacteroidota	C_Bacteroidia	O_Bacteroidales	F_Muribaculaceae	unclassified_F_Muribaculaceae	unclassified_F_Muribaculaceae
SV2932	K_Bacteria	P_Firmicutes	C_Clostridia	O_Peptostreptococcales-Tissierellales	F_Peptostreptococcaceae	unclassified_F_Peptostreptococcaceae	unclassified_F_Peptostreptococcaceae
SV2933	K_Bacteria	P_Firmicutes	C_Clostridia	O_Lachnospirales	F_Lachnospiraceae	unclassified_F_Lachnospiraceae	unclassified_F_Lachnospiraceae
SV2934	K_Bacteria	P_Bacteroidota	C_Bacteroidia	O_Bacteroidales	F_Muribaculaceae	unclassified_F_Muribaculaceae	unclassified_F_Muribaculaceae
SV2935	K_Bacteria	P_Firmicutes	C_Clostridia	O_Peptostreptococcales-Tissierellales	F_Peptostreptococcaceae	unclassified_F_Peptostreptococcaceae	unclassified_F_Peptostreptococcaceae
SV2936	K_Bacteria	P_Cyanobacteria	C_Vampirivibrionia	O_Gastranaerophilales	unclassified_O_Gastranaerophilales	unclassified_O_Gastranaerophilales	unclassified_O_Gastranaerophilales
SV2937	K_Bacteria	P_Firmicutes	C_Clostridia	O_Peptostreptococcales-Tissierellales	unclassified_O_Peptostreptococcales-Tissierellales	unclassified_O_Peptostreptococcales-Tissierellales	unclassified_O_Peptostreptococcales-Tissierellales
SV2938	K_Bacteria	P_Firmicutes	C_Bacilli	O_Erysipelotrichales	F_Erysipelatoclostridiaceae	G_Erysipelatoclostridium	unclassified_G_Erysipelatoclostridium
SV2939	K_Bacteria	P_Bacteroidota	C_Bacteroidia	O_Bacteroidales	F_Rikenellaceae	unclassified_F_Rikenellaceae	unclassified_F_Rikenellaceae
SV2940	K_Bacteria	P_Bacteroidota	C_Bacteroidia	O_Bacteroidales	F_Prevotellaceae	G_Prevotellaceae UCG-001	unclassified_G_Prevotellaceae UCG-001
SV2941	K_Bacteria	P_Firmicutes	C_Clostridia	O_Peptostreptococcales-Tissierellales	F_Peptostreptococcaceae	unclassified_F_Peptostreptococcaceae	unclassified_F_Peptostreptococcaceae
SV2942	K_Bacteria	P_Firmicutes	C_Bacilli	O_RF39	unclassified_O_RF39	unclassified_O_RF39	unclassified_O_RF39
SV2943	K_Bacteria	P_Bacteroidota	C_Bacteroidia	O_Bacteroidales	F_Prevotellaceae	G_Prevotellaceae UCG-001	unclassified_G_Prevotellaceae UCG-001
SV2944	K_Bacteria	P_Bacteroidota	C_Bacteroidia	O_Bacteroidales	F_Prevotellaceae	G_Prevotellaceae UCG-001	unclassified_G_Prevotellaceae UCG-001
SV2945	K_Bacteria	P_Firmicutes	C_Bacilli	O_Lactobacillales	unclassified_O_Lactobacillales	unclassified_O_Lactobacillales	unclassified_O_Lactobacillales
SV2946	K_Bacteria	P_Firmicutes	C_Bacilli	unclassified_C_Bacilli	unclassified_C_Bacilli	unclassified_C_Bacilli	unclassified_C_Bacilli
SV2947	K_Bacteria	P_Firmicutes	C_Bacilli	O_Lactobacillales	F_Lactobacillaceae	unclassified_F_Lactobacillaceae	unclassified_F_Lactobacillaceae
SV2948	K_Bacteria	P_Firmicutes	C_Bacilli	O_Lactobacillales	F_Lactobacillaceae	unclassified_F_Lactobacillaceae	unclassified_F_Lactobacillaceae
SV2949	K_Bacteria	P_Firmicutes	C_Bacilli	O_Lactobacillales	F_Lactobacillaceae	unclassified_F_Lactobacillaceae	unclassified_F_Lactobacillaceae
SV2950	K_Bacteria	P_Firmicutes	C_Clostridia	O_Lachnospirales	F_Lachnospiraceae	unclassified_F_Lachnospiraceae	unclassified_F_Lachnospiraceae
SV2951	K_Bacteria	P_Firmicutes	C_Bacilli	O_Lactobacillales	F_Lactobacillaceae	unclassified_F_Lactobacillaceae	unclassified_F_Lactobacillaceae
SV2952	K_Bacteria	P_Firmicutes	C_Bacilli	unclassified_C_Bacilli	unclassified_C_Bacilli	unclassified_C_Bacilli	unclassified_C_Bacilli
SV2953	K_Bacteria	P_Firmicutes	unclassified_P_Firmicutes	unclassified_P_Firmicutes	unclassified_P_Firmicutes	unclassified_P_Firmicutes	unclassified_P_Firmicutes
SV2954	K_Bacteria	P_Bacteroidota	C_Bacteroidia	O_Bacteroidales	F_Bacteroidaceae	G_Bacteroides	unclassified_G_Bacteroides
SV2955	K_Bacteria	P_Firmicutes	C_Bacilli	unclassified_C_Bacilli	unclassified_C_Bacilli	unclassified_C_Bacilli	unclassified_C_Bacilli
SV2956	K_Bacteria	P_Firmicutes	C_Clostridia	O_Peptostreptococcales-Tissierellales	unclassified_O_Peptostreptococcales-Tissierellales	unclassified_O_Peptostreptococcales-Tissierellales	unclassified_O_Peptostreptococcales-Tissierellales
SV2957	K_Bacteria	P_Firmicutes	C_Clostridia	O_Peptostreptococcales-Tissierellales	F_Peptostreptococcaceae	unclassified_F_Peptostreptococcaceae	unclassified_F_Peptostreptococcaceae
SV2958	K_Bacteria	P_Firmicutes	C_Bacilli	O_Lactobacillales	unclassified_O_Lactobacillales	unclassified_O_Lactobacillales	unclassified_O_Lactobacillales
SV2959	K_Bacteria	P_Bacteroidota	C_Bacteroidia	O_Bacteroidales	F_Muribaculaceae	unclassified_F_Muribaculaceae	unclassified_F_Muribaculaceae
SV2960	K_Bacteria	P_Bacteroidota	C_Bacteroidia	O_Bacteroidales	F_Muribaculaceae	unclassified_F_Muribaculaceae	unclassified_F_Muribaculaceae
SV2961	K_Bacteria	P_Firmicutes	C_Clostridia	O_Peptostreptococcales-Tissierellales	F_Peptostreptococcaceae	unclassified_F_Peptostreptococcaceae	unclassified_F_Peptostreptococcaceae
SV2962	K_Bacteria	P_Firmicutes	C_Clostridia	O_Peptostreptococcales-Tissierellales	unclassified_O_Peptostreptococcales-Tissierellales	unclassified_O_Peptostreptococcales-Tissierellales	unclassified_O_Peptostreptococcales-Tissierellales
SV2963	K_Bacteria	P_Bacteroidota	C_Bacteroidia	O_Bacteroidales	unclassified_O_Bacteroidales	unclassified_O_Bacteroidales	unclassified_O_Bacteroidales
SV2964	K_Bacteria	P_Firmicutes	C_Bacilli	O_Lactobacillales	F_Lactobacillaceae	unclassified_F_Lactobacillaceae	unclassified_F_Lactobacillaceae
SV2965	K_Bacteria	P_Firmicutes	C_Clostridia	unclassified_C_Clostridia	unclassified_C_Clostridia	unclassified_C_Clostridia	unclassified_C_Clostridia
SV2966	K_Bacteria	P_Firmicutes	C_Clostridia	O_Oscillospirales	unclassified_O_Oscillospirales	unclassified_O_Oscillospirales	unclassified_O_Oscillospirales
SV2967	K_Bacteria	P_Firmicutes	C_Bacilli	O_Lactobacillales	F_Lactobacillaceae	unclassified_F_Lactobacillaceae	unclassified_F_Lactobacillaceae
SV2968	K_Bacteria	P_Firmicutes	C_Clostridia	O_Peptostreptococcales-Tissierellales	unclassified_O_Peptostreptococcales-Tissierellales	unclassified_O_Peptostreptococcales-Tissierellales	unclassified_O_Peptostreptococcales-Tissierellales
SV2969	K_Bacteria	P_Firmicutes	C_Clostridia	O_Oscillospirales	F_Ruminococcaceae	G_Ruminococcus	unclassified_G_Ruminococcus
SV2970	K_Bacteria	P_Firmicutes	C_Bacilli	O_Lactobacillales	F_Lactobacillaceae	unclassified_F_Lactobacillaceae	unclassified_F_Lactobacillaceae
SV2971	K_Bacteria	P_Firmicutes	C_Bacilli	O_Lactobacillales	F_Lactobacillaceae	unclassified_F_Lactobacillaceae	unclassified_F_Lactobacillaceae
SV2972	K_Bacteria	P_Firmicutes	C_Clostridia	unclassified_C_Clostridia	unclassified_C_Clostridia	unclassified_C_Clostridia	unclassified_C_Clostridia
SV2973	K_Bacteria	P_Firmicutes	C_Clostridia	unclassified_C_Clostridia	unclassified_C_Clostridia	unclassified_C_Clostridia	unclassified_C_Clostridia
SV2974	K_Bacteria	P_Bacteroidota	C_Bacteroidia	O_Bacteroidales	F_Muribaculaceae	unclassified_F_Muribaculaceae	unclassified_F_Muribaculaceae
SV2975	K_Bacteria	P_Firmicutes	C_Bacilli	O_Lactobacillales	F_Lactobacillaceae	unclassified_F_Lactobacillaceae	unclassified_F_Lactobacillaceae
SV2976	K_Bacteria	P_Firmicutes	C_Clostridia	O_Lachnospirales	F_Lachnospiraceae	unclassified_F_Lachnospiraceae	unclassified_F_Lachnospiraceae
SV2977	K_Bacteria	P_Firmicutes	C_Bacilli	O_Lactobacillales	unclassified_O_Lactobacillales	unclassified_O_Lactobacillales	unclassified_O_Lactobacillales
