# Supplementary material for: The Influence of Omega‐3 Fatty Acids and Probiotics on Hippocampal Inflammation and Glial Cells in a Chronic Anorexia Nervosa Rat Model
Source: Int J Eat Disord. 2025 Oct 18;59(2):260–75. doi: 10.1002/eat.24574 (PMC12884241; doi:10.1002/eat.24574)
Supplement: Supplementary file 14 — Table S5: Results of Chao1 and Shannon index. [file EAT-59-260-s003.docx]

|  | **Sum Sq** | **Mean Sq** | **NumDF** | **DenDF** | **F value** | **Pr(>F)** |  |
| --- | --- | --- | --- | --- | --- | --- | --- |
| **Chao1_T1** | 1452,8 | 1452,8 | 1 | 38,397 | 1,7731 | 0,19085 |  |
| **Chao1_T2** | 93,3 | 93,3 | 1 | 37,616 | 0,1139 | 0,73767 |  |
| **batch** | 1657,2 | 207,1 | 8 | 37,607 | 0,2528 | 0,977 |  |
| **ABA** | 4763,8 | 4763,8 | 1 | 36,285 | 5,8141 | 0,02108 | * |
| **time** | 7985,1 | 2661,7 | 3 | 138,203 | 3,2485 | 0,02386 | * |
| **ABA:time** | 9224,5 | 3074,8 | 3 | 138,203 | 3,7527 | 0,0125 | * |
|  |  |  |  |  |  |  |  |
|  | **Sum Sq** | **Mean Sq** | **NumDF** | **DenDF** | **F value** | **Pr(>F)** |  |
| **Chao1_T1** | 1484,9 | 1484,9 | 1 | 35,429 | 1,9427 | 0,172054 |  |
| **Chao1_T2** | 578,1 | 578,1 | 1 | 34,451 | 0,7564 | 0,390471 |  |
| **batch** | 1140 | 142,5 | 8 | 34,709 | 0,1864 | 0,991235 |  |
| **group** | 6612,1 | 2204 | 3 | 34,442 | 2,8837 | 0,049736 | * |
| **time** | 21438,4 | 7146,1 | 3 | 132,28 | 9,3497 | 1,20E-05 | *** |
| **group:time** | 21405,4 | 2378,4 | 9 | 132,174 | 3,1118 | 0,001999 | ** |
|  |  |  |  |  |  |  |  |
|  | **Sum Sq** | **Mean Sq** | **NumDF** | **DenDF** | **F value** | **Pr(>F)** |  |
| **Shannon_T1** | 0,1399 | 0,13987 | 1 | 36,544 | 1,4327 | 0,239 |  |
| **Shannon_T2** | 0,0005 | 0,00052 | 1 | 39,116 | 0,0053 | 0,9424 |  |
| **batch** | 0,6178 | 0,07722 | 8,00E+00 | 35,961 | 0,791 | 0,6139 |  |
| **group** | 3,5205 | 1,17349 | 3,00E+00 | 36,276 | 12,0203 | 1,30E-05 | *** |
| **time** | 2,6177 | 0,87256 | 3 | 133,819 | 8,9378 | 1,94E-05 | *** |
| **group:time** | 1,3449 | 0,14943 | 9 | 133,616 | 1,5307 | 0,1433 |  |
|  |  |  |  |  |  |  |  |
|  | **Sum Sq** | **Mean Sq** | **NumDF** | **DenDF** | **F value** | **Pr(>F)** |  |
| **Shannon_T1** | 0,1929 | 0,1929 | 1 | 38,695 | 1,913 | 0,1745567 |  |
| **Shannon_T2** | 0,0044 | 0,0044 | 1 | 40,081 | 0,0441 | 0,8348055 |  |
| **batch** | 0,6602 | 0,0825 | 8 | 38,232 | 0,8182 | 0,5913621 |  |
| **ABA** | 3,7244 | 3,7244 | 1 | 36,906 | 36,9277 | 4,99E-07 | *** |
| **time** | 1,7853 | 0,5951 | 3 | 139,138 | 5,9005 | 8,06E-04 | *** |
| **ABA:time** | 0,3721 | 0,124 | 3 | 139,138 | 1,2298 | 0,3012723 |  |

**Table S5**
