# Supplementary material for: The Influence of Omega‐3 Fatty Acids and Probiotics on Hippocampal Inflammation and Glial Cells in a Chronic Anorexia Nervosa Rat Model
Source: Int J Eat Disord. 2025 Oct 18;59(2):260–75. doi: 10.1002/eat.24574 (PMC12884241; doi:10.1002/eat.24574)
Supplement: Supplementary file 15 — Table S6: Results of mediation analysis. [file EAT-59-260-s001.docx]

|  | **number of sign taxa** | **significant taxa (colored wells mean potential mediation based on the mediation model in lavaan; green p<0.05, yellow p<0.1)** | | | | | | | | | | | | | | | | | | | | | | | | | | | | |
| --- | --- | --- | --- | --- | --- | --- | --- | --- | --- | --- | --- | --- | --- | --- | --- | --- | --- | --- | --- | --- | --- | --- | --- | --- | --- | --- | --- | --- | --- | --- |
| **qPCR Aif1** | 11 | SV54 P= 0.356 | SV309 P= 0.144 | SV123 P= 0.885 | SV68 P= 0.573 | SV1 P= 0.527 | SV169 P= 0.755 | SV182 P= 0.576 | SV150 P= 0.567 | SV76 P= 0.425 | SV80 P= 0.512 | SV653 P= 0.085 |  |  |  |  |  |  |  |  |  |  |  |  |  |  |  |  |  |  |
| **qPCR Tnf** | 11 | SV106 P= 0.928 | SV197 P= 0.361 | SV273 P= 0.919 | SV54 P= 0.407 | SV653 P= 0.048 | SV80 P= 0.71 | SV25 P= 0.712 | SV113 P= 0.696 | SV182 P= 0.857 | SV51 P= 0.678 | SV263 P= 0.225 |  |  |  |  |  |  |  |  |  |  |  |  |  |  |  |  |  |  |
| **qPRC Il6** | 10 | SV54 P= 0.246 | SV197 P= 0.568 | SV106 P= 0.954 | SV273 P= 0.993 | SV34 P= 0.344 | SV80 P= 0.739 | SV149 P= 0.426 | SV69 P= 0.807 | SV361 P= 0.049 | SV182 P= 0.301 |  |  |  |  |  |  |  |  |  |  |  |  |  |  |  |  |  |  |  |
| **qPCR Bdnf** | 5 | SV228 P= 0.281 | SV96 P= 0.079 | SV173 P= 0.711 | SV265 P= 0.178 | SV30 P= 0.914 |  |  |  |  |  |  |  |  |  |  |  |  |  |  |  |  |  |  |  |  |  |  |  |  |
| **mean bodyweight between days 1 and 8** | 29 | SV19 P= 0.615 | SV26 P= 0.597 | SV207 P= 0.52 | SV103 P= 0.973 | SV96 P= 0.964 | SV61 P= 0.653 | SV73 P= 0.899 | SV597 P= 0.693 | SV34 P= 0.568 | SV146 P= 0.479 | SV104 P= 0.65 | SV200 P= 0.866 | SV288 P= 0.69 | SV219 P= 0.506 | SV2 P= 0.54 | SV224 P= 0.608 | SV20 P= 0.715 | SV99 P= 0.621 | SV442 P= 0.956 | SV113 P= 0.672 | SV24 P= 0.87 | SV82 P= 0.635 | SV116 P= 0.985 | SV5 P= 0.287 | SV79 P= 0.902 | SV134 P= 0.869 | SV32 P= 0.698 | SV456 P= 0.741 | SV95 P= 0.594 |
| **mean bodyweight between days 9 and 14 (excluding K)** | 0 |  |  |  |  |  |  |  |  |  |  |  |  |  |  |  |  |  |  |  |  |  |  |  |  |  |  |  |  |  |
| **mean bodyweight between days 15 and 35 (excluding K)** | 22 | SV597 P= 0.973 | SV2 P= 0.971 | SV219 P= 0.745 | SV7 P= 0.905 | SV19 P= 0.876 | SV20 P= 0.985 | SV26 P= 0.99 | SV32 P= 0.783 | SV43 P= 0.763 | SV96 P= 0.575 | SV146 P= 0.938 | SV200 P= 0.526 | SV207 P= 0.913 | SV231 P= 0.537 | SV773 P= 0.946 | SV442 P= 0.728 | SV16 P= 0.726 | SV34 P= 0.945 | SV104 P= 0.902 | SV456 P= 0.845 | SV103 P= 0.73 | SV174 P= 0.897 |  |  |  |  |  |  |  |
| **IHMC IBA1** | 9 | SV146 P= 0.001 | SV348 P= 0.239 | SV62 P= 0.717 | SV18 P= 0.32 | SV300 P= 0.114 | SV112 P= 0.005 | SV309 P= 0.053 | SV61 P= 0.641 | SV78 P= 0.605 |  |  |  |  |  |  |  |  |  |  |  |  |  |  |  |  |  |  |  |  |
| **IHMC MAP2** | 5 | SV205 P= 0.424 | SV40 P= 0.044 | SV150 P= 0.667 | SV52 P= 0.852 | SV273 P= 0.172 |  |  |  |  |  |  |  |  |  |  |  |  |  |  |  |  |  |  |  |  |  |  |  |  |
|  |  |  |  |  |  |  |  |  |  |  |  |  |  |  |  |  |  |  |  |  |  |  |  |  |  |  |  |  |  |  |
| **GENUS** |  |  |  |  |  |  |  |  |  |  |  |  |  |  |  |  |  |  |  |  |  |  |  |  |  |  |  |  |  |  |
| **qPCR Aif1** | 3 | SV182 P= 0.531 | SV653 P= 0.24 | SV1 P= 0.321 |  |  |  |  |  |  |  |  |  |  |  |  |  |  |  |  |  |  |  |  |  |  |  |  |  |  |
| **qPCR Tnf** | 1 | SV54 P=0.001 |  |  |  |  |  |  |  |  |  |  |  |  |  |  |  |  |  |  |  |  |  |  |  |  |  |  |  |  |
| **qPRC Il6** | 0 |  |  |  |  |  |  |  |  |  |  |  |  |  |  |  |  |  |  |  |  |  |  |  |  |  |  |  |  |  |
| **qPCR Bdnf** | 1 | SV473 P= 0.374 |  |  |  |  |  |  |  |  |  |  |  |  |  |  |  |  |  |  |  |  |  |  |  |  |  |  |  |  |
| **mean bodyweight between days 1 and 8** | 7 | SV19 P= 0.023 | SV99 P= 0.463 | SV353 P= 0.246 | SV57 P= 0.824 | SV24 P= 0.187 | SV501 P= 0.015 |  |  |  |  |  |  |  |  |  |  |  |  |  |  |  |  |  |  |  |  |  |  |  |
| **mean bodyweight between days 9 and 14 (excluding K)** | 2 | SV99 P= 0.255 | SV19 P= 0.324 |  |  |  |  |  |  |  |  |  |  |  |  |  |  |  |  |  |  |  |  |  |  |  |  |  |  |  |
| **mean bodyweight between days 15 and 35 (excluding K)** | 4 | SV99 P= 0.562 | SV19 P= 0.352 | SV442 P= 0.035 | SV773 P= 0.209 | |  |  |  |  |  |  |  |  |  |  |  |  |  |  |  |  |  |  |  |  |  |  |  |  |
| **IHMC IBA1** | 2 | SV18 P= 0.03 | SV342 P= 0.051 |  |  |  |  |  |  |  |  |  |  |  |  |  |  |  |  |  |  |  |  |  |  |  |  |  |  |  |
| **IHMC MAP2** | 2 | SV501 P= 0.017 | SV168 P= 0.169 |  |  |  |  |  |  |  |  |  |  |  |  |  |  |  |  |  |  |  |  |  |  |  |  |  |  |  |
|  |  |  |  |  |  |  |  |  |  |  |  |  |  |  |  |  |  |  |  |  |  |  |  |  |  |  |  |  |  |  |
| **FAMILY** |  |  |  |  |  |  |  |  |  |  |  |  |  |  |  |  |  |  |  |  |  |  |  |  |  |  |  |  |  |  |
| **qPCR Aif1** | 0 |  |  |  |  |  |  |  |  |  |  |  |  |  |  |  |  |  |  |  |  |  |  |  |  |  |  |  |  |  |
| **qPCR Tnf** | 1 | SV120 P= 0.055 |  |  |  |  |  |  |  |  |  |  |  |  |  |  |  |  |  |  |  |  |  |  |  |  |  |  |  |  |
| **qPRC Il6** | 0 |  |  |  |  |  |  |  |  |  |  |  |  |  |  |  |  |  |  |  |  |  |  |  |  |  |  |  |  |  |
| **qPCR Bdnf** | 0 |  |  |  |  |  |  |  |  |  |  |  |  |  |  |  |  |  |  |  |  |  |  |  |  |  |  |  |  |  |
| **mean bodyweight between days 1 and 8** | 6 | SV19 P= 0.008 | SV99 P= 0.284 | SV353 P= 0.231 | SV6 P= 0.763 | SV51 P= 0.853 | SV316 P= 0.017 |  |  |  |  |  |  |  |  |  |  |  |  |  |  |  |  |  |  |  |  |  |  |  |
| **mean bodyweight between days 9 and 14 (excluding K)** | 3 | SV99 P= 0.201 | SV19 P= 0.333 | SV353 P= 0.038 |  |  |  |  |  |  |  |  |  |  |  |  |  |  |  |  |  |  |  |  |  |  |  |  |  |  |
| **mean bodyweight between days 15 and 35 (excluding K)** | 3 |  |  |  |  |  |  |  |  |  |  |  |  |  |  |  |  |  |  |  |  |  |  |  |  |  |  |  |  |  |
| **IHMC IBA1** | 1 | SV18 P= 0.017 |  |  |  |  |  |  |  |  |  |  |  |  |  |  |  |  |  |  |  |  |  |  |  |  |  |  |  |  |
| **IHMC MAP2** |  |  |  |  |  |  |  |  |  |  |  |  |  |  |  |  |  |  |  |  |  |  |  |  |  |  |  |  |  |  |

**Table S6**
